# The atmospheric relevance of primary alcohols and imidogen reactions

**DOI:** 10.1038/s41598-023-35473-1

**Published:** 2023-06-05

**Authors:** Hamed Douroudgari, Hadi Zarepour, Morteza Vahedpour, Mahdi Jaberi, Mahdi Zarepour

**Affiliations:** Department of Chemistry, University of Zanjan, PO Box 38791-45371, Zanjan, Iran

**Keywords:** Environmental chemistry, Atmospheric chemistry

## Abstract

Organic alcohols as very volatile compounds play a crucial role in the air quality of the atmosphere. So, the removal processes of such compounds are an important atmospheric challenge. The main goal of this research is to discover the atmospheric relevance of degradation paths of linear alcohols by imidogen with the aid of simulation by quantum mechanical (QM) methods. To this end, we combine broad mechanistic and kinetic results to get more accurate information and to have a deeper insight into the behavior of the designed reactions. Thus, the main and necessary reaction pathways are explored by well-behaved QM methods for complete elucidation of the studying gaseous reactions. Moreover, the potential energy surfaces as  a main factor are computed for easier judging of the most probable pathways in the simulated reactions. Our attempt to find the occurrence of the considered reactions in the atmospheric conditions is completed by precisely evaluating the rate constants of all elementary reactions. All of the computed bimolecular rate constants have a positive dependency on both temperature and pressure. The kinetic results show that H-abstraction from the α carbon is dominant relative to the other sites. Finally, by the results of this study, we conclude that at moderate temperatures and pressures primary alcohols can degrade with imidogen, so they can get atmospheric relevance.

## Introduction

The atmospheric chemistry of alcohols is a significant global subject in the context of urban and regional air quality analysis due to their wide range of usages^1^. It has been an increased request for renewable biofuels (that are alcohol-based fuels) instead of unsustainable fossil fuels^2^. Because air pollution and climate change due to the explosive growth of the automobile industry and transportation that accelerates the consumption of fossil fuels in large quantities are global concerns^3–5^. On the other hand, to achieve efficient and clean combustion, the use of alternative fuels and the fuels derived from biomass which have a wide variation in their physicochemical properties carriages vast technical challenges^6–9^.

In the atmosphere and interstellar space, the most predominant non-methane organic species is methanol^10–12^, which is the main source of tropospheric CO^13^ and formaldehyde^14^, and also has small action in the tropical HOx and ozone budgets^15,16^. Methanol is emitted into the atmosphere by several sources such as vegetation^17,18^, plant growth^19,20^, biomass burning, biofuels^21,22^, plant matter decay^23,24^, and human activities in urban and industrial regions^25^. It is better to say that in the atmosphere, methane is generated by CH_3_O_2_ (peroxy radical) reactions^26,27^. The biogenic emissions of methanol have a particularly important influence on rainforest ecosystems, so they play a pivotal role in the tropospheric chemistry^28^.

Understanding the atmospheric cycle of ethanol is an important task in combustion chemistry^29^. Because it is a biogenic volatile organic compound that is highly used as a fuel for motor vehicles^30^. The same as methanol, ethanol is released into the atmosphere by different sources including vegetation and during biomass combustion. Also, various urban and industrial processes have an impact on the concentration of atmospheric ethanol^31^. The portion of ethanol that derives from biomass (bio-ethanol) is nowadays being developed as a renewable fuel that will decrease support for fossil fuels and global warming^32,33^. Moreover, there exists a beneficial profit in ethanol-utilizing as fuel for human health because of air quality^34,35^. Despite the mentioned crucial roles of ethanol that increase over time, atmospheric reports are rather sparse and the global sources and sinks for this compound are not comprehended in detail^32,36^.

Another vital alcohol is n-propanol which is considerably less toxic and less volatile than methanol^37^. Thus, it may be a desirable case for developing biofuels, but there are many discussions about that. It is well known that n-propanol serves high octane numbers and has lower corrosion than ethanol^38^. Despite these advantages, it cannot create a high energy density the same as ethanol. To resolve this issue, n-propanol in pure form may not be used as the ideal biofuel. Thus, propanol-based biofuel contains a considerable alcohol mixture^39^. These are the main ways for the emission of n-propanol into the atmosphere.

Among the biofuels and other alternative fuels, the use of n-butanol which is called biobutanol has increased remarkably in prominence over the past years^40–42^. This considerable attention is related to the nontoxic nature, low vapor pressure, and high-energy content of this compound. Also, butanol is more favorable than ethanol as biofuel^43^. Some investigations proved that in direct-injection spark-ignition engines, biobutanol acts fine in comparison with ethanol and consumes less fuel^44^. Also, it has been shown that *n*-butanol in addition to automobile fuel can be used as jet fuel after some changes^45,46^. From a combustion chemistry viewpoint, major reactions of n-butanol are associated with the hydrogen atom abstraction from different centers in the consumption pathway of high-temperature fuels^47,48^.

Up to now, we have spoken about the origins of releasing alcohol. Here, we briefly discuss the sinks of these species in the air. It is well affirmed that gaseous hydroxyl radical has high reactivity^49,50^ and concentration^51,52^ than all present active species in our ambient. So, this radical plays a crucial role in the removal processes of atmospheric pollutants. In addition, many scientific reports proved that atmospheric compounds react preferably with hydroxyl radicals. This statement is true for organic alcohols as well^10,53–58^. Accordingly, a literature survey shows that the gas phase reactions of alcohols have been widely considered by hydroxyl radicals both experimentally and theoretically^11,59–68^. Besides, the previously conducted theoretical studies for alcohols plus OH radicals revealed that the most favorable degradation pathways are the H abstraction reactions due to having small barriers energetically^47,48,69–71^. Therefore, hydroxyl radicals as a key atmospheric scavenger are the main sink for alcohols in the air. Also, it has been proved that other species such as HO_2_^72–75^, O^76–81^, O_2_^82–84^, O_3_^85–88^, N^89,90^, H^91–93^, F^94–98^, Cl^95,99–102^, NH^103–106^, NH_2_^107,108^, NO^109,110^, H_2_O^111,112^, NO_2_^109,113,114^, NO_3_^109,115–118^, CH_3_^72,119,120^ and Criegee intermediates^121,122^ have good contributions in eliminating atmospheric pollutants. Therefore, we must remember this point in mind that the role of other oxidants like NH can not be ignored because, in the absence of hydroxyl radicals, the principal task of these species is the elimination of pollutants from the air of living environments.

In this study, we will follow the reactions of the most volatile linear organic alcohols with an active atmospheric species, NH radical, by QM methods in the triplet state. The reason is to find the atmospheric relevance of linear alcohols in reaction with NH. It will be made clear, how the degradation of linear alcohols happens by imidogen in the atmosphere. The response to this question is our attempt in the current exploration, which will be helped to find a solution to a global atmospheric challenge (air quality). To have useful and wide information about the atmospheric chemistry of alcohols, four simple series including methanol, ethanol, *n*-propanol, and *n*-butanol will be examined. The most reactive center in the considered series will be discovered and discussed by well-known theories. The roles of thermodynamics and kinetics results will be evaluated in that relevance. And, the atoms-in-molecules theory (AIM) will be utilized to confirm the role of intermolecular interactions in the stability of fragments in stationary points. By using the DFT-M06-2X and CBS-QB3 levels, the paths and the change of energy of those paths for the H abstraction reactions of each alcohol will be calculated to construct the PESs of the simulated gas-phase reactions. The RRKM and TST/Eckart theories will be used to compute the temperature dependent and pressure-dependent rate constants of the considered elementary bimolecular reactions.

## Results and discussion

We begin to discuss details of four reactions such as CH_3_OH + NH, C_2_H_5_OH + NH, *n*-C_3_H_7_OH + NH, and *n*-C_4_H_9_OH + NH in the gas phase by QM methods. It should be noted the mean unsigned error for the M06-2X and the CBS-QB3 methods in the reactions of methanol + NH and ethanol + NH are calculated and listed in Tables 1 and 4, respectively. The results prove that the CBS-QB3 method has the closest energies to the predicted ones by the more accurate QM method, W1BD. Therefore, throughout the paper all energetics and rate constants are at the CBS-QB3 method otherwise, we mention the level of computation.

**Table 1:** The computed relative energies for stationary points of the CH_3_OH + NH reaction (Unit of all numbers is kcal mol^−1^).

Species	∆E (0 K) (W1BD)	∆E (0 K) (CBS-QB3)	∆ (E + ZPE) (M06-2X)	MUE^1^	MUE^2^	MUE^3^
R(m)	0.00	0.00	0.00	0.00	0.00	0.00
CR1(m)	− 2.10	− 0.94	− 2.95	0.00	1.16	0.85
CR2(m)	− 2.20	− 2.13	− 2.95	0.00	0.06	0.75
TS1(m)	17.67	16.63	14.50	0.00	1.04	3.17
TS2(m)	14.60	13.62	12.49	0.00	0.98	2.11
CP1(m)	9.76	10.04	9.10	0.00	0.28	0.66
CP2(m)	− 2.65	− 2.13	− 1.51	0.00	0.52	1.14
P1(m) (CH_3_O + NH_2_)	11.58	11.73	12.05	0.00	0.16	0.47
P2(m) (CH_2_OH + NH_2_)	2.52	3.20	5.08	0.00	0.68	2.56

MUE^1^, MUE^2^, and MUE^3^ are the mean unsinged errors for the W1BD, CBS-QB3, and M06-2X methods, respectively*.*

Since for the long-chain molecules, there are lots of conformers for reactants and transition states^122^, throughout the paper, we will discuss the reactions of more stable conformers for n-propanol and n-butanol to locate the global minimum of reactants and transition states. Reactions of other conformers are found in the supplementary material.

### Methanol plus NH reaction

**Figure 1:**
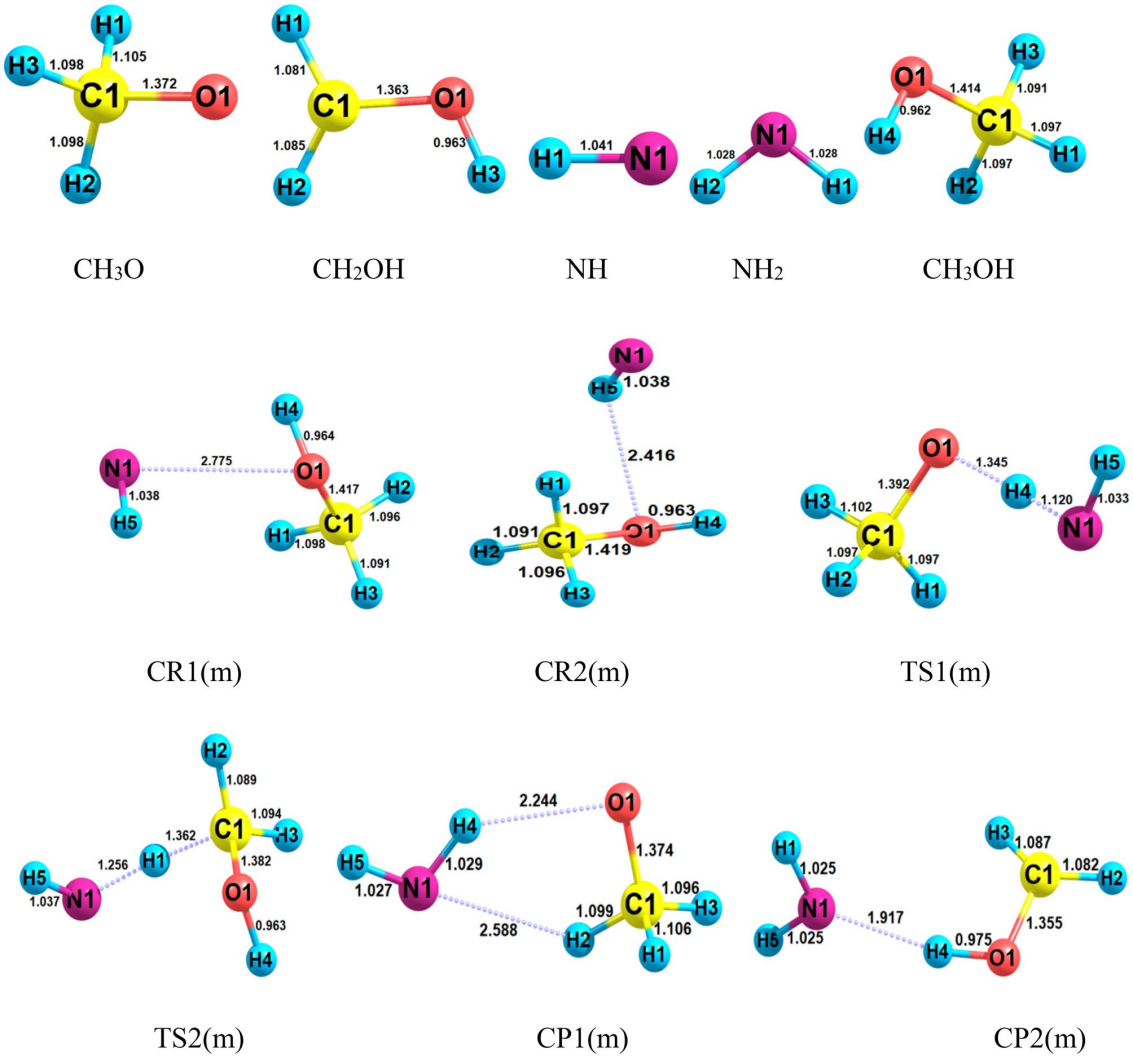
Structures of all stationary points including bond lengths (in Angstrom) in the CH_3_OH + NH reaction calculated at the M06-2X method.

**Figure 2:**
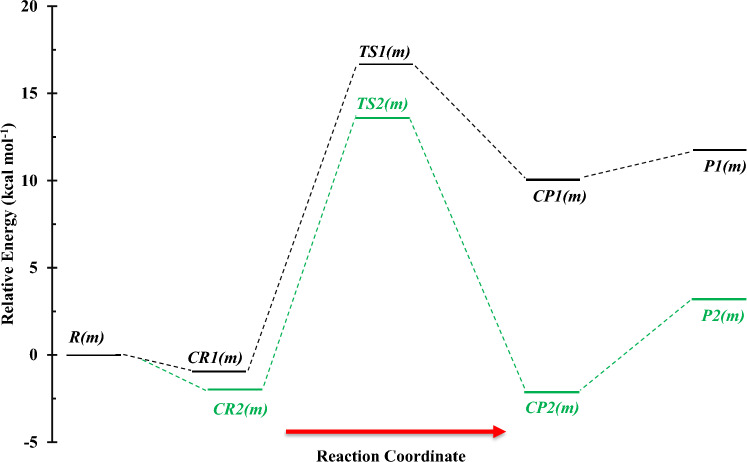
Potential energy surface of the CH_3_OH + NH reaction at the triplet ground state computed by the CBS-QB3 level.

Figures 1 and 2 display the structures of involved stationary points and PES profile, respectively, in the gas phase reaction of methanol with NH at the triplet state. The most probable initiation paths for the CH_3_OH + NH reaction are H atom abstraction pathways. Also, Tables 1 and 2 contain the relative and thermodynamic energies. The possible pathways in the CH_3_OH + NH reaction that causes to generate of two different products are summarized as follows:

$$R\left( m \right) \to CR1\left( m \right) \to TS1\left( m \right) \to CP1\left( m \right) \to P1\left( m \right) \, (CH_{3} O + \, NH_{2} )\quad \left( {{\text{R}}_{{1}} \left( {\text{m}} \right)} \right)$$

$$R\left( m \right) \to CR2\left( m \right) \to TS2\left( m \right) \to CP2\left( m \right) \to P2\left( m \right) \, (CH_{2} OH + \, NH_{2} )\quad \left( {{\text{R}}_{{2}} \left( {\text{m}} \right)} \right)$$

**Table 2:** Thermodynamic parameters for stationary points of the CH_3_OH + NH reaction (Unit of all numbers is kcal mol^−1^).

Species	*∆E˚*(*A*)	*∆E˚*(*B*)	*∆E˚*(*C*)	*∆H˚*(*A*)	*∆H˚*(*B*)	*∆H˚*(*C*)	*∆G˚*(*A*)	*∆G˚*(*B*)	*∆G˚*(*C*)	*T∆S˚*(*A*)	*T∆S˚*(*B*)	*T∆S˚*(*C*)
R(m)	0.00	0.00	0.00	0.00	0.00	0.00	0.00	0.00	0.00	0.00	0.00	0.00
CR1(m)	− 2.67	− 0.39	− 1.44	− 3.26	− 0.98	− 2.03	3.76	5.06	3.44	− 7.02	− 6.03	− 5.47
CR2(m)	− 2.63	− 1.67	− 1.69	− 3.22	− 2.26	− 2.28	3.67	3.63	3.24	− 6.90	− 5.89	− 5.52
TS1(m)	14.27	16.40	17.43	13.68	15.81	16.84	21.64	24.02	24.87	− 7.96	− 8.21	− 8.02
TS2(m)	12.17	13.43	14.40	11.58	12.83	13.81	20.00	20.82	21.62	− 8.42	− 7.99	− 7.81
CP1(m)	9.69	10.69	10.52	9.10	10.10	9.93	15.49	16.13	15.51	− 6.40	− 6.02	− 5.59
CP2(m)	− 1.43	− 1.45	− 1.91	− 2.02	− 2.04	− 2.50	5.59	3.87	3.19	− 7.61	− 5.91	− 5.69
P1(m) (CH_3_O + NH_2_)	12.15	11.88	11.68	12.15	11.88	11.68	11.22	10.96	10.81	0.94	0.92	0.88
P2(m) (CH_2_OH + NH_2_)	5.47	3.51	2.84	5.47	3.51	2.84	4.26	2.37	1.71	1.21	1.14	1.12

A, B, and C refer to the M06-2X, CBS-QB3, and W1BD methods, respectively.

Pre-reactive complexes (CRs) are key stationary points because they are starting point of many of the gas phase reactions. The intermolecular interactions have a crucial role in CR formation due to stabilization. Thus, we study CRs formation from thermodynamic and AIM theory viewpoints. Through a collision of the reactants, two pre-reactive complexes, *CR1*(*m*), and *CR2*(*m*) are formed. The *CR1*(*m*) due to a van der Waals interaction between 1O and 1N atoms ($\rho_{(LCP)} =$ 0.0131 e bohr^−3^ and $\nabla^{2} \rho_{(LCP)} =$ 0.0458 e bohr^−5^) with a bond distance of 2.775 Å has small relative stability (− 0.94 kcal/mol) compared to *CR2*(*m*). In the *CR2*(*m*) complex, the hydrogen bond between the H atom of the OH group and the N atom ($\rho_{(LCP)} =$ 0.0135 e bohr^−3^ and $\nabla^{2} \rho =$ 0.0504 e bohr^−5^) with a length of 2.416 Å leads to the stability of − 2.085 kcal mol^−1^. For the formation of *CR1*(*m*), the calculated enthalpy and Gibbs free energy changes (in standard conditions) are − 0.98 and 5.06 kcal/mol, respectively. The same parameters, *ΔH*^*0*^ and *ΔG*^*0*^, for *CR2*(*m*) are − 2.26 and 3.63 kcal/mol, respectively. These results confirm that the starting point for the title reaction is an exothermic and non-spontaneous step. For *CR1*(*m*) and *CR2*(*m*), the temperature dependent equilibrium constants in the range of 300–3000 K are listed in Supplementary Table S9. The following expressions are extracted by fitting the calculated values of the equilibrium constants to the expression $A\left( \frac{T}{300} \right){}^{m}\exp \left( {\frac{\Delta E}{{RT}}} \right)$.

1 $$K_{1A} = 7.15 \times 10^{ - 26} \left( \frac{T}{300} \right){}^{2.42 \pm 0.01}\exp \left[ {\frac{{(4.16 \pm 0.01)\,{\text{kcal}}\,{\text{mol}}^{ - 1} }}{RT}} \right],$$

2 $$K_{1B} = 3.69 \times 10^{ - 25} \left( \frac{T}{300} \right){}^{2.44 \pm 0.00}\exp \left[ {\frac{{(1.88 \pm 0.01){\text{kcal}}\,{\text{mol}}^{ - 1} }}{RT}} \right],$$

3 $$K_{2A} = 8.94 \times 10^{ - 26} \left( \frac{T}{300} \right){}^{2.43 \pm 0.01}\exp \left[ {\frac{{(4.12 \pm 0.01){\text{kcal}}\,{\text{mol}}^{ - 1} }}{RT}} \right],$$

4 $$K_{2B} = 4.60 \times 10^{ - 25} \left( \frac{T}{300} \right){}^{2.42 \pm 0.01}\exp \left[ {\frac{{(3.18 \pm 0.01){\text{kcal}}\,{\text{mol}}^{ - 1} }}{RT}} \right].$$

**Figure 3:**
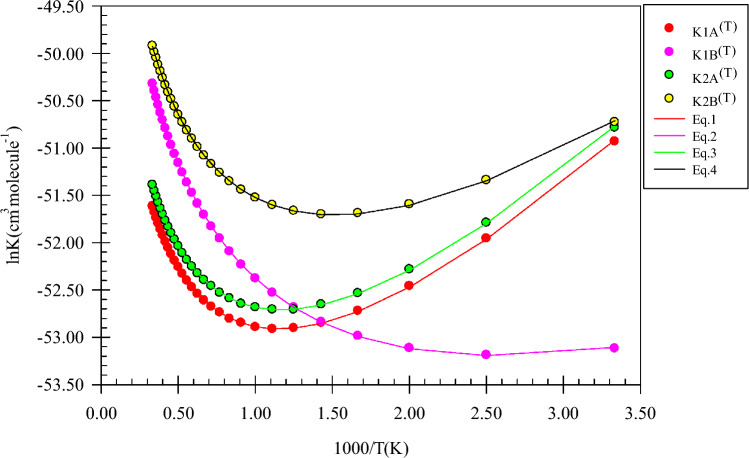
Equilibrium constants and associated fitted expressions of all prereactive complexes in the methanol plus imidogen reaction calculated at the M06-2X (**A**) and CBS-QB3 (**B**) methods.

As shown in Fig. 3, by increasing temperature, the equilibrium constants of the *CR1*(*m*) and *CR2*(*m*) computed by the CBS-QB3 method are decreased up to 800 K and after that, they are increased. For *CR1*(*m*), the obtained equilibrium constant at the M06-2X method has a similar treatment but for *CR2*(*m*), after 800 K a constant value with smooth growth is forecast. The results of the CBS-QB3 method show that the equilibrium constant of *CR2*(*m*) is 19.00, 2.34, and 1.49 times higher than *CR1*(*m*) at 300, 1000, and 3000 K.

The first path of methanol plus NH radical is highly endothermic (12.15 kcal mol^−1^ at the M06-2X method and 11.88 kcal mol^−1^ at the CBS-QB3 method) at the standard condition and has a higher energy barrier in comparison with the second pathway. In this path, NH moiety in the pre-reactive complex *CR1*(*m*) due to having a suitable orientation gets a hydrogen atom from the hydroxyl group by surmounting *TS1*(*m*) with an energy barrier of 17.61 kcal mol^−1^ at the CBS-QB3 level. This value is just 0.18 kcal mol^−1^ higher than the respective value computed by the M06-2X method. The optimized structure for *TS1*(*m*) shows that the H4 atom is located between O1 and N1 atoms (O1…H4…N1) with bond lengths of 1.345 Å and 1.120 Å, respectively. The second pathway (R_2_(m)) which has a lower barrier height is the H abstraction reaction from the methyl group. So, this is a more feasible reaction than R1(m). The difference between the energy barriers of *TS1*(*m*) and *TS2*(*m*) are 1.88 and 1.99 kcal mol^−1^ at the CBS-QB3 and M06-2X methods, respectively. The structure of *TS2*(*m*) accompanied by the imaginary frequency along the reaction coordinate in both methods displays that the H1 atom is transferring from the methyl group to imidogen moiety. Also, IRC calculations of *TS1*(*m*) and *TS2*(*m*) confirm the existence of the discussed pathways. The exit channels of R_1_(m) and R_2_(m) paths are met with the post-reactive complexes of *CP1*(*m*) and *CP2*(*m*), respectively. In the structure of the *CP1*(*m*), a five-membered ring structure ($\rho_{(RCP)} =$ 0.0077 e bohr^−3^ and $\nabla^{2} \rho_{(RCP)} =$ 0.0372 e bohr^−5^) is achieved. It contains a hydrogen bond between the atoms 1O and 4H ($\rho_{(LCP)} =$ 0.0153 e bohr^−3^ and $\nabla^{2} \rho_{(LCP)} =$ 0.0492 e bohr^−5^) and van der Waals bond between the atoms 1N and 2H ($\rho_{(LCP)} =$ 0.0087 e bohr^−3^ and $\nabla^{2} \rho_{(LCP)} =$ 0.0316 e bohr^−5^). And for the *CP2*(*m*) case, the AIM analysis demonstrates a van der Waals bond between the atoms 1N and 4H ($\rho_{(LCP)} =$ 0.0298 e bohr^−3^ and $\nabla^{2} \rho_{(LCP)} =$ 0.0849 e bohr^−5^).

**Figure 4:**
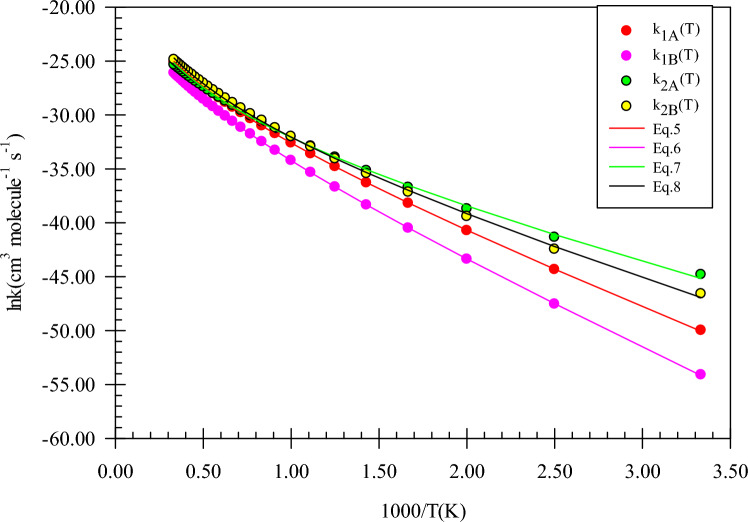
Graph of the high-pressure limit rate constants (cm^3^ molecule^−1^ s^−1^) and fitted non-Arrhenius expressions for the gas-phase formation of the *P1*(*m*) and *P2*(*m*) products calculated by the TST/Eckart theory at the M06-2X (**A**) and CBS-QB3 (**B**) methods.

The computed rate constants at the M06-2X (A) and CBS-QB3 (B) methods are fitted in the non-Arrhenius rate equation and depicted in Fig. 4. The following expressions are extracted in the 300–3000 K temperature range.

5 $$k_{1A} = 3.69 \times 10^{ - 14} \left( \frac{T}{300} \right){}^{3.39 \pm 0.03}\exp \left[ { - \frac{{(11.40 \pm 0.05){\text{kcal}}\,{\text{mol}}^{ - 1} }}{RT}} \right],$$

6 $$k_{1B} = 2.95 \times 10^{ - 14} \left( \frac{T}{300} \right){}^{3.26 \pm 0.02}\exp \left[ { - \frac{{(13.64 \pm 0.17){\text{kcal}}\,{\text{mol}}^{ - 1} }}{RT}} \right],$$

7 $$k_{2A} = 2.38 \times 10^{ - 15} \left( \frac{T}{300} \right){}^{4.19 \pm 0.13}\exp \left[ { - \frac{{(6.85 \pm 0.23){\text{kcal}}\,{\text{mol}}^{ - 1} }}{RT}} \right],$$

8 $$k_{2B} = 6.43 \times 10^{ - 15} \left( \frac{T}{300} \right){}^{4.07 \pm 0.12}\exp \left[ { - \frac{{(8.50 \pm 0.91){\text{kcal}}\,{\text{mol}}^{ - 1} }}{RT}} \right].$$

The fitted rate expression at the 300–3000 K temperature range for the *P1*(*m*) product formation is $2.95 \times 10^{ - 14} \left( \frac{T}{300} \right){}^{3.26}\exp \left( { - \frac{{13.64\,{\text{kcal}}\,{\text{mol}}^{ - 1} }}{RT}} \right)$ cm^3^ molecule^−1^ s^−1^ at the CBS-QB3 method. For this product, the rate constant predicted by the CBS-QB3 method is 10.06, 3.74, and 2.76 times higher than that of the M06-2X method at 600, 1500, and 3000 K (see Supplementary Table S13). So, the rate expression for *P1*(*m*) at the M06-2X method ($3.69 \times 10^{ - 14} \left( \frac{T}{300} \right){}^{3.39}\exp \left( { - \frac{{11.40\,{\text{kcal}}\,{\text{mol}}^{ - 1} }}{RT}} \right)$ cm^3^ molecule^−1^ s^−1^) has no meaningful difference from that of the CBS-QB3 method. As shown in Fig. 4, our computed rate constants in both theoretical methods demonstrate that the R_2_(m) path is kinetically dominant to the first path with the fitted rate expressions of $2.38 \times 10^{ - 15} \left( \frac{T}{300} \right){}^{4.19}\exp \left( { - \frac{{6.85\,{\text{kcal}}\,{\text{mol}}^{ - 1} }}{RT}} \right)$ and $6.43 \times 10^{ - 15} \left( \frac{T}{300} \right){}^{4.07}\exp \left( { - \frac{{8.50\,{\text{kcal}}\,{\text{mol}}^{ - 1} }}{RT}} \right)$ cm^3^ molecule^-1^ s^-1^ at the CBS-QB3 and M06-2X methods, respectively. Also, for this path, the ratio of calculated rate constants by both methods k(T)_CBS-QB3_/k(T)_M06-2X_ is 0.64, 1.28, and 1.59 at 600, 1500, and 3000 K.

In Table 3, the calculated branching ratios for the methanol and NH reaction are listed. Comparing branching ratios of the *P1*(*m*) and *P2*(*m*) reveals that in the atmospheric condition, methanol degradation proceeds by hydrogen atom abstraction from the methyl group until 1000 K, and after this temperature, the OH center takes also part in removing atmospheric methanol. From energetics and kinetics points of view, it can be concluded that in the methanol plus NH reaction, the hydrogen abstraction reaction of the methyl group occurs more easily than that of the OH group.

**Table 3:** Branching ratios of all channels in the methanol plus NH reaction.

T(K)	k_1_(A)/S(A)	k_1_(B)/S(B)	k_2_(A)/S(A)	k_2_(B)/S(B)
300	5.64E−01	3.08E−02	9.94E + 01	9.99E + 01
400	4.78E + 00	4.95E-01	9.52E + 01	9.94E + 01
500	1.17E + 01	1.67E + 00	8.83E + 01	9.82E + 01
600	1.86E + 01	3.27E + 00	8.14E + 01	9.66E + 01
700	2.45E + 01	5.00E + 00	7.55E + 01	9.49E + 01
800	2.93E + 01	6.68E + 00	7.07E + 01	9.32E + 01
900	3.32E + 01	8.25E + 00	6.68E + 01	9.16E + 01
1000	3.63E + 01	9.67E + 00	6.37E + 01	9.02E + 01
1100	3.89E + 01	1.10E + 01	6.11E + 01	8.89E + 01
1200	4.11E + 01	1.21E + 01	5.89E + 01	8.78E + 01
1300	4.30E + 01	1.32E + 01	5.70E + 01	8.67E + 01
1400	4.46E + 01	1.41E + 01	5.54E + 01	8.58E + 01
1500	4.60E + 01	1.50E + 01	5.40E + 01	8.49E + 01
1600	4.72E + 01	1.58E + 01	5.28E + 01	8.41E + 01
1700	4.83E + 01	1.65E + 01	5.17E + 01	8.34E + 01
1800	4.92E + 01	1.72E + 01	5.08E + 01	8.27E + 01
1900	5.01E + 01	1.78E + 01	4.99E + 01	8.21E + 01
2000	5.09E + 01	1.84E + 01	4.91E + 01	8.16E + 01
2100	5.16E + 01	1.89E + 01	4.84E + 01	8.10E + 01
2200	5.23E + 01	1.94E + 01	4.77E + 01	8.05E + 01
2300	5.29E + 01	1.99E + 01	4.71E + 01	8.01E + 01
2400	5.34E + 01	2.03E + 01	4.66E + 01	7.96E + 01
2500	5.39E + 01	2.07E + 01	4.61E + 01	7.92E + 01
2600	5.44E + 01	2.11E + 01	4.56E + 01	7.89E + 01
2700	5.48E + 01	2.14E + 01	4.52E + 01	7.85E + 01
2800	5.52E + 01	2.18E + 01	4.48E + 01	7.82E + 01
2900	5.56E + 01	2.21E + 01	4.44E + 01	7.79E + 01
3000	5.59E + 01	2.24E + 01	4.41E + 01	7.76E + 01

A and B refer to the M06-2X and CBS-QB3 methods, respectively. Also, S = $\sum_{i=1}^{N}ki$.

**Figure 5:**
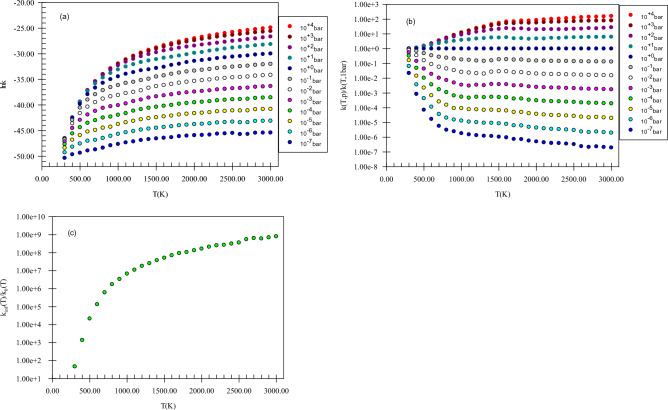
Pressure-dependent rate constants (**a**), reduced rate constants (**b**), and the ratio of *k*_*∞*_*/k*_*0*_ (**c**) for H abstraction from the methyl group of methanol by NH in the triplet state computed at the CBS-QB3 method.

Figure 5 shows the pressure-dependent rate constants, reduced rate constants, and the ratio *k*_*∞*_*/k*_*0*_ for H abstraction from the methyl group of methanol by NH. Our computed k(T,p) has positive dependence on pressure. This claim can be proved by the ratios *k*_*∞*_*/k*_*0*_ and *k*(*T,p*)/*k*(*T,1 bar*) (see Fig. 5b,c) because as mentioned in the rate constant calculation section, the ratios *k*_*∞*_*/k*_*0*_ and *k*(*T,p*)/*k*(*T,1 bar*) play a key role to argue the behavior of rate constants in the falloff regime. It is worth mentioning that we study the pressure effect only on the main reaction pathway of the selected reactions. For H abstraction from the methyl group of methanol by NH, the ratio *k*_*∞*_*/k*_*0*_ at 600, 1500, and 3000 K, are 1.33E + 05, 5.17E + 07, and 8.07E + 08, respectively. Also, the reduced rate constant for this channel at 600 K, *k*(*600 K,p*)*/k*(*600 K,1 bar*), in *p* = 10^–2^, 10^–1^, 10, and 10^2^ bar is 8.89E−02, 3.50E−01, 1.80, and 2.18, respectively. The same values at 1500 K (3000 K) are 2.86E−02 (1.31E−01), 1.88E−01 (1.98), 5.51 (6.27), and 2.14E + 01 (2.37E + 01), respectively. These results show that pressure increase has a positive impact on the hydrogen abstraction from the methyl group of methanol. Finally, these calculations prove that H abstraction from the CH_3_ group occurs easily at moderate temperatures and pressures (see Supplementary Table S17).

### Ethanol plus NH reaction

**Figure 6:**
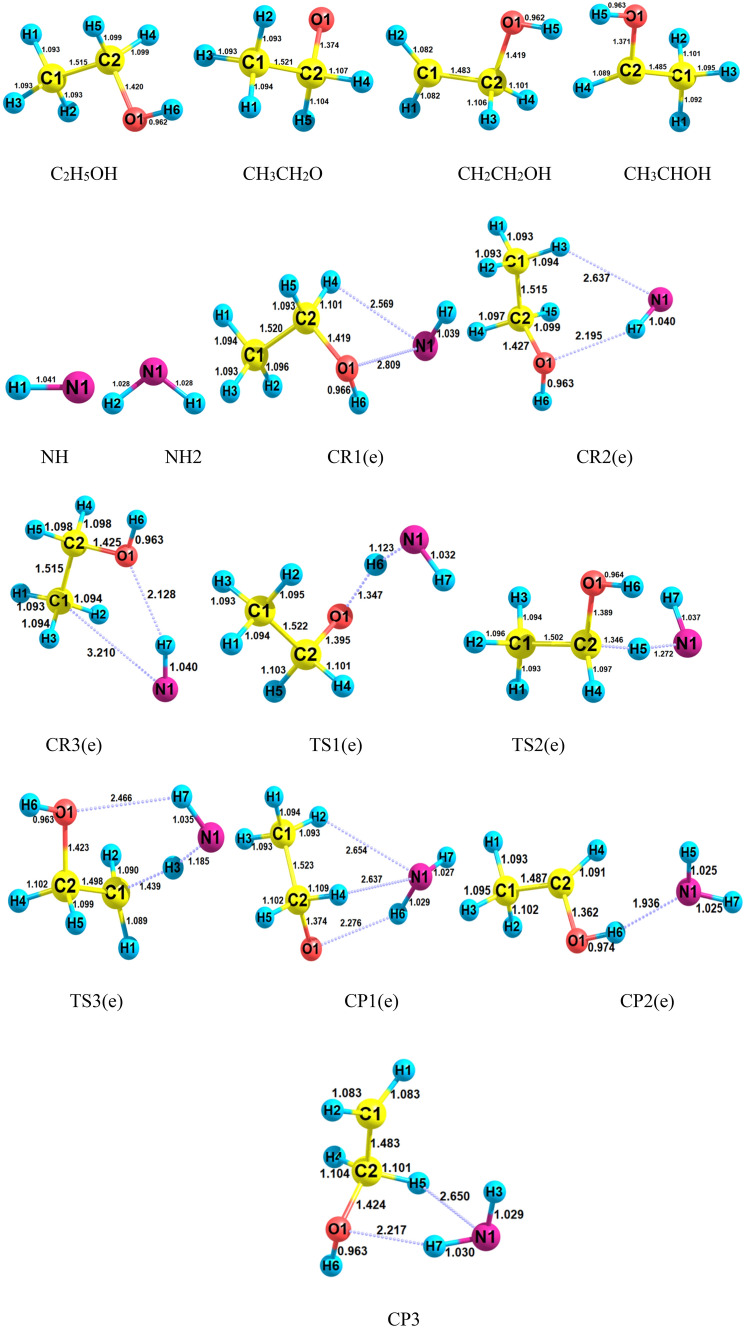
Structures of all stationary points including bond lengths (in Angstrom) in the C_2_H_5_OH + NH reaction calculated at the M06-2X method.

**Figure 7:**
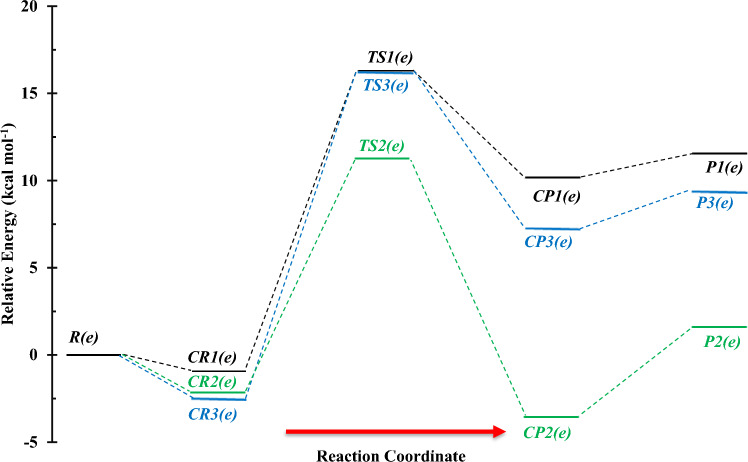
Potential energy surface of the C_2_H_5_OH + NH reaction at the triplet ground state computed by the CBS-QB3 level.

For the C_2_H_5_OH + NH reaction, the structures of all stationary points and the profile of PES are shown in Figs. 6 and 7, respectively. Also, calculated relative energies and thermodynamic parameters are listed in Tables 4 and 5. Furthermore, different orientations of NH are considered and the obtained results are in supplementary data (see Supplementary Figs. S1 and S2 and Supplementary Tables S25–S30). In the reaction between ethanol and imidogen, three individual reaction pathways are predicted based on a hydrogen atom migration from the oxygen and alpha and beta carbons (C_α_ and C_β_) centers. The paths are summarized as follows:

$$R\left( e \right) \to CR1\left( e \right) \to TS1\left( e \right) \to CP1\left( e \right) \to P1\left( e \right)(CH_{3} CH_{2} O \, + \, NH_{2} )\quad \left( {{\text{R}}_{{1}} \left( {\text{e}} \right)} \right)$$

$$R\left( e \right) \to CR2\left( e \right) \to TS2\left( e \right) \to CP2\left( e \right) \to P2\left( e \right)(CH_{3} CHOH \, + \, NH_{2} )\quad \left( {{\text{R}}_{{2}} \left( {\text{e}} \right)} \right)$$

$$R\left( e \right) \to CR3\left( e \right) \to TS3\left( e \right) \to CP3\left( e \right) \to P3\left( e \right)(CH_{2} CH_{2} OH \, + \, NH_{2} )\quad \left( {{\text{R}}_{{3}} \left( {\text{e}} \right)} \right)$$

**Table 4:** The computed relative energies for the stationary points of the C_2_H_5_OH + NH reaction (Unit of all numbers is kcal mol^−1^).

Species	∆E (0 K) (W1BD)	∆E (0 K) (CBS-QB3)	∆ (E + ZPE) (M06-2X)	MUE^1^	MUE^2^	MUE^3^
R(e)	0.00	0.00	0.00	0.00	0.00	0.00
CR1(e)	− 1.96	− 0.97	− 2.60	0.00	0.99	0.64
CR2(e)	− 2.34	− 2.22	− 3.71	0.00	0.12	1.37
CR3(e)	− 2.52	− 2.56	− 3.49	0.00	0.04	0.97
TS1(e)	17.33	16.30	14.56	0.00	1.03	2.77
TS2(e)	12.57	11.28	10.56	0.00	1.29	2.01
TS3(e)	16.98	16.17	15.10	0.00	0.81	1.88
CP1(e)	9.90	10.18	8.79	0.00	0.28	1.11
CP2(e)	− 3.84	− 3.56	− 3.16	0.00	0.28	0.68
CP3(e)	6.55	7.21	7.33	0.00	0.66	0.78
P1(e) (CH_3_CH_2_O + NH_2_)	11.38	11.55	11.75	0.00	0.17	0.37
P2(e) (CH_3_CHOH + NH_2_)	1.11	1.61	3.47	0.00	0.50	2.36
P3(e) (CH_2_CH_2_OH + NH_2_)	8.65	9.32	11.40	0.00	0.67	2.75

MUE^1^, MUE^2^, and MUE^3^ are the mean unsinged errors for the W1BD, CBS-QB3, and M06-2X methods, respectively*.*

**Table 5:** Thermodynamic parameters for stationary points of the C_2_H_5_OH + NH reaction (Unit of all numbers is kcal mol^−1^).

Species	∆E˚(A)	∆E˚(B)	∆E˚(C)	∆H˚(A)	∆H˚(B)	∆H˚(C)	∆G˚(A)	∆G˚(B)	∆G˚(C)	T∆S˚(A)	T∆S˚(B)	T∆S˚(C)
R(e)	0.00	0.00	0.00	0.00	0.00	0.00	0.00	0.00	0.00	0.00	0.00	0.00
CR1(e)	− 2.95	− 0.35	− 1.23	− 3.54	− 0.94	− 1.82	5.47	5.01	3.63	− 9.01	− 5.95	− 5.45
CR2(e)	− 3.43	− 1.79	− 1.82	− 4.03	− 2.38	− 2.41	3.34	3.73	3.43	− 7.36	− 6.11	− 5.84
CR3(e)	− 3.41	− 2.62	− 2.51	− 4.00	− 3.21	− 3.10	3.97	4.55	4.45	− 7.97	− 7.76	− 7.55
TS1(e)	14.28	16.14	17.20	13.69	15.55	16.61	22.44	23.70	24.59	− 8.75	− 8.15	− 7.98
TS2(e)	10.39	11.25	12.56	9.80	10.66	11.97	18.30	18.71	19.90	− 8.50	− 8.05	− 7.93
TS3(e)	14.77	15.92	16.81	14.18	15.33	16.22	23.20	24.14	24.76	− 9.02	− 8.81	− 8.54
CP1(e)	9.37	10.94	10.78	8.78	10.35	10.19	15.60	16.15	15.45	− 6.82	− 5.80	− 5.26
CP2(e)	− 2.75	− 2.65	− 2.89	− 3.34	− 3.24	− 3.48	3.60	2.29	1.86	− 6.94	− 5.52	− 5.34
CP3(e)	8.24	8.32	7.81	7.64	7.73	7.22	13.93	12.88	11.49	− 6.29	− 5.15	− 4.27
P1(e) (CH_3_CH_2_O + NH_2_)	11.98	11.97	11.78	11.98	11.97	11.78	10.77	10.24	10.10	1.21	1.74	1.68
P2(e) (CH_3_CHOH + NH_2_)	3.83	1.97	1.47	3.83	1.97	1.47	2.41	0.55	0.07	1.42	1.42	1.40
P3(e) (CH_2_CH_2_OH + NH_2_)	11.88	9.86	9.22	11.89	9.86	9.22	10.28	8.07	7.30	1.60	1.79	1.92

A, B, and C refer to the M06-2X, CBS-QB3, and W1BD methods, respectively.

Each path starts with a pre-reactive collision complex. The AIM topological analysis uncovers that unlike the prereactive complexes of CH_3_OH plus NH reaction, *CR1*(*e*) and *CR2*(*e*) have ring critical points. *CR1*(*e*) has a four-membered ring structure with two van der Waals interactions. The 1N…1O interaction with the charge density of $\rho_{(LCP)} =$ 0.0135 e bohr^−3^ and $\nabla^{2} \rho_{(LCP)} =$ 0.0540 e bohr^−5^ is stranger than H4…N1 interaction ($\rho_{(LCP)} =$ 0.0090 e bohr^−3^ and $\nabla^{2} \rho_{(LCP)} =$ 0.0344 e bohr^−5^). Also, the AIM of *CR2*(*e*) shows a ring structure with six members ($\rho_{(RCP)} =$ 0.0059 e bohr^−3^ and $\nabla^{2} \rho_{(RCP)} =$ 0.0256 e bohr^−5^). This complex has a hydrogen bond between the atoms 7H and 1O ($\rho_{(LCP)} =$ 0.0176 e bohr^−3^ and $\nabla^{2} \rho_{(LCP)} =$ 0.0532 e bohr^−5^). The *CR3*(*e*) ring is similar to *CR2*(*e*) but with five members. The hydrogen bond of *CR3*(*e*) is stronger than that of *CR2*(*e*) but the van der Waals interaction is weaker. The relative stability of *CR1*(*e*), *CR2*(*e*), and *CR3*(*e*) are − 0.940, − 2.38, and − 3.21 kcal mol^−1^, respectively. The equilibrium expressions of the mentioned complexes are as follows:

9 $$K_{1A} = 6.31 \times 10^{ - 27} \left( \frac{T}{300} \right){}^{1.39 \pm 0.01}\exp \left[ {\frac{{(3.87 \pm 0.01){\text{kcal}}\,{\text{mol}}^{ - 1} }}{RT}} \right],$$

10 $$K_{1B} = 4.24 \times 10^{ - 25} \left( \frac{T}{300} \right){}^{2.44 \pm 0.00}\exp \left[ {\frac{{(1.84 \pm 0.01){\text{kcal}}\,{\text{mol}}^{ - 1} }}{RT}} \right],$$

11 $$K_{2A} = 3.94 \times 10^{ - 26} \left( \frac{T}{300} \right){}^{2.43 \pm 0.01}\exp \left[ {\frac{{(4.94 \pm 0.01){\text{kcal}}\,{\text{mol}}^{ - 1} }}{RT}} \right],$$

12 $$K_{2B} = 3.16 \times 10^{ - 25} \left( \frac{T}{300} \right){}^{2.42 \pm 0.01}\exp \left[ {\frac{{(3.30 \pm 0.01){\text{kcal}}\,{\text{mol}}^{ - 1} }}{RT}} \right],$$

13 $$K_{3A} = 1.38 \times 10^{ - 26} \left( \frac{T}{300} \right){}^{2.38 \pm 0.01}\exp \left[ {\frac{{(4.93 \pm 0.02){\text{kcal}}\,{\text{mol}}^{ - 1} }}{RT}} \right],$$

14 $$K_{3B} = 5.26 \times 10^{ - 26} \left( \frac{T}{300} \right){}^{1.43 \pm 0.00}\exp \left[ {\frac{{(3.52 \pm 0.01){\text{kcal}}\,{\text{mol}}^{ - 1} }}{RT}} \right].$$

**Figure 8:**
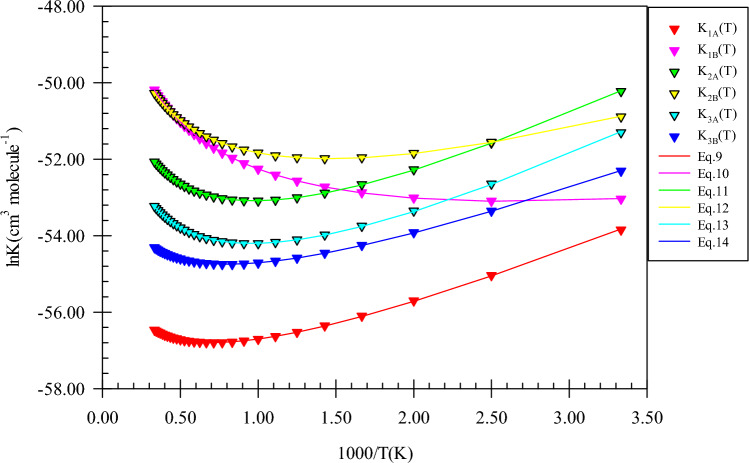
Equilibrium constants and associated fitted expressions of all prereactive complexes in the ethanol plus imidogen reaction calculated at the M06-2X (**A**) and CBS-QB3 (**B**) methods.

The graph of the computed equilibrium constants along with their fitted expressions is shown in Fig. 8. The equilibrium constants of *CR1*(*e*) and *CR2*(*e*) complexes have similar behavior compared to the equilibrium constants of *CR1*(*m*) and *CR2*(*m*) in the used computational methods. Also, the *CR3*(*e*) equilibrium constant is similar to *CR2*(*m*). The computed Gibbs free energy for *CR1*(*e*) is 5.01 kcal mol^−1^ which is 0.05 kcal mol^−1^ lower than that of *CR1*(*m*). So, *CR1*(*e*) and *CR1*(*m*) have close equilibrium constants in the 300–3000 K temperature range. Also, the free energies of *CR2*(*e*) and *CR2*(*m*) have a 0.10 kcal mol^−1^ difference. Thus, they have near values for equilibrium constants. In addition, this statement is correct about the comparison of *CR2*(*e*) and *CR3*(*e*) equilibrium constants (see Supplementary Table S10).

The complex of the first path, *CR1*(*e*), after supplying the necessary barrier energy through the saddle point *TS1*(*e*) transforms to the corresponding post-reactive complex. The IRC calculation confirms the H atom transfer from the oxygen atom of the hydroxyl group to the nitrogen atom of the imidogen and also the existence of the minima structures of *CR1*(*e*) and *CP1*(*e*) before and after *TS1*(*e*), respectively. The obtained barrier height for the first path is 17.27 kcal mol^−1^ which is 0.34 kcal mol^−1^ lower than the barrier energy of *TS1*(*m*). Moreover, the results depicted in Figs. 1 and 6 show that the N…H and O…H bond lengths in *TS1*(*m*) and *TS1*(*e*) have negligible differences. These bonds are 0.003 and 0.002 Å longer in *TS1*(*e*), respectively. The second reaction, R_2_(e), involves H abstraction from the C_α_ center. This reaction has a 3.77 kcal mol^−1^ lower energy barrier than R_1_(e). The difference in barrier energies of *TS1*(*e*) and *TS2*(*e*) may relate to the hyperconjugation phenomenon. So, the radical center on the alpha carbon can get stability by this phenomenon. Also, another reason is related to the difference in the N…H bond length in both TSs. Since the hyperconjugation phenomenon cannot take place for *TS3*(*e*), its relative energy is close to *TS1*(*e*). It should be noted that the barrier energy of *TS2*(*e*) is 5.23 kcal mol^−1^ lower than *TS3*(*e*). The difference is related to the abovementioned reasons and also the discrepancy of the relative energies of respective reactive complexes that *CR3*(*e*) is 0.34 kcal mol^−1^ more stable than *CR2*(*e*). In the structure of the *TS2*(*e*), the 5H-2C covalent bond is broken up with a length of 1.346 Å, and the N-5H covalent bond is created with a length of 1.272 Å. The *CP2*(*e*) complex is the final step of this process that converts to the final product *P2*(*e*) without passing any transition state.

The AIM results indicate that the post-reactive complex *CP1*(*e*) optimized structure involves a van der Waals interaction between the atoms 1N and 2H ($\rho_{(LCP)} =$ 0.0077 e bohr^−3^ and $\nabla^{2} \rho_{(LCP)} =$ 0.0252 e bohr^−5^), a van der Waals interaction between the atoms 4H and 1N ($\rho_{(LCP)} =$ 0.0089 e bohr^−3^ and $\nabla^{2} \rho_{(LCP)} =$ 0.0323), and a hydrogen bond interaction between the atoms 6H and 1O ($\rho_{(LCP)} =$ 0.0135 e bohr^−3^ and $\nabla^{2} \rho_{(LCP)} =$ 0.0487 e bohr^−5^), leading to form two five-membered ring structures among 1O, 6H, 1N, 4H, and 2C atoms ($\rho_{(RCP1)} =$ 0.0084 e bohr^−3^ and $\nabla^{2} \rho_{(RCP1)} =$ 0.0355 e bohr^−5^) and 1N, 2H, 1C, 2C, and 4H atoms ($\rho_{(RCP2)} =$ 0.0067 e bohr^−3^ and $\nabla^{2} \rho_{(RCP2)} =$ 0.0260 e bohr^−5^). Also, the *CP2*(*e*) has just a van der Waals interaction between the atoms 1N and 6H ($\rho_{(LCP)} =$ 0.0285 e bohr^−3^ and $\nabla^{2} \rho_{(LCP)} =$ 0.0819 e bohr^−5^). Also, For *CP3*(*e*), this analysis exhibits a five-membered ring structure with a van der Waals inaction between the atoms 1N and 5H ($\rho_{(RCP)} =$ 0.0081 e bohr^−3^ and $\nabla^{2} \rho_{(RCP)} =$ 0.0324 e bohr^−5^) and a hydrogen bond between the atoms 7H and 1O ($\rho_{(LCP)} =$ 0.0165 e bohr^−3^ and $\rho_{(LCP)} =$ 0.0517 e bohr^−5^).

For the above-discussed paths, the following rate expressions are extracted from the calculated values by the TST/Eckart theory at the M06-2X (A) and CBS-QB3 (B) methods.

15 $$k_{1A} = 9.36 \times 10^{ - 15} \left( \frac{T}{300} \right){}^{3.31 \pm 0.03}\exp \left[ { - \frac{{(11.31 \pm 0.05){\text{kcal}}\,{\text{mol}}^{ - 1} }}{RT}} \right],$$

16 $$k_{1B} = 2.81 \times 10^{ - 14} \left( \frac{T}{300} \right){}^{3.29 \pm 0.03}\exp \left[ { - \frac{{(13.35 \pm 0.04){\text{kcal}}\,{\text{mol}}^{ - 1} }}{RT}} \right],$$

17 $$k_{2A} = 2.75 \times 10^{ - 15} \left( \frac{T}{300} \right){}^{4.08 \pm 0.12}\exp \left[ { - \frac{{(5.32 \pm 0.21){\text{kcal}}\,{\text{mol}}^{ - 1} }}{RT}} \right],$$

18 $$k_{2B} = 8.95 \times 10^{ - 15} \left( \frac{T}{300} \right){}^{3.95 \pm 0.11}\exp \left[ { - \frac{{(6.73 \pm 0.19){\text{kcal}}\,{\text{mol}}^{ - 1} }}{RT}} \right],$$

19 $$k_{3A} = 3.22 \times 10^{ - 15} \left( \frac{T}{300} \right){}^{3.73 \pm 0.07}\exp \left[ { - \frac{{(11.01 \pm 0.13){\text{kcal}}\,{\text{mol}}^{ - 1} }}{RT}} \right],$$

20 $$k_{3B} = 4.10 \times 10^{ - 15} \left( \frac{T}{300} \right){}^{3.76 \pm 0.08}\exp \left[ { - \frac{{(12.08 \pm 0.14){\text{kcal}}\,{\text{mol}}^{ - 1} }}{RT}} \right].$$

**Figure 9:**
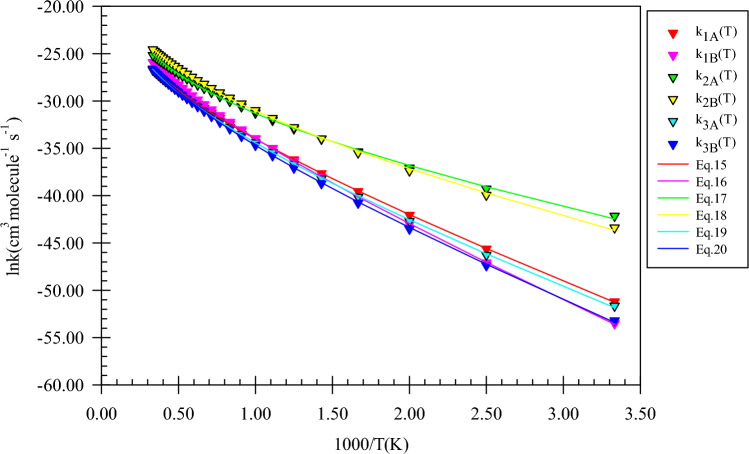
Graph of the high-pressure limit rate constants (cm^3^ molecule^−1^ s^−1^) and fitted non-Arrhenius expressions for the gas-phase formation of the *P1*(*e*), *P2*(*e*), and *P3*(*e*) products calculated by the TST/Eckart theory at the M06-2X (**A**) and CBS-QB3 (**B**) methods.

In Fig. 9, the graph of rate constants and associated fitted expressions are sketched. The results of rate constants for H abstraction from the OH and methyl groups of ethanol show that transferring a hydrogen atom from these groups to imidogen has near rate constants at the M06-2X method. So, roughly similar expressions $9.36 \times 10^{{ - 15}} \left( {\frac{T}{{300}}} \right){}^{{3.31}}\exp \left( { - \frac{{11.31\,{\text{kcal}}\,{\text{mol}}^{{ - 1}} }}{{RT}}} \right)$ and $3.22 \times 10^{ - 15} \left( \frac{T}{300} \right){}^{3.73}\exp \left( { - \frac{{11.01\,{\text{kcal}}\,{\text{mol}}^{ - 1} }}{RT}} \right)$ cm^3^ molecule^−1^ s^−1^ are extracted, respectively, for OH and CH_3_ groups. But, there are small differences in the calculated rate constants by the CBS-QB3 method; i.e., the rate of the R_1_(e) path is 1.86, 2.10, and 2.00 times more than R_3_(e) at 600 K, 1500, 3000 K, respectively. These differences explicitly are seen from the rate expressions of $2.81 \times 10^{ - 14} \left( \frac{T}{300} \right){}^{3.29}\exp \left( { - \frac{{13.35\,{\text{kcal}}\,{\text{mol}}^{ - 1} }}{RT}} \right)$ cm^3^ molecule^−1^ s^−1^ (for CH_3_CH_2_O + NH_2_ formation) and $4.10 \times 10^{ - 15} \left( \frac{T}{300} \right){}^{3.76}\exp \left( { - \frac{{12.08\,{\text{kcal}}\,{\text{mol}}^{ - 1} }}{RT}} \right)$ cm^3^ molecule^−1^ s^−1^ (for CH_2_CH_2_OH + NH_2_ formation).

Also, as expected quantitatively, the ratio of the rate of R_1_(e) to R_1_(m) channels is just 1.41 (600 K), 1.27 (1500 K), and 1.23 (3000 K) at the CBS-QB3 method. Thus, in comparison with the CH_3_OH + NH reaction, there is no sensible difference in the rate of H atom transfer from the OH group of ethanol to imidogen.

The rate expressions $2.75 \times 10^{{ - 15}} \left( {\frac{T}{{300}}} \right){}^{{4.08}}\exp \left( { - \frac{{5.32\,{\text{kcal}}\,{\text{mol}}^{{ - 1}} }}{{RT}}} \right)$ cm^3^ molecule^−1^ s^−1^ at the M06-2X method and $8.95 \times 10^{ - 15} \left( \frac{T}{300} \right){}^{3.95}\exp \left( { - \frac{{6.73\,{\text{kcal}}\,{\text{mol}}^{ - 1} }}{RT}} \right)$ cm^3^ molecule^−1^ s^−1^ at the CBS-QB3 method are for the H abstraction reaction from the middle center of ethanol which is the main reaction channel. It should be noted that the rate of the R_2_(e) channel at 600, 1500, and 3000 K is 108.86, 8.64, and 3.84 times higher than R_1_(e) and is 204.25, 17.37, and 7.68 times higher than R_3_(e) at the CBS-QB3 method, respectively. However, the R_2_(e) channel has a 5.53, 1.94, and 1.36 times higher rate than the similar channel in methanol plus NH reaction, R_2_(m), at 600, 1500, and 3000 K temperatures, respectively.

Branching ratios of all products in the ethanol and NH reaction are collected in Table 6. According to the data in Table 6, the branching ratio of product *P2 *(*e*) in the temperature range of 300–3000 K prevails over the others. The calculated production percentage at the M06-2X method for *P1*(*e*) formation up to 1500 K is below 10% and at 3000 K is 16%. Also, a roughly similar trend is seen for the CBS-QB3 results. Instead, the branching ratio of *P3*(*e*) in both methods is different. In the M06-2X method, it has 14.7% percent of formation but in the CBS-QB3 method, the *P3*(*e*) percentage is below 10% at 3000 K.

**Table 6:** Branching ratios of all channels in the ethanol plus NH reactions.

T(K)	k_1_(A)/S(A)	k_1_(B)/S(B)	k_2_(A)/S(A)	k_2_(B)/S(B)	k_3_(A)/S(A)	k_3_(B)/S(B)
300	1.16E−02	3.94E−03	1.00E + 02	1.00E + 02	7.44E−03	5.54E−03
400	1.74E−01	7.72E−02	9.97E + 01	9.99E + 01	9.11E−02	5.74E−02
500	6.67E−01	3.54E−01	9.90E + 01	9.94E + 01	3.51E−01	2.10E−01
600	1.48E + 00	8.98E−01	9.77E + 01	9.86E + 01	8.15E−01	4.82E−01
700	2.49E + 00	1.68E + 00	9.61E + 01	9.75E + 01	1.45E + 00	8.62E−01
800	3.59E + 00	2.63E + 00	9.42E + 01	9.61E + 01	2.21E + 00	1.32E + 00
900	4.70E + 00	3.67E + 00	9.23E + 01	9.45E + 01	3.04E + 00	1.83E + 00
1000	5.77E + 00	4.76E + 00	9.03E + 01	9.29E + 01	3.90E + 00	2.36E + 00
1100	6.78E + 00	5.85E + 00	8.85E + 01	9.12E + 01	4.76E + 00	2.90E + 00
1200	7.71E + 00	6.92E + 00	8.67E + 01	8.96E + 01	5.59E + 00	3.43E + 00
1300	8.57E + 00	7.95E + 00	8.50E + 01	8.81E + 01	6.40E + 00	3.94E + 00
1400	9.36E + 00	8.94E + 00	8.35E + 01	8.66E + 01	7.16E + 00	4.44E + 00
1500	1.01E + 01	9.87E + 00	8.20E + 01	8.52E + 01	7.88E + 00	4.90E + 00
1600	1.08E + 01	1.07E + 01	8.07E + 01	8.39E + 01	8.57E + 00	5.35E + 00
1700	1.14E + 01	1.16E + 01	7.94E + 01	8.27E + 01	9.20E + 00	5.76E + 00
1800	1.19E + 01	1.23E + 01	7.83E + 01	8.15E + 01	9.80E + 00	6.16E + 00
1900	1.24E + 01	1.31E + 01	7.72E + 01	8.04E + 01	1.04E + 01	6.52E + 00
2000	1.29E + 01	1.38E + 01	7.62E + 01	7.94E + 01	1.09E + 01	6.87E + 00
2100	1.34E + 01	1.44E + 01	7.53E + 01	7.84E + 01	1.14E + 01	7.19E + 00
2200	1.38E + 01	1.50E + 01	7.44E + 01	7.75E + 01	1.18E + 01	7.50E + 00
2300	1.41E + 01	1.56E + 01	7.36E + 01	7.66E + 01	1.23E + 01	7.78E + 00
2400	1.45E + 01	1.61E + 01	7.28E + 01	7.58E + 01	1.27E + 01	8.05E + 00
2500	1.48E + 01	1.66E + 01	7.21E + 01	7.51E + 01	1.31E + 01	8.30E + 00
2600	1.51E + 01	1.71E + 01	7.14E + 01	7.44E + 01	1.34E + 01	8.54E + 00
2700	1.54E + 01	1.75E + 01	7.08E + 01	7.37E + 01	1.38E + 01	8.76E + 00
2800	1.57E + 01	1.80E + 01	7.02E + 01	7.31E + 01	1.41E + 01	8.97E + 00
2900	1.59E + 01	1.84E + 01	6.97E + 01	7.25E + 01	1.44E + 01	9.17E + 00
3000	1.62E + 01	1.87E + 01	6.92E + 01	7.19E + 01	1.47E + 01	9.36E + 00

A and B refer to the M06-2X and CBS-QB3 methods, respectively. Also, S = $\sum_{i=1}^{N}ki$.

**Figure 10:**
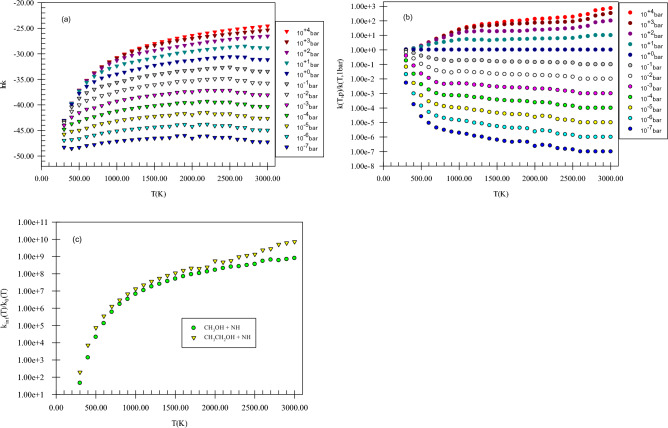
Pressure-dependent rate constants (**a**), reduced rate constants (**b**), and the ratio *k*_*∞*_*/k*_*0*_ (**c**) for H abstraction from the C_α_ center of ethanol by NH in the triplet state computed at the CBS-QB3 method.

Our compared pressure-dependent rate constants, reduced rate constants, and the ratio *k*_*∞*_*/k*_*0*_ for H abstraction from the C_α_ center of ethanol by NH are shown in Fig. 10. In Fig. 10a, the positive dependence of k(T,p) to pressure increase is seen. As mentioned above, this can illustrate more clearly by the ratio *k*_*∞*_*/k*_*0*_ (see Fig. 10c). This ratio for the H abstraction from the Cα center of ethanol by NH, at 600, 1500, and 3000 K is 3.49E + 05, 1.11E + 08, and 7.24E + 09, respectively. Also, the reduced rate constants for this channel demonstrate directly this statement (see Fig. 10b). The reduced rate constant for this reaction at 600 K, *k*(*600 K,p*)*/k*(*600 K,1 bar*), when *p* is 10^–2^, 10^–1^, 10, and 10^2^ bar is 6.28E−02, 2.90E−01, 2.21, and 3.01, respectively. The values of this ratio when *p* gets the same values at 1500 K are 2.20E−02, 1.58E−01, 4.87, and 1.84E + 01, and at 3000 K are 1.00E−02, 1.00E−01, 1.59, and 100, respectively. To have insight into the difference of *k*(*T,p*) between methanol and ethanol reactions, we define two ratios as (*k*_*∞*_*/k*_*0*_)_*e*_*/*(*k*_*∞*_*/k*_*0*_)_*m*_ and *k*(*T,p*)_*e*_*/*(*k*(*T,p*)_*m*_. The prior ratio at 600, 1500, and 3000 K is 2.62, 2.14, and 8.98, respectively. And the last ratio in the pressures of 10^–2^, 10^–1^, 10, 1, and 10^2^ bar and temperature of 600 K is 3.35, 3.39, 4.74, 5.81, and 6.55, respectively. These values change to 1.32, 1.44, 1.71, 1.51, and 1.47, at 1500 K and 2.02E−01, 2.37E−01, 3.09E−01, 4.93E−01, and 1.13 at 3000 K, respectively. Through the results argued in this paragraph, we understand that the rate constant of ethanol at high pressures and low temperatures is higher than methanol but at high temperatures is lower. These results show that hydrogen abstraction from the C_α_ center of ethanol occurs easly than the methyl group of methanol when pressure increases at moderate temperatures but at high temperatures, an inverse behavior is expected.

### N-propanol plus NH reaction

**Figure 11:**
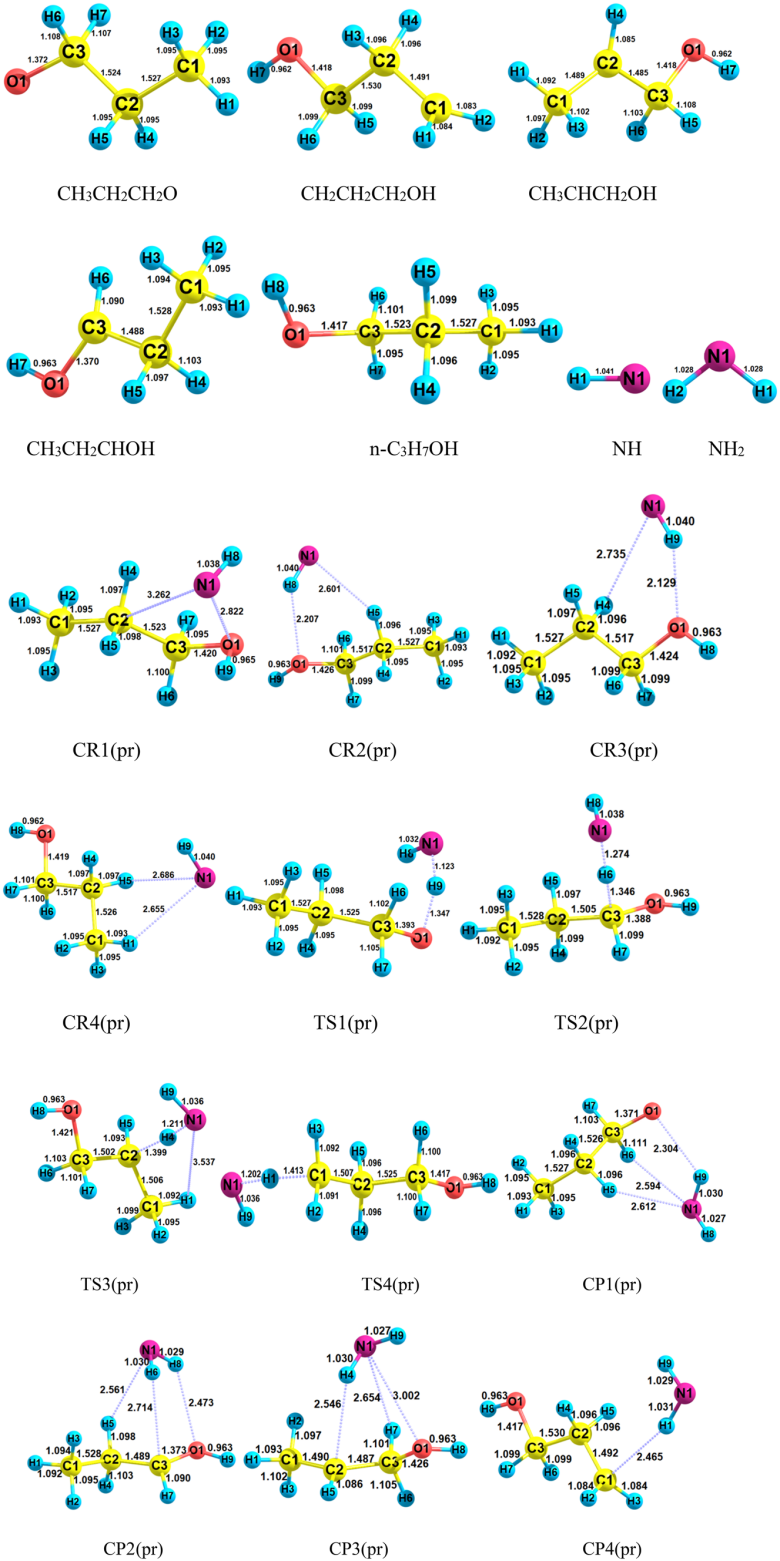
Structures of all stationary points including bond lengths (in Angstrom) in the *n*-C_3_H_7_OH + NH reaction calculated at the M06-2X method.

**Figure 12:**
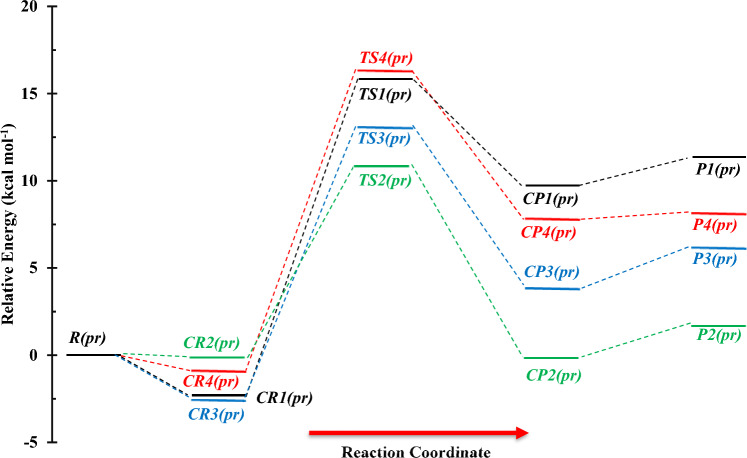
Potential energy surface of the *n*-C_3_H_7_OH + NH reaction at the triplet ground state computed by the CBS-QB3 level.

The structures including geometrical parameters and PES for n-propanol plus NH reaction are represented in Figs. 11 and 12, respectively. Also, the computed relative energies and thermodynamic parameters are collected in Table 7. Moreover, other conformers and their reactions are brought out in the supplementary data (see Supplementary Figs. S3–S8, and Supplementary Tables S31–S42). The most likelihood atmospheric paths of the *n*-C_3_H_7_OH + NH (*R*(*pr*)) reaction are found and the main annihilation pathways are presented as follows:

$$R\left( {pr} \right) \to CR1\left( {pr} \right) \to TS1\left( {pr} \right) \to CP1\left( {pr} \right) \to P1\left( {pr} \right) \, \left( {CH_{3} CH_{2} CH_{2} O \, + \, NH_{2} } \right) \quad \left( {{\text{R}}_{{1}} (pr)} \right)$$

$$R\left( {pr} \right) \to CR2\left( {pr} \right) \to TS2\left( {pr} \right) \to CP2\left( {pr} \right) \to P2\left( {pr} \right) \, \left( {CH_{3} CH_{2} CHOH \, + \, NH_{2} } \right)\quad \left( {{\text{R}}_{{2}} (pr)} \right)$$

$$R\left( {pr} \right) \to CR3\left( {pr} \right) \to TS3\left( {pr} \right) \to CP3\left( {pr} \right) \to P3\left( {pr} \right) \, \left( {CH_{3} CHCH_{2} OH \, + \, NH_{2} } \right)\quad \left( {{\text{R}}_{{3}} (pr)} \right)$$

$$R\left( {pr} \right) \to CR4\left( {pr} \right) \to TS4\left( {pr} \right) \to CP4\left( {pr} \right) \to P4\left( {pr} \right) \, \left( {CH_{2} CH_{2} CH_{2} OH \, + \, NH_{2} } \right)\quad \left( {{\text{R}}_{{4}} (pr)} \right)$$

**Table 7:** Relative energies and thermodynamic parameters for stationary points of the *n*-C_3_H_7_OH + NH reaction (Unit of all numbers is kcal mol^−1^).

Species	∆(E + ZPE)(A)	∆E(0 K)(B)	∆E˚(A)	∆E˚(B)	∆H˚(A)	∆H˚(B)	∆G˚(A)	∆G˚(B)	T∆S˚(A)	T∆S˚(B)
R(pr)	0.00	0.00	0.00	0.00	0.00	0.00	0.00	0.00	0.00	0.00
CR1(pr)	− 2.90	− 2.43	− 2.65	− 3.15	− 3.24	− 1.97	4.62	3.56	− 7.86	− 5.53
CR2(pr)	− 3.56	− 0.20	− 3.39	− 0.27	− 3.98	0.91	3.85	5.33	− 7.83	− 4.42
CR3(pr)	− 3.81	− 2.48	− 3.53	− 3.17	− 4.13	− 1.99	3.52	3.40	− 7.65	− 5.38
CR4(pr)	− 1.28	− 0.93	− 0.67	− 1.01	− 1.26	0.18	5.21	2.84	− 6.47	− 2.66
TS1(pr)	14.09	15.83	13.89	14.59	13.30	15.77	21.86	23.07	− 8.56	− 7.30
TS2(pr)	10.25	10.76	10.01	9.61	9.42	10.80	18.39	18.22	− 8.97	− 7.42
TS3(pr)	12.03	13.02	12.08	11.79	11.49	12.97	19.65	20.99	− 8.16	− 8.02
TS4(pr)	16.25	16.29	16.14	15.20	15.55	16.39	24.00	49.60	− 8.45	− 33.21
CP1(pr)	8.18	9.72	8.42	9.38	7.82	10.57	15.44	15.63	− 7.62	− 5.06
CP2(pr)	0.73	− 0.20	1.52	− 0.27	0.93	0.91	7.50	5.33	− 6.58	− 4.42
CP3(pr)	4.29	3.79	5.33	3.88	4.73	5.06	10.47	8.87	− 5.74	− 3.81
CP4(pr)	8.83	7.78	9.51	8.01	8.92	9.20	15.28	12.58	− 6.35	− 3.39
P1(pr) (CH_3_CH_2_CH_2_O + NH_2_)	11.71	11.37	11.89	10.55	10.73	12.33	8.61	10.17	2.12	2.16
P2(pr) (CH_3_CH_2_CHOH + NH_2_)	3.93	1.69	4.21	0.86	4.21	2.64	2.94	0.54	1.28	2.10
P3(pr) (CH_3_CHCH_2_OH + NH_2_)	8.23	6.14	8.87	5.63	8.88	7.41	6.51	4.36	2.36	3.05
P4(pr) (CH_2_CH_2_CH_2_OH + NH_2_)	10.11	8.09	10.73	7.03	10.73	8.81	8.61	7.22	2.12	1.59

A and B refer to the M06-2X and CBS-QB3 methods, respectively.

The same as the above-discussed paths for methanol and ethanol reactions with NH, these pathways all include conventional hydrogen atom transfer reactions.

Ring structures for prereactive complexes of n-propanol plus NH reaction are analysed by AIM theory. The same as *CR1*(*e*) and *CR2*(*e*), *CR1*(*pr*) and *CR2*(*pr*) have similar four and six-membered ring structures, respectively. The structure of *CR1*(*pr*) includes two van der Waals interactions. One of them is the 1N…1O bond ($\rho_{(LCP)} =$ 0.0121 e bohr^−3^ and $\nabla^{2} \rho =$ 0.0458 e bohr^−5^) and the other is the C2…N1 bond ($\rho_{(LCP)} =$ 0.0062 e bohr^−3^ and $\nabla^{2} \rho =$ 0.0208 e bohr^−5^). Also, for *CR2*(*pr*), the 8H…1O (hydrogen) bond ($\rho_{(LCP)} =$ 0.0172 e bohr^−3^ and $\nabla^{2} \rho =$ 0.0521 e bohr^−5^) along with the N1…H5 van der Waals interaction ($\rho_{(LCP)} =$ 0.0077 e bohr^−3^ and $\nabla^{2} \rho =$ 0.0271 e bohr^−5^) is formed before reaction. The third complex *CR3*(*pr*) has a different ring structure compared to *CR3*(*e*). The difference goes back to the van der Waals interaction that in *CR3*(*e*) is formed between methyl carbon of ethanol and nitrogen of imidogen, but in *CR3*(*pr*), it is created between the hydrogen (4H) of β carbon and N atom of NH. Also, hydrogen bonds of the *CR3*(*pr*) complex ($\rho_{(RCP)} =$ 0.0180 e bohr^−3^ and $\nabla^{2} \rho_{(RCP)} =$ 0.0554 e bohr^−5^) are similar to *CR3*(*e*). About the final complex, *CR4*(*pr*), two van der Waals interactions are the reason of the formation of a five-membered ring structure ($\rho_{(RCP)} =$ 0.0061 e bohr^−3^ and $\nabla^{2} \rho_{(RCP)} =$ 0.0220 e bohr^−5^). Finally, it should be noted that the relative stability of *CR1*(*pr*), *CR2*(*pr*), and *CR3*(*pr*) are − 0.944, − 0.944, and − 0.944 kcal mol^−1^, respectively, which are close to the relative stability of *CR1*(*e*), *CR2*(*e*), and *CR3*(*e*). The equilibrium expressions for *CR1*(*pr*), *CR2*(*pr*), *CR3*(*pr*), and *CR4*(*pr*) complexes are as follows:

21 $$K_{1A} = 1.75 \times 10^{ - 26} \left( \frac{T}{300} \right){}^{2.38 \pm 0.01}\exp \left[ {\frac{{(4.13 \pm 0.01){\text{kcal}}\,{\text{mol}}^{ - 1} }}{RT}} \right],$$

22 $$K_{1B} = 3.09 \times 10^{ - 25} \left( \frac{T}{300} \right){}^{2.43 \pm 0.01}\exp \left[ {\frac{{(3.48 \pm 0.01){\text{kcal}}\,{\text{mol}}^{ - 1} }}{RT}} \right],$$

23 $$K_{2B} = 1.78 \times 10^{ - 26} \left( \frac{T}{300} \right){}^{2.38 \pm 0.01}\exp \left[ {\frac{{(4.90 \pm 0.01){\text{kcal}}\,{\text{mol}}^{ - 1} }}{RT}} \right],$$

24 $$K_{2B} = 3.94 \times 10^{ - 25} \left( \frac{T}{300} \right){}^{2.43 \pm 0.01}\exp \left[ {\frac{{(3.50 \pm 0.01){\text{kcal}}\,{\text{mol}}^{ - 1} }}{RT}} \right],$$

25 $$K_{3A} = 2.49 \times 10^{ - 26} \left( \frac{T}{300} \right){}^{2.39 \pm 0.01}\exp \left[ {\frac{{(5.03 \pm 0.01){\text{kcal}}\,{\text{mol}}^{ - 1} }}{RT}} \right],$$

26 $$K_{3B} = 3.97 \times 10^{ - 25} \left( \frac{T}{300} \right){}^{2.43 \pm 0.01}\exp \left[ {\frac{{(3.50 \pm 0.01){\text{kcal}}\,{\text{mol}}^{ - 1} }}{RT}} \right],$$

27 $$K_{4A} = 1.87 \times 10^{ - 25} \left( \frac{T}{300} \right){}^{2.45 \pm 0.00}\exp \left[ {\frac{{(2.15 \pm 0.01){\text{kcal}}\,{\text{mol}}^{ - 1} }}{RT}} \right],$$

28 $$K_{4B} = 4.16 \times 10^{ - 23} \left( \frac{T}{300} \right){}^{2.50 \pm 0.00}\exp \left[ {\frac{{(1.31 \pm 0.01){\text{kcal}}\,{\text{mol}}^{ - 1} }}{RT}} \right].$$

**Figure 13:**
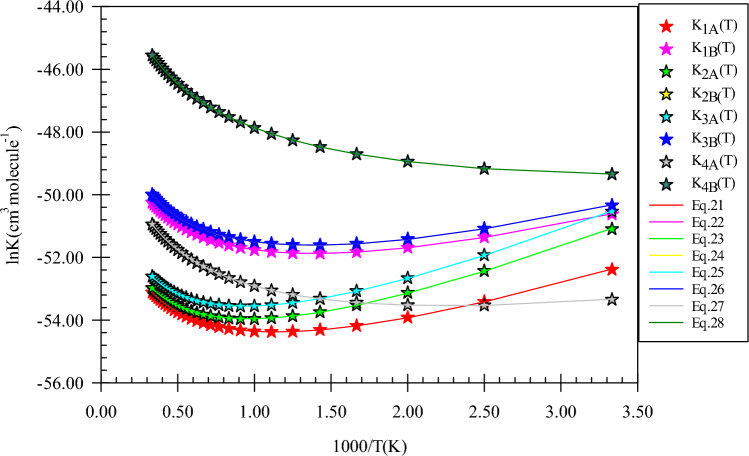
Equilibrium constants and associated fitted expressions of all prereactive complexes in the n-propanol plus imidogen reaction calculated at the M06-2X (**A**) and CBS-QB3 (**B**) methods.

The sketch of equilibrium constants and fitted expressions are brought out in Fig. 13. The free energies of *CR1*(*pr*), *CR2*(*pr*), *CR3*(*pr*), and *CR4*(*pr*) complexes are 3.56, 5.33, 3.40, and 2.84 kcal mol^−1^, respectively, at the CBS-QB3 method. Thus, the equilibrium constants of these complexes are 3.11E−23, 4.05E−23, 4.06E−23, and 7.07E−22 cm^3^ molecule^−1^ at 600 K, respectively. The values of the equilibrium constants increase to 1.51E−22, 1.94E−22, 1.95E−22, and 1.65E−20 cm^3^ molecule^−1^ at 3000 K, respectively. It is better to say that the ratios K_CR1(pr)_/K_CR1(e)_, K_CR2(pr)_/K_CR2(e)_, and K_CR3(pr)_/K_CR3(e)_ are 2.86, 1.48, and 14.76 at 600 K, 1.25, 1.36, and 37.45 at 1500 K and 0.95, 1.32, and 75.50 at 3000 K, respectively.

In our first suggested route R_1_(pr), the covalent 1O-9H bond with a length of 1.347 Å is ruptured and another covalent bond between the atoms 1N and 6H with a length of 1.123 Å is built. This reaction begins with *CR1*(*pr*) complex formation and after surmounting the barrier of 18.26 kcal mol^−1^ converts to *CP1*(*pr*). This path ends with *P1*(*pr*) product formation after the dissociation of *CP1*(*pr*) to its fragments (CH_3_CHOH and NH_2_) without any barrier. Compare to the methanol and ethanol reactions, the hydrogen abstraction from the OH group of n-propanol is relatively difficult due to the mentioned barrier energy which is 0.65 and 1.00 kcal mol^−1^ higher than *TS1*(*m*) and *TS1*(*e*), respectively.

The second, third, and fourth proposed paths are R_2_(pr), R_3_(pr), and R_4_(pr) which describe the H abstraction reaction from C_α_, C_β_, and C_γ_, respectively. For this, we have designed three paths that include the formation of the *CR2*(*pr*), *CR3*(*pr*), and *CR4*(*pr*) complexes due to the presence of the intermolecular interactions between reactants, the saddle points *TS2*(*pr*), *TS3*(*pr*), and *TS4*(*pr*) with the barrier energies of 10.96, 15.50, and 17.21 kcal mol^−1^, and the formation of the *CP2*(*pr*), *CP3*(*pr*), and *CP4*(*pr*) product complexes. Finally, the products *P2*(*pr*), *P3*(*pr*), and *P4*(*pr*) are released after the dissociation of the bonds between the NH_2_ group and the ethanol residues including CH_3_CH_2_O, CH_3_CHOH, and CH_2_CH_2_OH, respectively. The second, third, and fourth saddle points simulate the cleavage of the 3C-6H, 2C-4H, and 1C-1H covalent bonds with lengths of 1.346, 1.399, and 1.413 Å, respectively, and simultaneously the formation of the N-6H, N-4H, and N-1H covalent bonds with lengths of 1.274, 1.211, and 1202 Å, respectively.

The AIM calculations show that the *CP1*(*pr*) optimized structure has two rings that each has five members (see Fig. 11). This structure includes two van der Waals bonds between the atoms 1N and 5H ($\rho_{(RCP)} =$ 0.0083 e bohr^−3^ and $\nabla^{2} \rho_{(RCP)} =$ 0.0271 e bohr^−5^) and the atoms 1N and 6H ($\rho_{(LCP)} =$ 0.0095 e bohr^−3^ and $\nabla^{2} \rho_{(LCP)} =$ 0.0342 e bohr^−5^) and a hydrogen bond between the atoms 1O and 9H ($\rho_{(LCP)} =$ 0.0605 e bohr^−3^ and $\nabla^{2} \rho_{(LCP)} =$ 0.1582 e bohr^−5^). Also, the optimized structure of *CP2*(*pr*) includes two five-membered ring structures in which NH moiety connects to n-propanol by two van der Waals interactions between the atoms 1N and 5H ($\rho_{(LCP)} =$ 0.0085 e bohr^−3^ and $\nabla^{2} \rho_{(LCP)} =$ 0.0296 e bohr^−5^) and the atoms 6H and 3C ($\rho_{(LCP)} =$ 0.0085 e bohr^−3^ and $\nabla^{2} \rho_{(LCP)} =$ 0.0268 e bohr^−5^) and a hydrogen bond between the atoms 8H and 1O ($\rho_{(LCP)} =$ 0.0102 e bohr^−3^ and $\nabla^{2} \rho_{(LCP)} =$ 0.0356 e bohr^−5^). The attained *CP3*(*pr*) complex has two ring structures that each has four members, containing three van der Waals bonds between the atoms 2C and 4H ($\rho_{(LCP)} =$ 0.0101 e bohr^−3^ and $\nabla^{2} \rho =$ 0.0323 e bohr^−5^), the 7H and 1N atoms ($\rho_{(LCP)} =$ 0.0084 e bohr^−3^ and $\nabla^{2} \rho_{(LCP)} =$ 0.0315 e bohr^−5^), and the atoms 1N and 1O ($\rho_{(LCP)} =$ 0.0091 e bohr^−3^ and $\nabla^{2} \rho_{(LCP)} =$ 0.0340 e bohr^−5^). The *CP4*(*pr*) complex contains only a van der Waals bond between the 1C and 1H atoms ($\rho_{(LCP)} =$ 0.0113 e bohr^−3^ and $\nabla^{2} \rho_{(LCP)} =$ 0.0300 e bohr^−5^).

**Figure 14:**
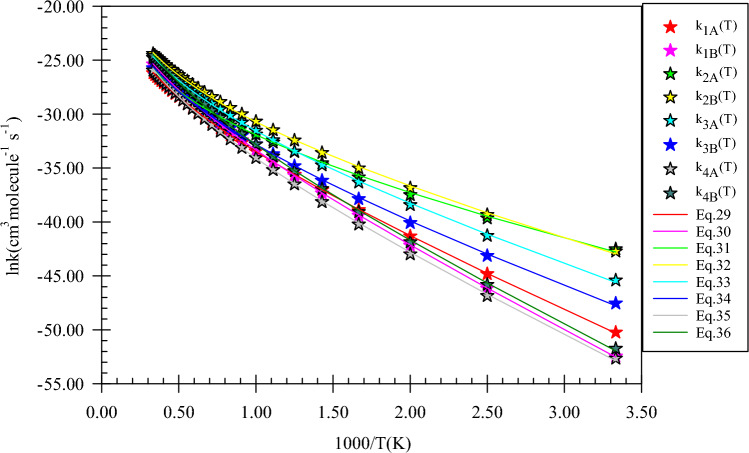
Graph of the high-pressure limit rate constants (cm^3^ molecule^−1^ s^−1^) and fitted non-Arrhenius expressions for the gas-phase formation of the *P1*(*pr*), *P2*(*pr*), *P3*(*pr*), and *P4*(*pr*) products calculated by the TST/Eckart theory at the M06-2X (**A**) and CBS-QB3 (**B**) methods.

The rate constants of all reaction channels accompanied by their fitted expressions are depicted in Fig. 14. Also, the results of branching ratios for the *n*-propanol and NH reaction are tabulated in Table 8. The kinetics of the product generation via the R_1_(pr)–R_4_(pr) reaction pathways are investigated to have a suitable criterion for the conversion of reactants to each product. The fitted rate expressions for the R_1_(pr) to R_4_(pr) paths are as follows:

29 $$k_{1A} = 1.33 \times 10^{ - 14} \left( \frac{T}{300} \right){}^{3.32 \pm 0.03}\exp \left[ { - \frac{{(10.95 \pm 0.05){\text{kcal}}\,{\text{mol}}^{ - 1} }}{RT}} \right],$$

30 $$k_{1B} = 4.28 \times 10^{ - 14} \left( \frac{T}{300} \right){}^{3.31 \pm 0.03}\exp \left[ { - \frac{{(12.96 \pm 0.05){\text{kcal}}\,{\text{mol}}^{ - 1} }}{RT}} \right],$$

31 $$k_{2A} = 1.62 \times 10^{ - 15} \left( \frac{T}{300} \right){}^{3.96 \pm 0.10}\exp \left[ { - \frac{{(5.20 \pm 0.18){\text{kcal}}\,{\text{mol}}^{ - 1} }}{RT}} \right],$$

32 $$k_{2B} = 1.13 \times 10^{ - 14} \left( \frac{T}{300} \right){}^{3.87 \pm 0.09}\exp \left[ { - \frac{{(6.48 \pm 0.16){\text{kcal}}\,{\text{mol}}^{ - 1} }}{RT}} \right],$$

33 $$k_{3A} = 1.21 \times 10^{ - 14} \left( \frac{T}{300} \right){}^{3.79 \pm 0.08}\exp \left[ { - \frac{{(8.09 \pm 0.14){\text{kcal}}\,{\text{mol}}^{ - 1} }}{RT}} \right],$$

34 $$k_{3B} = 4.60 \times 10^{ - 15} \left( \frac{T}{300} \right){}^{3.85 \pm 0.09}\exp \left[ { - \frac{{(8.80 \pm 0.15){\text{kcal}}\,{\text{mol}}^{ - 1} }}{RT}} \right],$$

35 $$k_{4A} = 6.80 \times 10^{ - 15} \left( \frac{T}{300} \right){}^{3.83 \pm 0.08}\exp \left[ { - \frac{{(12.07 \pm 0.15){\text{kcal}}\,{\text{mol}}^{ - 1} }}{RT}} \right],$$

36 $$k_{4B} = 2.85 \times 10^{ - 14} \left( \frac{T}{300} \right){}^{3.82 \pm 0.08}\exp \left[ { - \frac{{(12.38 \pm 0.15){\text{kcal}}\,{\text{mol}}^{ - 1} }}{RT}} \right].$$

**Table 8:** Branching ratios of all channels in the n-propanol plus NH reaction.

T(K)	k_1_(A)/S(A)	k_1_(B)/S(B)	k_2_(A)/S(A)	k_2_(B)/S(B)	k_3_(A)/S(A)	k_3_(B)/S(B)	k_4_(A)/S(A)	k_4_(B)/S(B)
300	4.13E−02	5.91E−03	9.47E + 01	9.92E + 01	5.24E + 00	8.01E−01	3.72E−03	1.21E−02
400	4.73E−01	1.03E−01	8.31E + 01	9.76E + 01	1.63E + 01	2.15E + 00	6.18E−02	1.48E−01
500	1.51E + 00	4.59E−01	7.05E + 01	9.52E + 01	2.77E + 01	3.74E + 00	2.87E−01	6.29E−01
600	2.86E + 00	1.14E + 00	5.99E + 01	9.20E + 01	3.65E + 01	5.30E + 00	7.43E−01	1.60E + 00
700	4.23E + 00	2.08E + 00	5.16E + 01	8.82E + 01	4.28E + 01	6.66E + 00	1.41E + 00	3.07E + 00
800	5.48E + 00	3.17E + 00	4.53E + 01	8.41E + 01	4.70E + 01	7.79E + 00	2.23E + 00	4.91E + 00
900	6.56E + 00	4.31E + 00	4.04E + 01	8.00E + 01	4.99E + 01	8.68E + 00	3.15E + 00	6.99E + 00
1000	7.48E + 00	5.43E + 00	3.65E + 01	7.60E + 01	5.19E + 01	9.36E + 00	4.11E + 00	9.17E + 00
1100	8.26E + 00	6.48E + 00	3.35E + 01	7.23E + 01	5.32E + 01	9.87E + 00	5.08E + 00	1.13E + 01
1200	8.91E + 00	7.45E + 00	3.10E + 01	6.88E + 01	5.41E + 01	1.03E + 01	6.04E + 00	1.35E + 01
1300	9.46E + 00	8.34E + 00	2.89E + 01	6.57E + 01	5.46E + 01	1.05E + 01	6.97E + 00	1.55E + 01
1400	9.94E + 00	9.13E + 00	2.72E + 01	6.28E + 01	5.50E + 01	1.07E + 01	7.85E + 00	1.74E + 01
1500	1.03E + 01	9.85E + 00	2.58E + 01	6.02E + 01	5.52E + 01	1.09E + 01	8.70E + 00	1.91E + 01
1600	1.07E + 01	1.05E + 01	2.45E + 01	5.78E + 01	5.53E + 01	1.09E + 01	9.49E + 00	2.07E + 01
1700	1.10E + 01	1.11E + 01	2.34E + 01	5.57E + 01	5.53E + 01	1.10E + 01	1.02E + 01	2.22E + 01
1800	1.13E + 01	1.16E + 01	2.25E + 01	5.38E + 01	5.53E + 01	1.10E + 01	1.09E + 01	2.36E + 01
1900	1.15E + 01	1.21E + 01	2.17E + 01	5.20E + 01	5.52E + 01	1.10E + 01	1.16E + 01	2.49E + 01
2000	1.17E + 01	1.25E + 01	2.09E + 01	5.05E + 01	5.51E + 01	1.10E + 01	1.22E + 01	2.60E + 01
2100	1.19E + 01	1.29E + 01	2.03E + 01	4.90E + 01	5.50E + 01	1.10E + 01	1.28E + 01	2.71E + 01
2200	1.21E + 01	1.32E + 01	1.97E + 01	4.77E + 01	5.48E + 01	1.10E + 01	1.34E + 01	2.81E + 01
2300	1.22E + 01	1.35E + 01	1.92E + 01	4.65E + 01	5.47E + 01	1.10E + 01	1.39E + 01	2.90E + 01
2400	1.24E + 01	1.38E + 01	1.87E + 01	4.54E + 01	5.45E + 01	1.09E + 01	1.44E + 01	2.98E + 01
2500	1.25E + 01	1.41E + 01	1.83E + 01	4.44E + 01	5.44E + 01	1.09E + 01	1.48E + 01	3.06E + 01
2600	1.26E + 01	1.43E + 01	1.79E + 01	4.34E + 01	5.42E + 01	1.09E + 01	1.53E + 01	3.14E + 01
2700	1.27E + 01	1.45E + 01	1.75E + 01	4.26E + 01	5.41E + 01	1.08E + 01	1.57E + 01	3.20E + 01
2800	1.28E + 01	1.48E + 01	1.72E + 01	4.18E + 01	5.39E + 01	1.08E + 01	1.61E + 01	3.27E + 01
2900	1.29E + 01	1.49E + 01	1.69E + 01	4.10E + 01	5.37E + 01	1.08E + 01	1.64E + 01	3.33E + 01
3000	1.30E + 01	1.51E + 01	1.66E + 01	4.03E + 01	5.36E + 01	1.07E + 01	1.68E + 01	3.38E + 01

A and B refer to the M06-2X and CBS-QB3 methods, respectively. Also, S = $\sum_{i=1}^{N}ki$.

Among four centers with different hydrogens from a chemical point of view, the α hydrogen has a higher rate constant. So, n-propanol destruction begins from the α carbon center. For this center, the rate constant obtained by the CBS-QB3 method is higher than that of the M06-2X method. The rate values by the expression of $1.13 \times 10^{ - 14} \left( \frac{T}{300} \right){}^{3.87}\exp \left( { - \frac{{6.48\,{\text{kcal}}\,{\text{mol}}^{ - 1} }}{RT}} \right)$ cm^3^ molecule^−1^ s^−1^ (CBS-QB3) is ~ 2 times higher than the corresponding values at the M06-2X method ($1.62 \times 10^{ - 15} \left( \frac{T}{300} \right){}^{3.96}\exp \left( { - \frac{{5.20\,{\text{kcal}}\,{\text{mol}}^{ - 1} }}{RT}} \right)$ cm^3^ molecule^−1^ s^−1^) in moderate temperatures. This ratio increases to 4.69 times at 3000 K. The hydrogen abstraction from the hydroxyl group has the rate expression of $1.33 \times 10^{ - 14} \left( \frac{T}{300} \right){}^{3.32}\exp \left( { - \frac{{10.95\,{\text{kcal}}\,{\text{mol}}^{ - 1} }}{RT}} \right)$ (M06-2X) and $4.28 \times 10^{ - 14} \left( \frac{T}{300} \right){}^{3.31}\exp \left( { - \frac{{12.96\,{\text{kcal}}\,{\text{mol}}^{ - 1} }}{RT}} \right)$ cm^3^ molecule^−1^ s^−1^ (CBS-QB3), which is approximately similar to the rate of the H abstraction from the OH group in methanol and ethanol reactions. Also, near rate constants are attained for hydrogen transfer to imidogen from the methyl groups of n-propanol and ethanol. For β carbon of n-propanol, a good rate constant is predicted at moderate temperatures after α carbon. The rate expression of this center is $1.21 \times 10^{ - 14} \left( \frac{T}{300} \right){}^{3.79}\exp \left( { - \frac{{8.09\,{\text{kcal}}\,{\text{mol}}^{ - 1} }}{RT}} \right)$ (M06-2X) and $4.60 \times 10^{ - 15} \left( \frac{T}{300} \right){}^{3.85}\exp \left( { - \frac{{8.80\,{\text{kcal}}\,{\text{mol}}^{ - 1} }}{RT}} \right)$ cm^3^ molecule^−1^ s^−1^ (CBS-QB3). In addition, the rate constant of *P3*(*pr*) formation obtained by the CBS-QB3 method at 600, 1500, and 3000 K is 4.65, 1.10, and 0.71 times more than the generation rate of *P1*(*pr*), and is 3.32, 0.57, and 0.32 times of the rate constant of *P4*(*pr*) production, respectively. As we can see in Fig. 14, the rates of all products get close to each other at very high temperatures. Because at high temperatures, the activation energy $E_{a} = E_{b} + mRT$ is being $E_{a} \approx mRT$ for all reactions and the parameter m for all channels is near to each other as well. About the branching ratios, the parentage of *P2*(*pr*) generation, the same as the abovementioned reaction is high at low and moderate temperatures (see Table 8). However, the branching ratios of *P1*(*pr*) and *P4*(*pr*) products against *P2*(*pr*) and *P3*(*pr*) are negligible at low temperatures, but at high temperatures *P1*(*pr*) and *P4*(*pr*) generation are appreciable.

**Figure 15:**
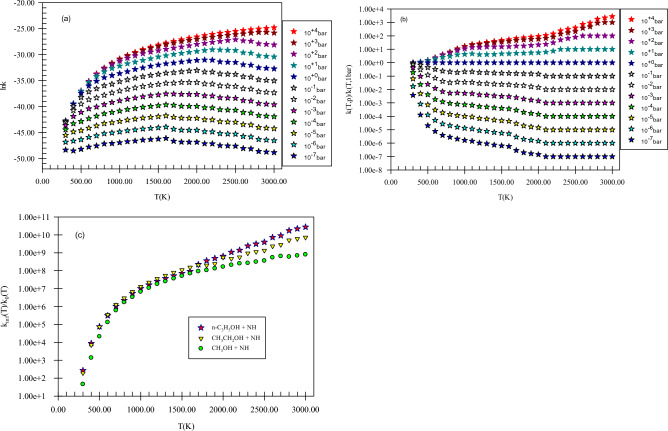
Pressure-dependent rate constants (**a**), reduced rate constants (**b**), and the ratio *k*_*∞*_*/k*_*0*_ (**c**) for H abstraction from the C_α_ center of propanol by NH in the triplet state computed at the CBS-QB3 method.

In Fig. 15, pressure-dependent rate constants, reduced rate constants, and the ratio *k*_*∞*_*/k*_*0*_ for H abstraction from the C_α_ center of propanol by NH are depicted. Our results uncovered that k(T,p) has a positive dependence on pressure. To express, how k(T,p) varies with pressure the ratio *k*_*∞*_*/k*_*0*_ is helpful as stated before (see Fig. 15c). The calculated *k*_*∞*_*/k*_*0*_ ratio for *P2*(*pr*) at 600, 1500, and 3000 K, are 3.15E + 05, 7.25E + 07, and 2.77E + 10, respectively. Also, in the pressures of 10^–2^, 10^–1^, 10, and 10^2^ bar, the reduced rate constant for *P2*(*pr*) product at 600 K, *k*(*600 K,p*)*/k*(*600 K,1 bar*), is 7.06E−02, 3.19E−01, 1.91, and 2.43, respectively. By increasing the temperature to 1500 (3000) K, the reduced rate gets the values of 2.41E−02 (1.00E−02), 1.67E−01 (1.00E−01), 4.67 (1.00E + 01), and 1.56E + 01(1.00E + 02), respectively. To have criteria for comparison with ethanol and methanol reactions, the ratios *k*(*T,p*)_pr_*/k*(*T,p*)_e_, *k*(*T,p*)_pr_*/*(*k*(*T,p*)_m_, (*k*_*∞*_*/k*_*0*_)_pr_*/*(*k*_*∞*_*/k*_*0*_)_e_, and (*k*_*∞*_*/k*_*0*_)_pr_*/*(*k*_*∞*_*/k*_*0*_)_m_ could be useful. We use the ratios *k*(*T,p*)_pr_*/k*(*T,p*)_e_ and *k*(*T,p*)_pr_*/*(*k*(*T,p*)_m_ to evaluate how the pressure-dependent rate constants change among the reactions of n-propanol, ethanol, and methanol. The ratio *k*(*T,p*)_pr_*/k*(*T,p*)_e_ at 600 K and in the pressures of 10^–2^, 10^–1^,1, 10, and 10^2^ bar is 1.62, 1.59, 1.44, 1.25, and 1.12, respectively. For 1500 K at the same pressures, the values of this ratio are 1.45, 1.41, 1.33, 1.28, and 1.13 and for 3000 K it remains constant (2.07E−1) at any pressure. And the ratio *k*(*T,p*)_pr_*/*(*k*(*T,p*)_m_ in the stated pressures is 5.44, 6.25, 6.85, 7.26, and 7.37 (at 600 K) and 1.92, 2.04, 2.28, 1.94, and 1.66 (at 1500 K) and 4.17E−02, 4.89E−02, 6.40E−02, 1.02E−01, and 2.35E−01 (at 3000 K), respectively. For the ratios (*k*_*∞*_*/k*_*0*_)_pr_*/*(*k*_*∞*_*/k*_*0*_)_e_, (*k*_*∞*_*/k*_*0*_)_pr_*/*(*k*_*∞*_*/k*_*0*_)_m_, the values 9.04 E−01 and 2.34 at 600 K and 6.54 E−01 and 1.40 at 1500 K and 3.28 and 3.43 at 3000 K are computed, respectively. Thus, in comparison with the ethanol reaction, the prior ratio has small (high) values at small and moderate (high) temperatures. But, compared to the ratio *k*_*∞*_*/k*_*0*_ for methanol, the ratio *k*_*∞*_*/k*_*0*_ for n-propanol has high values in the 300–3000 K temperature range. These results show that at high pressures, and low and moderate temperatures *k*(*T,p*) for propanol is higher than ethanol and methanol but at high temperatures, inverse behavior is seen. This may relate to a decrease in the reaction cross-section at high temperatures.

### N- butanol plus NH reaction

**Figure 16:**
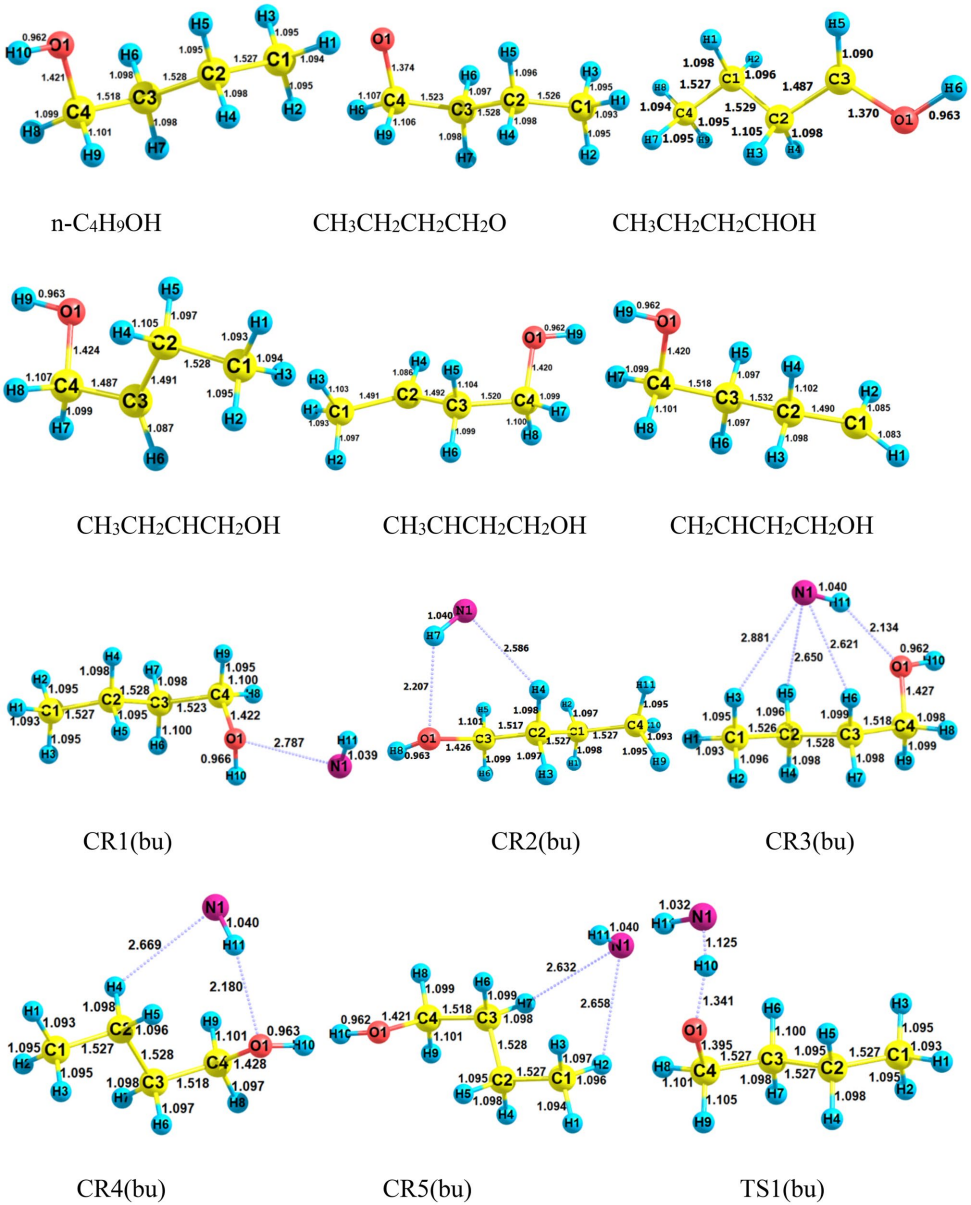

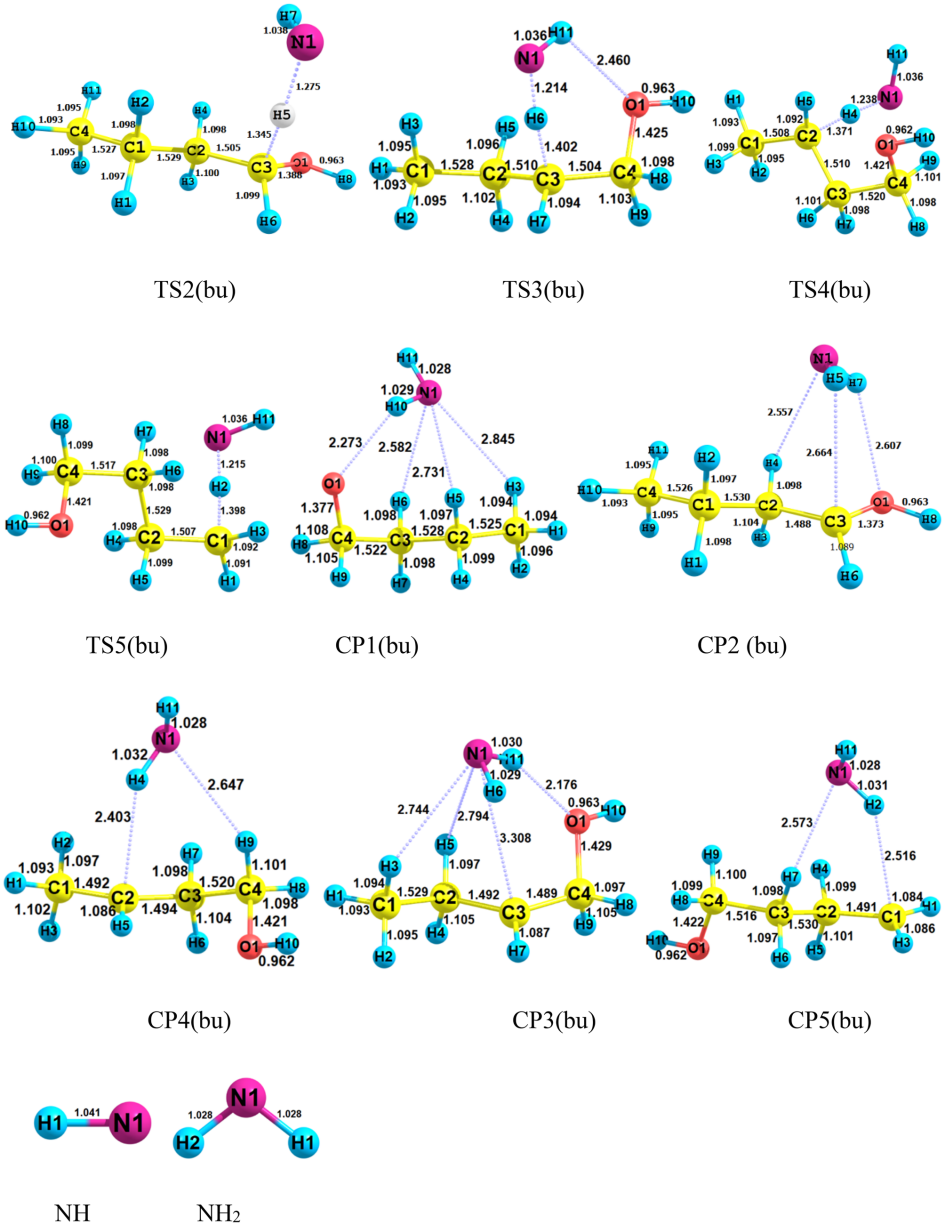
Structures of all stationary points including bond lengths (in Angstrom) in the *n*-C_4_H_9_OH + NH reaction calculated at the M06-2X/6–31 + G(d,p) level of theory.

**Figure 17:**
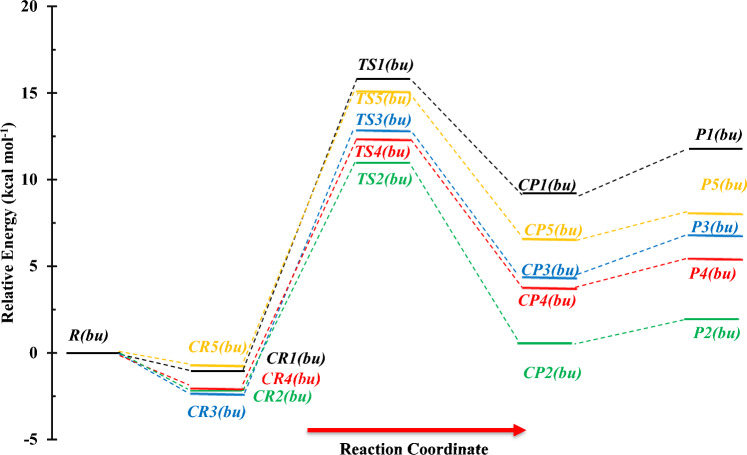
Potential energy surface of the *n*-C_4_H_9_OH + NH reaction at the triplet ground state computed by the CBS-QB3 level.

Selected geometrical parameters and structures for *n*-C_4_H_9_OH + NH reaction are represented in Fig. 16. Also, the PES profile for this reaction is sketched in Fig. 17. The calculated relative energies along with thermodynamic parameters by the M06-2X and CBS-QB3 methods are collected in Table 9. Furthermore, we have collected the data of other conformers of n-butanol and associated reactions in the supplementary material (please see Supplementary Tables S43–S62 and Supplementary Figs. S9–S18). From a chemical point of view, butanol has different hydrogen in five positions, which may react with imidogen in atmospheric conditions. So, five different paths are designed as

$$R\left( {bu} \right) \to CR1\left( {bu} \right) \to TS1\left( {bu} \right) \to CP1\left( {bu} \right) \to P1\left( {bu} \right) \, \left( {CH_{3} CH_{2} CH_{2} CH_{2} O \, + \, NH_{2} } \right)\quad \left( {{\text{R}}_{{1}} (bu)} \right)$$

$$R\left( {bu} \right) \to CR2\left( {bu} \right) \to TS2\left( {bu} \right) \to CP2\left( {bu} \right) \to P2\left( {bu} \right) \, (CH_{3} CH_{2} CH_{2} CHOH + \, NH_{2} )\quad \left( {{\text{R}}_{{2}} (bu)} \right)$$

$$R\left( {bu} \right) \to CR3\left( {bu} \right) \to TS3\left( {bu} \right) \to CP3\left( {bu} \right) \to P3\left( {bu} \right)(CH_{3} CH_{2} CHCH_{2} OH + \, NH_{2} )\quad \left( {{\text{R}}_{{3}} (bu)} \right)$$

$$R\left( {bu} \right) \to CR4\left( {bu} \right) \to TS4\left( {bu} \right) \to CP4\left( {bu} \right) \to P4\left( {bu} \right)(CH_{3} CHCH_{2} CH_{2} OH + \, NH_{2} )\quad \left( {{\text{R}}_{{4}} (bu)} \right)$$

$$R\left( {bu} \right) \to CR5\left( {bu} \right) \to TS5\left( {bu} \right) \to CP5\left( {bu} \right) \to P5\left( {bu} \right) \, (CH_{2} CH_{2} CH_{2} CH_{2} OH + \, NH_{2} )\quad \left( {{\text{R}}_{{5}} (bu)} \right)$$

**Table 9:** Relative energies and thermodynamic parameters for stationary points of the n-C_4_H_9_OH + NH reaction. (Unit of all numbers is kcal mol^-1^).

Species	∆(E + ZPE)(A)	∆E(0 K)(B)	∆E˚(A)	∆E˚(B)	∆H˚(A)	∆H˚(B)	∆G˚(A)	∆G˚(B)	T∆S˚(A)	T∆S˚(B)
R(bu)	0.00	0.00	0.00	0.00	0.00	0.00	0.00	0.00	0.00	0.00
CR1(bu)	− 3.38	− 1.05	− 3.18	0.50	− 3.77	− 0.39	3.73	4.69	− 7.51	− 5.08
CR2(bu)	− 3.67	− 2.18	− 3.79	− 0.77	− 4.38	− 1.66	4.41	3.79	− 8.80	− 5.45
CR3(bu)	− 4.90	− 2.41	− 4.68	− 1.06	− 5.27	− 1.95	2.81	3.30	− 8.08	− 5.25
CR4(bu)	− 3.91	− 2.09	− 3.62	− 0.76	− 4.21	− 1.65	3.58	4.00	− 7.79	− 5.64
CR5(bu)	− 1.65	− 0.75	− 1.08	1.26	− 1.67	0.37	5.09	3.64	− 6.76	− 3.27
TS1(bu)	14.32	15.81	14.06	16.56	13.47	15.67	22.23	23.28	− 8.77	− 7.61
TS2(bu)	10.13	10.97	10.13	11.93	9.53	11.04	17.84	18.42	− 8.30	− 7.38
TS3(bu)	11.80	12.81	11.73	13.65	11.14	12.76	19.89	20.87	− 8.75	− 8.11
TS4(bu)	11.99	12.29	11.94	13.31	11.35	12.42	19.99	19.77	− 8.64	− 7.34
TS5(bu)	14.75	15.06	14.66	16.00	14.07	15.11	22.57	22.31	− 8.50	− 7.20
CP1(bu)	7.88	9.21	8.12	10.90	7.53	10.01	15.53	15.50	− 8.00	− 5.49
CP2(bu)	0.97	0.44	1.73	2.47	1.14	1.58	7.76	5.85	− 6.62	− 4.27
CP3(bu)	3.61	4.32	4.53	6.47	3.94	5.58	10.66	9.08	− 6.72	− 3.50
CP4(bu)	3.89	3.70	4.51	5.90	3.92	5.01	10.87	8.99	− 6.95	− 3.99
CP5(bu)	7.35	6.54	7.73	8.72	7.14	7.83	14.83	11.66	− 7.69	− 3.83
P1(bu)(CH_3_CH_2_CH_2_CH_2_O + NH_2_)	11.94	11.77	12.07	12.81	12.07	12.51	11.01	10.87	1.06	1.64
P2(bu)(CH_3_CH_2_CH_2_CHOH + NH_2_)	3.75	1.94	4.29	3.23	4.29	2.93	2.23	0.71	2.06	2.22
P3(bu)(CH_3_CH_2_CHCH_2_OH + NH_2_)	8.55	6.75	9.15	8.23	9.15	7.93	6.90	4.95	2.25	2.98
P4(Bu)(CH_3_CHCH_2_CH_2_OH + NH_2_)	7.19	5.40	7.72	6.83	7.72	6.53	5.85	3.89	1.87	2.64
P5(Bu)(CH_2_CH_2_CH_2_CH_2_OH + NH_2_)	10.10	8.02	10.57	9.43	10.57	9.13	8.99	6.71	1.58	2.42

A and B refer to the M06-2X and CBS-QB3 methods, respectively.

As the predicted paths show, by the initial association of the reactants, five different pre-reactive complexes are obtained due to different locations and orientations of imidogen around n-butanol. For starting the n-butanol plus NH reaction, five different prereactive complexes are predicted by IRC calculations. More exploration of the obtained complexes by AIM theory shows that the structure of some complexes contains not only ring critical points but also a cage critical point. The first complex *CR1*(*bu*) the same as *CR1*(*m*) has only a van der Waals interaction between the N and O atoms ($\rho_{(LCP)} =$ 0.0128 e bohr^−3^ and $\nabla^{2} \rho =$ 0.0482 e bohr^−5^). In *CR2*(*bu*), the ring structure is the same as the rings of *CR2*(*e*) and *CR2*(*pr*) with a close value of density of ring critical points and its Laplacian ($\rho_{(RCP)} =$ 0.0063 e bohr^−3^ and $\nabla^{2} \rho_{(RCP)} =$ 0.0270 e bohr^−5^). The cage critical point is seen ($\rho_{(Cage)} =$ 0.0056 e bohr^−3^ and $\nabla^{2} \rho_{(Cage)} =$ 0.0240 e bohr^−5^) for *CR3*(*bu*) complex. This cage is formed by four intermolecular interactions. Three of that interactions are van der Waals type and one is hydrogen bond type ($\rho_{(LCP)} =$ 0.0179 e bohr^−3^ and $\nabla^{2} \rho_{(LCP)} =$ 0.0548 e bohr^−5^). The *CR4*(*bu*) is a seven-membered ring-like complex ($\rho_{(RCP)} =$ 0.0055 e bohr^−3^ and $\nabla^{2} \rho_{(RCP)} =$ 0.0222 e bohr^−5^) with two intermolecular interactions. In the *CR5*(*bu*) structure, n-butanol and imidogen moieties have two weak van der Waals interactions in a six-membered ring ($\rho_{(RCP)} =$ 0.0047 e bohr^−3^ and $\nabla^{2} \rho_{(RCP)} =$ 0.0160 e bohr^−5^). The equilibrium expressions of all complexes in the n-butanol plus NH reaction are as follows:

37 $$K_{1A} = 8.62 \times 10^{ - 26} \left( \frac{T}{300} \right){}^{1.46 \pm 0.00}\exp \left[ {\frac{{(4.07 \pm 0.00){\text{kcal}}\,{\text{mol}}^{ - 1} }}{RT}} \right],$$

38 $$K_{1B} = 6.80 \times 10^{ - 25} \left( \frac{T}{300} \right){}^{2.44 \pm 0.00}\exp \left[ {\frac{{(1.89 \pm 0.01){\text{kcal}}\,{\text{mol}}^{ - 1} }}{RT}} \right],$$

39 $$K_{2A} = 9.51 \times 10^{ - 27} \left( \frac{T}{300} \right){}^{1.42 \pm 0.01}\exp \left[ {\frac{{(4.69 \pm 0.01){\text{kcal}}\,{\text{mol}}^{ - 1} }}{RT}} \right],$$

40 $$K_{2B} = 3.60 \times 10^{ - 25} \left( \frac{T}{300} \right){}^{2.42 \pm 0.01}\exp \left[ {\frac{{(3.16 \pm 0.01){\text{kcal}}\,{\text{mol}}^{ - 1} }}{RT}} \right],$$

41 $$K_{3A} = 1.19 \times 10^{ - 26} \left( \frac{T}{300} \right){}^{2.41 \pm 0.01}\exp \left[ {\frac{{(6.17 \pm 0.01){\text{kcal}}\,{\text{mol}}^{ - 1} }}{RT}} \right],$$

42 $$K_{3B} = 4.99 \times 10^{ - 25} \left( \frac{T}{300} \right){}^{2.43 \pm 0.01}\exp \left[ {\frac{{(3.45 \pm 0.01){\text{kcal}}\,{\text{mol}}^{ - 1} }}{RT}} \right],$$

43 $$K_{4A} = 1.89 \times 10^{ - 26} \left( \frac{T}{300} \right){}^{2.43 \pm 0.01}\exp \left[ {\frac{{(6.06 \pm 0.01){\text{kcal}}\,{\text{mol}}^{ - 1} }}{RT}} \right],$$

44 $$K_{4B} = 2.55 \times 10^{ - 25} \left( \frac{T}{300} \right){}^{2.42 \pm 0.01}\exp \left[ {\frac{{(3.16 \pm 0.01){\text{kcal}}\,{\text{mol}}^{ - 1} }}{RT}} \right],$$

45 $$K_{3A} = 1.13 \times 10^{ - 25} \left( \frac{T}{300} \right){}^{2.47 \pm 0.00}\exp \left[ {\frac{{(2.57 \pm 0.01){\text{kcal}}\,{\text{mol}}^{ - 1} }}{RT}} \right],$$

46 $$K_{4B} = 1.48 \times 10^{ - 23} \left( \frac{T}{300} \right){}^{2.50 \pm 0.00}\exp \left[ {\frac{{(1.12 \pm 0.00){\text{kcal}}\,{\text{mol}}^{ - 1} }}{RT}} \right].$$

**Figure 18:**
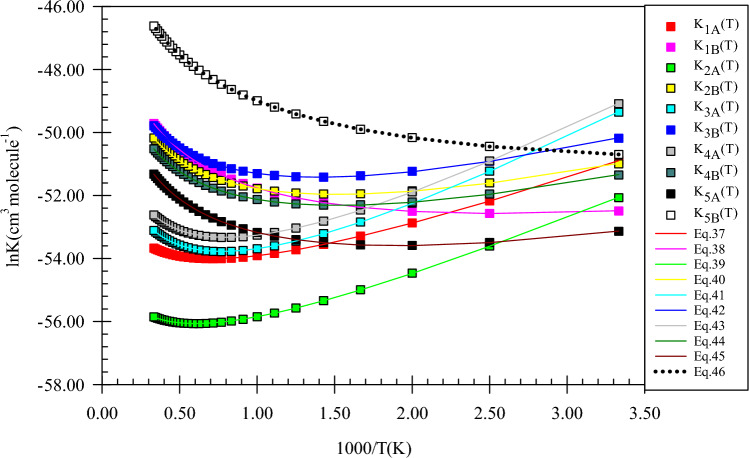
Equilibrium constants and associated fitted expressions of all prereactive complexes in the n-butanol plus imidogen reaction calculated at the M06-2X (**A**) and CBS-QB3 (**B**) methods.

In Fig. 18, all computed equilibrium constants and their fitted expressions are displayed. The value of the equilibrium constant at the CBS-QB3 method for the *CR1*(*bu*) complex at 600, 1500, and 3000 K is 1.81E−23, 6.45E−23, and 2.57E−22 cm^3^ molecule^−1^, respectively. Also, the ratios of K_CR1(bu)_/K_CR2(bu)_, K_CR1(bu)_/K_CR3(bu)_, K_CR1(bu)_/K_CR4(bu)_, and K_CR1(bu)_/K_CR5(bu)_ are 0.66, 0.37, 0.93, and 0.08 (600 K), 1.27, 0.82, 1.79, and 0.05 (1500 K), and 1.59, 1.08, 2.23, and 0.05 (3000 K), respectively. To compare with the previous reactions, the ratios of K_CR1(bu)_/K_CR1(m)_, K_CR1(bu)_/K_CR1(e)_, and K_CR1(bu)_/K_CR1(pr)_ may be helpful. The mentioned ratios at 600 K are 1.86, 1.66, and 0.58, respectively. Our results show that the ratio of K_CR1(bu)_/K_CR1(m)_, K_CR1(bu)_/K_CR1(e)_ are approximately constant and the ratio K_CR1(bu)_/K_CR1(pr)_ has a small variation over 300–3000 K temperature range. For the second complexes of the discussed reactions, the value of the same ratios, K_CR2(bu)_/K_CR2(m)_, K_CR2(bu)_/K_CR2(e)_, and K_CR2(bu)_/K_CR2(pr),_ at 600 K are 0.77, 1.01, and 0.68, respectively. It should be noted that the ratio K_CR2(bu)_/K_CR2(m)_ remains 0.77 over the 300–3000 K temperature range. Also, other ratios change slightly at the mentioned temperature range. The ratios K_CR3(bu)_/K_CR3(e)_ and K_CR3(bu)_/K_CR3(pr)_ are 17.82, and 1.21 at 600 K, respectively. The same as K_CR2(bu)_/K_CR2(m)_ ratio, the ratio K_CR3(bu)_/K_CR3(pr)_ is constant over the 300–3000 K temperature range, but the ratio K_CR3(bu)_/K_CR3(e)_ is increased to 45.91 at 1500 K and 92.64 at 3000 K. Finally, the ratio K_CR4(bu)_/K_CR4(pr)_ is 0.03, 0.01, and 0.01 at 600, 1500, and 3000 K, respectively.

In path R_1_(bu), we first study the hydrogen abstraction reaction of the OH group by NH the same as the above reactions. The energy barrier of the R_1_(bu) path is high similar to methanol, ethanol, and *n*-propanol reactions. In this path, the product complex *CP1*(*bu*) formation happens when a barrier of 16.86 kcal/mol is provided. The shift of the hydrogen atom from OH of *n*-CH_3_(CH_2_)_3_OH to the nitrogen of the NH fragment is confirmed by IRC calculation. Thus, in the structure of *TS1*, the H–O bond is breaking up (with a length of 1.398 Å) and, the N–H bond is forming (with a length of 1.215 Å). The barrier energy of the R_1_(bu) path is 1.40 kcal mol^−1^ lower than the R_1_(pr) path. In the final step, the *CP1*(*bu*) complex converts to the final product *P1*(*bu*) when the interactions in the eight-membered ring structure are ruptured. The type of interactions in the mentioned ring-like structure is three van der Waals interactions and one hydrogen bond interaction. The van der Waals interactions are between the 1N and 3H atoms ($\rho_{(LCP)} =$ 0.0062 e Bohr^−3^ and $\nabla^{2} \rho_{(LCP)} =$ 0.0203 e bohr^−5^), the 1N and 5H atoms ($\rho_{(LCP)} =$ 0.0072 e Bohr^−3^ and $\nabla^{2} \rho_{(LCP)} =$ 0.0262 e bohr^−5^), and the 6H and 1N atoms ($\rho_{(LCP)} =$ 0.0089 e bohr^−3^ and $\nabla^{2} \rho_{(LCP)} =$ 0.0295 e bohr^−5^). And the hydrogen bond is located between the 1O and 10H atoms($\rho_{(LCP)} =$ 0.0142 e bohr^−3^ and $\nabla^{2} \rho_{(LCP)} =$ 0.0434 e bohr^−5^).

In the second path, the *CP2*(*bu*) product complex is obtained while alpha hydrogen of n-butanol migrates to imidogen. According to the calculated energies, this process is more feasible than the R_1_(bu) reaction due to having a lower energy barrier (13.14 kcal/mol). Also, this path has a higher energy barrier than R_2_(pr). The difference is 2.18 kcal mol^-1^. In transition state 2, the length of the dissociating bond between the 2C and 4H atoms is 1.371 Å, and the length of the forming bond between the 1N and 4H atoms is 1.238 Å. The *P2*(*bu*) product is released after opening up the formed ring between the two moieties. Our results revealed that two ring critical points are situated among the 1N, 7H, 1O, 4H, and 3C atoms ($\rho_{(RCP1)} =$ 0.0073 e bohr^−3^ and $\nabla^{2} \rho_{(RCP1)} =$ 0.0278 e bohr^−5^) and the 1N, 5H, 2C, 5H, and 3C atoms ($\rho_{(RCP2)} =$ 0.0074 e bohr^−3^ and $\nabla^{2} \rho_{(RCP2)} =$ 0.0280 e bohr^−5^).

The beta hydrogen undergoes the reaction with NH via the *CR3*(*bu*) complex. This reaction requires an energy barrier of 15.22 kcal mol^−1^ for *CP3*(*bu*) generation. In comparison with the beta hydrogen of n-propanol, this hydrogen needs 0.28 kcal mol^−1^ lower energy for migration. Also, the length of both breaking and forming bonds in *TS3*(*bu*) is 0.003 Å higher than the respective bonds in *TS3*(*pr*). It is worth mentioning that the post-reactive complex *CP3*(*bu*) has an eight-membered ring structure. This structure involves three van der Waals bonds, between the 1N and 3H atoms ($\rho_{(LCP)} =$ 0.0061 e bohr^−3^ and $\nabla^{2} \rho_{(LCP)} =$ 0.0210 e bohr^−5^), the 5H and 1N atoms ($\rho_{(LCP)} =$ 0.0064 e bohr^−3^ and $\nabla^{2} \rho_{(LCP)} =$ 0.0236 e bohr^−5^), and the 1N and 3C atoms ($\rho_{(LCP)} =$ 0.0068 e bohr^−3^ and $\nabla^{2} \rho_{(LCP)} =$ 0.0192 e bohr^−5^). Also, there is a hydrogen bond interaction between the 11H and 1O atoms ($\rho_{(LCP)} =$ 0.0163 e bohr^−3^ and $\nabla^{2} \rho_{(LCP)} =$ 0.0514 e bohr^−5^). These interactions lead to the stability of *CP3*(*bu*) compared to *P3*(*bu*).

It is also possible that the reaction of gamma hydrogen observes in the gas phase. The feasible path for this hydrogen is R_4_(bu) which starts with the prereactive collision complex *CR4*(*bu*). The saddle point of this path *TS4*(*bu*) has 14.38 kcal mol^−1^ energy barrier which is 2.83 kcal mol^−1^ lower than the saddle point of *TS4*(*pr*). The transition state 4 involves cleavages of the bond between the atoms 2C and 4H (with a length of 1.371 Å) and at the same time, the bond between the atoms 1N and 4H is formed (with a length of 1.238 Å). Also, the formed post-reactive complex *CP4*(*bu*) has 4.08 kcal mol^−1^ lower relative energy than *CP4*(*pr*). These differences go back to the number and strength of the interactions in both complexes. For *CP4*(*bu*), AIM calculations show that it has two van der Waals bonds located between the 2C and 4H atoms ($\rho_{(LCP)} =$ 0.0132 e bohr^−3^ and $\nabla^{2} \rho_{(LCP)} =$ 0.0358 e bohr^−5^) and the 9H and 1N atoms ($\rho_{(LCP)} =$ 0.0075 e bohr^−3^ and $\nabla^{2} \rho_{(LCP)} =$ 0.0250 e bohr^−5^), causing to form a six-membered ring structure. However, after breaking the mentioned interaction, the *P4*(*bu*) product is yielded. R_5_(bu) is the last pathway that shows the simulation of the hydrogen abstraction from the methyl group with an energy barrier of 15.81 kcal mol^−1^. This path after R_1_(bu) has the highest energy barrier. Also, the post reactive complex of this path after *CR1*(*bu*) has the highest relative energy. This may relate to two weak van der Waals interactions that are the reason for the formation of a six-membered ring between the NH_2_ and CH_2_CH_2_CH_2_CH_2_OH moieties. Accordingly, the product of this path, *P5*(*bu*), is released in the exit channel after getting small energy (i.e., 1.48 kcal mol^−1^).

**Figure 19:**
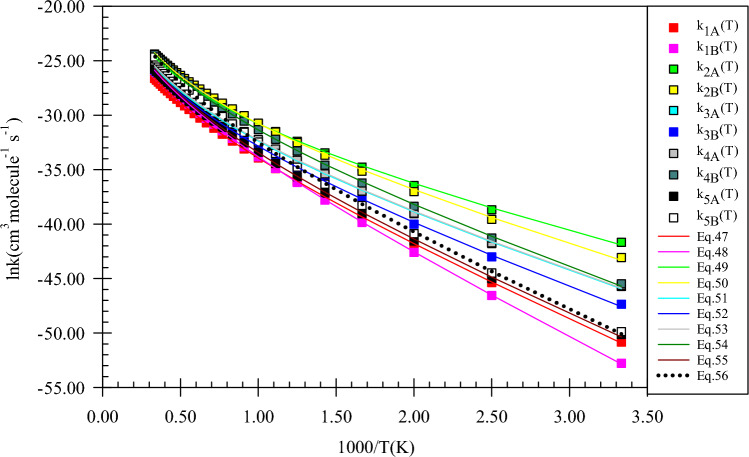
Graph of the high-pressure limit rate constants (cm^3^ molecule^−1^ s^−1^) and fitted non-Arrhenius expressions for the gas-phase formation of the *P1*(*bu*), *P2*(*bu*), *P3*(*bu*), *P4*(*bu*), and *P5*(*bu*) products calculated by the TST/Eckart theory at the M06-2X (**A**) and CBS-QB3 (**B**) methods.

In the sequel, the reaction of n-butanol plus imidogen is evaluated kinetically. Figure 19 displays the behavior of the reaction at different temperatures. The rates of all paths are computed and collected in Supplementary Table S16. Also, the fitted rate expressions are as follows:

47 $$k_{1A} = 8.94 \times 10^{ - 15} \left( \frac{T}{300} \right){}^{3.32 \pm 0.03}\exp \left[ { - \frac{{(11.07 \pm 0.05){\text{kcal}}\,{\text{mol}}^{ - 1} }}{RT}} \right],$$

48 $$k_{1B} = 2.46 \times 10^{ - 14} \left( \frac{T}{300} \right){}^{3.31 \pm 0.03}\exp \left[ { - \frac{{(12.83 \pm 0.05){\text{kcal}}\,{\text{mol}}^{ - 1} }}{RT}} \right],$$

49 $$k_{2A} = 5.83 \times 10^{ - 15} \left( \frac{T}{300} \right){}^{3.94 \pm 0.10}\exp \left[ { - \frac{{(5.46 \pm 0.17){\text{kcal}}\,{\text{mol}}^{ - 1} }}{RT}} \right],$$

50 $$k_{2B} = 1.26 \times 10^{ - 14} \left( \frac{T}{300} \right){}^{3.85 \pm 0.09}\exp \left[ { - \frac{{(6.75 \pm 0.16){\text{kcal}}\,{\text{mol}}^{ - 1} }}{RT}} \right],$$

51 $$k_{3A} = 4.11 \times 10^{ - 15} \left( \frac{T}{300} \right){}^{3.82 \pm 0.09}\exp \left[ { - \frac{{7.64 \pm 0.15){\text{kcal}}\,{\text{mol}}^{ - 1} }}{RT}} \right],$$

52 $$k_{3B} = 4.18 \times 10^{ - 15} \left( \frac{T}{300} \right){}^{3.83 \pm 0.09}\exp \left[ { - \frac{{(8.63 \pm 0.15){\text{kcal}}\,{\text{mol}}^{ - 1} }}{RT}} \right],$$

53 $$k_{4A} = 4.01 \times 10^{ - 15} \left( \frac{T}{300} \right){}^{3.90 \pm 0.09}\exp \left[ { - \frac{{(7.59 \pm 0.16){\text{kcal}}\,{\text{mol}}^{ - 1} }}{RT}} \right],$$

54 $$k_{4B} = 1.54 \times 10^{ - 14} \left( \frac{T}{300} \right){}^{3.85 \pm 0.09}\exp \left[ { - \frac{{(8.29 \pm 0.15){\text{kcal}}\,{\text{mol}}^{ - 1} }}{RT}} \right],$$

55 $$k_{5A} = 5.85 \times 10^{ - 15} \left( \frac{T}{300} \right){}^{3.87 \pm 0.09}\exp \left[ { - \frac{{(10.46 \pm 0.15){\text{kcal}}\,{\text{mol}}^{ - 1} }}{RT}} \right],$$

56 $$k_{5B} = 1.99 \times 10^{ - 14} \left( \frac{T}{300} \right){}^{3.84 \pm 0.08}\exp \left[ { - \frac{{(11.05 \pm 0.15){\text{kcal}}\,{\text{mol}}^{ - 1} }}{RT}} \right].$$

Our calculated rate constants for hydrogen atom abstraction from the OH group of n-butanol indicate that this group is less reactive at low temperatures. The fitted rate expressions at the M06-2X method, $8.94 \times 10^{ - 15} \left( \frac{T}{300} \right){}^{3.32}\exp \left( { - \frac{{11.07\,{\text{kcal}}\,{\text{mol}}^{ - 1} }}{RT}} \right),$ cm^3^ molecule^−1^ s^−1^, and the CBS-QB3 method, $2.46 \times 10^{ - 14} \left( \frac{T}{300} \right){}^{3.31}\exp \left( { - \frac{{12.83\,{\text{kcal}}\,{\text{mol}}^{ - 1} }}{RT}} \right),$ cm^3^ molecule^−1^ s^−1^, confirm this statement due to high activation energy. It might be useful to argue about the differences in rate constants by their ratios for the same centers in different alcohols. The ratios k_R1(bu)_/k_R1(m)_, k_R1(bu)_/k_R1(e)_, and k_R1(bu)_/k_R1(pr)_ at 600 K are 1.93, 1.37, and 0.64, respectively. These ratios are 1.37, 1.07, and 0.60 at 1500 K and are 1.22, 0.99, and 0.59 at 3000 K, respectively. By this comparison, we understand that there is no sensible difference among the rate constants of the OH group of simple alcohols in the reaction with NH. After the hydroxyl group, the methyl group has less reactivity. This forecast coincides with the activation energies predicted by the rate expressions of $5.85 \times 10^{ - 15} \left( \frac{T}{300} \right){}^{3.87}\exp \left( { - \frac{{10.46\,{\text{kcal}}\,{\text{mol}}^{ - 1} }}{RT}} \right)$ cm^3^ molecule^−1^ s^−1^ (M06-2X) and $1.99 \times 10^{ - 14} \left( \frac{T}{300} \right){}^{3.84}\exp \left( { - \frac{{11.05\,{\text{kcal}}\,{\text{mol}}^{ - 1} }}{RT}} \right)$ cm^3^ molecule^−1^ s^−1^ (CBS-QB3). On the other hand, the rate constant of H abstraction from the methyl group of n-butanol is 12.02 and 2.15 times of the methyl group of ethanol and n-propanol at 600 K (7.89 and 1.13 times at 1500 K, and 6.94 and 0.92 times at 3000 K), respectively, which shows n-propanol and n-butanol have close rate constants. So, the methyl group of simple alcohols has a similar rate for H abstraction by NH moiety as the carbon chain length increases. The centers of β and γ carbons have an almost similar rate in a hydrogen atom transfer to NH. The α carbon similar to the previous reactions is more reactive than the other centers according to the rate expressions of $5.83 \times 10^{ - 15} \left( \frac{T}{300} \right){}^{3.94}\exp \left( { - \frac{{5.46\,{\text{kcal}}\,{\text{mol}}^{ - 1} }}{RT}} \right)$ cm^3^ molecule^−1^ s^−1^ (M06-2X) and $1.26 \times 10^{ - 14} \left( \frac{T}{300} \right){}^{3.85}\exp \left( { - \frac{{6.75\,{\text{kcal}}\,{\text{mol}}^{ - 1} }}{RT}} \right)$ cm^3^ molecule^−1^ s^−1^ (CBS-QB3). These results accompanied by the above-discussed reactions lead us to conclude that the connected carbon to the OH group and also the middle carbons of the carbon chain may have higher reactivity with active species than other centers. Also, these paths have higher branching ratios (see Table 10) at moderate temperatures. Analogous to methanol, ethanol, and n-propanol, in the reaction of n-butanol plus NH reaction, the rate of Cα reaction is higher than in the other centers. This statement because of having smaller activation energies is obviously seen in the rate expressions $k_{2A} = 5.83 \times 10^{ - 15} \left( \frac{T}{300} \right){}^{3.94 \pm 0.10}\exp \left[ { - \frac{{(5.46 \pm 0.17){\text{kcal}}\,{\text{mol}}^{ - 1} }}{RT}} \right]$ (CBS-QB3) and $k_{2B} = 1.26 \times 10^{ - 14} \left( \frac{T}{300} \right){}^{3.85 \pm 0.09}\exp \left[ { - \frac{{(6.75 \pm 0.16){\text{kcal}}\,{\text{mol}}^{ - 1} }}{RT}} \right]$ cm^3^ molecule^−1^ s^−1^ (M06-2X). Also, the ratios k_R2(bu)_/k_R2(m)_, k_R2(bu)_/k_R2(e)_, and k_R2(bu)_/k_R2(pr)_ are 7.62, 1.38, and 0.88 (600 K), 2.41, 1.24, and 0.99 (1500 K), and 1.63, 1.19, and 1.02 (3000 K), respectively.

**Table 10:** Branching ratios of all channels in the *n*-butanol plus NH reaction.

T(K)	k_1_(A)/S(A)	k_1_(B)/S(B)	k_2_(A)/S(A)	k_2_(B)/S(B)	k_3_(A)/S(A)	k_3_(B)/S(B)	k_4_(A)/S(A)	k_4_(B)/S(B)	k_5_(A)/S(A)	k_5_(B)/S(B)
300	1.00E−02	5.49E−03	9.65E + 01	9.05E + 01	1.68E + 00	1.24E + 00	1.83E + 00	8.17E + 00	2.02E−02	1.01E−01
400	1.13E−01	7.41E−02	9.14E + 01	8.21E + 01	4.05E + 00	2.58E + 00	4.28E + 00	1.47E + 01	1.61E−01	5.86E−01
500	3.87E−01	2.80E−01	8.58E + 01	7.48E + 01	6.46E + 00	3.75E + 00	6.83E + 00	1.96E + 01	5.25E−01	1.57E + 00
600	8.02E−01	6.20E-01	8.05E + 01	6.87E + 01	8.50E + 00	4.66E + 00	9.09E + 00	2.31E + 01	1.12E + 00	2.93E + 00
700	1.29E + 00	1.05E + 00	7.58E + 01	6.35E + 01	1.01E + 01	5.36E + 00	1.09E + 01	2.56E + 01	1.88E + 00	4.51E + 00
800	1.78E + 00	1.51E + 00	7.16E + 01	5.92E + 01	1.14E + 01	5.87E + 00	1.24E + 01	2.73E + 01	2.74E + 00	6.15E + 00
900	2.26E + 00	1.97E + 00	6.81E + 01	5.55E + 01	1.24E + 01	6.26E + 00	1.36E + 01	2.85E + 01	3.65E + 00	7.77E + 00
1000	2.71E + 00	2.42E + 00	6.50E + 01	5.25E + 01	1.32E + 01	6.54E + 00	1.46E + 01	2.93E + 01	4.56E + 00	9.32E + 00
1100	3.12E + 00	2.84E + 00	6.23E + 01	4.98E + 01	1.38E + 01	6.76E + 00	1.53E + 01	2.98E + 01	5.45E + 00	1.08E + 01
1200	3.49E + 00	3.24E + 00	6.00E + 01	4.76E + 01	1.42E + 01	6.92E + 00	1.60E + 01	3.02E + 01	6.31E + 00	1.21E + 01
1300	3.82E + 00	3.60E + 00	5.80E + 01	4.56E + 01	1.46E + 01	7.04E + 00	1.65E + 01	3.04E + 01	7.12E + 00	1.33E + 01
1400	4.12E + 00	3.93E + 00	5.62E + 01	4.39E + 01	1.49E + 01	7.13E + 00	1.69E + 01	3.05E + 01	7.88E + 00	1.45E + 01
1500	4.39E + 00	4.24E + 00	5.46E + 01	4.24E + 01	1.52E + 01	7.20E + 00	1.72E + 01	3.06E + 01	8.60E + 00	1.55E + 01
1600	4.64E + 00	4.52E + 00	5.32E + 01	4.11E + 01	1.54E + 01	7.26E + 00	1.75E + 01	3.07E + 01	9.27E + 00	1.64E + 01
1700	4.87E + 00	4.78E + 00	5.20E + 01	4.00E + 01	1.55E + 01	7.30E + 00	1.78E + 01	3.07E + 01	9.89E + 00	1.73E + 01
1800	5.07E + 00	5.02E + 00	5.08E + 01	3.89E + 01	1.56E + 01	7.33E + 00	1.80E + 01	3.07E + 01	1.05E + 01	1.81E + 01
1900	5.26E + 00	5.24E + 00	4.98E + 01	3.80E + 01	1.58E + 01	7.35E + 00	1.81E + 01	3.06E + 01	1.10E + 01	1.88E + 01
2000	5.43E + 00	5.44E + 00	4.89E + 01	3.71E + 01	1.58E + 01	7.37E + 00	1.83E + 01	3.06E + 01	1.15E + 01	1.95E + 01
2100	5.59E + 00	5.63E + 00	4.81E + 01	3.64E + 01	1.59E + 01	7.38E + 00	1.84E + 01	3.05E + 01	1.20E + 01	2.01E + 01
2200	5.74E + 00	5.81E + 00	4.73E + 01	3.57E + 01	1.60E + 01	7.39E + 00	1.85E + 01	3.05E + 01	1.24E + 01	2.07E + 01
2300	5.87E + 00	5.97E + 00	4.66E + 01	3.50E + 01	1.60E + 01	7.39E + 00	1.86E + 01	3.04E + 01	1.29E + 01	2.12E + 01
2400	6.00E + 00	6.13E + 00	4.60E + 01	3.45E + 01	1.61E + 01	7.40E + 00	1.87E + 01	3.03E + 01	1.33E + 01	2.17E + 01
2500	6.11E + 00	6.27E + 00	4.54E + 01	3.39E + 01	1.61E + 01	7.40E + 00	1.88E + 01	3.03E + 01	1.36E + 01	2.22E + 01
2600	6.22E + 00	6.40E + 00	4.48E + 01	3.34E + 01	1.61E + 01	7.40E + 00	1.89E + 01	3.02E + 01	1.40E + 01	2.26E + 01
2700	6.33E + 00	6.53E + 00	4.43E + 01	3.30E + 01	1.62E + 01	7.40E + 00	1.89E + 01	3.01E + 01	1.43E + 01	2.30E + 01
2800	6.42E + 00	6.65E + 00	4.38E + 01	3.26E + 01	1.62E + 01	7.39E + 00	1.90E + 01	3.00E + 01	1.46E + 01	2.34E + 01
2900	6.51E + 00	6.76E + 00	4.34E + 01	3.22E + 01	1.62E + 01	7.39E + 00	1.90E + 01	3.00E + 01	1.49E + 01	2.37E + 01
3000	6.60E + 00	6.87E + 00	4.30E + 01	3.18E + 01	1.62E + 01	7.39E + 00	1.90E + 01	2.99E + 01	1.52E + 01	2.40E + 01

A and B refer to the M06-2X and CBS-QB3 methods, respectively. Also, S = $\sum_{i=1}^{N}ki$.

Our computed branching ratios for the n-butanol and imidogen reaction are listed in Table 10. This table shows that *P2*(*bu*) has a higher percentage of generation at lower temperatures. At moderate temperatures, *P3*(*bu*) and P4(bu) gradually become important. It should be noted that *P4*(*bu*) has a higher branching ratio than *P3*(*bu*). Above moderate temperatures and especially at high temperatures, *P5*(*bu*) also become more and more important but *P1*(*bu*) remains unimportant.

**Figure 20:**
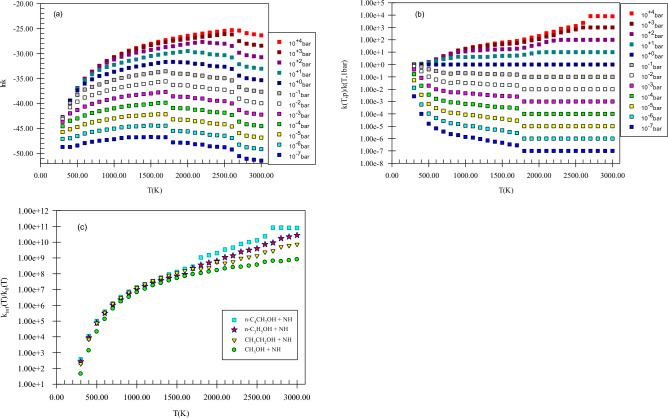
Pressure-dependent rate constants (**a**), reduced rate constants (**b**), and the ratio *k*_*∞*_*/k*_*0*_ (**c**) for H abstraction from the C_α_ center of n-butanol by NH in the triplet state computed at the CBS-QB3 method.

For n-butanol with NH reaction, the pressure-dependent and reduced rate constants along with the ratio *k*_*∞*_*/k*_*0*_ are displayed in Fig. 20. Through Figs. 5a, 10a, 15a, and 20a, we understand that the rate of H abstraction reaction from the carbon center that is connected to the OH group is increased by pressure. For the Cα center of n-butanol in reaction with NH, k(T,p) at 600 K in the pressures of 10^–2^, 10^–1^,1, 10, and 10^2^ bar is 1.41E−17, 6.92E−17, 2.30E−16, 6.61E−16, and 5.81E−16 cm^3^ molecule^-1^ s^-1^, respectively. We reasoned this increase by the ratio *k*_*∞*_*/k*_*0*_ for the above reactions. Here, we also continue the same argument. Our estimation for the *k*_*∞*_*/k*_*0*_ ratio of the *P2*(*bu*) formation path at 600, 1500, and 3000 K are 3.94E + 05, 1.30E + 08, and 8.02E + 10, respectively. As Fig. 20c shows, we can say an overall remark that the ratio *k*_*∞*_*/k*_*0*_ increases by carbon chain length increasing at high temperatures. This statement can explicitly be shown by the (*k*_*∞*_*/k*_*0*_)_bu_*/*(*k*_*∞*_*/k*_*0*_)_pr_, (*k*_*∞*_*/k*_*0*_)_bu_*/*(*k*_*∞*_*/k*_*0*_)_e,_ and (*k*_*∞*_*/k*_*0*_)_bu_*/*(*k*_*∞*_*/k*_*0*_)_m_ ratios, which are 2.96, 1.13, and 1.25 at 600 K and 2.52, 1.17, and 1.80 at 1500 K and 9.94E + 01, 1.11E + 01, and 2.90 at 3000 K, respectively. Another evidence for a rate increase is the reduced rate constant. The reduced rate constant for *P2*(*bu*) at 600 K, *k*(*600 K,p*)*/k*(*600 K,1 bar*), in the pressures of 10^–2^, 10^–1^, 10, and 10^2^ bar are 6.12E−02, 3.01E−01, 2.01, and 2.53, respectively. Also, the same values for *k*(*1500 K,p*)*/k*(*1500 K,1 bar*) (and *k*(*3000 K,p*)*/k*(*3000 K,1 bar*)) are 2.32E−02 (1.00E−02), 1.60E−01 (1.00E−01), 5.04 (1.00E + 01), and 1.73E + 01(1.00E + 02), respectively.

It can be said that temperature increase at high pressures is slightly not suitable for n-butanol by considering the ratios *k*(*T,p*)_bu_*/k*(*T,p*)_m_, *k*(*T,p*)_bu_*/k*(*T,p*)_e_, and *k*(*T,p*)_bu_*/k*(*T,p*)_pr_ (see Fig. 20a). The first ratio has values of 4.71 (at 600 K and 0.01 bar), 5.89 (at 600 K and 0.1 bar) 6.84 (at 600 K and 1 bar), 7.62 (at 600 K and 10 bar), and 7.94 (at 600 K and 100 bar). At 1500 and 3000 K this ratio has values of 1.54 and 3.04E−03 (at 0.01 bar), 1.69 and 3.58E−03 (at 0.1 bar), 1.89 and 4.67E−03 (at 1 bar), 1.81 and 7.54E−03 (at 10 bar), and 1.60 and 1.71E−02 (at 100 bar), respectively. The second and third ratios at 600 K find the values of 1.41 and 8.66E−01, 1.50 and 9.42E−01, 1.44 and 9.98E−01, 1.31, and 1.05, and 1.21 and 1.08, respectively, at 10^–2^, 10^–1^, 10, and 10^2^ bar. For the *k*(*1500,p*)_bu_*/k*(*1500,p*)_e_ and *k*(*1500,p*)_bu_*/k*(*1500,p*)_pr_ ratios the obtained values at the same pressures are 1.17 and 8.01E−01, 1.17, and 8.31E−01, 1.15 and 8.68E−01, 1.19, and 935E−01, and 1.08 and 9.61E−01, respectively. Also, when the temperature increases to 3000 K, these ratios get the values 1.51E−2 and 7.30E−2 at all pressures.

### Some atmospheric events

In this section, the gas phase degradation of primary alcohols is investigated according to the real conditions of our ambient. The considering condition is the relative humidity since it plays an important role in the atmosphere. As is well proved, the main source of hydroxyl radicals in the air is water molecules. The reaction of water molecules with the produced atomic oxygen from the photolysis of ozone lead to the formation of OH radicals as follows^123,124^

57 $${\text{O}}_{{3}} + hv \to {\text{O}}_{{2}} + {\text{ O,}}$$

58 $${\text{O}} + {\text{ H}}_{{2}} {\text{O}} \to {\text{2OH}}{.}$$

Also, another way for the production of hydroxyl radicals is the direct photolysis of water molecules^125–127^.

59 $${\text{H}}_{{2}} {\text{O}} + hv \to {\text{OH }} + {\text{ H}}{.}$$

The formed atomic hydrogen from  Eq. 59 and from other atmospheric reactions can react with ozone and generate OH radicals as well^128,129^.

60 $${\text{H}} + {\text{ O}}_{{3}} \to {\text{OH }} + {\text{ O}}_{{2}} .$$

It should be noted that all the abovementioned reactions can be regarded as domain sources of OH production in the air^123–125^. So, the concentration of hydroxyl radical will change by relative humidity. For relative humidity, three states are feasible, including high, moderate, and low relative humidity. In high relative humidity, the concentration of OH radical is higher than other radical species near ~ 10^6^ cm^3^ molecule^−1^^51,52^. Also, theoretical^10,53–58^ and experimental^11,59–68^ types of research show that the rate of methanol + OH reaction has an order of ~ 10^–12^ cm^3^ molecule^−1^ s^−1^ at 298 K. This order for ethanol^63–70,130,131^, n-propanol^37,64,65,71,132,133^, and n-butanol^42,48,65,134–141^ plus OH reactions is almost the similar to the methanol + OH reaction. Because these rates are usually higher than the rates of other reactions of alcohols with atmospheric radicals and also due to high concentration of OH radicals, it can be concluded that the action of other radicals in comparison with OH radicals is negligible in the degradation of alcohols in an ambient having high relative humidity. But, in the relative humidity in which the concentration of OH radicals is the same as other active oxidants such as O, F, Cl, and NH species, temperature and pressure have a crucial role in the activity of atmospheric species. Thus, by considering the relative humidity of an ambient and its temperatures and pressure, and also the reactivity of atmospheric oxidants, it can refer to the following cases.

- In low temperatures and pressures, the activity of OH radicals is almost higher than many oxidant species (this statement is based on the reported rate constants). So, again the main sink of alcohol removal from the atmosphere is OH radicals.
- As is well known, for many atmospheric reactions by increasing in temperature and pressure, the rate of many atmospheric reactions is increased (the inverse behavior is observed for barrierless reactions). So, in high pressures and temperatures, the rate of some reacting species such as O^142^, F^143,144^, Cl^145–147^, and NH is the same as OH radicals or higher. Therefore, there is a competition between F, Cl, and ^3^NH radicals for the elimination of atmospheric pollutants (like alcohols).
- In low relative humidity in which the concentration of OH radicals is negligible, other reactive species play a vital role based on their activity and concentration in the degradation of atmospheric species (like alcohols).

It has been proved that NH_3_ molecules have a good concentration in the atmosphere^148^. We^108^ and others^149–154^ proved that the principal reaction of NH_3_ is as follows:

61 $${\text{NH}}_{{3}} + {\text{ OH}} \to {\text{NH}}_{{2}} + {\text{ H}}_{{2}} {\text{O}}{.}$$

The produced amidogen (NH_2_) molecules can undergo the following reaction^108,155^

62 $${\text{NH}}_{{2}} + {\text{OH}} \to {\text{NH }} + {\text{H}}_{{2}} {\text{O}}{.}$$

Also, amidogen radical and atomic nitrogen through the following reaction yields imidogen molecules^156^

63 $${\text{NH}}_{{2}} + {\text{ N}} \to {\text{NH }} + {\text{ NH}}{.}$$

On the other hand, the photodissociation of NH_3_^157–159^ and HN_3_^160^ will help the NH generation in the atmosphere by the following dissociation reactions.

64 $${\text{NH}}_{{3}} + hv \to {\text{NH}}_{{2}} + {\text{ H,}}$$

65 $${\text{NH}}_{{2}} + hv \to {\text{NH }} + {\text{ H,}}$$

66 $${\text{NH}}_{{3}} + hv \to {\text{NH }} + {\text{ H}}_{{2}} ,$$

67 $${\text{HN}}_{{3}} + hv \to {\text{NH }} + {\text{ N}}_{{2}} .$$

Therefore, based on Eqs. (62–67), we understand that in high and low relative humidity, the concentration of NH radicals may be appreciable. But, as aforementioned the concentration of OH radicals in high relative humidity is very high. So, the concentration of NH is very low than hydroxyl radicals. On the other hand, our computed rate constants demonstrate that roughly at a temperature above 400 K, the reactions of NH with alcohols will take place in the atmosphere. This temperature is supplied by sun radiation mainly. Therefore, it can be understood that imidogen in dry air will act as an excellent scavenger to have a clean atmosphere from pollutants such as methanol, ethanol, n-propanol, and n-butanol.

### Summary

In this paper, atmospheric degradation of primary alcohols as a main atmospheric challenge was discussed because air quality directly depends on the concentration alcohols. To prove the subject, we examined the atmospheric relevance of reactions of linear organic alcohols with imidogen by using two different theoretical approaches to have comprehensive information about the chemistry of those reactions. Accordingly, two well-behaved theoretical methods, CBS-QB3 and M06-2X, based on trusted QM formalisms were used to prove the studying subject. By the used methods, all the most probable channels for removing the selected alcohols were designed and so the PES of all reactions were established. Also, the necessary energetic parameters such as thermodynamic variables and relative energies were computed for involved stationary points. Through the accomplished results, it was proven that the energy barrier of the H abstraction reaction from the connected carbon to the OH group (C_α_) is lower than in the other centers. And, the products of this channel is more stable than the others. Thus, in atmospheric conditions, the destruction of linear organic alcohols begins through this center. To discover what temperatures and pressures in which the simulated reactions find atmospheric relevance, the temperature- and pressure-dependent rate constants were calculated for all channels of each reaction. Our computed rate constants by both QM methods revealed that the reactions of target alcohols with imidogen are meaningful at moderate temperatures and pressures. In addition, compared to methanol and ethanol, the results uncovered that the rate of long chain length alcohols and NH reactions at high pressures and moderate temperatures is high but at high temperatures, a similar rate is predicted for all alcohols. In summary, the executed procedure in this study made clear that the atmospheric relevance of the atmospheric alcohols is possible at moderate temperatures and pressures.

### Computational details

For four reactions under consideration here, full geometry optimization together with harmonic vibrational frequency computations are carried out for all involved molecules such as reactants (Rs), products (Ps), and transition states (TSs) by the validated meta hybrid density functional method, M06-2X^161,162^. The used basis set for the M06-2X method is the Pople double zeta 6–31 + G(d,p) type^163^. Our recent investigations demonstrated that the structures involved in the reaction pathways and also the kinetic analysis of the H abstraction mechanism in the gas phase reactions can obtain with high accuracy by employing the M06-2X method^164,165^. After predicting reliable structures, the most popular composite method, CBS-QB3^166^, is used to calculate more precise energetic parameters. To prove the reliability of the mentioned methods^83^, the W1BD^167^ method which uses the Brueckner doubles^168,169^ (BD and BD(T)) methods instead of the coupled cluster^170^ (CCSD and CCSD(T)) methods in the Weizmann-1 theory is applied to predict the exact energies of the stationary points. It is better to say that the W1BD is utilized only in methanol and ethanol plus NH reactions due to its high computational cost. The results revealed that the energy barriers calculated by the W1BD and CBS-QB3 methods are in satisfactory agreement with each other.

According to the obtained harmonic vibrational frequencies, we find the lack of imaginary frequency in the final structures verifying that the structure is true minima. Also, the presence of only one negative eigenvalue in the Hessian (force constant) matrices confirms that the structure is a transition state (TS) one. To find the possible reaction pathways, the connectivity of saddle points to both respective per-reactive and post-reactive complexes is considered by the intrinsic reaction coordinate (IRC) calculations^171–173^. For IRC evaluation, the M06-2X/6–31 + G(d,p) level of theory is selected. For completing the IRC path to reach the proper minima, we optimize the geometries of the first point on the left side and the last point on the right side of the saddle point. All electronic structure calculations for extracting the geometries and energies of the components of the reaction are done by the Gaussian 09 package^174^.

The well-known theory for the topological analysis of the wave function, AIM theory, is utilized to specify the main bonding features of all structures^175^. Particularly, the electronic charge density $\rho$ (in e bohr^-3^) and its Laplacian at critical points $\nabla^{2} \rho$ (in e bohr^-5^) analysis is used to determine the nature of the newly formed bonds. To this end, the first-order density matrix (Hessian Matrix) is computed by AIM 2000 software at the M06-2X/6–31 + G(d,p) level.

### Temperature and pressure dependent rate constants calculations

The kinetics of the selected alcohols in reaction with NH is determined at different temperatures and pressures. In addition, the fitted rate expressions of the high-pressure-limit rate constants are extracted to achieve the Arrhenius parameters of all channels at various temperatures. Both abovementioned QM methods are used for calculations of rate constants over the temperature range of 300–3000 K. For pressure effect, the CBS-QBS method is utilized in the range of 1.00E−07 to 1.00E + 04 bar. The transition state theory^176^ (TST) is carried out to compute the high-pressure limit rate constants of entire elementary reactions. Also, since all pathways of the above-discussed reactions involve the conventional hydrogen atom transfer, tunneling correction is important. A rather simple proposed method for computing the quantum tunneling effect is the introduced formula by Carl Eckart^177,178^. This approach is a special case of the tunneling method that is named zero-curvature tunneling (ZCT)^92,179^. The ZCT method is also referred to as ZCT-0. The ZCT-0 to establish the ground-state potential energy curve uses the Eckart function. As TST calculations predict the rate of reactions by a simple algorithm, the Eckart and ZCT approaches by using a similar manner forecast the tunneling correction. In other words, these approaches just need information on the stationary points such as reactants, transition state, and products along the minimum energy path (MEP). The Eckart method uses just the energies of the mentioned stationary points but the ZCT method in addition to the energy needs to the other parameters such as geometries, gradients, and Hessians. By these statements, one leads to conclude that both Eckart and ZCT models are the most logical choices for an acceptable predicting tunneling effect value accompanied by the TST calculations. It shall be emphasized that the produced values by these approaches are significantly more precise than the same value predicted by the Wigner method, without no extra computational attempts, when the TST theory is employed for yielding more accurate rate constant. Despite these statements, these results demonstrated that the Eckart predicted values are oftentimes higher than the values obtained by the ZCT approaches at low temperatures. And it has also been proved that tunneling is more important below a temperature of 200 K^180^. This means that the error of the Eckart method may be seen under 200 K. But, the forecasted values by the Eckart method at temperatures above room temperature are very near to the value obtained by the suggested more correct approximations ^177,181,182^, including the small curvature tunneling, SCT. So, the ZCT values compared to the same SCT values are lower to some extent^181^. It seems that the origin of the error in the Eckart approach is related to the corner-cutting effects that are not included in it. However, this point is clearly noticeable that the Eckart function is often too narrow, which can compensate for the mentioned error^177^.

As we know, atmospheric reactions have different rates by variation of altitude. The change in reaction rate is mainly sensible in lower altitudes, 0.00 to 12.00 km, where for the 1.00 km change in height, about 6.49 K variation is seen in the ambient temperature. Thus, important factors such as thermochemical properties, kinetics, and so reactivity should be a function of both temperature and pressure. This conclusion leads us to investigate the influence of pressure on the reactions of linear organic alcohols to have more precise results by defining reliable conditions. Therefore, in addition to temperature, the effect of atmospheric pressure on the rate constant of the main reaction pathway of each alcohol is argued based on the chemical activation mechanism. To compute the temperature and pressure dependent rate constant, k(T,p), the strong collision approach by considering the atmospheric nitrogen (a species with high concentration) as the bath gas for producing energized molecules is chosen. The Rice–Ramsperger–Kassel–Marcus (RRKM) theory^183^ by using the Ssumes program^184^ is applied to predict the behavior of rate constants in the falloff regime. The used Lennard–Jones parameters for imidogen, methanol, ethanol, n-propanol, and n-butanol are 2.650 Å and 80.000 K, 3.626 Å and 481.800 K, 4.530 Å and 362.600 K, 4.55 Å, and 576.680 K, and 4.688 Å and 531.3000 K, respectively^185^. The energetic parameters of the CBS-QB3 method and the Lennard–Jones parameters are used to generate the input of RRKM calculations. It should be noted that k(T,p) is calculated in the pressures of 1.00E−7 to 1.00E + 4 bar over the 300–3000 K temperature range. For H abstraction channels, k(T,p) graphs of methanol, ethanol, n-propanol, and n-butanol reactions with NH are sketched in Figures, 5,10, 15, and 20, respectively. Also, k(T,p) values of the mentioned reactions are listed in Supplementary Table S17–S20, respectively. The simple form of the pressure-dependent rate constant is as follows^164,183^:

68 $$k(T,P) = \frac{{k_{\infty } }}{{1 + \frac{{k_{\infty } }}{{k_{0} }}}}.$$

As we can see from the simple form of the equation defined for the pressure-dependent rate constant, the ratio of *k*_*∞*_*/k*_*0*_ is a key term to forecast the falloff regime behavior of the rate constant. Also, defining the reduced rate constant as the ratio of *k*(*T,p*) into *k*(*T,1 bar*) gives a piece of clear information about the effect of pressure on the reaction of each channel. Therefore, through both ratios of *k*_*∞*_*/k*_*0*_ and *k*(*T,p*)/*k*(*T,1 bar*), we consider the pressure effect on the gas-phase reactions of methanol, ethanol, *n*-propanol, and *n*-butanol with NH.

A standard software, Gpop program^186^, is implemented for computing all high-pressure limit rate constants.

## Supplementary Information

Supplementary Information.

## Data Availability

All data generated or analysed during this study are included in this published article [and its supplementary information files].
