# Supplementary material for: The atmospheric relevance of primary alcohols and imidogen reactions
Source: Sci Rep. 2023 Jun 5;13:9150. doi: 10.1038/s41598-023-35473-1 (PMC10241922; doi:10.1038/s41598-023-35473-1)
Supplement: Supplementary file 1 — Supplementary Information. [file 41598_2023_35473_MOESM1_ESM.docx]

**The atmospheric relevance of primary alcohols and imidogen reactions**

Hamed Douroudgari^*^, Hadi Zarepour, Morteza Vahedpour^*^, Mahdi Jaberi, Mahdi Zarepour

*Department of Chemistry, University of Zanjan, PO Box 38791-45371, Zanjan, Iran*

E-mail:  [douroudgari@znu.ac.ir](mailto:%20douroudgari@znu.ac.ir)

E-mail:  [vahed@znu.ac.ir](mailto:%20vahed@znu.ac.ir)

**Figures**

**Figure S1**. Structures of all stationary points including bond lengths (in angstrom) in the C_2_H_5_OH + NH reaction calculated at the M06-2X method. . ………………………………………………………52

**Figure S2**. Potential energy surface of the C_2_H_5_OH + NH reaction at the triplet ground state computed by the CBS-QB3 level. . ……………………………………………………..………………………53

**Figure S3**. Structures of all stationary points including bond lengths (in angstrom) in the n-C_3_H_7_OH + NH reaction calculated at the M06-2X method. . ………………………………………………………62

**Figure S4**. Potential energy surface of the n-propanol-a + NH reaction at the triplet ground state computed by the CBS-QB3 level. . ………………………………………………………….………………63

**Figure S5**. Structures of all stationary points including bond lengths (in angstrom) in the n-C_3_H_7_OH + NH reaction calculated at the M06-2X method. . ………………………………………...………………70

**Figure S6**. Potential energy surface of the n-propanol-b reaction at the triplet ground state computed by the CBS-QB3 level. . ………………………………………………………………71

**Figure S7**. Structures of all stationary points including bond lengths (in angstrom) in the n-C_3_H_7_OH + NH reaction calculated at the M06-2X method. . ……………………………………...…………………78

**Figure S8**. Potential energy surface of the n-propanol-c reaction at the triplet ground state computed by the CBS-QB3 level. . ………………………………………………………………79

**Figure S9**. Structures of all stationary points including bond lengths (in Angstrom) in the n-C_4_H_9_OH + NH reaction calculated at the M06-2X/6-31+G(d,p) level of theory. . ……..………86

**Figure S10**. Potential energy surface of the n-butanol-a + NH reaction at the triplet ground state computed by the CBS-QB3 level. . ……………………………………………………..………87

**Figure S11**. Structures of all stationary points including bond lengths (in angstrom) in the n-butanol-b + NH reaction calculated at the M06-2X/6-31+G(d,p) level of theory. . ……………94

**Figure S12**. Potential energy surface of the n-butanol-b + NH reaction at the triplet ground state computed by the CBS-QB3 level. . ……………………………………………………….……95

**Figure S13**. Structures of all stationary points including bond lengths (in angstrom) in the n-butanol-c + NH reaction calculated at the M06-2X/6-31+G(d,p) level of theory. . ……….…102

**Figure S14**. Potential energy surface of the n-butanol-c + NH reaction at the triplet ground state computed by the CBS-QB3 level. . ……………………………………………………………103

**Figure S15**. Structures of all stationary points including bond lengths (in angstrom) in the n-butanol-d + NH reaction calculated at the M06-2X/6-31+G(d,p) level of theory……………….111

**Figure S16**. Potential energy surface of the n-butanol-d + NH reaction at the triplet ground state computed by the CBS-QB3 level. . ……………………………………………………….……112

**Figure S17**. Structures of all stationary points including bond lengths (in angstrom) in the n-butanol-e + NH reaction calculated at the M06-2X/6-31+G(d,p) level of theory………………..120

**Figure S18**. Potential energy surface of the n-butanol-e + NH reaction at the triplet ground state computed by the CBS-QB3 level. ……………………………………………………………121

**Tables**

**Table S1**. Absolute energies and thermodynamic parameters for stationary points of the CH_3_OH + NH reaction. (All energies are in Hartree and entropies are in cal mol^-1^ K^-1^)………………..…9

**Table S1b**. Absolute energies and thermodynamic parameters for stationary points of the CH_3_OH + NH reaction computed at the W1BD level. ……………………………….………………..…10

**Table S2**. The CCSD(T) absolute energies (in Hartree) and T1 diagnostic values for stationary points of the methanol + NH reaction calculated at the CCSD(T)/6-31+g(d**´**)//M06-2X level of theory. ……………………………….…………………………………………………….…..…11

**Table 3a**. Absolute energies and thermodynamic parameters for stationary points of the C_2_H_5_OH + NH reaction. (All energies are in Hartree and entropies are in cal mol^-1^ K^-1^)…………….…….12

**Table S3b**. Absolute energies and thermodynamic parameters for stationary points of the C_2_H_5_OH + NH reaction computed at the W1BD level. ……………………….……………...…13

**Table S4**. The CCSD(T) absolute energies (in Hartree) and T1 diagnostic values for stationary points of the ethanol + NH reaction calculated at the CCSD(T)/6-31+g(d**´**)//M06-2X level of theory. ……………………………….………………………………………………………..…14

**Table S5**. Absolute energies and thermodynamic parameters for stationary points of the n-C_3_H_7_OH + NH reaction. (All energies are in Hartree and entropies are in cal mol^-1^ K^-1^)……………..….15

**Table S6**. The CCSD(T) absolute energies (in Hartree) and T1 diagnostic values for stationary points of the n-propanol + NH reaction calculated at the CCSD(T)/6-31+g(d**´**)//M06-2X level of theory. ……………………………….………………………………………………………..…16

**Table S7**. Absolute energies and thermodynamic parameters for stationary points of the n-C_4_H_9_OH + NH reaction. (All energies are in Hartree and entropies are in cal mol^-1^ K^-1^)……….……...…17

**Table S8**. The CCSD(T) absolute energies (in Hartree) and T1 diagnostic values for stationary points of the n-butanol + NH reaction calculated at the CCSD(T)/6-31+g(d**´**)//M06-2X level of theory. ……………………………….…………………………………………………..…..…18

**Table S9.** Calculated equilibrium constants for pre-reactive complexes in the CH_3_OH + NH reaction computed at two computational levels……………………………………………...…19

**Table S10.** Equilibrium constants for pre-reactive complexes of ethanol plus imidogen reactions computed at two computational levels……………………………………………………………20

**Table S11.** Calculated equilibrium constants for pre-reactive complexes in the n-C_3_H_7_OH + NH reaction computed at two computational levels…………………………………………………21

**Table S12.** Calculated equilibrium constants for pre-reactive complexes in the n-C_4_H_9_OH + NH reaction computed at two computational levels…………………………………………………22

**Table S13**. Rate constants (cm^3^ molecule^-1^ s^-1^) of all channels in the methanol plus NH reaction…………………………………………………………………………………………...23

**Table S14**. Rate constants (cm^3^ molecule^-1^ s^-1^) of all channels in the ethanol plus NH reaction…………………………………………………………………………………………...24

**Table S15**. Rate constants (cm^3^ molecule^-1^ s^-1^) of all channels in the n-propanol plus NH reaction. …………………………………….……………………………………………………………...25

**Table S16**. Rate constants (cm^3^ molecule^-1^ s^-1^) of all channels in the n-butanol plus NH reaction. ……………………….…………………….………………………………………………...…...26

**Table S17**. Calculated pressure-dependent rate constants for the H atom transfer of the methyl group in the methanol plus NH reaction at the CBS-QB3 method. ……………...……..………...27

**Table S18**. Calculated pressure-dependent rate constants for the H atom transfer of the Cα center in the ethanol plus NH reaction at the CBS-QB3 method. . ……………………………..……...30

**Table S19**. Calculated pressure-dependent rate constants for the H atom transfer of the Cα center in the n-propanol plus NH reaction at the CBS-QB3 method. . ………………………………...33

**Table S20**. Calculated pressure-dependent rate constants for the H atom transfer of the Cα center in the n-butanol plus NH reaction at the CBS-QB3 method. . ……………………….…..……...34

**Table S21.** Topological parameters such as ring critical point (RCP) and bond critical points and (in atomic unit) for all species of the CH_3_OH + NH reaction calculated at the M06-2X method….. ………………………………………………………………………………..……………..…...39

**Table S22.** Topological parameters such as ring critical point (RCP) and line critical points (LCP) (in atomic unit) for all species of the C_2_H_5_OH+ NH reaction calculated at the M06-2X method. . . …………………………………………………………………………….….………………..40

**Table S23.** Topological parameters such as ring critical point (RCP) and line critical points (LCP) for all species of the n-C_3_H_7_OH+ NH reaction calculated at the M06-2X method…………..…………………………………………………………….…………..……...42

**Table S24.** Topological parameters such as line critical point (LCP) and ring critical point (RCP) and line critical points (LCP) for all species of the n-C_4_H_9_OH + NH reaction calculated at the M06-2X method…………………………………………………………………………………..….46

**Table S25**. The computed relative energies for the stationary points of the C_2_H_5_OH + NH reaction. (Unit of all numbers is kcal mol^-1^) . ………………………………………………………………………54

**Table S26**. Thermodynamic parameters for stationary points of the C_2_H_5_OH + NH reaction. (Unit of all numbers is kcal mol^-1^) . ……………………………………………………….……………55

**Table S27**. Absolute energies and thermodynamic parameters for stationary points of the C_2_H_5_OH + NH reaction computed at the W1BD level. . …………………………………………………56

**Table S28**. Absolute energies and thermodynamic parameters for stationary points of the C_2_H_5_OH + NH reaction computed at the CBS-QB3 level. . ……………………………………….………57

**Table S29**. Absolute energies and thermodynamic parameters for stationary points of the C_2_H_5_OH + NH reaction calculated at the M06-2X method. . ……………………………………..………58

**Table S30**. The CCSD(T) absolute energies (in Hartree) and T1 diagnostic values for stationary points of the ethanol + NH reaction calculated at the CCSD(T)/6-31+g(d**´**)//M06-2X level of theory. . ……………………………………………………………………………….…………59

**Table S31**. Relative energies and thermodynamic parameters for stationary points of the n-propanol-a + NH. (Unit of all numbers is kcal mol^-1^) . …………………………………..………65

**Table S32**. Relative energies and thermodynamic parameters for stationary points of the n-propanol-a + NH calculated at the CBS-QB3 method. . …………………………….……………65

**Table S33**. Relative energies and thermodynamic parameters for stationary points of the n-propanol-a + NH calculated at the M06-2X method. . ……………………………………..……66

**Table S34**. The CCSD(T) absolute energies (in Hartree) and T1 diagnostic values for stationary points of the n-propanol-a + NH reaction calculated at the CCSD(T)/6-31+g(d**´**)//M06-2X level of theory. . ……………………………………………………………………………………..……67

**Table S35**. Relative energies and thermodynamic parameters for stationary points of the n-propanol-b + NH. (Unit of all numbers is kcal mol^-1^) . ……………………………….…………72

**Table S36**. Relative energies and thermodynamic parameters for stationary points of the n-propanol-b + NH calculated at the CBS-QB3 method. . …………………………………………73

**Table S37**. Relative energies and thermodynamic parameters for stationary points of the n-propanol-b + NH calculated at the M06-2X method. . ………………………………..…………74

**Table S38**. The CCSD(T) absolute energies (in Hartree) and T1 diagnostic values for stationary points of the n-propanol-b + NH reaction calculated at the CCSD(T)/6-31+g(d**´**)//M06-2X level of theory. . ……………………………………………………………………………….…………75

**Table S39**. Relative energies and thermodynamic parameters for stationary points of the n-propanol-c + NH. (Unit of all numbers is kcal mol^-1^) . …………………………………………80

**Table S40**. Relative energies and thermodynamic parameters for stationary points of the n-propanol-c + NH calculated at the CBS-QB3 method. . ………………..………………………81

**Table S41**. Relative energies and thermodynamic parameters for stationary points of the n-propanol-c + NH calculated at the M06-2X method. . …………………………………………82

**Table S42**. The CCSD(T) absolute energies (in Hartree) and T1 diagnostic values for stationary points of the n-propanol-c + NH reaction calculated at the CCSD(T)/6-31+g(d**´**)//M06-2X level of theory………….……………………………………………………. ………………………83

**Table S43**. Relative energies and thermodynamic parameters for stationary points of the n-butanol-a + NH reaction. (Unit of all numbers is kcal mol^-1^) . …………………………………88

**Table S44**. Relative energies and thermodynamic parameters for stationary points of the n-butanol-a + NH reaction calculated at the CBS-QB3 level. . ……………………………………89

**Table S45**. Relative energies and thermodynamic parameters for stationary points of the n-butanol-a + NH reaction calculated at the M06-2X method. . ………………………..…………90

**Table S46**. The CCSD(T) absolute energies (in Hartree) and T1 diagnostic values for stationary points of the n-butanol-a + NH reaction calculated at the CCSD(T)/6-31+g(d**´**)//M06-2X level of theory. . …………………………………………………………………………………………91

**Table S47**. Relative energies and thermodynamic parameters for stationary points of the n-butanol-b + NH reaction. (Unit of all numbers is kcal mol^-1^) . …………………………………96

**Table S48**. Relative energies and thermodynamic parameters for stationary points of the n-butanol-b + NH reaction calculated at the CBS-QB3 level. ……………………………………97

**Table S49**. Relative energies and thermodynamic parameters for stationary points of the n-butanol-b + NH reaction calculated at the M06-2X method. . …………………………………98

**Table S50**. The CCSD(T) absolute energies (in Hartree) and T1 diagnostic values for stationary points of the n-butanol-b + NH reaction calculated at the CCSD(T)/6-31+g(d**´**)//M06-2X level of theory. . …………………………………………………………………………………………99

**Table S51**. Relative energies and thermodynamic parameters for stationary points of the n-butanol-c + NH reaction. (Unit of all numbers is kcal mol^-1^) . …………………………………104

**Table S52**. Absolute energies and thermodynamic parameters for stationary points of the n-butanol-b + NH reaction calculated at the CBS-QB3 level. . …………………………………105

**Table S53**. Relative energies and thermodynamic parameters for stationary points of the n-butanol-c + NH reaction calculated at the M06-2X method. . ………………………………106

**Table S54**. The CCSD(T) absolute energies (in Hartree) and T1 diagnostic values for stationary points of the n-butanol-c + NH reaction calculated at the CCSD(T)/6-31+g(d**´**)//M06-2X level of theory. . ……………………………………………………………………………..…………107

**Table S55**. Relative energies and thermodynamic parameters for stationary points of the n-butanol-c + NH reaction. (Unit of all numbers is kcal mol^-1^) . …………………………………113

**Table S56**. Absolute energies and thermodynamic parameters for stationary points of the n-butanol-d + NH reaction calculated at the CBS-QB3 level. . …………………………………114

**Table S57**. Relative energies and thermodynamic parameters for stationary points of the n-butanol-d + NH reaction calculated at the M06-2X method. ……………………………………115

**Table S58**. The CCSD(T) absolute energies (in Hartree) and T1 diagnostic values for stationary points of the n-butanol-d + NH reaction calculated at the CCSD(T)/6-31+g(d**´**)//M06-2X level of theory. . ………………………………………………………………………………....………116

**Table S59**. Relative energies and thermodynamic parameters for stationary points of the n-butanol-e + NH reaction. (Unit of all numbers is kcal mol^-1^) . ……………………..…………122

**Table S60**. Absolute energies and thermodynamic parameters for stationary points of the n-butanol-e + NH reaction calculated at the CBS-QB3 level. . …………………………..………123

**Table S61**. Relative energies and thermodynamic parameters for stationary points of the n-butanol-e + NH reaction calculated at the M06-2X method. . ………………………….………124

**Table S62**. The CCSD(T) absolute energies (in Hartree) and T1 diagnostic values for stationary points of the n-butanol-e + NH reaction calculated at the CCSD(T)/6-31+g(d**´**)//M06-2X level of theory. . ………………………………………………………………………….……………125

**Table S63.** Cartesian coordinates (Å) of all optimized species in the CH_3_OH + NH reaction… ………………………………………………………………………………………….……....126

**Table S64.** Cartesian coordinates (Å) of all optimized species in the C_2_H_5_OH + NH reaction... ………………………………………………………………………………………..…...…..130

**Table S65.** Cartesian coordinates (Å) of all optimized species in the n-C_3_H_7_OH+ NH reaction… ……………………………………………………………………………………..….........…..136

**Table S66.** Cartesian coordinates (Å) of all optimized species in the n-C_4_H_9_OH+ NH reaction… ………………………………………………………………………………………….…......146

**Table S1a**. Absolute energies and thermodynamic parameters for stationary points of the CH_3_OH + NH reaction. (All energies are in Hartree and entropies are in cal mol^-1^ K^-1^)

| **Species** | ***E+ZPE(A)*** | ***E(0K)(B)*** | ***E˚(A)*** | ***E˚(B)*** | ***H˚(A)*** | ***H˚(B)*** | ***G˚(A)*** | ***G˚(B)*** | ***S˚(A)*** |
| --- | --- | --- | --- | --- | --- | --- | --- | --- | --- |
| **NH** | -55.1902 | -55.1445 | -55.1879 | -55.1421 | -55.1869 | -55.1412 | -55.2075 | -55.1617 | 43.257 |
| **CH_3_OH** | -115.6195 | -115.5399 | -115.6162 | -115.5366 | -115.6153 | -115.5357 | -115.6422 | -115.5627 | 56.546 |
| **CR1(m)** | -170.8144 | -170.6859 | -170.8084 | -170.6787 | -170.8074 | -170.6769 | -170.8437 | -170.7244 | 76.270 |
| **CR2(m)** | -170.8144 | -170.6878 | -170.8083 | -170.6794 | -170.8074 | -170.6784 | -170.8438 | -170.7164 | 76.674 |
| **TS1(m)** | -170.7866 | -170.6579 | -170.7814 | -170.6814 | -170.7804 | -170.6805 | -170.8152 | -170.7186 | 73.092 |
| **TS2(m)** | -170.7898 | -170.6627 | -170.7847 | -170.6526 | -170.7838 | -170.6517 | -170.8178 | -170.6861 | 71.556 |
| **CP1(m)** | -170.7952 | -170.6684 | -170.7887 | -170.6574 | -170.7877 | -170.6564 | -170.8250 | -170.6912 | 78.345 |
| **CP2(m)** | -170.8121 | -170.6878 | -170.8064 | -170.6617 | -170.8054 | -170.6608 | -170.8407 | -170.6987 | 74.263 |
| **NH_2_** | -55.8293 | -55.7912 | -55.8265 | -55.7884 | -55.8255 | -55.7874 | -55.8476 | -55.8095 | 46.500 |
| **CH_3_O** | -114.9612 | -114.8745 | -114.9689 | -114.8715 | -114.9680 | -114.8705 | -114.9952 | -114.8974 | 57.346 |
| **CH_2_OH** | -114.9723 | -114.8881 | -114.9583 | -114.8848 | -114.9573 | -114.8839 | -114.9842 | -114.9111 | 56.450 |

A and B refer to the M06-2X and CBS-QB3 methods, respectively.

**Table S1b**. Absolute energies and thermodynamic parameters for stationary points of the CH_3_OH + NH reaction computed at the W1BD level.

| **Species** | ***E(0K) (Hartree)*** | ***E˚(Hartree)*** | ***H˚(Hartree))*** | ***G˚(Hartree)*** |
| --- | --- | --- | --- | --- |
| **Methanol** | -115.7324 | -115.7290 | -115.7281 | -115.7552 |
| **NH** | -55.2362 | -55.2338 | -55.2329 | -55.2534 |
| **CR1(m)** | -170.9719 | -170.9651 | -170.9642 | -171.0031 |
| **CR2(m)** | -170.9721 | -170.9655 | -170.9646 | -171.0034 |
| **TS1(m)** | -170.9404 | -170.9351 | -170.9341 | -170.9690 |
| **TS2(m)** | -170.9453 | -170.9399 | -170.9389 | -170.9741 |
| **CP1(m)** | -170.9530 | -170.9461 | -170.9451 | -170.9839 |
| **CP2(m)** | -170.9728 | -170.9659 | -170.9649 | -171.0035 |
| **NH2** | -55.8834 | -55.8806 | -55.8796 | -55.9017 |
| **CH_3_O** | -115.0667 | -115.0637 | -115.0627 | -115.0897 |
| **CH_2_OH** | -115.0811 | -115.0778 | -115.0768 | -115.1041 |

**Table S2**. The CCSD(T) absolute energies (in Hartree) and T1 diagnostic values for stationary points of the methanol + NH reaction calculated at the CCSD(T)/6-31+g(d**´**)//M06-2X level of theory.

| **Species** | ***CCSD(T)*** | ***T1diagnostic*** |
| --- | --- | --- |
| **Methanol** | -115.3909 | 0.0107 |
| **NH** | -55.0832 | 0.0107 |
| **CR1(m)** | -170.4781 | 0.0118 |
| **CR2(m)** | -170.4815 | 0.0108 |
| **TS1(m)** | -170.4392 | 0.0266 |
| **TS2(m)** | -170.4382 | 0.0187 |
| **CP1(m)** | -170.4607 | 0.0174 |
| **CP2(m)** | -170.4715 | 0.0158 |
| **CH_3_O** | -55.7165 | 0.0101 |
| **NH_2_** | -114.7409 | 0.0171 |
| **CH_2_OH** | -114.7386 | 0.0188 |

**Table S3a**. Absolute energies and thermodynamic parameters for stationary points of the C_2_H_5_OH + NH reaction. (All energies are in Hartree and entropies are in cal mol^-1^ K^-1^)

| **Species** | ***E+ZPE(A)*** | ***E(0K)(B)*** | ***E˚(A)*** | ***E˚(B)*** | ***H˚(A)*** | ***H˚(B)*** | ***G˚(A)*** | ***G˚(B)*** | ***S˚(A)*** |
| --- | --- | --- | --- | --- | --- | --- | --- | --- | --- |
| **C_2_H_5_OH** | -154.8913 | -154.7702 | -154.8871 | -154.7658 | -154.8861 | -154.7649 | -154.9167 | -154.7956 | 64.248 |
| **CR1(e)** | -210.0857 | -209.9162 | -210.0796 | -209.9085 | -210.0787 | -209.9076 | -210.1154 | -209.9493 | 77.276 |
| **CR2(e)** | -210.0875 | -209.9182 | -210.0804 | -209.9108 | -210.0795 | -209.9099 | -210.1188 | -209.9514 | 82.813 |
| **CR3(e)** | -210.0871 | -209.9187 | -210.0804 | -209.9121 | -210.0794 | -209.9112 | -210.1178 | -209.9500 | 80.775 |
| **TS1(e)** | -210.0583 | -209.8887 | -210.0522 | -209.8822 | -210.0512 | -209.8813 | -210.0884 | -209.9195 | 78.167 |
| **TS2(e)** | -210.0647 | -209.8967 | -210.0584 | -209.8900 | -210.0574 | -209.8891 | -210.0950 | -209.9275 | 78.998 |
| **TS3(e)** | -210.0575 | -209.8889 | -210.0514 | -209.8826 | -210.0505 | -209.8816 | -210.0872 | -209.9188 | 77.262 |
| **CP1(e)** | -210.0675 | -209.8984 | -210.0600 | -209.8905 | -210.0591 | -209.8896 | -210.0993 | -209.9316 | 84.632 |
| **CP2(e)** | -210.0866 | -209.9203 | -210.0793 | -209.9122 | -210.0784 | -209.9112 | -210.1184 | -209.9537 | 84.227 |
| **CP3(e)** | -210.0699 | -209.9031 | -210.0618 | -209.8947 | -210.0609 | -209.8938 | -210.1019 | -209.9368 | 86.423 |
| **CH_3_CH_2_O** | -154.2335 | -154.1050 | -154.2294 | -154.1005 | -154.2285 | -154.0996 | -154.2594 | -154.1315 | 65.058 |
| **CH_3_CHOH** | -154.2467 | -154.1209 | -154.2424 | -154.1165 | -154.2414 | -154.1155 | -154.2727 | -154.1469 | 65.766 |
| **CH_2_CH_2_OH** | -154.2341 | -154.1086 | -154.2295 | -154.1039 | -154.2286 | -154.1029 | -154.2601 | -154.1349 | 66.376 |

A and B refer to the M06-2X and CBS-QB3 methods, respectively.

**Table S3b**. Absolute energies and thermodynamic parameters for stationary points of the C_2_H_5_OH + NH reaction computed at the W1BD level.

| **Species** | ***E(0K)(Hartree)*** | ***E˚(Hartree)*** | ***H˚(Hartree)*** | ***G˚(Hartree)*** |
| --- | --- | --- | --- | --- |
| **Ethanol** | -155.0318 | -155.0274 | -155.0265 | -155.0572 |
| **CR1(e)** | -210.2711 | -210.2632 | -210.2623 | -210.3049 |
| **CR2(e)** | -210.2717 | -210.2641 | -210.2632 | -210.3052 |
| **CR3(e)** | -210.2720 | -210.2652 | -210.2643 | -210.3036 |
| **TS1(e)** | -210.2403 | -210.2338 | -210.2329 | -210.2715 |
| **TS2(e)** | -210.2479 | -210.2412 | -210.2403 | -210.2790 |
| **TS3(e)** | -210.2409 | -210.2344 | -210.2335 | -210.2712 |
| **CP1(e)** | -210.2522 | -210.2441 | -210.2431 | -210.2861 |
| **CP2(e)** | -210.2741 | -210.2658 | -210.2649 | -210.3077 |
| **CP3(e)** | -210.2575 | -210.2488 | -210.2478 | -210.2924 |
| **CH_3_CH_2_O** | -154.3664 | -154.3619 | -154.3610 | -154.3929 |
| **CH_3_CHOH** | -154.3828 | -154.3783 | -154.3774 | -154.4088 |
| **CH_2_CH_2_OH** | -154.3708 | -154.3660 | -154.3650 | -154.3973 |

**Table S4**. The CCSD(T) absolute energies (in Hartree) and T1 diagnostic values for stationary points of the ethanol + NH reaction calculated at the CCSD(T)/6-31+g(d**´**)//M06-2X level of theory.

| **Species** | ***CCSD(T)*** | ***T1diagnostic*** |
| --- | --- | --- |
| **CH_3_CH_2_OH** | -154.5806 | 0.0106 |
| **CR1(e)** | -209.6678 | 0.0116 |
| **CR2(e)** | -209.6712 | 0.0108 |
| **CR3(e)** | -209.6714 | 0.0107 |
| **TS1(e)** | -209.6290 | 0.0246 |
| **TS2(e)** | -209.6314 | 0.0173 |
| **TS3(e)** | -209.6238 | 0.0157 |
| **CP1(e)** | -209.6503 | 0.0179 |
| **CP2(e)** | -209.6631 | 0.0149 |
| **CP3(e)** | -209.6434 | 0.0121 |
| **CH_3_CH_2_O** | -153.9281 | 0.0178 |
| **CH_3_CHOH** | -153.9329 | 0.0154 |
| **CH_2_CH_2_OH** | -153.9197 | 0.0124 |

**Table S5**. Absolute energies and thermodynamic parameters for stationary points of the n-C_3_H_7_OH + NH reaction. (All energies are in Hartree and entropies are in cal mol^-1^ K^-1^)

| **Species** | ***E+ZPE(A)*** | ***E(0K)(B)*** | ***E˚(A)*** | ***E˚(B)*** | ***H˚(A)*** | ***H˚(B)*** | ***G˚(A)*** | ***G˚(B)*** | ***S˚(A)*** |
| --- | --- | --- | --- | --- | --- | --- | --- | --- | --- |
| **n-C_3_H_7_OH** | -194.1580 | -193.9952 | -194.1525 | -193.9896 | -194.1516 | -193.9887 | -194.1858 | -194.0229 | 72.064 |
| **CR1(pr)** | -249.3529 | -249.1435 | -249.3446 | -249.1349 | -249.3437 | -249.1339 | -249.3859 | -249.1790 | 88.947 |
| **CR2(pr)** | -249.3539 | -249.1400 | -249.3458 | -249.1303 | -249.3448 | -249.1293 | -249.3872 | -249.1762 | 89.062 |
| **CR3(pr)** | -249.3543 | -249.1436 | -249.3460 | -249.1349 | -249.3451 | -249.1340 | -249.3877 | -249.1793 | 89.678 |
| **CR4(pr)** | -249.3503 | -249.1411 | -249.3415 | -249.1315 | -249.3405 | -249.1305 | -249.3850 | -249.1802 | 93.611 |
| **TS1(pr)** | -249.3258 | -249.1144 | -249.3183 | -249.1066 | -249.3173 | -249.1057 | -249.3585 | -249.1479 | 86.627 |
| **TS2(pr)** | -249.3319 | -249.1225 | -249.3244 | -249.1145 | -249.3235 | -249.1136 | -249.3640 | -249.1556 | 85.227 |
| **TS3(pr)** | -249.3291 | -249.1189 | -249.3211 | -249.1111 | -249.3202 | -249.1101 | -249.3620 | -249.1512 | 87.938 |
| **TS4(pr)** | -249.3224 | -249.1137 | -249.3147 | -249.1056 | -249.3137 | -249.1047 | -249.3551 | -249.1056 | 86.979 |
| **CP1(pr)** | -249.3352 | -249.1242 | -249.3270 | -249.1149 | -249.3260 | -249.1140 | -249.3687 | -249.1598 | 89.780 |
| **CP2(pr)** | -249.3471 | -249.1400 | -249.3380 | -249.1303 | -249.3370 | -249.1293 | -249.3813 | -249.1762 | 93.265 |
| **CP3(pr)** | -249.3414 | -249.1336 | -249.3319 | -249.1237 | -249.3310 | -249.1227 | -249.3766 | -249.1705 | 96.067 |
| **CP4(pr)** | -249.3342 | -249.1273 | -249.3252 | -249.1171 | -249.3243 | -249.1161 | -249.3690 | -249.1646 | 94.012 |
| **CH_3_CH_2_CH_2_O** | -193.5003 | -193.3303 | -193.4950 | -193.3247 | -193.4940 | -193.3237 | -193.5285 | -193.3589 | 72.528 |
| **CH_3_CH_2_CHOH** | -193.5127 | -193.3458 | -193.5072 | -193.3401 | -193.5063 | -193.3392 | -193.5410 | -193.3743 | 73.107 |
| **CH_3_CHCH_2_OH** | -193.5059 | -193.3387 | -193.4998 | -193.3325 | -193.4988 | -193.3316 | -193.5353 | -193.3682 | 76.739 |
| **CH_2_CH_2_CH_2_OH** | -193.5029 | -193.3356 | -193.4968 | -193.3303 | -193.4959 | -193.3294 | -193.5320 | -193.3636 | 75.929 |

A and B refer to the M06-2X and CBS-QB3 methods, respectively.

**Table S6**. The CCSD(T) absolute energies (in Hartree) and T1 diagnostic values for stationary points of the n-propanol + NH reaction calculated at the CCSD(T)/6-31+g(d**´**)//M06-2X level of theory.

| **Species** | ***CCSD(T)*** | ***T1 diagnostic*** |
| --- | --- | --- |
| **Propanol** | -193.7650 | 0.0105 |
| **CR1(pr)** | -248.8558 | 0.0106 |
| **CR2(pr)** | -248.8557 | 0.0105 |
| **CR3(pr)** | -248.8557 | 0.0105 |
| **CR4(pr)** | -248.8503 | 0.0105 |
| **TS1(pr)** | -248.8139 | 0.0227 |
| **TS2(pr)** | -248.8163 | 0.0162 |
| **TS3(pr)** | -248.8129 | 0.0148 |
| **TS4(pr)** | -248.8066 | 0.0145 |
| **CP1(pr)** | -248.8350 | 0.0164 |
| **CP2(pr)** | -248.8399 | 0.0136 |
| **CP3(pr)** | -248.8327 | 0.0123 |
| **CP4(pr)** | -248.8257 | 0.0118 |
| **CH_3_CH_2_CH_2_O** | -193.1126 | 0.0161 |
| **CH_3_CH_2_CHOH** | -193.1169 | 0.0144 |
| **CH_3_CHCH_2_OH** | -193.1085 | 0.0124 |
| **CH_2_CH_2_CH_2_OH** | -193.1062 | 0.0119 |

**Table S7**. Absolute energies and thermodynamic parameters for stationary points of the n-C_4_H_9_OH + NH reaction. (All energies are in Hartree and entropies are in cal mol^-1^ K^-1^)

| **Species** | ***E+ZPE(A)*** | ***E(0K)(B)*** | ***E˚(A)*** | ***E˚(B)*** | ***H˚(A)*** | ***H˚(B)*** | ***G˚(A)*** | ***G˚(B)*** | ***S˚(A)*** |  |
| --- | --- | --- | --- | --- | --- | --- | --- | --- | --- | --- |
| **n-C_4_H_9_OH** | -233.4250 | -233.2213 | -233.2135 | -55.1879 | -233.2144 | -55.1869 | -55.2075 | -233.2513 | 43.257 |  |
| **CR1(bu)** | -288.6206 | -288.3674 | -288.3572 | -233.4184 | -288.3562 | -233.4174 | -233.4549 | -288.4056 | 78.986 |  |
| **CR2(bu)** | -288.6211 | -288.3692 | -288.3592 | -288.6113 | -288.3583 | -288.6104 | -288.6565 | -288.4070 | 97.064 |  |
| **CR3(bu)** | -288.6231 | -288.3696 | -288.3597 | -288.6123 | -288.3587 | -288.6113 | -288.6554 | -288.4078 | 92.739 |  |
| **CR4(bu)** | -288.6215 | -288.3691 | -288.3592 | -288.6137 | -288.3582 | -288.6127 | -288.6579 | -288.4067 | 95.145 |  |
| **CR5(bu)** | -288.6179 | -288.3669 | -288.3560 | -288.6120 | -288.3550 | -288.6110 | -288.6567 | -288.4073 | 96.103 |  |
| **TS1(bu)** | -288.5924 | -288.3405 | -288.3316 | -288.6080 | -288.3306 | -288.6070 | -288.6543 | -288.3760 | 99.578 |  |
| **TS2(bu)** | -288.5991 | -288.3483 | -288.3390 | -288.5838 | -288.3380 | -288.5829 | -288.6270 | -288.3837 | 92.845 |  |
| **TS3(bu)** | -288.5964 | -288.3453 | -288.3362 | -288.5901 | -288.3353 | -288.5891 | -288.6340 | -288.3798 | 94.401 |  |
| **TS4(bu)** | -288.5961 | -288.3462 | -288.3368 | -288.5875 | -288.3358 | -288.5866 | -288.6307 | -288.3816 | 92.897 |  |
| **TS5(bu)** | -288.5917 | -288.3417 | -288.3325 | -288.5872 | -288.3315 | -288.5863 | -288.6306 | -288.3775 | 93.256 |  |
| **CP1(bu)** | -288.6027 | -288.3511 | -288.3406 | -288.5829 | -288.3397 | -288.5819 | -288.6265 | -288.3884 | 93.718 |  |
| **CP2(bu)** | -288.6137 | -288.3650 | -288.3540 | -288.5933 | -288.3531 | -288.5923 | -288.6377 | -288.4038 | 95.409 |  |
| **CP3(bu)** | -288.6095 | -288.3589 | -288.3477 | -288.6035 | -288.3467 | -288.6025 | -288.6501 | -288.3986 | 100.055 |  |
| **CP4(bu)** | -288.6090 | -288.3598 | -288.3486 | -288.5990 | -288.3476 | -288.5981 | -288.6454 | -288.3987 | 99.717 |  |
| **CP5(bu)** | -288.6035 | -288.3553 | -288.3441 | -288.5990 | -288.3431 | -288.5981 | -288.6451 | -288.3945 | 98.916 |  |
| **CH_3_CH_2_CH_2_CH_2_O** | -55.8293 | -232.5558 | -232.5492 | -288.5939 | -232.5483 | -288.5930 | -288.6388 | -232.5862 | 96.463 |  |
| **CH_3_CH_2_CH_2_CHOH** | -232.7669 | -232.5715 | -232.5645 | -232.7605 | -232.5635 | -232.7596 | -232.7973 | -232.6024 | 79.296 |  |
| **CH_3_CH_2_CHCH_2_OH** | -232.7800 | -232.5638 | -232.5565 | -232.7729 | -232.5556 | -232.7720 | -232.8113 | -232.5957 | 82.653 |  |
| **CH_3_CHCH_2_CH_2_O** | -232.7723 | -232.5660 | -232.5587 | -232.7652 | -232.5578 | -232.7642 | -232.8038 | -232.5974 | 83.297 |  |
| **CH_2_CH_2_CH_2_CH_2_OH** | -232.7745 | -232.5618 | -232.5546 | -232.7675 | -232.5537 | -232.7665 | -232.8055 | -232.5929 | 82.025 |  |

A and B refer to the M06-2X and CBS-QB3 methods, respectively.

**Table S8**. The CCSD(T) absolute energies (in Hartree) and T1 diagnostic values for stationary points of the n-butanol + NH reaction calculated at the CCSD(T)/6-31+g(d**´**)//M06-2X level of theory.

| **Species** | ***CCSD(T)*** | ***T1 diagnostic*** |
| --- | --- | --- |
| **butanol** | -232.9503 | 0.0103 |
| **CR1(bu)** | -288.0374 | 0.0110 |
| **CR2(bu)** | -288.0405 | 0.0104 |
| **CR3(bu)** | -288.0411 | 0.0104 |
| **CR4(bu)** | -288.0403 | 0.0105 |
| **CR5(bu)** | -288.0352 | 0.0105 |
| **TS1(bu)** | -287.9990 | 0.0213 |
| **TS2(bu)** | -288.0012 | 0.0154 |
| **TS3(bu)** | -287.9987 | 0.0143 |
| **TS4(bu)** | -287.9985 | 0.0141 |
| **TS5(bu)** | -287.9939 | 0.0139 |
| **CP1(bu)** | -288.0209 | 0.0153 |
| **CP2(bu)** | -288.0240 | 0.0131 |
| **CP3(bu)** | -288.0181 | 0.0124 |
| **CP4(bu)** | -288.0176 | 0.0121 |
| **CP5(bu)** | -288.0129 | 0.0117 |
| **CH_3_CH_2_CH_2_CH_2_O** | -232.2979 | 0.0152 |
| **CH_3_CH_2_CH_2_CHOH** | -232.3015 | 0.0136 |
| **CH_3_CH_2_CHCH_2_OH** | -232.2935 | 0.0125 |
| **CH_3_CHCH_2_CH_2_OH** | -232.2959 | 0.0122 |
| **CH_2_CH_2_CH_2_CH_2_OH** | -232.2918 | 0.0117 |

**Table S9.** Calculated equilibrium constants for pre-reactive complexes in the CH_3_OH + NH reaction computed at two computational levels.

| T | K_1_(A) | K_1_(B) | K_2_(A) | K_2_(B) |
| --- | --- | --- | --- | --- |
| 300 | 7.61E-23 | 8.55E-24 | 8.82E-23 | 9.33E-23 |
| 400 | 2.73E-23 | 7.97E-24 | 3.22E-23 | 5.06E-23 |
| 500 | 1.65E-23 | 8.58E-24 | 1.97E-23 | 3.92E-23 |
| 600 | 1.27E-23 | 9.75E-24 | 1.53E-23 | 3.57E-23 |
| 700 | 1.12E-23 | 1.13E-23 | 1.36E-23 | 3.53E-23 |
| 800 | 1.06E-23 | 1.32E-23 | 1.29E-23 | 3.66E-23 |
| 900 | 1.05E-23 | 1.54E-23 | 1.29E-23 | 3.89E-23 |
| 1000 | 1.07E-23 | 1.79E-23 | 1.32E-23 | 4.20E-23 |
| 1100 | 1.12E-23 | 2.07E-23 | 1.37E-23 | 4.57E-23 |
| 1200 | 1.17E-23 | 2.38E-23 | 1.45E-23 | 4.99E-23 |
| 1300 | 1.25E-23 | 2.73E-23 | 1.54E-23 | 5.47E-23 |
| 1400 | 1.33E-23 | 3.10E-23 | 1.65E-23 | 5.99E-23 |
| 1500 | 1.42E-23 | 3.51E-23 | 1.76E-23 | 6.56E-23 |
| 1600 | 1.52E-23 | 3.95E-23 | 1.89E-23 | 7.18E-23 |
| 1700 | 1.63E-23 | 4.42E-23 | 2.03E-23 | 7.85E-23 |
| 1800 | 1.75E-23 | 4.93E-23 | 2.18E-23 | 8.56E-23 |
| 1900 | 1.88E-23 | 5.48E-23 | 2.34E-23 | 9.32E-23 |
| 2000 | 2.02E-23 | 6.06E-23 | 2.52E-23 | 1.01E-22 |
| 2100 | 2.16E-23 | 6.67E-23 | 2.70E-23 | 1.10E-22 |
| 2200 | 2.31E-23 | 7.33E-23 | 2.89E-23 | 1.19E-22 |
| 2300 | 2.47E-23 | 8.02E-23 | 3.09E-23 | 1.28E-22 |
| 2400 | 2.64E-23 | 8.76E-23 | 3.30E-23 | 1.38E-22 |
| 2500 | 2.82E-23 | 9.53E-23 | 3.53E-23 | 1.49E-22 |
| 2600 | 3.01E-23 | 1.03E-22 | 3.76E-23 | 1.60E-22 |
| 2700 | 3.20E-23 | 1.12E-22 | 4.01E-23 | 1.71E-22 |
| 2800 | 3.40E-23 | 1.21E-22 | 4.26E-23 | 1.84E-22 |
| 2900 | 3.62E-23 | 1.30E-22 | 4.53E-23 | 1.96E-22 |
| 3000 | 3.84E-23 | 1.40E-22 | 4.81E-23 | 2.09E-22 |

A and B refer to the M06-2X and CBS-QB3 methods, respectively.

**Table S10.** Equilibrium constants for pre-reactive complexes of ethanol plus imidogen reactions computed at two computational levels.

| T | K_1_(A) | K_1_(B) | K_2_(A) | K_2_(B) | K_3_(A) | K_3_(B) |
| --- | --- | --- | --- | --- | --- | --- |
| 300 | 4.11E-24 | 9.25E-24 | 1.54E-22 | 7.92E-23 | 5.25E-23 | 1.92E-23 |
| 400 | 1.25E-24 | 8.75E-24 | 4.00E-23 | 4.08E-23 | 1.37E-23 | 6.74E-24 |
| 500 | 6.44E-25 | 9.50E-24 | 1.99E-23 | 3.06E-23 | 6.76E-24 | 3.83E-24 |
| 600 | 4.32E-25 | 1.09E-23 | 1.35E-23 | 2.73E-23 | 4.54E-24 | 2.75E-24 |
| 700 | 3.35E-25 | 1.27E-23 | 1.08E-23 | 2.66E-23 | 3.61E-24 | 2.24E-24 |
| 800 | 2.83E-25 | 1.48E-23 | 9.57E-24 | 2.73E-23 | 3.17E-24 | 1.97E-24 |
| 900 | 2.54E-25 | 1.73E-23 | 9.00E-24 | 2.87E-23 | 2.97E-24 | 1.82E-24 |
| 1000 | 2.36E-25 | 2.02E-23 | 8.80E-24 | 3.08E-23 | 2.88E-24 | 1.73E-24 |
| 1100 | 2.25E-25 | 2.34E-23 | 8.84E-24 | 3.33E-23 | 2.88E-24 | 1.69E-24 |
| 1200 | 2.19E-25 | 2.69E-23 | 9.04E-24 | 3.62E-23 | 2.93E-24 | 1.67E-24 |
| 1300 | 2.16E-25 | 3.08E-23 | 9.35E-24 | 3.95E-23 | 3.02E-24 | 1.67E-24 |
| 1400 | 2.15E-25 | 3.51E-23 | 9.77E-24 | 4.31E-23 | 3.14E-24 | 1.69E-24 |
| 1500 | 2.16E-25 | 3.97E-23 | 1.03E-23 | 4.71E-23 | 3.29E-24 | 1.71E-24 |
| 1600 | 2.18E-25 | 4.48E-23 | 1.08E-23 | 5.14E-23 | 3.46E-24 | 1.74E-24 |
| 1700 | 2.20E-25 | 5.02E-23 | 1.14E-23 | 5.60E-23 | 3.65E-24 | 1.78E-24 |
| 1800 | 2.24E-25 | 5.60E-23 | 1.21E-23 | 6.10E-23 | 3.86E-24 | 1.83E-24 |
| 1900 | 2.28E-25 | 6.22E-23 | 1.29E-23 | 6.63E-23 | 4.09E-24 | 1.87E-24 |
| 2000 | 2.33E-25 | 6.88E-23 | 1.37E-23 | 7.19E-23 | 4.33E-24 | 1.93E-24 |
| 2100 | 2.39E-25 | 7.58E-23 | 1.45E-23 | 7.78E-23 | 4.59E-24 | 1.98E-24 |
| 2200 | 2.44E-25 | 8.33E-23 | 1.54E-23 | 8.41E-23 | 4.87E-24 | 2.04E-24 |
| 2300 | 2.50E-25 | 9.12E-23 | 1.64E-23 | 9.07E-23 | 5.16E-24 | 2.10E-24 |
| 2400 | 2.57E-25 | 9.96E-23 | 1.74E-23 | 9.77E-23 | 5.46E-24 | 2.16E-24 |
| 2500 | 2.63E-25 | 1.08E-22 | 1.84E-23 | 1.05E-22 | 5.79E-24 | 2.23E-24 |
| 2600 | 2.70E-25 | 1.18E-22 | 1.95E-23 | 1.13E-22 | 6.12E-24 | 2.29E-24 |
| 2700 | 2.78E-25 | 1.27E-22 | 2.07E-23 | 1.21E-22 | 6.48E-24 | 2.36E-24 |
| 2800 | 2.85E-25 | 1.38E-22 | 2.19E-23 | 1.29E-22 | 6.84E-24 | 2.43E-24 |
| 2900 | 2.92E-25 | 1.48E-22 | 2.31E-23 | 1.38E-22 | 7.23E-24 | 2.51E-24 |
| 3000 | 3.00E-25 | 1.60E-22 | 2.44E-23 | 1.47E-22 | 7.63E-24 | 2.58E-24 |

**Table S11.** Calculated equilibrium constants for pre-reactive complexes in the n-C_3_H_7_OH + NH reaction computed at two computational levels.

| T | K_1_(A) | K_1_(B) | K_2_(A) | K_2_(B) | K_3_(A) | K_3_(B) | K_4_(A) | K_4_(B) |
| --- | --- | --- | --- | --- | --- | --- | --- | --- |
| 300 | 1.77E-23 | 1.04E-22 | 6.45E-23 | 1.38E-22 | 1.13E-22 | 1.38E-22 | 6.81E-24 | 3.72E-22 |
| 400 | 6.37E-24 | 4.98E-23 | 1.70E-23 | 6.54E-23 | 2.81E-23 | 6.56E-23 | 5.67E-24 | 4.43E-22 |
| 500 | 3.85E-24 | 3.58E-23 | 8.46E-24 | 4.68E-23 | 1.35E-23 | 4.70E-23 | 5.71E-24 | 5.58E-22 |
| 600 | 2.96E-24 | 3.11E-23 | 5.72E-24 | 4.05E-23 | 8.96E-24 | 4.06E-23 | 6.21E-24 | 7.07E-22 |
| 700 | 2.60E-24 | 2.97E-23 | 4.57E-24 | 3.86E-23 | 7.05E-24 | 3.87E-23 | 6.98E-24 | 8.89E-22 |
| 800 | 2.46E-24 | 3.00E-23 | 4.02E-24 | 3.89E-23 | 6.15E-24 | 3.90E-23 | 7.96E-24 | 1.10E-21 |
| 900 | 2.43E-24 | 3.13E-23 | 3.77E-24 | 4.05E-23 | 5.71E-24 | 4.06E-23 | 9.12E-24 | 1.35E-21 |
| 1000 | 2.47E-24 | 3.32E-23 | 3.67E-24 | 4.29E-23 | 5.53E-24 | 4.31E-23 | 1.05E-23 | 1.63E-21 |
| 1100 | 2.56E-24 | 3.57E-23 | 3.67E-24 | 4.61E-23 | 5.50E-24 | 4.62E-23 | 1.19E-23 | 1.95E-21 |
| 1200 | 2.69E-24 | 3.86E-23 | 3.75E-24 | 4.98E-23 | 5.58E-24 | 5.00E-23 | 1.36E-23 | 2.31E-21 |
| 1300 | 2.85E-24 | 4.19E-23 | 3.87E-24 | 5.40E-23 | 5.74E-24 | 5.42E-23 | 1.54E-23 | 2.71E-21 |
| 1400 | 3.03E-24 | 4.56E-23 | 4.03E-24 | 5.87E-23 | 5.96E-24 | 5.89E-23 | 1.74E-23 | 3.14E-21 |
| 1500 | 3.24E-24 | 4.97E-23 | 4.22E-24 | 6.39E-23 | 6.22E-24 | 6.41E-23 | 1.96E-23 | 3.62E-21 |
| 1600 | 3.46E-24 | 5.40E-23 | 4.44E-24 | 6.94E-23 | 6.53E-24 | 6.97E-23 | 2.19E-23 | 4.14E-21 |
| 1700 | 3.71E-24 | 5.87E-23 | 4.69E-24 | 7.55E-23 | 6.88E-24 | 7.57E-23 | 2.44E-23 | 4.70E-21 |
| 1800 | 3.97E-24 | 6.38E-23 | 4.96E-24 | 8.19E-23 | 7.27E-24 | 8.22E-23 | 2.71E-23 | 5.31E-21 |
| 1900 | 4.26E-24 | 6.92E-23 | 5.26E-24 | 8.88E-23 | 7.68E-24 | 8.91E-23 | 3.00E-23 | 5.96E-21 |
| 2000 | 4.56E-24 | 7.49E-23 | 5.57E-24 | 9.61E-23 | 8.13E-24 | 9.65E-23 | 3.31E-23 | 6.66E-21 |
| 2100 | 4.88E-24 | 8.09E-23 | 5.91E-24 | 1.04E-22 | 8.61E-24 | 1.04E-22 | 3.64E-23 | 7.41E-21 |
| 2200 | 5.21E-24 | 8.73E-23 | 6.26E-24 | 1.12E-22 | 9.12E-24 | 1.12E-22 | 3.98E-23 | 8.20E-21 |
| 2300 | 5.57E-24 | 9.41E-23 | 6.64E-24 | 1.21E-22 | 9.65E-24 | 1.21E-22 | 4.35E-23 | 9.05E-21 |
| 2400 | 5.94E-24 | 1.01E-22 | 7.04E-24 | 1.30E-22 | 1.02E-23 | 1.30E-22 | 4.74E-23 | 9.95E-21 |
| 2500 | 6.34E-24 | 1.09E-22 | 7.46E-24 | 1.39E-22 | 1.08E-23 | 1.40E-22 | 5.14E-23 | 1.09E-20 |
| 2600 | 6.75E-24 | 1.16E-22 | 7.89E-24 | 1.49E-22 | 1.14E-23 | 1.50E-22 | 5.57E-23 | 1.19E-20 |
| 2700 | 7.17E-24 | 1.25E-22 | 8.35E-24 | 1.60E-22 | 1.21E-23 | 1.60E-22 | 6.02E-23 | 1.30E-20 |
| 2800 | 7.62E-24 | 1.33E-22 | 8.83E-24 | 1.71E-22 | 1.28E-23 | 1.71E-22 | 6.49E-23 | 1.41E-20 |
| 2900 | 8.09E-24 | 1.42E-22 | 9.32E-24 | 1.82E-22 | 1.35E-23 | 1.83E-22 | 6.98E-23 | 1.52E-20 |
| 3000 | 8.57E-24 | 1.51E-22 | 9.84E-24 | 1.94E-22 | 1.42E-23 | 1.95E-22 | 7.50E-23 | 1.65E-20 |

A and B refer to the M06-2X and CBS-QB3 methods, respectively.

**Table S12.** Calculated equilibrium constants for pre-reactive complexes in the n-C_4_H_9_OH + NH reaction computed at two computational levels.

| T | K_1_(A) | K_1_(B) | K_2_(A) | K_2_(B) | K_3_(A) | K_3_(B) | K_4_(A) | K_4_(B) | K_5_(A) | K_5_(B) |
| --- | --- | --- | --- | --- | --- | --- | --- | --- | --- | --- |
| 300 | 7.88E-23 | 1.60E-23 | 2.44E-23 | 7.15E-23 | 3.68E-22 | 1.61E-22 | 4.82E-22 | 5.01E-23 | 8.37E-24 | 9.61E-23 |
| 400 | 2.20E-23 | 1.48E-23 | 5.26E-24 | 3.90E-23 | 5.67E-23 | 7.79E-23 | 7.84E-23 | 2.74E-23 | 5.87E-24 | 1.24E-22 |
| 500 | 1.09E-23 | 1.59E-23 | 2.22E-24 | 3.03E-23 | 2.06E-23 | 5.63E-23 | 2.94E-23 | 2.13E-23 | 5.34E-24 | 1.64E-22 |
| 600 | 7.20E-24 | 1.81E-23 | 1.31E-24 | 2.76E-23 | 1.13E-23 | 4.90E-23 | 1.65E-23 | 1.95E-23 | 5.44E-24 | 2.14E-22 |
| 700 | 5.53E-24 | 2.09E-23 | 9.27E-25 | 2.74E-23 | 7.78E-24 | 4.69E-23 | 1.16E-23 | 1.93E-23 | 5.84E-24 | 2.75E-22 |
| 800 | 4.66E-24 | 2.44E-23 | 7.34E-25 | 2.83E-23 | 6.14E-24 | 4.74E-23 | 9.27E-24 | 2.00E-23 | 6.44E-24 | 3.48E-22 |
| 900 | 4.16E-24 | 2.85E-23 | 6.24E-25 | 3.01E-23 | 5.28E-24 | 4.94E-23 | 8.06E-24 | 2.13E-23 | 7.18E-24 | 4.32E-22 |
| 1000 | 3.86E-24 | 3.31E-23 | 5.56E-25 | 3.25E-23 | 4.81E-24 | 5.25E-23 | 7.40E-24 | 2.30E-23 | 8.06E-24 | 5.28E-22 |
| 1100 | 3.68E-24 | 3.82E-23 | 5.13E-25 | 3.54E-23 | 4.55E-24 | 5.65E-23 | 7.06E-24 | 2.51E-23 | 9.06E-24 | 6.37E-22 |
| 1200 | 3.57E-24 | 4.39E-23 | 4.86E-25 | 3.87E-23 | 4.43E-24 | 6.11E-23 | 6.91E-24 | 2.74E-23 | 1.02E-23 | 7.59E-22 |
| 1300 | 3.52E-24 | 5.02E-23 | 4.67E-25 | 4.23E-23 | 4.40E-24 | 6.63E-23 | 6.90E-24 | 3.01E-23 | 1.14E-23 | 8.95E-22 |
| 1400 | 3.50E-24 | 5.71E-23 | 4.56E-25 | 4.64E-23 | 4.43E-24 | 7.22E-23 | 6.99E-24 | 3.30E-23 | 1.28E-23 | 1.04E-21 |
| 1500 | 3.51E-24 | 6.45E-23 | 4.50E-25 | 5.08E-23 | 4.51E-24 | 7.85E-23 | 7.14E-24 | 3.61E-23 | 1.42E-23 | 1.21E-21 |
| 1600 | 3.54E-24 | 7.26E-23 | 4.47E-25 | 5.56E-23 | 4.63E-24 | 8.55E-23 | 7.36E-24 | 3.95E-23 | 1.58E-23 | 1.39E-21 |
| 1700 | 3.59E-24 | 8.13E-23 | 4.47E-25 | 6.07E-23 | 4.78E-24 | 9.29E-23 | 7.62E-24 | 4.32E-23 | 1.75E-23 | 1.58E-21 |
| 1800 | 3.65E-24 | 9.06E-23 | 4.49E-25 | 6.62E-23 | 4.96E-24 | 1.01E-22 | 7.93E-24 | 4.71E-23 | 1.94E-23 | 1.79E-21 |
| 1900 | 3.72E-24 | 1.01E-22 | 4.53E-25 | 7.21E-23 | 5.16E-24 | 1.09E-22 | 8.28E-24 | 5.13E-23 | 2.13E-23 | 2.02E-21 |
| 2000 | 3.80E-24 | 1.11E-22 | 4.58E-25 | 7.83E-23 | 5.39E-24 | 1.18E-22 | 8.66E-24 | 5.58E-23 | 2.34E-23 | 2.26E-21 |
| 2100 | 3.89E-24 | 1.22E-22 | 4.64E-25 | 8.49E-23 | 5.63E-24 | 1.28E-22 | 9.07E-24 | 6.05E-23 | 2.56E-23 | 2.52E-21 |
| 2200 | 3.98E-24 | 1.34E-22 | 4.72E-25 | 9.19E-23 | 5.89E-24 | 1.38E-22 | 9.51E-24 | 6.55E-23 | 2.79E-23 | 2.80E-21 |
| 2300 | 4.08E-24 | 1.47E-22 | 4.80E-25 | 9.93E-23 | 6.18E-24 | 1.49E-22 | 9.99E-24 | 7.07E-23 | 3.04E-23 | 3.09E-21 |
| 2400 | 4.19E-24 | 1.61E-22 | 4.89E-25 | 1.07E-22 | 6.47E-24 | 1.60E-22 | 1.05E-23 | 7.63E-23 | 3.30E-23 | 3.41E-21 |
| 2500 | 4.29E-24 | 1.75E-22 | 4.98E-25 | 1.15E-22 | 6.79E-24 | 1.72E-22 | 1.10E-23 | 8.21E-23 | 3.57E-23 | 3.74E-21 |
| 2600 | 4.41E-24 | 1.90E-22 | 5.09E-25 | 1.24E-22 | 7.12E-24 | 1.84E-22 | 1.16E-23 | 8.82E-23 | 3.86E-23 | 4.09E-21 |
| 2700 | 4.53E-24 | 2.05E-22 | 5.20E-25 | 1.33E-22 | 7.47E-24 | 1.97E-22 | 1.22E-23 | 9.45E-23 | 4.16E-23 | 4.46E-21 |
| 2800 | 4.65E-24 | 2.22E-22 | 5.31E-25 | 1.42E-22 | 7.83E-24 | 2.11E-22 | 1.28E-23 | 1.01E-22 | 4.48E-23 | 4.84E-21 |
| 2900 | 4.77E-24 | 2.39E-22 | 5.43E-25 | 1.52E-22 | 8.21E-24 | 2.25E-22 | 1.34E-23 | 1.08E-22 | 4.81E-23 | 5.25E-21 |
| 3000 | 4.90E-24 | 2.57E-22 | 5.55E-25 | 1.62E-22 | 8.61E-24 | 2.39E-22 | 1.41E-23 | 1.15E-22 | 5.15E-23 | 5.68E-21 |

A and B refers to the M06-2X and CBS-QB3 methods, respectively.

**Table S13**. Rate constants (cm^3^ molecule^-1^ s^-1^) of all channels in the methanol plus NH reaction.

| T | k_1_(A) | k_1_(B) | k_2_(A) | k_2_(B) |
| --- | --- | --- | --- | --- |
| 300 | 1.96E-22 | 3.17E-24 | 3.45E-20 | 5.81E-21 |
| 400 | 5.51E-20 | 2.24E-21 | 1.10E-18 | 3.66E-19 |
| 500 | 2.05E-18 | 1.43E-19 | 1.54E-17 | 7.65E-18 |
| 600 | 2.60E-17 | 2.58E-18 | 1.13E-16 | 7.24E-17 |
| 700 | 1.74E-16 | 2.21E-17 | 5.35E-16 | 4.07E-16 |
| 800 | 7.71E-16 | 1.17E-16 | 1.86E-15 | 1.61E-15 |
| 900 | 2.58E-15 | 4.51E-16 | 5.20E-15 | 4.94E-15 |
| 1000 | 7.05E-15 | 1.37E-15 | 1.24E-14 | 1.27E-14 |
| 1100 | 1.65E-14 | 3.52E-15 | 2.59E-14 | 2.83E-14 |
| 1200 | 3.45E-14 | 7.89E-15 | 4.94E-14 | 5.67E-14 |
| 1300 | 6.57E-14 | 1.60E-14 | 8.71E-14 | 1.04E-13 |
| 1400 | 1.16E-13 | 2.97E-14 | 1.44E-13 | 1.79E-13 |
| 1500 | 1.93E-13 | 5.16E-14 | 2.27E-13 | 2.91E-13 |
| 1600 | 3.05E-13 | 8.49E-14 | 3.41E-13 | 4.50E-13 |
| 1700 | 4.63E-13 | 1.33E-13 | 4.95E-13 | 6.69E-13 |
| 1800 | 6.76E-13 | 2.00E-13 | 6.97E-13 | 9.60E-13 |
| 1900 | 9.57E-13 | 2.91E-13 | 9.53E-13 | 1.34E-12 |
| 2000 | 1.32E-12 | 4.12E-13 | 1.27E-12 | 1.82E-12 |
| 2100 | 1.78E-12 | 5.66E-13 | 1.67E-12 | 2.42E-12 |
| 2200 | 2.34E-12 | 7.62E-13 | 2.14E-12 | 3.15E-12 |
| 2300 | 3.04E-12 | 1.00E-12 | 2.71E-12 | 4.03E-12 |
| 2400 | 3.87E-12 | 1.30E-12 | 3.37E-12 | 5.09E-12 |
| 2500 | 4.85E-12 | 1.66E-12 | 4.15E-12 | 6.32E-12 |
| 2600 | 6.01E-12 | 2.08E-12 | 5.05E-12 | 7.76E-12 |
| 2700 | 7.36E-12 | 2.58E-12 | 6.07E-12 | 9.42E-12 |
| 2800 | 8.92E-12 | 3.16E-12 | 7.24E-12 | 1.13E-11 |
| 2900 | 1.07E-11 | 3.83E-12 | 8.55E-12 | 1.35E-11 |
| 3000 | 1.27E-11 | 4.60E-12 | 1.00E-11 | 1.59E-11 |

A and B refer to the M06-2X and CBS-QB3 methods, respectively

**Table S14**. Rate constants (cm^3^ molecule^-1^ s^-1^) of all channels in the ethanol plus NH reaction.

| T | k_1_(A) | k_1_(B) | k_2_(A) | k_2_(B) | k_3_(A) | k_3_(B) |
| --- | --- | --- | --- | --- | --- | --- |
| 300 | 5.78E-23 | 5.65E-24 | 4.98E-19 | 1.43E-19 | 3.70E-23 | 7.95E-24 |
| 400 | 1.52E-20 | 3.55E-21 | 8.75E-18 | 4.59E-18 | 7.99E-21 | 2.64E-21 |
| 500 | 5.46E-19 | 2.11E-19 | 8.10E-17 | 5.93E-17 | 2.87E-19 | 1.25E-19 |
| 600 | 6.75E-18 | 3.64E-18 | 4.46E-16 | 4.00E-16 | 3.72E-18 | 1.96E-18 |
| 700 | 4.42E-17 | 3.03E-17 | 1.70E-15 | 1.76E-15 | 2.58E-17 | 1.56E-17 |
| 800 | 1.93E-16 | 1.58E-16 | 5.05E-15 | 5.77E-15 | 1.19E-16 | 7.93E-17 |
| 900 | 6.35E-16 | 5.97E-16 | 1.25E-14 | 1.54E-14 | 4.11E-16 | 2.97E-16 |
| 1000 | 1.71E-15 | 1.80E-15 | 2.68E-14 | 3.50E-14 | 1.16E-15 | 8.90E-16 |
| 1100 | 3.96E-15 | 4.56E-15 | 5.17E-14 | 7.10E-14 | 2.78E-15 | 2.26E-15 |
| 1200 | 8.18E-15 | 1.02E-14 | 9.20E-14 | 1.31E-13 | 5.93E-15 | 5.03E-15 |
| 1300 | 1.54E-14 | 2.04E-14 | 1.53E-13 | 2.26E-13 | 1.15E-14 | 1.01E-14 |
| 1400 | 2.70E-14 | 3.78E-14 | 2.41E-13 | 3.66E-13 | 2.07E-14 | 1.88E-14 |
| 1500 | 4.46E-14 | 6.54E-14 | 3.63E-13 | 5.65E-13 | 3.49E-14 | 3.25E-14 |
| 1600 | 7.00E-14 | 1.07E-13 | 5.25E-13 | 8.36E-13 | 5.58E-14 | 5.32E-14 |
| 1700 | 1.05E-13 | 1.67E-13 | 7.37E-13 | 1.19E-12 | 8.54E-14 | 8.33E-14 |
| 1800 | 1.53E-13 | 2.51E-13 | 1.01E-12 | 1.66E-12 | 1.26E-13 | 1.25E-13 |
| 1900 | 2.16E-13 | 3.64E-13 | 1.34E-12 | 2.24E-12 | 1.80E-13 | 1.82E-13 |
| 2000 | 2.96E-13 | 5.13E-13 | 1.75E-12 | 2.96E-12 | 2.50E-13 | 2.56E-13 |
| 2100 | 3.97E-13 | 7.04E-13 | 2.24E-12 | 3.83E-12 | 3.38E-13 | 3.51E-13 |
| 2200 | 5.21E-13 | 9.45E-13 | 2.82E-12 | 4.88E-12 | 4.48E-13 | 4.72E-13 |
| 2300 | 6.72E-13 | 1.24E-12 | 3.50E-12 | 6.12E-12 | 5.83E-13 | 6.21E-13 |
| 2400 | 8.53E-13 | 1.61E-12 | 4.29E-12 | 7.56E-12 | 7.46E-13 | 8.03E-13 |
| 2500 | 1.07E-12 | 2.04E-12 | 5.19E-12 | 9.24E-12 | 9.40E-13 | 1.02E-12 |
| 2600 | 1.32E-12 | 2.56E-12 | 6.22E-12 | 1.12E-11 | 1.17E-12 | 1.28E-12 |
| 2700 | 1.61E-12 | 3.17E-12 | 7.39E-12 | 1.33E-11 | 1.44E-12 | 1.59E-12 |
| 2800 | 1.94E-12 | 3.88E-12 | 8.70E-12 | 1.58E-11 | 1.74E-12 | 1.94E-12 |
| 2900 | 2.33E-12 | 4.70E-12 | 1.02E-11 | 1.86E-11 | 2.10E-12 | 2.35E-12 |
| 3000 | 2.76E-12 | 5.64E-12 | 1.18E-11 | 2.17E-11 | 2.50E-12 | 2.82E-12 |

A and B refer to the M06-2X and CBS-QB3 methods, respectively. Also, S = $\sum_{i=1}^{N} ki$

**Table S15**. Rate constants (cm^3^ molecule^-1^ s^-1^) of all channels in the n-propanol plus NH reaction.

| T | k_1_(A) | k_1_(B) | k_2_(A) | k_2_(B) | k_3_(A) | k_3_(B) | k_4_(A) | k_4_(B) |
| --- | --- | --- | --- | --- | --- | --- | --- | --- |
| 300 | 1.50E-22 | 1.65E-23 | 3.45E-19 | 2.78E-19 | 1.91E-20 | 2.24E-21 | 1.35E-23 | 3.38E-23 |
| 400 | 3.44E-20 | 8.85E-21 | 6.04E-18 | 8.37E-18 | 1.19E-18 | 1.84E-19 | 4.49E-21 | 1.27E-20 |
| 500 | 1.13E-18 | 4.79E-19 | 5.26E-17 | 9.93E-17 | 2.07E-17 | 3.91E-18 | 2.14E-19 | 6.56E-19 |
| 600 | 1.31E-17 | 7.77E-18 | 2.74E-16 | 6.27E-16 | 1.67E-16 | 3.61E-17 | 3.40E-18 | 1.09E-17 |
| 700 | 8.21E-17 | 6.18E-17 | 1.00E-15 | 2.62E-15 | 8.30E-16 | 1.98E-16 | 2.74E-17 | 9.13E-17 |
| 800 | 3.47E-16 | 3.12E-16 | 2.86E-15 | 8.27E-15 | 2.97E-15 | 7.66E-16 | 1.41E-16 | 4.83E-16 |
| 900 | 1.11E-15 | 1.15E-15 | 6.85E-15 | 2.14E-14 | 8.47E-15 | 2.32E-15 | 5.35E-16 | 1.87E-15 |
| 1000 | 2.94E-15 | 3.39E-15 | 1.44E-14 | 4.75E-14 | 2.04E-14 | 5.85E-15 | 1.62E-15 | 5.73E-15 |
| 1100 | 6.71E-15 | 8.47E-15 | 2.72E-14 | 9.45E-14 | 4.32E-14 | 1.29E-14 | 4.13E-15 | 1.48E-14 |
| 1200 | 1.37E-14 | 1.86E-14 | 4.76E-14 | 1.72E-13 | 8.30E-14 | 2.56E-14 | 9.27E-15 | 3.36E-14 |
| 1300 | 2.55E-14 | 3.70E-14 | 7.80E-14 | 2.91E-13 | 1.47E-13 | 4.67E-14 | 1.88E-14 | 6.87E-14 |
| 1400 | 4.43E-14 | 6.78E-14 | 1.21E-13 | 4.66E-13 | 2.45E-13 | 7.96E-14 | 3.50E-14 | 1.29E-13 |
| 1500 | 7.25E-14 | 1.16E-13 | 1.80E-13 | 7.11E-13 | 3.87E-13 | 1.28E-13 | 6.09E-14 | 2.26E-13 |
| 1600 | 1.13E-13 | 1.89E-13 | 2.59E-13 | 1.04E-12 | 5.84E-13 | 1.97E-13 | 1.00E-13 | 3.74E-13 |
| 1700 | 1.69E-13 | 2.94E-13 | 3.60E-13 | 1.48E-12 | 8.49E-13 | 2.92E-13 | 1.57E-13 | 5.90E-13 |
| 1800 | 2.44E-13 | 4.38E-13 | 4.87E-13 | 2.04E-12 | 1.20E-12 | 4.17E-13 | 2.37E-13 | 8.93E-13 |
| 1900 | 3.42E-13 | 6.33E-13 | 6.45E-13 | 2.73E-12 | 1.64E-12 | 5.80E-13 | 3.45E-13 | 1.31E-12 |
| 2000 | 4.68E-13 | 8.88E-13 | 8.35E-13 | 3.59E-12 | 2.20E-12 | 7.85E-13 | 4.88E-13 | 1.85E-12 |
| 2100 | 6.25E-13 | 1.21E-12 | 1.06E-12 | 4.63E-12 | 2.88E-12 | 1.04E-12 | 6.72E-13 | 2.56E-12 |
| 2200 | 8.17E-13 | 1.62E-12 | 1.33E-12 | 5.87E-12 | 3.71E-12 | 1.35E-12 | 9.04E-13 | 3.45E-12 |
| 2300 | 1.05E-12 | 2.13E-12 | 1.65E-12 | 7.32E-12 | 4.69E-12 | 1.73E-12 | 1.19E-12 | 4.57E-12 |
| 2400 | 1.33E-12 | 2.74E-12 | 2.01E-12 | 9.02E-12 | 5.85E-12 | 2.17E-12 | 1.54E-12 | 5.93E-12 |
| 2500 | 1.66E-12 | 3.48E-12 | 2.42E-12 | 1.10E-11 | 7.21E-12 | 2.70E-12 | 1.97E-12 | 7.58E-12 |
| 2600 | 2.04E-12 | 4.35E-12 | 2.89E-12 | 1.32E-11 | 8.77E-12 | 3.30E-12 | 2.47E-12 | 9.54E-12 |
| 2700 | 2.49E-12 | 5.38E-12 | 3.42E-12 | 1.57E-11 | 1.06E-11 | 4.01E-12 | 3.07E-12 | 1.19E-11 |
| 2800 | 3.00E-12 | 6.57E-12 | 4.02E-12 | 1.86E-11 | 1.26E-11 | 4.81E-12 | 3.76E-12 | 1.46E-11 |
| 2900 | 3.58E-12 | 7.94E-12 | 4.68E-12 | 2.18E-11 | 1.49E-11 | 5.71E-12 | 4.55E-12 | 1.77E-11 |
| 3000 | 4.24E-12 | 9.51E-12 | 5.41E-12 | 2.54E-11 | 1.75E-11 | 6.73E-12 | 5.47E-12 | 2.13E-11 |

A and B refers to the M06-2X/6-31+G(d,p) and CBS-QB3 levels, respectively. Also, S = $\sum_{i=1}^{N} ki$.

**Table S16**. Rate constants (cm^3^ molecule^-1^ s^-1^) of all channels in the n-butanol plus NH reaction.

| T | k_1_(A) | k_1_(B) | k_2_(A) | k_2_(B) | k_3_(A) | k_3_(B) | k_4_(A) | k_4_(B) | k_5_(A) | k_5_(B) |
| --- | --- | --- | --- | --- | --- | --- | --- | --- | --- | --- |
| 300 | 8.28E-23 | 1.19E-23 | 7.96E-19 | 1.96E-19 | 1.39E-20 | 2.69E-21 | 1.51E-20 | 1.77E-20 | 1.67E-22 | 2.19E-22 |
| 400 | 1.97E-20 | 5.98E-21 | 1.59E-17 | 6.62E-18 | 7.05E-19 | 2.09E-19 | 7.46E-19 | 1.19E-18 | 2.81E-20 | 4.73E-20 |
| 500 | 6.64E-19 | 3.13E-19 | 1.47E-16 | 8.38E-17 | 1.11E-17 | 4.20E-18 | 1.17E-17 | 2.20E-17 | 9.01E-19 | 1.76E-18 |
| 600 | 7.90E-18 | 4.98E-18 | 7.92E-16 | 5.52E-16 | 8.37E-17 | 3.75E-17 | 8.94E-17 | 1.86E-16 | 1.10E-17 | 2.36E-17 |
| 700 | 5.04E-17 | 3.91E-17 | 2.97E-15 | 2.37E-15 | 3.97E-16 | 2.00E-16 | 4.28E-16 | 9.56E-16 | 7.36E-17 | 1.68E-16 |
| 800 | 2.15E-16 | 1.95E-16 | 8.64E-15 | 7.64E-15 | 1.38E-15 | 7.59E-16 | 1.50E-15 | 3.52E-15 | 3.31E-16 | 7.95E-16 |
| 900 | 6.97E-16 | 7.12E-16 | 2.10E-14 | 2.01E-14 | 3.82E-15 | 2.26E-15 | 4.20E-15 | 1.03E-14 | 1.12E-15 | 2.81E-15 |
| 1000 | 1.85E-15 | 2.09E-15 | 4.45E-14 | 4.52E-14 | 9.01E-15 | 5.64E-15 | 9.98E-15 | 2.52E-14 | 3.12E-15 | 8.03E-15 |
| 1100 | 4.26E-15 | 5.18E-15 | 8.51E-14 | 9.08E-14 | 1.88E-14 | 1.23E-14 | 2.10E-14 | 5.43E-14 | 7.44E-15 | 1.96E-14 |
| 1200 | 8.71E-15 | 1.13E-14 | 1.50E-13 | 1.67E-13 | 3.56E-14 | 2.42E-14 | 3.99E-14 | 1.06E-13 | 1.58E-14 | 4.24E-14 |
| 1300 | 1.63E-14 | 2.24E-14 | 2.48E-13 | 2.84E-13 | 6.24E-14 | 4.39E-14 | 7.03E-14 | 1.89E-13 | 3.04E-14 | 8.31E-14 |
| 1400 | 2.84E-14 | 4.10E-14 | 3.87E-13 | 4.58E-13 | 1.03E-13 | 7.43E-14 | 1.16E-13 | 3.18E-13 | 5.43E-14 | 1.51E-13 |
| 1500 | 4.66E-14 | 7.01E-14 | 5.79E-13 | 7.02E-13 | 1.61E-13 | 1.19E-13 | 1.83E-13 | 5.07E-13 | 9.12E-14 | 2.56E-13 |
| 1600 | 7.28E-14 | 1.14E-13 | 8.35E-13 | 1.03E-12 | 2.41E-13 | 1.82E-13 | 2.75E-13 | 7.71E-13 | 1.45E-13 | 4.13E-13 |
| 1700 | 1.09E-13 | 1.76E-13 | 1.17E-12 | 1.47E-12 | 3.48E-13 | 2.69E-13 | 3.99E-13 | 1.13E-12 | 2.22E-13 | 6.37E-13 |
| 1800 | 1.58E-13 | 2.62E-13 | 1.58E-12 | 2.03E-12 | 4.88E-13 | 3.83E-13 | 5.60E-13 | 1.60E-12 | 3.26E-13 | 9.45E-13 |
| 1900 | 2.22E-13 | 3.77E-13 | 2.10E-12 | 2.74E-12 | 6.65E-13 | 5.30E-13 | 7.65E-13 | 2.21E-12 | 4.65E-13 | 1.36E-12 |
| 2000 | 3.03E-13 | 5.28E-13 | 2.73E-12 | 3.61E-12 | 8.85E-13 | 7.15E-13 | 1.02E-12 | 2.97E-12 | 6.44E-13 | 1.89E-12 |
| 2100 | 4.06E-13 | 7.21E-13 | 3.49E-12 | 4.66E-12 | 1.16E-12 | 9.45E-13 | 1.34E-12 | 3.91E-12 | 8.71E-13 | 2.58E-12 |
| 2200 | 5.32E-13 | 9.63E-13 | 4.38E-12 | 5.92E-12 | 1.48E-12 | 1.23E-12 | 1.72E-12 | 5.05E-12 | 1.15E-12 | 3.43E-12 |
| 2300 | 6.84E-13 | 1.26E-12 | 5.43E-12 | 7.40E-12 | 1.87E-12 | 1.56E-12 | 2.17E-12 | 6.42E-12 | 1.50E-12 | 4.48E-12 |
| 2400 | 8.66E-13 | 1.62E-12 | 6.64E-12 | 9.13E-12 | 2.32E-12 | 1.96E-12 | 2.70E-12 | 8.03E-12 | 1.91E-12 | 5.75E-12 |
| 2500 | 1.08E-12 | 2.06E-12 | 8.03E-12 | 1.11E-11 | 2.85E-12 | 2.43E-12 | 3.32E-12 | 9.92E-12 | 2.41E-12 | 7.27E-12 |
| 2600 | 1.33E-12 | 2.57E-12 | 9.60E-12 | 1.34E-11 | 3.46E-12 | 2.97E-12 | 4.04E-12 | 1.21E-11 | 2.99E-12 | 9.07E-12 |
| 2700 | 1.63E-12 | 3.17E-12 | 1.14E-11 | 1.60E-11 | 4.15E-12 | 3.59E-12 | 4.86E-12 | 1.46E-11 | 3.67E-12 | 1.12E-11 |
| 2800 | 1.96E-12 | 3.87E-12 | 1.34E-11 | 1.89E-11 | 4.94E-12 | 4.30E-12 | 5.79E-12 | 1.75E-11 | 4.46E-12 | 1.36E-11 |
| 2900 | 2.34E-12 | 4.67E-12 | 1.56E-11 | 2.22E-11 | 5.83E-12 | 5.11E-12 | 6.83E-12 | 2.07E-11 | 5.35E-12 | 1.64E-11 |
| 3000 | 2.77E-12 | 5.59E-12 | 1.81E-11 | 2.59E-11 | 6.82E-12 | 6.01E-12 | 8.00E-12 | 2.44E-11 | 6.37E-12 | 1.96E-11 |

A and B refer to the M06-2X and CBS-QB3 methods, respectively. Also, S = $\sum_{i=1}^{N} ki$.

**Table S17**. Calculated pressure-dependent rate constants for the H atom transfer of the methyl group in the methanol plus NH reaction at the CBS-QB3 method.

| T/K | P/bar | | k (cm^3^ mol^−1^ s^−1^) | | P/bar | | k (cm^3^ mol^−1^ s^−1^) | | P/bar | | k (cm^3^ mol^−1^ s^−1^) | P/bar | k (cm^3^ mol^−1^ s^−1^) |  |  |
| --- | --- | --- | --- | --- | --- | --- | --- | --- | --- | --- | --- | --- | --- | --- | --- |
| 300 | | | | 1.00E-07 | | 1.34E-22 | | 1.00E-06 | | 3.99E-22 | | 1.00E-05 | 9.89E-22 | 1.00E-04 | 2.01E-21 |
| 400 | | | | 1.00E-07 | | 2.71E-22 | | 1.00E-06 | | 1.19E-21 | | 1.00E-05 | 4.65E-21 | 1.00E-04 | 1.59E-20 |
| 500 | | | | 1.00E-07 | | 3.69E-22 | | 1.00E-06 | | 2.21E-21 | | 1.00E-05 | 1.16E-20 | 1.00E-04 | 5.43E-20 |
| 600 | | | | 1.00E-07 | | 5.71E-22 | | 1.00E-06 | | 3.83E-21 | | 1.00E-05 | 2.30E-20 | 1.00E-04 | 1.26E-19 |
| 700 | | | | 1.00E-07 | | 7.03E-22 | | 1.00E-06 | | 4.46E-21 | | 1.00E-05 | 3.15E-20 | 1.00E-04 | 2.11E-19 |
| 800 | | | | 1.00E-07 | | 9.90E-22 | | 1.00E-06 | | 6.55E-21 | | 1.00E-05 | 4.90E-20 | 1.00E-04 | 3.52E-19 |
| 900 | | | | 1.00E-07 | | 1.58E-21 | | 1.00E-06 | | 9.87E-21 | | 1.00E-05 | 5.91E-20 | 1.00E-04 | 4.29E-19 |
| 1000 | | | | 1.00E-07 | | 2.04E-21 | | 1.00E-06 | | 1.46E-20 | | 1.00E-05 | 1.00E-19 | 1.00E-04 | 6.04E-19 |
| 1100 | | | | 1.00E-07 | | 2.78E-21 | | 1.00E-06 | | 2.02E-20 | | 1.00E-05 | 1.40E-19 | 1.00E-04 | 8.41E-19 |
| 1200 | | | | 1.00E-07 | | 3.38E-21 | | 1.00E-06 | | 2.54E-20 | | 1.00E-05 | 1.88E-19 | 1.00E-04 | 1.35E-18 |
| 1300 | | | | 1.00E-07 | | 4.43E-21 | | 1.00E-06 | | 3.38E-20 | | 1.00E-05 | 2.53E-19 | 1.00E-04 | 1.83E-18 |
| 1400 | | | | 1.00E-07 | | 5.22E-21 | | 1.00E-06 | | 4.07E-20 | | 1.00E-05 | 3.12E-19 | 1.00E-04 | 2.36E-18 |
| 1500 | | | | 1.00E-07 | | 6.06E-21 | | 1.00E-06 | | 4.83E-20 | | 1.00E-05 | 3.78E-19 | 1.00E-04 | 2.91E-18 |
| 1600 | | | | 1.00E-07 | | 6.79E-21 | | 1.00E-06 | | 5.56E-20 | | 1.00E-05 | 4.45E-19 | 1.00E-04 | 3.50E-18 |
| 1700 | | | | 1.00E-07 | | 7.63E-21 | | 1.00E-06 | | 6.37E-20 | | 1.00E-05 | 5.20E-19 | 1.00E-04 | 4.16E-18 |
| 1800 | | | | 1.00E-07 | | 9.40E-21 | | 1.00E-06 | | 7.89E-20 | | 1.00E-05 | 6.48E-19 | 1.00E-04 | 5.22E-18 |
| 1900 | | | | 1.00E-07 | | 1.04E-20 | | 1.00E-06 | | 8.86E-20 | | 1.00E-05 | 7.40E-19 | 1.00E-04 | 6.06E-18 |
| 2000 | | | | 1.00E-07 | | 1.14E-20 | | 1.00E-06 | | 9.85E-20 | | 1.00E-05 | 8.34E-19 | 1.00E-04 | 6.93E-18 |
| 2100 | | | | 1.00E-07 | | 1.19E-20 | | 1.00E-06 | | 1.05E-19 | | 1.00E-05 | 9.07E-19 | 1.00E-04 | 7.69E-18 |
| 2200 | | | | 1.00E-07 | | 1.27E-20 | | 1.00E-06 | | 1.14E-19 | | 1.00E-05 | 1.00E-18 | 1.00E-04 | 8.59E-18 |
| 2300 | | | | 1.00E-07 | | 1.52E-20 | | 1.00E-06 | | 1.36E-19 | | 1.00E-05 | 1.19E-18 | 1.00E-04 | 1.03E-17 |
| 2400 | | | | 1.00E-07 | | 1.62E-20 | | 1.00E-06 | | 1.46E-19 | | 1.00E-05 | 1.30E-18 | 1.00E-04 | 1.13E-17 |
| 2500 | | | | 1.00E-07 | | 1.74E-20 | | 1.00E-06 | | 1.58E-19 | | 1.00E-05 | 1.41E-18 | 1.00E-04 | 1.24E-17 |
| 2600 | | | | 1.00E-07 | | 1.41E-20 | | 1.00E-06 | | 1.41E-19 | | 1.00E-05 | 1.41E-18 | 1.00E-04 | 1.29E-17 |
| 2700 | | | | 1.00E-07 | | 1.43E-20 | | 1.00E-06 | | 1.43E-19 | | 1.00E-05 | 1.43E-18 | 1.00E-04 | 1.38E-17 |
| 2800 | | | | 1.00E-07 | | 1.83E-20 | | 1.00E-06 | | 1.83E-19 | | 1.00E-05 | 1.80E-18 | 1.00E-04 | 1.63E-17 |
| 2900 | | | | 1.00E-07 | | 1.86E-20 | | 1.00E-06 | | 1.86E-19 | | 1.00E-05 | 1.86E-18 | 1.00E-04 | 1.73E-17 |
| 3000 | | | | 1.00E-07 | | 1.90E-20 | | 1.00E-06 | | 1.90E-19 | | 1.00E-05 | 1.90E-18 | 1.00E-04 | 1.84E-17 |
| T/K | | P/bar | | k (cm^3^ mol^−1^ s^−1^) | | P/bar | | k (cm^3^ mol^−1^ s^−1^) | | P/bar | | k (cm^3^ mol^−1^ s^−1^) | P/bar | k (cm^3^ mol^−1^ s^−1^) |  |
| 300 | | 1.00$E$-03 | | 3.36E-21 | | 1.00E-02 | | 4.71E-21 | | 1.00E-01 | | 5.67E-21 | 1.00E+00 | 6.06E-21 |  |
| 400 | | 1.00$E$-03 | | 4.65E-20 | | 1.00E-02 | | 1.14E-19 | | 1.00E-01 | | 2.23E-19 | 1.00E+00 | 3.22E-19 |  |
| 500 | | 1.00$E$-03 | | 2.27E-19 | | 1.00E-02 | | 8.33E-19 | | 1.00E-01 | | 2.48E-18 | 1.00E+00 | 5.18E-18 |  |
| 600 | | 1.00$E$-03 | | 6.43E-19 | | 1.00E-02 | | 2.99E-18 | | 1.00E-01 | | 1.18E-17 | 1.00E+00 | 3.36E-17 |  |
| 700 | | 1.00$E$-03 | | 1.29E-18 | | 1.00E-02 | | 7.13E-18 | | 1.00E-01 | | 3.40E-17 | 1.00E+00 | 1.22E-16 |  |
| 800 | | 1.00$E$-03 | | 2.33E-18 | | 1.00E-02 | | 1.41E-17 | | 1.00E-01 | | 7.53E-17 | 1.00E+00 | 3.15E-16 |  |
| 900 | | 1.00$E$-03 | | 3.20E-18 | | 1.00E-02 | | 2.23E-17 | | 1.00E-01 | | 1.35E-16 | 1.00E+00 | 6.45E-16 |  |
| 1000 | | 1.00$E$-03 | | 3.93E-18 | | 1.00E-02 | | 2.90E-17 | | 1.00E-01 | | 2.01E-16 | 1.00E+00 | 1.11E-15 |  |
| 1100 | | 1.00$E$-03 | | 5.60E-18 | | 1.00E-02 | | 4.28E-17 | | 1.00E-01 | | 3.09E-16 | 1.00E+00 | 1.82E-15 |  |
| 1200 | | 1.00$E$-03 | | 8.76E-18 | | 1.00E-02 | | 5.37E-17 | | 1.00E-01 | | 3.81E-16 | 1.00E+00 | 2.50E-15 |  |
| 1300 | | 1.00$E$-03 | | 1.18E-17 | | 1.00E-02 | | 7.29E-17 | | 1.00E-01 | | 5.32E-16 | 1.00E+00 | 3.63E-15 |  |
| 1400 | | 1.00$E$-03 | | 1.74E-17 | | 1.00E-02 | | 1.19E-16 | | 1.00E-01 | | 6.92E-16 | 1.00E+00 | 4.40E-15 |  |
| 1500 | | 1.00$E$-03 | | 2.21E-17 | | 1.00E-02 | | 1.64E-16 | | 1.00E-01 | | 1.07E-15 | 1.00E+00 | 5.72E-15 |  |
| 1600 | | 1.00$E$-03 | | 2.71E-17 | | 1.00E-02 | | 2.04E-16 | | 1.00E-01 | | 1.34E-15 | 1.00E+00 | 7.08E-15 |  |
| 1700 | | 1.00$E$-03 | | 3.28E-17 | | 1.00E-02 | | 2.53E-16 | | 1.00E-01 | | 1.81E-15 | 1.00E+00 | 1.02E-14 |  |
| 1800 | | 1.00$E$-03 | | 4.15E-17 | | 1.00E-02 | | 3.22E-16 | | 1.00E-01 | | 2.32E-15 | 1.00E+00 | 1.29E-14 |  |
| 1900 | | 1.00$E$-03 | | 4.88E-17 | | 1.00E-02 | | 3.84E-16 | | 1.00E-01 | | 2.84E-15 | 1.00E+00 | 1.80E-14 |  |
| 2000 | | 1.00$E$-03 | | 5.66E-17 | | 1.00E-02 | | 4.52E-16 | | 1.00E-01 | | 3.40E-15 | 1.00E+00 | 2.24E-14 |  |
| 2100 | | 1.00$E$-03 | | 6.40E-17 | | 1.00E-02 | | 5.20E-16 | | 1.00E-01 | | 3.98E-15 | 1.00E+00 | 2.67E-14 |  |
| 2200 | | 1.00$E$-03 | | 7.24E-17 | | 1.00E-02 | | 5.96E-16 | | 1.00E-01 | | 4.64E-15 | 1.00E+00 | 3.19E-14 |  |
| 2300 | | 1.00$E$-03 | | 8.68E-17 | | 1.00E-02 | | 7.18E-16 | | 1.00E-01 | | 5.60E-15 | 1.00E+00 | 3.86E-14 |  |
| 2400 | | 1.00$E$-03 | | 9.69E-17 | | 1.00E-02 | | 8.11E-16 | | 1.00E-01 | | 6.41E-15 | 1.00E+00 | 4.50E-14 |  |
| 2500 | | 1.00$E$-03 | | 1.08E-16 | | 1.00E-02 | | 9.12E-16 | | 1.00E-01 | | 7.31E-15 | 1.00E+00 | 5.21E-14 |  |
| 2600 | | 1.00$E$-03 | | 1.14E-16 | | 1.00E-02 | | 9.79E-16 | | 1.00E-01 | | 8.01E-15 | 1.00E+00 | 5.84E-14 |  |
| 2700 | | 1.00$E$-03 | | 1.23E-16 | | 1.00E-02 | | 1.07E-15 | | 1.00E-01 | | 8.89E-15 | 1.00E+00 | 6.58E-14 |  |
| 2800 | | 1.00$E$-03 | | 1.45E-16 | | 1.00E-02 | | 1.26E-15 | | 1.00E-01 | | 1.05E-14 | 1.00E+00 | 7.79E-14 |  |
| 2900 | | 1.00$E$-03 | | 1.55E-16 | | 1.00E-02 | | 1.37E-15 | | 1.00E-01 | | 1.15E-14 | 1.00E+00 | 8.68E-14 |  |
| 3000 | | 1.00$E$-03 | | 1.67E-16 | | 1.00E-02 | | 1.48E-15 | | 1.00E-01 | | 1.26E-14 | 1.00E+00 | 9.64E-14 |  |
| T/K | | P/bar | | k (cm^3^ mol^−1^ s^−1^) | | P/bar | | k (L mol^−1^ s ^−1^) | | P/bar | | k (L mol^−1^ s ^−1^) | P/bar | k (L mol^−1^ s ^−1^) |  |
| 300 | | 1.00E+01 | | 6.14E-21 | | 1.00E+02 | | 6.15E-21 | | 1.00E+03 | | 6.15E-21 | 1.00E+04 | 6.15E-21 |  |
| 400 | | 1.00E+01 | | 3.63E-19 | | 1.00E+02 | | 3.70E-19 | | 1.00E+03 | | 3.71E-19 | 1.00E+04 | 3.71E-19 |  |
| 500 | | 1.00E+01 | | 7.20E-18 | | 1.00E+02 | | 7.78E-18 | | 1.00E+03 | | 7.86E-18 | 1.00E+04 | 7.87E-18 |  |
| 600 | | 1.00E+01 | | 6.06E-17 | | 1.00E+02 | | 7.31E-17 | | 1.00E+03 | | 7.54E-17 | 1.00E+04 | 7.57E-17 |  |
| 700 | | 1.00E+01 | | 2.82E-16 | | 1.00E+02 | | 3.98E-16 | | 1.00E+03 | | 4.27E-16 | 1.00E+04 | 4.30E-16 |  |
| 800 | | 1.00E+01 | | 8.86E-16 | | 1.00E+02 | | 1.48E-15 | | 1.00E+03 | | 1.69E-15 | 1.00E+04 | 1.72E-15 |  |
| 900 | | 1.00E+01 | | 2.12E-15 | | 1.00E+02 | | 4.18E-15 | | 1.00E+03 | | 5.16E-15 | 1.00E+04 | 5.33E-15 |  |
| 1000 | | 1.00E+01 | | 4.22E-15 | | 1.00E+02 | | 9.64E-15 | | 1.00E+03 | | 1.30E-14 | 1.00E+04 | 1.37E-14 |  |
| 1100 | | 1.00E+01 | | 7.52E-15 | | 1.00E+02 | | 1.92E-14 | | 1.00E+03 | | 2.84E-14 | 1.00E+04 | 3.08E-14 |  |
| 1200 | | 1.00E+01 | | 1.19E-14 | | 1.00E+02 | | 3.41E-14 | | 1.00E+03 | | 5.53E-14 | 1.00E+04 | 6.16E-14 |  |
| 1300 | | 1.00E+01 | | 1.82E-14 | | 1.00E+02 | | 5.61E-14 | | 1.00E+03 | | 9.84E-14 | 1.00E+04 | 1.13E-13 |  |
| 1400 | | 1.00E+01 | | 2.46E-14 | | 1.00E+02 | | 8.54E-14 | | 1.00E+03 | | 1.63E-13 | 1.00E+04 | 1.94E-13 |  |
| 1500 | | 1.00E+01 | | 3.15E-14 | | 1.00E+02 | | 1.22E-13 | | 1.00E+03 | | 2.53E-13 | 1.00E+04 | 3.13E-13 |  |
| 1600 | | 1.00E+01 | | 4.11E-14 | | 1.00E+02 | | 1.72E-13 | | 1.00E+03 | | 3.76E-13 | 1.00E+04 | 4.82E-13 |  |
| 1700 | | 1.00E+01 | | 5.02E-14 | | 1.00E+02 | | 2.24E-13 | | 1.00E+03 | | 5.35E-13 | 1.00E+04 | 7.13E-13 |  |
| 1800 | | 1.00E+01 | | 6.44E-14 | | 1.00E+02 | | 2.99E-13 | | 1.00E+03 | | 7.42E-13 | 1.00E+04 | 1.02E-12 |  |
| 1900 | | 1.00E+01 | | 8.18E-14 | | 1.00E+02 | | 3.67E-13 | | 1.00E+03 | | 9.89E-13 | 1.00E+04 | 1.41E-12 |  |
| 2000 | | 1.00E+01 | | 1.08E-13 | | 1.00E+02 | | 4.44E-13 | | 1.00E+03 | | 1.28E-12 | 1.00E+04 | 1.89E-12 |  |
| 2100 | | 1.00E+01 | | 1.27E-13 | | 1.00E+02 | | 5.42E-13 | | 1.00E+03 | | 1.66E-12 | 1.00E+04 | 2.51E-12 |  |
| 2200 | | 1.00E+01 | | 1.68E-13 | | 1.00E+02 | | 6.46E-13 | | 1.00E+03 | | 2.05E-12 | 1.00E+04 | 3.24E-12 |  |
| 2300 | | 1.00E+01 | | 2.02E-13 | | 1.00E+02 | | 7.82E-13 | | 1.00E+03 | | 2.56E-12 | 1.00E+04 | 4.10E-12 |  |
| 2400 | | 1.00E+01 | | 2.51E-13 | | 1.00E+02 | | 9.36E-13 | | 1.00E+03 | | 3.08E-12 | 1.00E+04 | 5.13E-12 |  |
| 2500 | | 1.00E+01 | | 3.02E-13 | | 1.00E+02 | | 1.14E-12 | | 1.00E+03 | | 3.67E-12 | 1.00E+04 | 6.34E-12 |  |
| 2600 | | 1.00E+01 | | 3.45E-13 | | 1.00E+02 | | 1.30E-12 | | 1.00E+03 | | 4.36E-12 | 1.00E+04 | 7.74E-12 |  |
| 2700 | | 1.00E+01 | | 3.99E-13 | | 1.00E+02 | | 1.58E-12 | | 1.00E+03 | | 5.03E-12 | 1.00E+04 | 9.28E-12 |  |
| 2800 | | 1.00E+01 | | 4.72E-13 | | 1.00E+02 | | 1.83E-12 | | 1.00E+03 | | 5.99E-12 | 1.00E+04 | 1.12E-11 |  |
| 2900 | | 1.00E+01 | | 5.35E-13 | | 1.00E+02 | | 2.21E-12 | | 1.00E+03 | | 6.81E-12 | 1.00E+04 | 1.31E-11 |  |
| 3000 | | 1.00E+01 | | 6.05E-13 | | 1.00E+02 | | 2.63E-12 | | 1.00E+03 | | 7.76E-12 | 1.00E+04 | 1.53E-11 |  |

**Table S18**. Calculated pressure-dependent rate constants for the H atom transfer of the Cα center in the ethanol plus NH reaction at the CBS-QB3 method.

| T/K | P/bar | k (cm^3^ mol^−1^ s^−1^) | P/bar | k (cm^3^ mol^−1^ s^−1^) | P/bar | k (cm^3^ mol^−1^ s^−1^) | P/bar | k (cm^3^ mol^−1^ s^−1^) |
| --- | --- | --- | --- | --- | --- | --- | --- | --- |
| 300 | 1.00E-07 | 1.05E-21 | 1.00E-06 | 4.09E-21 | 1.00E-05 | 1.33E-20 | 1.00E-04 | 3.57E-20 |
| 400 | 1.00E-07 | 8.31E-22 | 1.00E-06 | 4.64E-21 | 1.00E-05 | 2.41E-20 | 1.00E-04 | 1.04E-19 |
| 500 | 1.00E-07 | 1.03E-21 | 1.00E-06 | 5.64E-21 | 1.00E-05 | 2.89E-20 | 1.00E-04 | 1.68E-19 |
| 600 | 1.00E-07 | 1.47E-21 | 1.00E-06 | 8.29E-21 | 1.00E-05 | 4.45E-20 | 1.00E-04 | 2.87E-19 |
| 700 | 1.00E-07 | 1.76E-21 | 1.00E-06 | 1.14E-20 | 1.00E-05 | 7.15E-20 | 1.00E-04 | 4.12E-19 |
| 800 | 1.00E-07 | 2.50E-21 | 1.00E-06 | 1.66E-20 | 1.00E-05 | 1.07E-19 | 1.00E-04 | 6.18E-19 |
| 900 | 1.00E-07 | 2.95E-21 | 1.00E-06 | 2.07E-20 | 1.00E-05 | 1.42E-19 | 1.00E-04 | 9.53E-19 |
| 1000 | 1.00E-07 | 3.45E-21 | 1.00E-06 | 2.53E-20 | 1.00E-05 | 1.81E-19 | 1.00E-04 | 1.26E-18 |
| 1100 | 1.00E-07 | 4.08E-21 | 1.00E-06 | 3.12E-20 | 1.00E-05 | 2.31E-19 | 1.00E-04 | 1.67E-18 |
| 1200 | 1.00E-07 | 4.59E-21 | 1.00E-06 | 3.64E-20 | 1.00E-05 | 2.79E-19 | 1.00E-04 | 2.08E-18 |
| 1300 | 1.00E-07 | 5.23E-21 | 1.00E-06 | 4.28E-20 | 1.00E-05 | 3.38E-19 | 1.00E-04 | 2.59E-18 |
| 1400 | 1.00E-07 | 5.72E-21 | 1.00E-06 | 4.80E-20 | 1.00E-05 | 3.89E-19 | 1.00E-04 | 3.07E-18 |
| 1500 | 1.00E-07 | 6.27E-21 | 1.00E-06 | 5.35E-20 | 1.00E-05 | 4.44E-19 | 1.00E-04 | 3.58E-18 |
| 1600 | 1.00E-07 | 6.57E-21 | 1.00E-06 | 5.92E-20 | 1.00E-05 | 5.03E-19 | 1.00E-04 | 4.16E-18 |
| 1700 | 1.00E-07 | 6.88E-21 | 1.00E-06 | 6.44E-20 | 1.00E-05 | 5.55E-19 | 1.00E-04 | 4.68E-18 |
| 1800 | 1.00E-07 | 9.85E-21 | 1.00E-06 | 8.44E-20 | 1.00E-05 | 7.19E-19 | 1.00E-04 | 6.03E-18 |
| 1900 | 1.00E-07 | 1.07E-20 | 1.00E-06 | 9.18E-20 | 1.00E-05 | 7.88E-19 | 1.00E-04 | 6.70E-18 |
| 2000 | 1.00E-07 | 6.14E-21 | 1.00E-06 | 6.14E-20 | 1.00E-05 | 6.14E-19 | 1.00E-04 | 6.14E-18 |
| 2100 | 1.00E-07 | 9.24E-21 | 1.00E-06 | 9.24E-20 | 1.00E-05 | 9.13E-19 | 1.00E-04 | 7.93E-18 |
| 2200 | 1.00E-07 | 9.61E-21 | 1.00E-06 | 9.61E-20 | 1.00E-05 | 9.61E-19 | 1.00E-04 | 8.58E-18 |
| 2300 | 1.00E-07 | 7.16E-21 | 1.00E-06 | 7.16E-20 | 1.00E-05 | 7.16E-19 | 1.00E-04 | 7.16E-18 |
| 2400 | 1.00E-07 | 7.24E-21 | 1.00E-06 | 7.24E-20 | 1.00E-05 | 7.24E-19 | 1.00E-04 | 7.24E-18 |
| 2500 | 1.00E-07 | 7.35E-21 | 1.00E-06 | 7.35E-20 | 1.00E-05 | 7.35E-19 | 1.00E-04 | 7.35E-18 |
| 2600 | 1.00E-07 | 5.03E-21 | 1.00E-06 | 5.03E-20 | 1.00E-05 | 5.03E-19 | 1.00E-04 | 5.03E-18 |
| 2700 | 1.00E-07 | 4.96E-21 | 1.00E-06 | 4.96E-20 | 1.00E-05 | 4.96E-19 | 1.00E-04 | 4.96E-18 |
| 2800 | 1.00E-07 | 3.24E-21 | 1.00E-06 | 3.24E-20 | 1.00E-05 | 3.24E-19 | 1.00E-04 | 3.24E-18 |
| 2900 | 1.00E-07 | 3.10E-21 | 1.00E-06 | 3.10E-20 | 1.00E-05 | 3.10E-19 | 1.00E-04 | 3.10E-18 |
| 3000 | 1.00E-07 | 2.98E-21 | 1.00E-06 | 2.98E-20 | 1.00E-05 | 2.98E-19 | 1.00E-04 | 2.98E-18 |
| T/K | P/bar | k (cm^3^ mol^−1^ s^−1^) | P/bar | k (cm^3^ mol^−1^ s^−1^) | P/bar | k (cm^3^ mol^−1^ s^−1^) | P/bar | k (cm^3^ mol^−1^ s^−1^) |
| 300 | 1.00$E$-03 | 7.70E-20 | 1.00E-02 | 1.31E-19 | 1.00E-01 | 1.76E-19 | 1.00E+00 | 1.97E-19 |
| 400 | 1.00$E$-03 | 3.84E-19 | 1.00E-02 | 1.18E-18 | 1.00E-01 | 2.83E-18 | 1.00E+00 | 4.73E-18 |
| 500 | 1.00$E$-03 | 9.16E-19 | 1.00E-02 | 4.22E-18 | 1.00E-01 | 1.55E-17 | 1.00E+00 | 4.00E-17 |
| 600 | 1.00$E$-03 | 1.81E-18 | 1.00E-02 | 1.00E-17 | 1.00E-01 | 4.62E-17 | 1.00E+00 | 1.59E-16 |
| 700 | 1.00$E$-03 | 2.36E-18 | 1.00E-02 | 1.54E-17 | 1.00E-01 | 9.08E-17 | 1.00E+00 | 4.01E-16 |
| 800 | 1.00$E$-03 | 3.66E-18 | 1.00E-02 | 2.56E-17 | 1.00E-01 | 1.65E-16 | 1.00E+00 | 8.20E-16 |
| 900 | 1.00$E$-03 | 6.17E-18 | 1.00E-02 | 3.51E-17 | 1.00E-01 | 2.17E-16 | 1.00E+00 | 1.29E-15 |
| 1000 | 1.00$E$-03 | 8.71E-18 | 1.00E-02 | 5.84E-17 | 1.00E-01 | 3.41E-16 | 1.00E+00 | 1.78E-15 |
| 1100 | 1.00$E$-03 | 1.18E-17 | 1.00E-02 | 8.12E-17 | 1.00E-01 | 4.74E-16 | 1.00E+00 | 2.55E-15 |
| 1200 | 1.00$E$-03 | 1.52E-17 | 1.00E-02 | 1.08E-16 | 1.00E-01 | 7.19E-16 | 1.00E+00 | 3.83E-15 |
| 1300 | 1.00$E$-03 | 1.94E-17 | 1.00E-02 | 1.41E-16 | 1.00E-01 | 9.60E-16 | 1.00E+00 | 5.06E-15 |
| 1400 | 1.00$E$-03 | 2.36E-17 | 1.00E-02 | 1.76E-16 | 1.00E-01 | 1.24E-15 | 1.00E+00 | 7.52E-15 |
| 1500 | 1.00$E$-03 | 2.82E-17 | 1.00E-02 | 2.16E-16 | 1.00E-01 | 1.55E-15 | 1.00E+00 | 9.80E-15 |
| 1600 | 1.00$E$-03 | 3.35E-17 | 1.00E-02 | 2.62E-16 | 1.00E-01 | 1.92E-15 | 1.00E+00 | 1.24E-14 |
| 1700 | 1.00$E$-03 | 3.85E-17 | 1.00E-02 | 3.07E-16 | 1.00E-01 | 2.31E-15 | 1.00E+00 | 1.53E-14 |
| 1800 | 1.00$E$-03 | 4.96E-17 | 1.00E-02 | 3.97E-16 | 1.00E-01 | 2.98E-15 | 1.00E+00 | 1.97E-14 |
| 1900 | 1.00$E$-03 | 5.60E-17 | 1.00E-02 | 4.56E-16 | 1.00E-01 | 3.50E-15 | 1.00E+00 | 2.37E-14 |
| 2000 | 1.00$E$-03 | 5.39E-17 | 1.00E-02 | 4.56E-16 | 1.00E-01 | 3.65E-15 | 1.00E+00 | 2.60E-14 |
| 2100 | 1.00$E$-03 | 6.83E-17 | 1.00E-02 | 5.76E-16 | 1.00E-01 | 4.61E-15 | 1.00E+00 | 3.26E-14 |
| 2200 | 1.00$E$-03 | 7.46E-17 | 1.00E-02 | 6.38E-16 | 1.00E-01 | 5.19E-15 | 1.00E+00 | 3.76E-14 |
| 2300 | 1.00$E$-03 | 7.16E-17 | 1.00E-02 | 6.90E-16 | 1.00E-01 | 5.73E-15 | 1.00E+00 | 4.26E-14 |
| 2400 | 1.00$E$-03 | 7.24E-17 | 1.00E-02 | 7.24E-16 | 1.00E-01 | 6.30E-15 | 1.00E+00 | 4.79E-14 |
| 2500 | 1.00$E$-03 | 7.35E-17 | 1.00E-02 | 7.35E-16 | 1.00E-01 | 6.88E-15 | 1.00E+00 | 5.34E-14 |
| 2600 | 1.00$E$-03 | 5.03E-17 | 1.00E-02 | 5.03E-16 | 1.00E-01 | 5.03E-15 | 1.00E+00 | 5.03E-14 |
| 2700 | 1.00$E$-03 | 4.96E-17 | 1.00E-02 | 4.96E-16 | 1.00E-01 | 4.96E-15 | 1.00E+00 | 4.96E-14 |
| 2800 | 1.00$E$-03 | 3.24E-17 | 1.00E-02 | 3.24E-16 | 1.00E-01 | 3.24E-15 | 1.00E+00 | 3.24E-14 |
| 2900 | 1.00$E$-03 | 3.10E-17 | 1.00E-02 | 3.10E-16 | 1.00E-01 | 3.10E-15 | 1.00E+00 | 3.10E-14 |
| 3000 | 1.00$E$-03 | 2.98E-17 | 1.00E-02 | 2.98E-16 | 1.00E-01 | 2.98E-15 | 1.00E+00 | 2.98E-14 |
| T/K | P/bar | k (cm^3^ mol^−1^ s^−1^) | P/bar | k (L mol^−1^ s ^−1^) | P/bar | k (L mol^−1^ s ^−1^) | P/bar | k (L mol^−1^ s ^−1^) |
| 300 | 1.00E+01 | 2.01E-19 | 1.00E+02 | 2.02E-19 | 1.00E+03 | 2.02E-19 | 1.00E+04 | 2.02E-19 |
| 400 | 1.00E+01 | 5.67E-18 | 1.00E+02 | 5.86E-18 | 1.00E+03 | 5.88E-18 | 1.00E+04 | 5.88E-18 |
| 500 | 1.00E+01 | 6.48E-17 | 1.00E+02 | 7.39E-17 | 1.00E+03 | 7.53E-17 | 1.00E+04 | 7.55E-17 |
| 600 | 1.00E+01 | 3.52E-16 | 1.00E+02 | 4.79E-16 | 1.00E+03 | 5.08E-16 | 1.00E+04 | 5.12E-16 |
| 700 | 1.00E+01 | 1.15E-15 | 1.00E+02 | 1.94E-15 | 1.00E+03 | 2.22E-15 | 1.00E+04 | 2.26E-15 |
| 800 | 1.00E+01 | 2.78E-15 | 1.00E+02 | 5.67E-15 | 1.00E+03 | 7.16E-15 | 1.00E+04 | 7.43E-15 |
| 900 | 1.00E+01 | 5.35E-15 | 1.00E+02 | 1.30E-14 | 1.00E+03 | 1.85E-14 | 1.00E+04 | 1.98E-14 |
| 1000 | 1.00E+01 | 8.61E-15 | 1.00E+02 | 2.53E-14 | 1.00E+03 | 4.06E-14 | 1.00E+04 | 4.50E-14 |
| 1100 | 1.00E+01 | 1.35E-14 | 1.00E+02 | 4.42E-14 | 1.00E+03 | 7.85E-14 | 1.00E+04 | 9.08E-14 |
| 1200 | 1.00E+01 | 1.80E-14 | 1.00E+02 | 6.86E-14 | 1.00E+03 | 1.37E-13 | 1.00E+04 | 1.67E-13 |
| 1300 | 1.00E+01 | 2.47E-14 | 1.00E+02 | 1.03E-13 | 1.00E+03 | 2.24E-13 | 1.00E+04 | 2.85E-13 |
| 1400 | 1.00E+01 | 3.32E-14 | 1.00E+02 | 1.39E-13 | 1.00E+03 | 3.40E-13 | 1.00E+04 | 4.56E-13 |
| 1500 | 1.00E+01 | 4.77E-14 | 1.00E+02 | 1.80E-13 | 1.00E+03 | 4.90E-13 | 1.00E+04 | 6.95E-13 |
| 1600 | 1.00E+01 | 6.02E-14 | 1.00E+02 | 2.35E-13 | 1.00E+03 | 6.90E-13 | 1.00E+04 | 1.02E-12 |
| 1700 | 1.00E+01 | 8.18E-14 | 1.00E+02 | 2.99E-13 | 1.00E+03 | 9.16E-13 | 1.00E+04 | 1.43E-12 |
| 1800 | 1.00E+01 | 1.05E-13 | 1.00E+02 | 3.82E-13 | 1.00E+03 | 1.22E-12 | 1.00E+04 | 1.96E-12 |
| 1900 | 1.00E+01 | 1.31E-13 | 1.00E+02 | 4.90E-13 | 1.00E+03 | 1.54E-12 | 1.00E+04 | 2.62E-12 |
| 2000 | 1.00E+01 | 1.52E-13 | 1.00E+02 | 6.20E-13 | 1.00E+03 | 1.86E-12 | 1.00E+04 | 3.40E-12 |
| 2100 | 1.00E+01 | 1.89E-13 | 1.00E+02 | 7.56E-13 | 1.00E+03 | 2.34E-12 | 1.00E+04 | 4.36E-12 |
| 2200 | 1.00E+01 | 2.23E-13 | 1.00E+02 | 9.57E-13 | 1.00E+03 | 2.80E-12 | 1.00E+04 | 5.47E-12 |
| 2300 | 1.00E+01 | 2.59E-13 | 1.00E+02 | 1.12E-12 | 1.00E+03 | 3.33E-12 | 1.00E+04 | 6.79E-12 |
| 2400 | 1.00E+01 | 2.99E-13 | 1.00E+02 | 1.37E-12 | 1.00E+03 | 3.93E-12 | 1.00E+04 | 8.26E-12 |
| 2500 | 1.00E+01 | 3.41E-13 | 1.00E+02 | 1.62E-12 | 1.00E+03 | 4.66E-12 | 1.00E+04 | 9.92E-12 |
| 2600 | 1.00E+01 | 3.83E-13 | 1.00E+02 | 1.86E-12 | 1.00E+03 | 5.34E-12 | 1.00E+04 | 1.19E-11 |
| 2700 | 1.00E+01 | 4.29E-13 | 1.00E+02 | 2.14E-12 | 1.00E+03 | 6.30E-12 | 1.00E+04 | 1.40E-11 |
| 2800 | 1.00E+01 | 3.24E-13 | 1.00E+02 | 2.42E-12 | 1.00E+03 | 7.09E-12 | 1.00E+04 | 1.64E-11 |
| 2900 | 1.00E+01 | 3.10E-13 | 1.00E+02 | 2.74E-12 | 1.00E+03 | 8.36E-12 | 1.00E+04 | 1.89E-11 |
| 3000 | 1.00E+01 | 2.98E-13 | 1.00E+02 | 2.98E-12 | 1.00E+03 | 9.81E-12 | 1.00E+04 | 2.16E-11 |

**Table S19**. Calculated pressure-dependent rate constants for the H atom transfer of the Cα center in the n-propanol plus NH reaction at the CBS-QB3 method.

| T/K | P/bar | k (cm^3^ mol^−1^ s^−1^) | P/bar | k (cm^3^ mol^−1^ s^−1^) | P/bar | k (cm^3^ mol^−1^ s^−1^) | P/bar | k (cm^3^ mol^−1^ s^−1^) |
| --- | --- | --- | --- | --- | --- | --- | --- | --- |
| 300 | 1.00E-07 | 1.02E-21 | 1.00E-06 | 4.57E-21 | 1.00E-05 | 1.69E-20 | 1.00E-04 | 5.02E-20 |
| 400 | 1.00E-07 | 8.65E-22 | 1.00E-06 | 5.60E-21 | 1.00E-05 | 3.31E-20 | 1.00E-04 | 1.61E-19 |
| 500 | 1.00E-07 | 1.16E-21 | 1.00E-06 | 7.42E-21 | 1.00E-05 | 4.22E-20 | 1.00E-04 | 2.43E-19 |
| 600 | 1.00E-07 | 1.77E-21 | 1.00E-06 | 1.16E-20 | 1.00E-05 | 6.71E-20 | 1.00E-04 | 4.16E-19 |
| 700 | 1.00E-07 | 2.20E-21 | 1.00E-06 | 1.56E-20 | 1.00E-05 | 1.05E-19 | 1.00E-04 | 6.83E-19 |
| 800 | 1.00E-07 | 3.24E-21 | 1.00E-06 | 2.34E-20 | 1.00E-05 | 1.62E-19 | 1.00E-04 | 1.07E-18 |
| 900 | 1.00E-07 | 3.50E-21 | 1.00E-06 | 2.68E-20 | 1.00E-05 | 1.96E-19 | 1.00E-04 | 1.39E-18 |
| 1000 | 1.00E-07 | 4.11E-21 | 1.00E-06 | 3.27E-20 | 1.00E-05 | 2.48E-19 | 1.00E-04 | 1.82E-18 |
| 1100 | 1.00E-07 | 5.05E-21 | 1.00E-06 | 4.12E-20 | 1.00E-05 | 3.21E-19 | 1.00E-04 | 2.42E-18 |
| 1200 | 1.00E-07 | 5.72E-21 | 1.00E-06 | 4.79E-20 | 1.00E-05 | 3.85E-19 | 1.00E-04 | 2.98E-18 |
| 1300 | 1.00E-07 | 6.78E-21 | 1.00E-06 | 5.79E-20 | 1.00E-05 | 4.75E-19 | 1.00E-04 | 3.76E-18 |
| 1400 | 1.00E-07 | 7.50E-21 | 1.00E-06 | 6.51E-20 | 1.00E-05 | 5.45E-19 | 1.00E-04 | 4.42E-18 |
| 1500 | 1.00E-07 | 8.33E-21 | 1.00E-06 | 7.29E-20 | 1.00E-05 | 6.20E-19 | 1.00E-04 | 5.13E-18 |
| 1600 | 1.00E-07 | 9.54E-21 | 1.00E-06 | 8.37E-20 | 1.00E-05 | 7.20E-19 | 1.00E-04 | 6.04E-18 |
| 1700 | 1.00E-07 | 5.46E-21 | 1.00E-06 | 5.46E-20 | 1.00E-05 | 5.46E-19 | 1.00E-04 | 5.47E-18 |
| 1800 | 1.00E-07 | 4.59E-21 | 1.00E-06 | 4.59E-20 | 1.00E-05 | 4.59E-19 | 1.00E-04 | 4.59E-18 |
| 1900 | 1.00E-07 | 4.51E-21 | 1.00E-06 | 4.51E-20 | 1.00E-05 | 4.51E-19 | 1.00E-04 | 4.51E-18 |
| 2000 | 1.00E-07 | 4.51E-21 | 1.00E-06 | 4.51E-20 | 1.00E-05 | 4.51E-19 | 1.00E-04 | 4.51E-18 |
| 2100 | 1.00E-07 | 3.41E-21 | 1.00E-06 | 3.41E-20 | 1.00E-05 | 3.41E-19 | 1.00E-04 | 3.41E-18 |
| 2200 | 1.00E-07 | 3.31E-21 | 1.00E-06 | 3.31E-20 | 1.00E-05 | 3.31E-19 | 1.00E-04 | 3.31E-18 |
| 2300 | 1.00E-07 | 2.36E-21 | 1.00E-06 | 2.36E-20 | 1.00E-05 | 2.36E-19 | 1.00E-04 | 2.36E-18 |
| 2400 | 1.00E-07 | 2.22E-21 | 1.00E-06 | 2.22E-20 | 1.00E-05 | 2.22E-19 | 1.00E-04 | 2.22E-18 |
| 2500 | 1.00E-07 | 2.11E-21 | 1.00E-06 | 2.11E-20 | 1.00E-05 | 2.11E-19 | 1.00E-04 | 2.11E-18 |
| 2600 | 1.00E-07 | 1.36E-21 | 1.00E-06 | 1.36E-20 | 1.00E-05 | 1.36E-19 | 1.00E-04 | 1.36E-18 |
| 2700 | 1.00E-07 | 1.26E-21 | 1.00E-06 | 1.26E-20 | 1.00E-05 | 1.26E-19 | 1.00E-04 | 1.26E-18 |
| 2800 | 1.00E-07 | 7.67E-22 | 1.00E-06 | 7.67E-21 | 1.00E-05 | 7.67E-20 | 1.00E-04 | 7.67E-19 |
| 2900 | 1.00E-07 | 6.85E-22 | 1.00E-06 | 6.85E-21 | 1.00E-05 | 6.85E-20 | 1.00E-04 | 6.85E-19 |
| 3000 | 1.00E-07 | 6.17E-22 | 1.00E-06 | 6.17E-21 | 1.00E-05 | 6.17E-20 | 1.00E-04 | 6.17E-19 |
| T/K | P/bar | k (cm^3^ mol^−1^ s^−1^) | P/bar | k (cm^3^ mol^−1^ s^−1^) | P/bar | k (cm^3^ mol^−1^ s^−1^) | P/bar | k (cm^3^ mol^−1^ s^−1^) |
| 300 | 1.00$E$-03 | 1.14E-19 | 1.00E-02 | 1.95E-19 | 1.00E-01 | 2.51E-19 | 1.00E+00 | 2.69E-19 |
| 400 | 1.00$E$-03 | 6.41E-19 | 1.00E-02 | 2.02E-18 | 1.00E-01 | 4.54E-18 | 1.00E+00 | 6.74E-18 |
| 500 | 1.00$E$-03 | 1.43E-18 | 1.00E-02 | 7.02E-18 | 1.00E-01 | 2.51E-17 | 1.00E+00 | 5.77E-17 |
| 600 | 1.00$E$-03 | 2.78E-18 | 1.00E-02 | 1.62E-17 | 1.00E-01 | 7.35E-17 | 1.00E+00 | 2.30E-16 |
| 700 | 1.00$E$-03 | 3.97E-18 | 1.00E-02 | 2.32E-17 | 1.00E-01 | 1.35E-16 | 1.00E+00 | 5.70E-16 |
| 800 | 1.00$E$-03 | 6.22E-18 | 1.00E-02 | 3.81E-17 | 1.00E-01 | 2.42E-16 | 1.00E+00 | 1.16E-15 |
| 900 | 1.00$E$-03 | 9.53E-18 | 1.00E-02 | 6.09E-17 | 1.00E-01 | 3.21E-16 | 1.00E+00 | 1.70E-15 |
| 1000 | 1.00$E$-03 | 1.29E-17 | 1.00E-02 | 8.74E-17 | 1.00E-01 | 5.29E-16 | 1.00E+00 | 2.50E-15 |
| 1100 | 1.00$E$-03 | 1.76E-17 | 1.00E-02 | 1.22E-16 | 1.00E-01 | 7.54E-16 | 1.00E+00 | 3.56E-15 |
| 1200 | 1.00$E$-03 | 2.23E-17 | 1.00E-02 | 1.59E-16 | 1.00E-01 | 1.03E-15 | 1.00E+00 | 5.54E-15 |
| 1300 | 1.00$E$-03 | 2.87E-17 | 1.00E-02 | 2.09E-16 | 1.00E-01 | 1.38E-15 | 1.00E+00 | 7.47E-15 |
| 1400 | 1.00$E$-03 | 3.47E-17 | 1.00E-02 | 2.59E-16 | 1.00E-01 | 1.75E-15 | 1.00E+00 | 1.02E-14 |
| 1500 | 1.00$E$-03 | 4.11E-17 | 1.00E-02 | 3.14E-16 | 1.00E-01 | 2.18E-15 | 1.00E+00 | 1.30E-14 |
| 1600 | 1.00$E$-03 | 4.93E-17 | 1.00E-02 | 3.84E-16 | 1.00E-01 | 2.72E-15 | 1.00E+00 | 1.66E-14 |
| 1700 | 1.00$E$-03 | 4.85E-17 | 1.00E-02 | 3.95E-16 | 1.00E-01 | 2.95E-15 | 1.00E+00 | 1.90E-14 |
| 1800 | 1.00$E$-03 | 4.59E-17 | 1.00E-02 | 4.59E-16 | 1.00E-01 | 3.54E-15 | 1.00E+00 | 2.32E-14 |
| 1900 | 1.00$E$-03 | 4.51E-17 | 1.00E-02 | 4.51E-16 | 1.00E-01 | 4.03E-15 | 1.00E+00 | 2.73E-14 |
| 2000 | 1.00$E$-03 | 4.51E-17 | 1.00E-02 | 4.51E-16 | 1.00E-01 | 4.51E-15 | 1.00E+00 | 3.16E-14 |
| 2100 | 1.00$E$-03 | 3.41E-17 | 1.00E-02 | 3.41E-16 | 1.00E-01 | 3.41E-15 | 1.00E+00 | 3.41E-14 |
| 2200 | 1.00$E$-03 | 3.31E-17 | 1.00E-02 | 3.31E-16 | 1.00E-01 | 3.31E-15 | 1.00E+00 | 3.31E-14 |
| 2300 | 1.00$E$-03 | 2.36E-17 | 1.00E-02 | 2.36E-16 | 1.00E-01 | 2.36E-15 | 1.00E+00 | 2.36E-14 |
| 2400 | 1.00$E$-03 | 2.22E-17 | 1.00E-02 | 2.22E-16 | 1.00E-01 | 2.22E-15 | 1.00E+00 | 2.22E-14 |
| 2500 | 1.00$E$-03 | 2.11E-17 | 1.00E-02 | 2.11E-16 | 1.00E-01 | 2.11E-15 | 1.00E+00 | 2.12E-14 |
| 2600 | 1.00$E$-03 | 1.36E-17 | 1.00E-02 | 1.36E-16 | 1.00E-01 | 1.36E-15 | 1.00E+00 | 1.36E-14 |
| 2700 | 1.00$E$-03 | 1.26E-17 | 1.00E-02 | 1.26E-16 | 1.00E-01 | 1.26E-15 | 1.00E+00 | 1.26E-14 |
| 2800 | 1.00$E$-03 | 7.67E-18 | 1.00E-02 | 7.67E-17 | 1.00E-01 | 7.67E-16 | 1.00E+00 | 7.68E-15 |
| 2900 | 1.00$E$-03 | 6.85E-18 | 1.00E-02 | 6.85E-17 | 1.00E-01 | 6.85E-16 | 1.00E+00 | 6.85E-15 |
| 3000 | 1.00$E$-03 | 6.17E-18 | 1.00E-02 | 6.17E-17 | 1.00E-01 | 6.17E-16 | 1.00E+00 | 6.17E-15 |
| T/K | P/bar | k (cm^3^ mol^−1^ s^−1^) | P/bar | k (cm^3^ mol^−1^ s ^−1^) | P/bar | k (cm^3^ mol^−1^ s ^−1^) | P/bar | k (cm^3^ mol^−1^ s ^−1^) |
| 300 | 1.00E+01 | 2.72E-19 | 1.00E+02 | 2.72E-19 | 1.00E+03 | 2.72E-19 | 1.00E+04 | 2.72E-19 |
| 400 | 1.00E+01 | 7.48E-18 | 1.00E+02 | 7.59E-18 | 1.00E+03 | 7.60E-18 | 1.00E+04 | 7.60E-18 |
| 500 | 1.00E+01 | 8.16E-17 | 1.00E+02 | 8.79E-17 | 1.00E+03 | 8.87E-17 | 1.00E+04 | 8.88E-17 |
| 600 | 1.00E+01 | 4.40E-16 | 1.00E+02 | 5.39E-16 | 1.00E+03 | 5.56E-16 | 1.00E+04 | 5.58E-16 |
| 700 | 1.00E+01 | 1.44E-15 | 1.00E+02 | 2.13E-15 | 1.00E+03 | 2.30E-15 | 1.00E+04 | 2.33E-15 |
| 800 | 1.00E+01 | 3.49E-15 | 1.00E+02 | 6.17E-15 | 1.00E+03 | 7.17E-15 | 1.00E+04 | 7.32E-15 |
| 900 | 1.00E+01 | 6.66E-15 | 1.00E+02 | 1.42E-14 | 1.00E+03 | 1.81E-14 | 1.00E+04 | 1.88E-14 |
| 1000 | 1.00E+01 | 1.06E-14 | 1.00E+02 | 2.76E-14 | 1.00E+03 | 3.92E-14 | 1.00E+04 | 4.18E-14 |
| 1100 | 1.00E+01 | 1.66E-14 | 1.00E+02 | 4.86E-14 | 1.00E+03 | 7.55E-14 | 1.00E+04 | 8.26E-14 |
| 1200 | 1.00E+01 | 2.25E-14 | 1.00E+02 | 7.57E-14 | 1.00E+03 | 1.32E-13 | 1.00E+04 | 1.49E-13 |
| 1300 | 1.00E+01 | 3.09E-14 | 1.00E+02 | 1.15E-13 | 1.00E+03 | 2.15E-13 | 1.00E+04 | 2.52E-13 |
| 1400 | 1.00E+01 | 4.30E-14 | 1.00E+02 | 1.55E-13 | 1.00E+03 | 3.28E-13 | 1.00E+04 | 4.00E-13 |
| 1500 | 1.00E+01 | 6.09E-14 | 1.00E+02 | 2.03E-13 | 1.00E+03 | 4.74E-13 | 1.00E+04 | 6.05E-13 |
| 1600 | 1.00E+01 | 7.79E-14 | 1.00E+02 | 2.67E-13 | 1.00E+03 | 6.69E-13 | 1.00E+04 | 8.79E-13 |
| 1700 | 1.00E+01 | 9.78E-14 | 1.00E+02 | 3.32E-13 | 1.00E+03 | 8.91E-13 | 1.00E+04 | 1.23E-12 |
| 1800 | 1.00E+01 | 1.22E-13 | 1.00E+02 | 4.11E-13 | 1.00E+03 | 1.19E-12 | 1.00E+04 | 1.69E-12 |
| 1900 | 1.00E+01 | 1.48E-13 | 1.00E+02 | 5.30E-13 | 1.00E+03 | 1.49E-12 | 1.00E+04 | 2.24E-12 |
| 2000 | 1.00E+01 | 1.77E-13 | 1.00E+02 | 6.85E-13 | 1.00E+03 | 1.84E-12 | 1.00E+04 | 2.91E-12 |
| 2100 | 1.00E+01 | 2.10E-13 | 1.00E+02 | 8.15E-13 | 1.00E+03 | 2.28E-12 | 1.00E+04 | 3.72E-12 |
| 2200 | 1.00E+01 | 2.44E-13 | 1.00E+02 | 1.02E-12 | 1.00E+03 | 2.72E-12 | 1.00E+04 | 4.66E-12 |
| 2300 | 1.00E+01 | 2.36E-13 | 1.00E+02 | 1.20E-12 | 1.00E+03 | 3.25E-12 | 1.00E+04 | 5.77E-12 |
| 2400 | 1.00E+01 | 2.22E-13 | 1.00E+02 | 1.44E-12 | 1.00E+03 | 3.81E-12 | 1.00E+04 | 7.01E-12 |
| 2500 | 1.00E+01 | 2.12E-13 | 1.00E+02 | 1.68E-12 | 1.00E+03 | 4.50E-12 | 1.00E+04 | 8.38E-12 |
| 2600 | 1.00E+01 | 1.36E-13 | 1.00E+02 | 1.36E-12 | 1.00E+03 | 5.16E-12 | 1.00E+04 | 9.96E-12 |
| 2700 | 1.00E+01 | 1.26E-13 | 1.00E+02 | 1.26E-12 | 1.00E+03 | 6.07E-12 | 1.00E+04 | 1.16E-11 |
| 2800 | 1.00E+01 | 7.68E-14 | 1.00E+02 | 7.68E-13 | 1.00E+03 | 6.83E-12 | 1.00E+04 | 1.34E-11 |
| 2900 | 1.00E+01 | 6.85E-14 | 1.00E+02 | 6.85E-13 | 1.00E+03 | 6.86E-12 | 1.00E+04 | 1.52E-11 |
| 3000 | 1.00E+01 | 6.17E-14 | 1.00E+02 | 6.18E-13 | 1.00E+03 | 6.18E-12 | 1.00E+04 | 1.71E-11 |

**Table S20**. Calculated pressure-dependent rate constants for the H atom transfer of the Cα center in the n-butanol plus NH reaction at the CBS-QB3 method.

| T/K | P/bar | k (cm^3^ mol^−1^ s^−1^) | P/bar | k (cm^3^ mol^−1^ s^−1^) | P/bar | k (cm^3^ mol^−1^ s^−1^) | P/bar | k (cm^3^ mol^−1^ s^−1^) |
| --- | --- | --- | --- | --- | --- | --- | --- | --- |
| 300 | 1.00E-07 | 7.04E-22 | 1.00E-06 | 3.48E-21 | 1.00E-05 | 1.39E-20 | 1.00E-04 | 4.41E-20 |
| 400 | 1.00E-07 | 7.05E-22 | 1.00E-06 | 4.02E-21 | 1.00E-05 | 2.45E-20 | 1.00E-04 | 1.34E-19 |
| 500 | 1.00E-07 | 9.44E-22 | 1.00E-06 | 6.20E-21 | 1.00E-05 | 3.81E-20 | 1.00E-04 | 2.12E-19 |
| 600 | 1.00E-07 | 1.54E-21 | 1.00E-06 | 1.04E-20 | 1.00E-05 | 6.68E-20 | 1.00E-04 | 3.82E-19 |
| 700 | 1.00E-07 | 1.97E-21 | 1.00E-06 | 1.41E-20 | 1.00E-05 | 9.63E-20 | 1.00E-04 | 6.33E-19 |
| 800 | 1.00E-07 | 2.42E-21 | 1.00E-06 | 1.83E-20 | 1.00E-05 | 1.31E-19 | 1.00E-04 | 9.07E-19 |
| 900 | 1.00E-07 | 2.86E-21 | 1.00E-06 | 2.26E-20 | 1.00E-05 | 1.69E-19 | 1.00E-04 | 1.22E-18 |
| 1000 | 1.00E-07 | 3.34E-21 | 1.00E-06 | 2.74E-20 | 1.00E-05 | 2.13E-19 | 1.00E-04 | 1.59E-18 |
| 1100 | 1.00E-07 | 4.27E-21 | 1.00E-06 | 3.56E-20 | 1.00E-05 | 2.83E-19 | 1.00E-04 | 2.16E-18 |
| 1200 | 1.00E-07 | 4.86E-21 | 1.00E-06 | 4.13E-20 | 1.00E-05 | 3.37E-19 | 1.00E-04 | 2.65E-18 |
| 1300 | 1.00E-07 | 4.91E-21 | 1.00E-06 | 4.30E-20 | 1.00E-05 | 3.64E-19 | 1.00E-04 | 2.98E-18 |
| 1400 | 1.00E-07 | 4.98E-21 | 1.00E-06 | 4.77E-20 | 1.00E-05 | 4.09E-19 | 1.00E-04 | 3.41E-18 |
| 1500 | 1.00E-07 | 5.20E-21 | 1.00E-06 | 5.20E-20 | 1.00E-05 | 4.58E-19 | 1.00E-04 | 3.87E-18 |
| 1600 | 1.00E-07 | 4.84E-21 | 1.00E-06 | 4.84E-20 | 1.00E-05 | 4.84E-19 | 1.00E-04 | 4.70E-18 |
| 1700 | 1.00E-07 | 4.93E-21 | 1.00E-06 | 4.93E-20 | 1.00E-05 | 4.93E-19 | 1.00E-04 | 4.93E-18 |
| 1800 | 1.00E-07 | 1.76E-21 | 1.00E-06 | 1.76E-20 | 1.00E-05 | 1.76E-19 | 1.00E-04 | 1.76E-18 |
| 1900 | 1.00E-07 | 1.65E-21 | 1.00E-06 | 1.65E-20 | 1.00E-05 | 1.65E-19 | 1.00E-04 | 1.65E-18 |
| 2000 | 1.00E-07 | 1.58E-21 | 1.00E-06 | 1.58E-20 | 1.00E-05 | 1.58E-19 | 1.00E-04 | 1.58E-18 |
| 2100 | 1.00E-07 | 1.21E-21 | 1.00E-06 | 1.21E-20 | 1.00E-05 | 1.21E-19 | 1.00E-04 | 1.21E-18 |
| 2200 | 1.00E-07 | 1.13E-21 | 1.00E-06 | 1.13E-20 | 1.00E-05 | 1.13E-19 | 1.00E-04 | 1.13E-18 |
| 2300 | 1.00E-07 | 8.10E-22 | 1.00E-06 | 8.10E-21 | 1.00E-05 | 8.10E-20 | 1.00E-04 | 8.10E-19 |
| 2400 | 1.00E-07 | 7.27E-22 | 1.00E-06 | 7.27E-21 | 1.00E-05 | 7.27E-20 | 1.00E-04 | 7.27E-19 |
| 2500 | 1.00E-07 | 6.66E-22 | 1.00E-06 | 6.66E-21 | 1.00E-05 | 6.66E-20 | 1.00E-04 | 6.66E-19 |
| 2600 | 1.00E-07 | 4.25E-22 | 1.00E-06 | 4.25E-21 | 1.00E-05 | 4.25E-20 | 1.00E-04 | 4.25E-19 |
| 2700 | 1.00E-07 | 1.12E-22 | 1.00E-06 | 1.12E-21 | 1.00E-05 | 1.12E-20 | 1.00E-04 | 1.12E-19 |
| 2800 | 1.00E-07 | 6.68E-23 | 1.00E-06 | 6.68E-22 | 1.00E-05 | 6.68E-21 | 1.00E-04 | 6.68E-20 |
| 2900 | 1.00E-07 | 5.44E-23 | 1.00E-06 | 5.44E-22 | 1.00E-05 | 5.44E-21 | 1.00E-04 | 5.44E-20 |
| 3000 | 1.00E-07 | 4.51E-23 | 1.00E-06 | 4.50E-22 | 1.00E-05 | 4.51E-21 | 1.00E-04 | 4.51E-20 |
| T/K | P/bar | k (cm^3^ mol^−1^ s^−1^) | P/bar | k (cm^3^ mol^−1^ s^−1^) | P/bar | k (cm^3^ mol^−1^ s^−1^) | P/bar | k (cm^3^ mol^−1^ s^−1^) |
| 300 | 1.00$E$-03 | 1.07E-19 | 1.00E-02 | 1.90E-19 | 1.00E-01 | 2.47E-19 | 1.00E+00 | 2.65E-19 |
| 400 | 1.00$E$-03 | 5.85E-19 | 1.00E-02 | 1.96E-18 | 1.00E-01 | 4.61E-18 | 1.00E+00 | 6.99E-18 |
| 500 | 1.00$E$-03 | 1.15E-18 | 1.00E-02 | 6.13E-18 | 1.00E-01 | 2.41E-17 | 1.00E+00 | 5.90E-17 |
| 600 | 1.00$E$-03 | 2.27E-18 | 1.00E-02 | 1.41E-17 | 1.00E-01 | 6.92E-17 | 1.00E+00 | 2.30E-16 |
| 700 | 1.00$E$-03 | 4.00E-18 | 1.00E-02 | 2.24E-17 | 1.00E-01 | 1.18E-16 | 1.00E+00 | 5.39E-16 |
| 800 | 1.00$E$-03 | 6.01E-18 | 1.00E-02 | 3.40E-17 | 1.00E-01 | 1.94E-16 | 1.00E+00 | 1.05E-15 |
| 900 | 1.00$E$-03 | 8.51E-18 | 1.00E-02 | 5.60E-17 | 1.00E-01 | 3.21E-16 | 1.00E+00 | 1.51E-15 |
| 1000 | 1.00$E$-03 | 1.15E-17 | 1.00E-02 | 7.86E-17 | 1.00E-01 | 4.86E-16 | 1.00E+00 | 2.47E-15 |
| 1100 | 1.00$E$-03 | 1.59E-17 | 1.00E-02 | 1.11E-16 | 1.00E-01 | 7.04E-16 | 1.00E+00 | 3.60E-15 |
| 1200 | 1.00$E$-03 | 2.01E-17 | 1.00E-02 | 1.45E-16 | 1.00E-01 | 9.44E-16 | 1.00E+00 | 5.28E-15 |
| 1300 | 1.00$E$-03 | 2.35E-17 | 1.00E-02 | 1.75E-16 | 1.00E-01 | 1.19E-15 | 1.00E+00 | 6.95E-15 |
| 1400 | 1.00$E$-03 | 2.76E-17 | 1.00E-02 | 2.12E-16 | 1.00E-01 | 1.49E-15 | 1.00E+00 | 8.98E-15 |
| 1500 | 1.00$E$-03 | 3.19E-17 | 1.00E-02 | 2.52E-16 | 1.00E-01 | 1.81E-15 | 1.00E+00 | 1.13E-14 |
| 1600 | 1.00$E$-03 | 3.90E-17 | 1.00E-02 | 3.11E-16 | 1.00E-01 | 2.28E-15 | 1.00E+00 | 1.44E-14 |
| 1700 | 1.00$E$-03 | 4.38E-17 | 1.00E-02 | 3.56E-16 | 1.00E-01 | 2.67E-15 | 1.00E+00 | 1.74E-14 |
| 1800 | 1.00$E$-03 | 1.76E-17 | 1.00E-02 | 1.76E-16 | 1.00E-01 | 1.76E-15 | 1.00E+00 | 1.76E-14 |
| 1900 | 1.00$E$-03 | 1.65E-17 | 1.00E-02 | 1.65E-16 | 1.00E-01 | 1.65E-15 | 1.00E+00 | 1.65E-14 |
| 2000 | 1.00$E$-03 | 1.58E-17 | 1.00E-02 | 1.58E-16 | 1.00E-01 | 1.58E-15 | 1.00E+00 | 1.58E-14 |
| 2100 | 1.00$E$-03 | 1.21E-17 | 1.00E-02 | 1.21E-16 | 1.00E-01 | 1.21E-15 | 1.00E+00 | 1.22E-14 |
| 2200 | 1.00$E$-03 | 1.13E-17 | 1.00E-02 | 1.13E-16 | 1.00E-01 | 1.13E-15 | 1.00E+00 | 1.13E-14 |
| 2300 | 1.00$E$-03 | 8.10E-18 | 1.00E-02 | 8.10E-17 | 1.00E-01 | 8.10E-16 | 1.00E+00 | 8.11E-15 |
| 2400 | 1.00$E$-03 | 7.27E-18 | 1.00E-02 | 7.27E-17 | 1.00E-01 | 7.28E-16 | 1.00E+00 | 7.28E-15 |
| 2500 | 1.00$E$-03 | 6.66E-18 | 1.00E-02 | 6.66E-17 | 1.00E-01 | 6.66E-16 | 1.00E+00 | 6.67E-15 |
| 2600 | 1.00$E$-03 | 4.25E-18 | 1.00E-02 | 4.25E-17 | 1.00E-01 | 4.25E-16 | 1.00E+00 | 4.25E-15 |
| 2700 | 1.00$E$-03 | 1.12E-18 | 1.00E-02 | 1.12E-17 | 1.00E-01 | 1.12E-16 | 1.00E+00 | 1.12E-15 |
| 2800 | 1.00$E$-03 | 6.68E-19 | 1.00E-02 | 6.68E-18 | 1.00E-01 | 6.68E-17 | 1.00E+00 | 6.69E-16 |
| 2900 | 1.00$E$-03 | 5.44E-19 | 1.00E-02 | 5.44E-18 | 1.00E-01 | 5.44E-17 | 1.00E+00 | 5.44E-16 |
| 3000 | 1.00$E$-03 | 4.51E-19 | 1.00E-02 | 4.51E-18 | 1.00E-01 | 4.51E-17 | 1.00E+00 | 4.50E-16 |
| T/K | P/bar | k (cm^3^ mol^−1^ s^−1^) | P/bar | k (cm^3^ mol^−1^ s ^−1^) | P/bar | k (cm^3^ mol^−1^ s ^−1^) | P/bar | k (cm^3^ mol^−1^ s ^−1^) |
| 300 | 1.00E+01 | 2.67E-19 | 1.00E+02 | 2.67E-19 | 1.00E+03 | 2.67E-19 | 1.00E+04 | 2.67E-19 |
| 400 | 1.00E+01 | 7.79E-18 | 1.00E+02 | 7.91E-18 | 1.00E+03 | 7.92E-18 | 1.00E+04 | 7.93E-18 |
| 500 | 1.00E+01 | 8.64E-17 | 1.00E+02 | 9.38E-17 | 1.00E+03 | 9.48E-17 | 1.00E+04 | 9.49E-17 |
| 600 | 1.00E+01 | 4.61E-16 | 1.00E+02 | 5.81E-16 | 1.00E+03 | 6.03E-16 | 1.00E+04 | 6.05E-16 |
| 700 | 1.00E+01 | 1.48E-15 | 1.00E+02 | 2.29E-15 | 1.00E+03 | 2.52E-15 | 1.00E+04 | 2.55E-15 |
| 800 | 1.00E+01 | 3.51E-15 | 1.00E+02 | 6.59E-15 | 1.00E+03 | 7.88E-15 | 1.00E+04 | 8.08E-15 |
| 900 | 1.00E+01 | 6.40E-15 | 1.00E+02 | 1.50E-14 | 1.00E+03 | 1.99E-14 | 1.00E+04 | 2.09E-14 |
| 1000 | 1.00E+01 | 9.81E-15 | 1.00E+02 | 2.84E-14 | 1.00E+03 | 4.30E-14 | 1.00E+04 | 4.65E-14 |
| 1100 | 1.00E+01 | 1.51E-14 | 1.00E+02 | 4.96E-14 | 1.00E+03 | 8.25E-14 | 1.00E+04 | 9.23E-14 |
| 1200 | 1.00E+01 | 2.19E-14 | 1.00E+02 | 7.49E-14 | 1.00E+03 | 1.43E-13 | 1.00E+04 | 1.67E-13 |
| 1300 | 1.00E+01 | 2.89E-14 | 1.00E+02 | 1.11E-13 | 1.00E+03 | 2.32E-13 | 1.00E+04 | 2.82E-13 |
| 1400 | 1.00E+01 | 4.25E-14 | 1.00E+02 | 1.46E-13 | 1.00E+03 | 3.51E-13 | 1.00E+04 | 4.48E-13 |
| 1500 | 1.00E+01 | 5.70E-14 | 1.00E+02 | 1.95E-13 | 1.00E+03 | 5.00E-13 | 1.00E+04 | 6.77E-13 |
| 1600 | 1.00E+01 | 7.40E-14 | 1.00E+02 | 2.53E-13 | 1.00E+03 | 7.04E-13 | 1.00E+04 | 9.85E-13 |
| 1700 | 1.00E+01 | 9.25E-14 | 1.00E+02 | 3.39E-13 | 1.00E+03 | 9.25E-13 | 1.00E+04 | 1.38E-12 |
| 1800 | 1.00E+01 | 1.10E-13 | 1.00E+02 | 4.07E-13 | 1.00E+03 | 1.21E-12 | 1.00E+04 | 1.88E-12 |
| 1900 | 1.00E+01 | 1.30E-13 | 1.00E+02 | 5.38E-13 | 1.00E+03 | 1.49E-12 | 1.00E+04 | 2.50E-12 |
| 2000 | 1.00E+01 | 1.53E-13 | 1.00E+02 | 6.75E-13 | 1.00E+03 | 1.82E-12 | 1.00E+04 | 3.23E-12 |
| 2100 | 1.00E+01 | 1.22E-13 | 1.00E+02 | 8.16E-13 | 1.00E+03 | 2.24E-12 | 1.00E+04 | 4.12E-12 |
| 2200 | 1.00E+01 | 1.13E-13 | 1.00E+02 | 9.77E-13 | 1.00E+03 | 2.69E-12 | 1.00E+04 | 5.10E-12 |
| 2300 | 1.00E+01 | 8.12E-14 | 1.00E+02 | 8.14E-13 | 1.00E+03 | 3.18E-12 | 1.00E+04 | 6.26E-12 |
| 2400 | 1.00E+01 | 7.30E-14 | 1.00E+02 | 7.32E-13 | 1.00E+03 | 3.82E-12 | 1.00E+04 | 7.44E-12 |
| 2500 | 1.00E+01 | 6.68E-14 | 1.00E+02 | 6.70E-13 | 1.00E+03 | 4.61E-12 | 1.00E+04 | 8.67E-12 |
| 2600 | 1.00E+01 | 4.26E-14 | 1.00E+02 | 4.27E-13 | 1.00E+03 | 4.29E-12 | 1.00E+04 | 1.01E-11 |
| 2700 | 1.00E+01 | 1.12E-14 | 1.00E+02 | 1.12E-13 | 1.00E+03 | 1.12E-12 | 1.00E+04 | 9.33E-12 |
| 2800 | 1.00E+01 | 6.68E-15 | 1.00E+02 | 6.69E-14 | 1.00E+03 | 6.70E-13 | 1.00E+04 | 5.55E-12 |
| 2900 | 1.00E+01 | 5.45E-15 | 1.00E+02 | 5.45E-14 | 1.00E+03 | 5.46E-13 | 1.00E+04 | 4.44E-12 |
| 3000 | 1.00E+01 | 4.51E-15 | 1.00E+02 | 4.51E-14 | 1.00E+03 | 4.52E-13 | 1.00E+04 | 3.62E-12 |

**Table S21.** Topological parameters such as ring critical point (RCP) and bond critical points and (in atomic unit) for all species of the CH_3_OH + NH reaction calculated at the M06-2X method.

| Species | Bond | Eigenvalues of the Hessian matrix | | | ρ | ∇^2^ ρ |
| --- | --- | --- | --- | --- | --- | --- |
|  |  | λ_1_ | λ_2_ | λ_3_ |  |  |
| CR1 | C1-H1 | -0.7457 | -0.7145 | 0.4778 | 0.2796 | -0.9824 |
|  | C1-H2 | -0.7498 | -0.7184 | 0.4793 | 0.2807 | -0.9889 |
|  | C1-H3 | -0.7691 | -0.7386 | 0.4801 | 0.2847 | -1.0276 |
|  | O1-H4 | -1.8522 | -1.8046 | 1.5581 | 0.3648 | -2.0988 |
|  | N1-O1 | -0.0109 | -0.0067 | 0.0662 | 0.0131 | 0.0485 |
|  | N1-H5 | -1.2159 | -1.2155 | 0.6735 | 0.3333 | -1.7579 |
| CR2 | C1-H1 | -0.7510 | -0.7196 | 0.4785 | 0.2806 | -0.9921 |
|  | C1-H2 | -0.7694 | -0.7385 | 0.4800 | 0.2847 | -1.0278 |
|  | C1-H3 | -0.7509 | -0.7191 | 0.4788 | 0.2810 | -0.9912 |
|  | O1-H4 | -1.8505 | -1.8021 | 1.5570 | 0.3656 | -2.0956 |
|  | O1-H6 | -0.0135 | -0.0075 | 0.0714 | 0.0135 | 0.0504 |
|  | N1-H5 | -1.2270 | -1.2257 | 0.6813 | 0.3336 | -1.7714 |
|  | C1-O1 | -0.4510 | -0.4484 | 0.5160 | 0.2534 | -0.3833 |
| 3TS1 | C1-H1 | -0.7515 | -0.7234 | 0.4816 | 0.2802 | -0.9932 |
|  | C1-H2 | -0.7511 | -0.7228 | 0.4816 | 0.2802 | -0.9924 |
|  | C1-H3 | -0.7165 | -0.6950 | 0.4708 | 0.2718 | -0.9407 |
|  | O1-H4 | -0.2951 | -0.2803 | 0.6756 | 0.1164 | 0.1003 |
|  | N1-H4 | -0.8263 | -0.8180 | 0.6267 | 0.2492 | -1.0176 |
|  | N1-H5 | -1.2129 | -1.1997 | 0.6753 | 0.3335 | -1.7372 |
| 3TS2 | C1-H1 | -0.3071 | -0.2964 | 0.4220 | 0.1414 | -0.1815 |
|  | C1-H2 | -0.7766 | -0.7387 | 0.4746 | 0.2855 | -1.0408 |
|  | C1-H3 | -0.7514 | -0.7144 | 0.4723 | 0.2806 | -0.9935 |
|  | O1-H4 | -1.8694 | -1.8276 | 1.5737 | 0.3653 | -2.1232 |
|  | N1-H1 | -0.4804 | -0.4733 | 0.6470 | 0.1763 | -0.3067 |
|  | N1-H5 | -1.1784 | -1.1613 | 0.6539 | 0.3299 | -1.6859 |
| CP1 | C1-H1 | -0.6967 | -0.6748 | 0.4681 | 0.2663 | -0.9034 |
|  | C1-H2 | -0.7508 | -0.7224 | 0.4782 | 0.2780 | -0.9950 |
|  | C1-H3 | -0.7544 | -0.7266 | 0.4826 | 0.2811 | -0.9984 |
|  | O1-H4 | -0.0176 | -0.0164 | 0.0832 | 0.0153 | 0.0492 |
|  | N1-H4 | -1.2472 | -1.2296 | 0.7067 | 0.3344 | -1.7701 |
|  | N1-H5 | -1.2204 | -1.1976 | 0.6778 | 0.3363 | -1.7403 |
|  | N1-H2 | -0.0074 | -0.0056 | 0.0446 | 0.0087 | 0.0316 |
|  | RCP | -0.0059 | 0.0073 | 0.0358 | 0.0077 | 0.0372 |
| CP2 | C1-H2 | -0.7891 | -0.7310 | 0.4656 | 0.2876 | -1.0545 |
|  | C1-H3 | -0.7620 | -0.7053 | 0.4632 | 0.2823 | -1.0041 |
|  | O1-H4 | -1.8667 | -1.8327 | 1.5934 | 0.3487 | -2.1059 |
|  | N1-H4 | -0.0404 | -0.0398 | 0.1651 | 0.0298 | 0.0849 |
|  | N1-H5 | -1.2511 | -1.2326 | 0.7070 | 0.3370 | -1.7767 |
|  | N1-H1 | -1.2520 | -1.2336 | 0.7077 | 0.3369 | -1.7779 |

**Table S22.** Topological parameters such as ring critical point (RCP) and line critical points (LCP) (in atomic unit) for all species of the C_2_H_5_OH+ NH reaction calculated at the M06-2X method.

| Species | Bond | Eigenvalues of the Hessian matrix | | | ρ | ∇^2^ ρ |
| --- | --- | --- | --- | --- | --- | --- |
|  |  | λ_1_ | λ_2_ | λ_3_ |  |  |
| CR1 | C1-H1 | -0.6937 | -0.6913 | 0.4569 | 0.2737 | -0.9281 |
|  | C1-H2 | -0.6899 | -0.6872 | 0.4562 | 0.2731 | -0.9209 |
|  | C1-H3 | -0.7021 | -0.7000 | 0.4605 | 0.2754 | -0.9417 |
|  | C2-H4 | -0.7383 | -0.7100 | 0.4798 | 0.2787 | -0.9685 |
|  | C2-H5 | -0.7673 | -0.7399 | 0.4827 | 0.2850 | -1.0245 |
|  | O1-H6 | -1.8360 | -1.7891 | 1.5466 | 0.3627 | -2.0785 |
|  | N1-H4 | -0.0071 | -0.0032 | 0.0447 | 0.0090 | 0.0344 |
|  | N1-O1 | -0.0127 | -0.0074 | 0.0741 | 0.0135 | 0.0540 |
| CR2 | C1-H1 | -0.6975 | -0.6933 | 0.4574 | 0.2744 | -0.9334 |
|  | C1-H2 | -0.7044 | -0.7014 | 0.4612 | 0.2757 | -0.9446 |
|  | C1-H3 | -0.7026 | -0.6991 | 0.4592 | 0.2751 | -0.9425 |
|  | C2-H4 | -0.7507 | -0.7214 | 0.4804 | 0.2817 | -0.9917 |
|  | C2-H5 | -0.7509 | -0.7218 | 0.4816 | 0.2812 | -0.9911 |
|  | O1-H6 | -1.8447 | -1.7967 | 1.5535 | 0.3652 | -2.0879 |
|  | N1-H3 | -0.0052 | -0.0042 | 0.0347 | 0.0071 | 0.0253 |
|  | O1-H7 | -0.0202 | -0.0188 | 0.0921 | 0.0176 | 0.0532 |
|  | RCP | -0.0016 | -0.0016 | 0.0211 | 0.0059 | 0.0256 |
| CR3 | C1-H1 | -0.6969 | -0.6926 | 0.4569 | 0.2743 | -0.9326 |
|  | C1-H2 | -0.7036 | -0.7003 | 0.4604 | 0.2753 | -0.9435 |
|  | C1-H3 | -0.7039 | -0.7006 | 0.4604 | 0.2753 | -0.9441 |
|  | C2-H4 | -0.7521 | -0.7224 | 0.4821 | 0.2818 | -0.9925 |
|  | O1-H6 | -1.8487 | -1.8010 | 1.5579 | 0.3652 | -2.0918 |
|  | C1-N1 | -0.0032 | -0.0012 | 0.0287 | 0.0067 | 0.0242 |
|  | O1-H7 | -0.0212 | -0.0194 | 0.0961 | 0.0180 | 0.0556 |
|  | N1-H7 | -1.2424 | -1.2421 | 0.6968 | 0.3329 | -1.7876 |
| 3TS1 | C1-H1 | -0.6958 | -0.6924 | 0.4574 | 0.2740 | -0.9307 |
|  | C1-H2 | -0.6973 | -0.6946 | 0.4575 | 0.2743 | -0.9344 |
|  | C1-H3 | -0.7062 | -0.7035 | 0.4615 | 0.2760 | -0.9482 |
|  | C2-H4 | -0.7352 | -0.7090 | 0.4802 | 0.2776 | -0.9640 |
|  | C2-H5 | -0.7195 | -0.7001 | 0.4746 | 0.2732 | -0.9450 |
|  | C2-O1 | -0.5043 | -0.4851 | 0.6291 | 0.2694 | -0.3602 |
|  | O1-H6 | -0.2966 | -0.2813 | 0.6723 | 0.1171 | 0.0944 |
|  | N1-H6 | -0.8295 | -0.8166 | 0.6204 | 0.2468 | -1.0257 |
| 3TS2 | C1-H1 | -0.7029 | -0.6984 | 0.4594 | 0.2751 | -0.9419 |
|  | C1-H2 | -0.6928 | -0.6905 | 0.4609 | 0.2726 | -0.9225 |
|  | C1-H3 | -0.7024 | -0.6990 | 0.4615 | 0.2748 | -0.9399 |
|  | C2-H4 | -0.7457 | -0.7095 | 0.4734 | 0.2800 | -0.9819 |
|  | C2-H5 | -0.3271 | -0.3168 | 0.4326 | 0.1484 | -0.2113 |
|  | C2-O1 | -0.5001 | -0.4627 | 0.7585 | 0.2666 | -0.2042 |
|  | O1-H6 | -1.8615 | -1.8198 | 1.5683 | 0.3648 | -2.1130 |
|  | N1-H5 | -0.4525 | -0.4455 | 0.6447 | 0.1696 | -0.2532 |
| 3TS3 | C1-H1 | -0.7205 | -0.7013 | 0.4548 | 0.2777 | -0.9669 |
|  | C1-H2 | -0.7220 | -0.7057 | 0.4571 | 0.2780 | -0.9705 |
|  | C1-H3 | -0.2185 | -0.2171 | 0.3638 | 0.1133 | -0.0718 |
|  | C2-H4 | -0.7344 | -0.7058 | 0.4803 | 0.2771 | -0.9599 |
|  | C2-H5 | -0.7501 | -0.7219 | 0.4834 | 0.2809 | -0.9886 |
|  | C2-O1 | -0.4533 | -0.4446 | 0.4886 | 0.2521 | -0.4093 |
|  | O1-H6 | -1.8477 | -1.7995 | 1.5559 | 0.3654 | -2.0913 |
|  | N1-H3 | -0.6304 | -0.6193 | 0.6415 | 0.2120 | -0.6082 |
| CP1 | C1-H1 | -0.6960 | -0.6928 | 0.4577 | 0.2741 | -0.9310 |
|  | C1-H2 | -0.7053 | -0.7027 | 0.4605 | 0.2754 | -0.9475 |
|  | C1-H3 | -0.7003 | -0.6975 | 0.4591 | 0.2752 | -0.9387 |
|  | C2-H4 | -0.7005 | -0.6857 | 0.4697 | 0.2671 | -0.9165 |
|  | C2-H5 | -0.7344 | -0.7069 | 0.4798 | 0.2765 | -0.9615 |
|  | O1-H6 | -0.0143 | -0.0123 | 0.0753 | 0.0135 | 0.0487 |
|  | N1-H2 | -0.0059 | -0.0048 | 0.0358 | 0.0077 | 0.0252 |
|  | N1-H6 | -1.2389 | -1.2208 | 0.6991 | 0.3340 | -1.7606 |
|  | N1-H4 | -0.0068 | -0.0042 | 0.0433 | 0.0089 | 0.0323 |
|  | RCP1 | -0.0053 | 0.0049 | 0.0359 | 0.0084 | 0.0355 |
|  | RCP2 | -0.0035 | 0.0055 | 0.0240 | 0.0067 | 0.0260 |
| CP2 | C1-H1 | -0.6986 | -0.6937 | 0.4583 | 0.2745 | -0.9339 |
|  | C1-H2 | -0.6740 | -0.6710 | 0.4616 | 0.2680 | -0.8834 |
|  | C1-H3 | -0.6976 | -0.6940 | 0.4624 | 0.2736 | -0.9292 |
|  | C2-H4 | -0.7516 | -0.6958 | 0.4636 | 0.2805 | -0.9838 |
|  | C2-O1 | -0.5678 | -0.4821 | 0.9930 | 0.2807 | -0.0570 |
|  | O1-H6 | -1.8670 | -1.8324 | 1.5920 | 0.3497 | -2.1074 |
|  | N1-H5 | -1.2488 | -1.2302 | 0.7049 | 0.3365 | -1.7741 |
|  | N1-H6 | -0.0379 | -0.0373 | 0.1572 | 0.0285 | 0.0819 |
| CP3 | C1-H1 | -0.7492 | -0.7240 | 0.4560 | 0.2833 | -1.0172 |
|  | C1-H2 | -0.7530 | -0.7284 | 0.4581 | 0.2832 | -1.0233 |
|  | C2-H4 | -0.7272 | -0.6987 | 0.4806 | 0.2751 | -0.9453 |
|  | C2-H5 | -0.7484 | -0.7203 | 0.4829 | 0.2796 | -0.9858 |
|  | O1-H7 | -0.0186 | -0.0172 | 0.0875 | 0.0165 | 0.0517 |
|  | O1-H6 | -1.8486 | -1.8006 | 1.5566 | 0.3648 | -2.0926 |
|  | N1-H3 | -1.2082 | -1.1849 | 0.6695 | 0.3344 | -1.7236 |

**Table S23.** Topological parameters such as ring critical point (RCP) and line critical points (LCP) for all species of the n-C_3_H_7_OH+ NH reaction calculated at the M06-2X method.

| Species | Bond | Eigenvalues of the Hessian matrix | | | ρ | ∇^2^ ρ |
| --- | --- | --- | --- | --- | --- | --- |
|  |  | λ_1_ | λ_2_ | λ_3_ |  |  |
| CR1 | C1-H1 | -0.7015 | -0.6978 | 0.4595 | 0.2755 | -0.9398 |
|  | C1-H2 | -0.6939 | -0.6898 | 0.4582 | 0.2739 | -0.9255 |
|  | C1-H3 | -0.6938 | -0.6897 | 0.4582 | 0.2739 | -0.9253 |
|  | C2-H4 | -0.6953 | -0.6937 | 0.4604 | 0.2745 | -0.9286 |
|  | C2-H5 | -0.6922 | -0.6906 | 0.4589 | 0.2740 | -0.9239 |
|  | C3-H6 | -0.7409 | -0.7121 | 0.4797 | 0.2794 | -0.9732 |
|  | C3-H7 | -0.7627 | -0.7345 | 0.4817 | 0.2839 | -1.0156 |
|  | N1-O1 | -0.0100 | -0.0044 | 0.0602 | 0.0121 | 0.0458 |
|  | N1-C2 | -0.0030 | -0.0010 | 0.0249 | 0.0062 | 0.0208 |
|  | C1-C2 | -0.4394 | -0.4392 | 0.3326 | 0.2422 | -0.5460 |
|  | C2-C3 | -0.4852 | -0.4642 | 0.3503 | 0.2526 | -0.5991 |
|  | C3-O1 | -0.4505 | -0.4493 | 0.5173 | 0.2533 | -0.3825 |
| CR2 | C1-H1 | -0.7020 | -0.6980 | 0.4594 | 0.2756 | -0.9406 |
|  | C1-H2 | -0.6923 | -0.6879 | 0.4577 | 0.2736 | -0.9226 |
|  | C1-H3 | -0.6970 | -0.6927 | 0.4591 | 0.2744 | -0.9306 |
|  | C2-H4 | -0.7027 | -0.7009 | 0.4630 | 0.2761 | -0.9406 |
|  | C2-H5 | -0.7007 | -0.7002 | 0.4613 | 0.2756 | -0.9396 |
|  | C3-H6 | -0.7453 | -0.7153 | 0.4797 | 0.2799 | -0.9809 |
|  | C3-H7 | -0.7445 | -0.7146 | 0.4786 | 0.2803 | -0.9805 |
|  | N1-H5 | -0.0057 | -0.0049 | 0.0378 | 0.0077 | 0.0271 |
|  | O1-H8 | -0.0195 | -0.0183 | 0.0899 | 0.0172 | 0.0521 |
|  | O1-H9 | -1.8458 | -1.7978 | 1.5545 | 0.3652 | -2.0891 |
|  | C1-C2 | -0.4411 | -0.4400 | 0.3326 | 0.2426 | -0.5485 |
|  | C2-C3 | -0.4901 | -0.4700 | 0.3465 | 0.2548 | -0.6135 |
|  | C3-O1 | -0.4372 | -0.4312 | 0.5027 | 0.2487 | -0.3657 |
|  | C1-H1 | -0.7040 | -0.7001 | 0.4601 | 0.2759 | -0.9441 |
| CR3 | C1-H2 | -0.6938 | -0.6893 | 0.4581 | 0.2739 | -0.9250 |
|  | C1-H3 | -0.6937 | -0.6892 | 0.4581 | 0.2738 | -0.9248 |
|  | C2-H4 | -0.7025 | -0.7014 | 0.4625 | 0.2758 | -0.9414 |
|  | C2-H5 | -0.7006 | -0.6993 | 0.4623 | 0.2754 | -0.9376 |
|  | C3-H6 | -0.7466 | -0.7160 | 0.4804 | 0.2805 | -0.9822 |
|  | C3-H7 | -0.7465 | -0.7161 | 0.4804 | 0.2805 | -0.9822 |
|  | O1-H8 | -1.8487 | -1.8010 | 1.5580 | 0.3650 | -2.0916 |
|  | C1-C2 | -0.4407 | -0.4397 | 0.3325 | 0.2425 | -0.5479 |
|  | C2-C3 | -0.4898 | -0.4702 | 0.3466 | 0.2547 | -0.6134 |
|  | C3-O1 | -0.4358 | -0.4315 | 0.5174 | 0.2488 | -0.3498 |
|  | N1-H4 | -0.0039 | -0.0022 | 0.0304 | 0.0068 | 0.0243 |
|  | O1-H9 | -0.0211 | -0.0193 | 0.0958 | 0.0180 | 0.0554 |
|  | C1-H1 | -0.7030 | -0.6990 | 0.4588 | 0.2753 | -0.9432 |
| CR4 | C1-H2 | -0.6921 | -0.6875 | 0.4575 | 0.2736 | -0.9221 |
|  | C1-H3 | -0.6942 | -0.6895 | 0.4582 | 0.2738 | -0.9255 |
|  | C2-H4 | -0.6928 | -0.6912 | 0.4597 | 0.2742 | -0.9242 |
|  | C2-H5 | -0.7017 | -0.7007 | 0.4621 | 0.2756 | -0.9402 |
|  | C3-H6 | -0.7411 | -0.7118 | 0.4799 | 0.2795 | -0.9730 |
|  | C3-H7 | -0.7404 | -0.7109 | 0.4799 | 0.2793 | -0.9714 |
|  | O1-H8 | -1.8432 | -1.7951 | 1.5514 | 0.3664 | -2.0869 |
|  | C1-C2 | -0.4411 | -0.4407 | 0.3325 | 0.2428 | -0.5494 |
|  | C2-C3 | -0.4915 | -0.4711 | 0.3477 | 0.2550 | -0.6150 |
|  | C3-O1 | -0.4573 | -0.4521 | 0.5162 | 0.2542 | -0.3932 |
|  | N1-H5 | -0.0049 | -0.0036 | 0.0345 | 0.0073 | 0.0261 |
|  | N1-H1 | -0.0059 | -0.0046 | 0.0349 | 0.0072 | 0.0243 |
|  | RCP | -0.0035 | 0.0044 | 0.0211 | 0.0061 | 0.0220 |
| 3TS1 | C1-H1 | -0.7023 | -0.6984 | 0.4595 | 0.2756 | -0.9411 |
|  | C1-H2 | -0.6941 | -0.6898 | -0.6830 | 0.2739 | -0.9258 |
|  | C1-H3 | -0.6956 | -0.6912 | 0.4587 | 0.2741 | -0.9281 |
|  | C2-H4 | -0.7037 | -0.7019 | 0.4630 | 0.2762 | -0.9425 |
|  | C2-H5 | -0.6910 | -0.6900 | 0.4583 | 0.2738 | -0.9228 |
|  | C3-H6 | -0.7352 | -0.7098 | 0.4801 | 0.2773 | -0.9649 |
|  | C3-H7 | -0.7098 | -0.6902 | 0.4714 | 0.2710 | -0.9287 |
|  | N1-H8 | -1.2198 | -1.2062 | 0.6809 | 0.3340 | -1.7451 |
|  | N1-H9 | -0.2963 | -0.2805 | 0.6726 | 0.1169 | 0.0959 |
|  | O1-H9 | -0.2963 | -0.2805 | 0.6726 | 0.1169 | 0.0959 |
|  | C1-C2 | -0.4400 | -0.4396 | 0.3327 | 0.2424 | -0.5469 |
|  | C2-C3 | -0.4797 | -0.4601 | 0.3492 | 0.2508 | -0.5906 |
|  | C3-O1 | -0.5065 | -0.4895 | 0.6499 | 0.2705 | -0.3462 |
| 3TS2 | C1-H1 | -0.7026 | -0.6979 | 0.4593 | 0.2756 | -0.9411 |
|  | C1-H2 | -0.6954 | -0.6906 | 0.4585 | 0.2741 | -0.9275 |
|  | C1-H3 | -0.7003 | -0.6959 | 0.4595 | 0.2747 | -0.9367 |
|  | C2-H4 | -0.6918 | -0.6897 | 0.4627 | 0.2729 | -0.9187 |
|  | C2-H5 | -0.6993 | -0.6981 | 0.4633 | 0.2750 | -0.9341 |
|  | C3-H6 | -0.3266 | -0.3156 | 0.4308 | 0.1484 | -0.2114 |
|  | C3-H7 | -0.7392 | -0.7029 | 0.4718 | 0.2785 | -0.9704 |
|  | N1-H8 | -1.1707 | -1.1526 | 0.6495 | 0.3289 | -1.6739 |
|  | N1-H9 | -0.4490 | -0.4418 | 0.6430 | 0.1687 | -0.2477 |
|  | C1-C2 | -0.4406 | -0.4388 | 0.3323 | 0.2421 | -0.5471 |
|  | C2-C3 | -0.5008 | -0.4722 | 0.3445 | 0.2591 | -0.6285 |
|  | C3-O1 | -0.5015 | -0.4660 | 0.7685 | 0.2669 | -0.1991 |
| 3TS3 | C1-H1 | -0.7030 | -0.6990 | 0.4588 | 0.2753 | -0.9432 |
|  | C1-H2 | -0.6942 | -0.6895 | 0.4582 | 0.2738 | -0.9255 |
|  | C1-H3 | -0.6921 | -0.6875 | 0.4575 | 0.2736 | -0.9221 |
|  | C2-H4 | -0.7017 | -0.7007 | 0.4621 | 0.2756 | -0.9402 |
|  | C2-H5 | -0.6928 | -0.6912 | 0.4597 | 0.2742 | -0.9242 |
|  | C3-H6 | -0.7404 | -0.7109 | 0.4799 | 0.2793 | -0.9714 |
|  | C3-H7 | -0.7411 | -0.7118 | 0.4799 | 0.2795 | -0.9730 |
|  | N1-H1 | -0.0059 | -0.0046 | 0.0349 | 0.0072 | 0.0243 |
|  | O1-H8 | -1.8432 | -1.7951 | 1.5514 | 0.3664 | -2.0869 |
|  | C1-C2 | -0.4411 | -0.4407 | 0.3325 | 0.2428 | -0.5494 |
|  | C2-C3 | -0.4915 | -0.4711 | 0.3477 | 0.2550 | -0.6150 |
|  | C3-O1 | -0.4573 | -0.4521 | 0.5162 | 0.2542 | -0.3932 |
|  | N1-H4 | -0.0049 | -0.0036 | 0.0345 | 0.0073 | 0.0261 |
| 3TS4 | C1-H1 | -0.2358 | -0.2333 | 0.3756 | 0.1196 | -0.0935 |
|  | C1-H2 | -0.7140 | -0.6973 | 0.4560 | 0.2768 | -0.9553 |
|  | C1-H3 | -0.7157 | -0.6984 | 0.4564 | 0.2769 | -0.9577 |
|  | C2-H4 | -0.7034 | -0.7003 | 0.4639 | 0.2757 | -0.9398 |
|  | C2-H5 | -0.7055 | -0.7026 | 0.4650 | 0.2759 | -0.9430 |
|  | C3-H6 | -0.7454 | -0.7166 | 0.4811 | 0.2803 | -0.9809 |
|  | C3-H7 | -0.7448 | -0.7160 | 0.4811 | 0.2802 | -0.9798 |
|  | N1-H1 | -0.5915 | -0.5829 | 0.6454 | 0.2028 | -0.5290 |
|  | O1-H8 | -1.8440 | -1.7961 | 1.5521 | 0.3659 | -2.0880 |
|  | C1-C2 | -0.4657 | -0.4514 | 0.3339 | 0.2513 | -0.5832 |
|  | C2-C3 | -0.4767 | -0.4562 | 0.3465 | 0.2494 | -0.5864 |
|  | C3-O1 | -0.4634 | -0.4554 | 0.5144 | 0.2555 | -0.4044 |
| CP1 | C1-H1 | -0.7010 | -0.6972 | 0.4592 | 0.2754 | -0.9390 |
|  | C1-H2 | -0.6925 | -0.6882 | 0.4577 | 0.2736 | -0.9230 |
|  | C1-H3 | -0.6972 | -0.6932 | 0.4592 | 0.2744 | -0.9312 |
|  | C2-H4 | -0.6985 | -0.6969 | 0.4611 | 0.2755 | -0.9342 |
|  | C2-H5 | -0.7041 | -0.7027 | 0.4626 | 0.2759 | -0.9442 |
|  | C3-H6 | -0.6931 | -0.6783 | 0.4676 | 0.2654 | -0.9038 |
|  | C3-H7 | -0.7286 | -0.7012 | 0.4787 | 0.2752 | -0.9511 |
|  | N1-H8 | -1.2201 | -1.1989 | 0.6789 | 0.3359 | -1.7401 |
|  | N1-H9 | -1.2371 | -1.2189 | 0.6976 | 0.3338 | -1.7584 |
|  | C1-C2 | -0.4405 | -0.4400 | 0.3329 | 0.2426 | -0.5476 |
|  | C2-C3 | -0.4761 | -0.4564 | 0.3481 | 0.2494 | -0.5843 |
|  | C3-O1 | -0.5523 | -0.5238 | 0.8376 | 0.2830 | -0.2385 |
|  | N1-H6 | -0.0076 | -0.0051 | 0.0469 | 0.0095 | 0.0342 |
|  | N1-H5 | -0.0066 | -0.0054 | 0.0392 | 0.0083 | 0.0271 |
|  | O1-H9 | -0.0686 | 0.0740 | 0.1528 | 0.0605 | 0.1582 |
| CP2 | C1-H1 | -0.7011 | -0.6965 | 0.4602 | 0.2750 | -0.9374 |
|  | C1-H2 | -0.6933 | -0.6888 | 0.4577 | 0.2738 | -0.9244 |
|  | C1-H3 | -0.7047 | -0.7002 | 0.4603 | 0.2760 | -0.9446 |
|  | C2-H4 | -0.6791 | -0.6756 | 0.4629 | 0.2696 | -0.8918 |
|  | C2-H5 | -0.7022 | -0.7006 | 0.4639 | 0.2747 | -0.9389 |
|  | N1-H6 | -1.2123 | -1.1904 | 0.6738 | 0.3338 | -1.7289 |
|  | C3-H7 | -0.7612 | -0.7083 | 0.4646 | 0.2825 | -1.0049 |
|  | N1-H8 | -1.2329 | -1.2130 | 0.6882 | 0.3354 | -1.7577 |
|  | C3-H6 | -0.0056 | -0.0043 | 0.0367 | 0.0085 | 0.0268 |
|  | O1-H8 | -0.0099 | -0.0081 | 0.0536 | 0.0102 | 0.0356 |
|  | C1-C2 | -0.4413 | -0.4377 | 0.3328 | 0.2418 | -0.5461 |
|  | C2-C3 | -0.5190 | -0.4776 | 0.3402 | 0.2656 | -0.6564 |
|  | C3-O1 | -0.5257 | -0.4566 | 0.9526 | 0.2711 | -0.0297 |
|  | N1-H6 | -1.2123 | -1.1904 | 0.6738 | 0.3338 | -1.7289 |
|  | N1-H5 | -0.0062 | -0.0050 | 0.0409 | 0.0085 | 0.0296 |
| CP3 | C1-H1 | -0.7051 | -0.6998 | 0.4617 | 0.2756 | -0.9432 |
|  | C1-H2 | -0.6979 | -0.6930 | 0.4621 | 0.2730 | -0.9288 |
|  | C1-H3 | -0.6713 | -0.6658 | 0.4585 | 0.2672 | -0.8786 |
|  | C2-H4 | -0.0087 | -0.0069 | 0.0479 | 0.0101 | 0.0323 |
|  | C2-H5 | -0.7421 | -0.7152 | 0.4582 | 0.2818 | -0.9990 |
|  | C3-H6 | -0.7216 | -0.6931 | 0.4790 | 0.2742 | -0.9356 |
|  | C3-H7 | -0.7504 | -0.7223 | 0.4824 | 0.2804 | -0.9902 |
|  | N1-H4 | -1.2276 | -1.2075 | 0.6897 | 0.3336 | -1.7453 |
|  | O1-H8 | -1.8405 | -1.7918 | 1.5498 | 0.3655 | -2.0825 |
|  | N1-H7 | -0.0061 | -0.0027 | 0.0402 | 0.0084 | 0.0315 |
|  | C1-C2 | -0.4905 | -0.4633 | 0.3346 | 0.2595 | -0.6192 |
|  | C2-C3 | -0.5329 | -0.4929 | 0.3491 | 0.2695 | -0.6767 |
|  | C3-O1 | -0.4494 | -0.4407 | 0.4710 | 0.2505 | -0.4191 |
|  | N1-O1 | -0.0052 | -0.0034 | 0.0426 | 0.0091 | 0.0340 |
| CP4 | C1-H1 | -0.0104 | -0.0099 | 0.0503 | 0.0113 | 0.0300 |
|  | C1-H2 | -0.7471 | -0.7230 | 0.4581 | 0.2829 | -1.0120 |
|  | C1-H3 | -0.7471 | -0.7229 | 0.4581 | 0.2829 | -1.0119 |
|  | C2-H4 | -0.7042 | -0.7006 | 0.4649 | 0.2754 | -0.9400 |
|  | C2-H5 | -0.7043 | -0.7007 | 0.4649 | 0.2754 | -0.9400 |
|  | C3-H6 | -0.7484 | -0.7195 | 0.4814 | 0.2809 | -0.9865 |
|  | C3-H7 | -0.7483 | -0.7195 | 0.4814 | 0.2809 | -0.9865 |
|  | N1-H1 | -1.2139 | -1.1946 | 0.6819 | 0.3321 | -1.7267 |
|  | O1-H8 | -1.8444 | -1.7966 | 1.5530 | 0.3657 | -2.0880 |
|  | C1-C2 | -0.4894 | -0.4652 | 0.3344 | 0.2591 | -0.6201 |
|  | C2-C3 | -0.4697 | -0.4480 | 0.3464 | 0.2463 | -0.5713 |
|  | C3-O1 | -0.4653 | -0.4562 | 0.5179 | 0.2559 | -0.4035 |

**Table S24.** Topological parameters such as line critical point (LCP) and ring critical point (RCP) and line critical points (LCP) for all species of the n-C_4_H_9_OH + NH reaction calculated at the M06-2X method.

| Species | Bond | Eigenvalues of the Hessian matrix | | | ρ | ∇^2^ ρ |
| --- | --- | --- | --- | --- | --- | --- |
|  |  | λ_1_ | λ_2_ | λ_3_ |  |  |
| CR1 | C1-H1 | -0.6981 | -0.6945 | 0.4585 | 0.2749 | -0.9341 |
|  | C1-H2 | -0.6914 | -0.6879 | 0.4573 | 0.2735 | -0.9219 |
|  | C1-H3 | -0.6914 | -0.6881 | 0.4574 | 0.2735 | -0.9220 |
|  | C2-H4 | -0.6902 | -0.6864 | 0.4590 | 0.2736 | -0.9176 |
|  | C2-H5 | -0.7070 | -0.7047 | 0.4639 | 0.2769 | -0.9478 |
|  | C3-H6 | -0.6792 | -0.6767 | 0.4556 | 0.2712 | -0.9003 |
|  | C3-H7 | -0.6860 | -0.6847 | 0.4572 | 0.2726 | -0.9135 |
|  | C4-H8 | -0.7433 | -0.7148 | 0.4790 | 0.2798 | -0.9792 |
|  | C4-H9 | -0.7596 | -0.7315 | 0.4808 | 0.2832 | -1.0103 |
|  | C1-C2 | -0.4440 | -0.4416 | 0.3342 | 0.2433 | -0.5514 |
|  | C2-C3 | -0.4487 | -0.4444 | 0.3373 | 0.2449 | -0.5558 |
|  | C3-C4 | -0.4823 | -0.4654 | 0.3493 | 0.2523 | -0.5983 |
|  | C4-O1 | -0.4466 | -0.4433 | 0.5082 | 0.2523 | -0.3817 |
|  | O1-H10 | -1.8287 | -1.7826 | 1.5410 | 0.3627 | -2.0703 |
|  | N1-O1 | -0.0105 | -0.0059 | 0.0646 | 0.0128 | 0.0482 |
| CR2 | RCP | -0.0010 | 0.0055 | 0.0225 | 0.0063 | 0.0270 |
| CR3 | C1-H1 | -0.6975 | -0.6931 | 0.4583 | 0.2747 | -0.9324 |
|  | C1-H2 | -0.6887 | -0.6845 | 0.4563 | 0.2730 | -1.0918 |
|  | C1-H3 | -0.7021 | -0.6986 | 0.4598 | 0.2751 | -0.9409 |
|  | C2-H4 | -0.6873 | -0.6839 | 0.4578 | 0.2731 | -0.9134 |
|  | C2-H5 | -0.7031 | -0.7018 | 0.4619 | 0.2761 | -0.9430 |
|  | C3-H6 | -0.6940 | -0.6909 | 0.4598 | 0.2736 | -0.9251 |
|  | C3-H7 | -0.6891 | -0.6857 | 0.4570 | 0.2732 | -0.9178 |
|  | C4-H8 | -0.7519 | -0.7213 | 0.4813 | 0.2817 | -0.9919 |
|  | C4-H9 | -0.7452 | -0.7150 | 0.4801 | 0.2802 | -0.9801 |
|  | N1-H6 | -0.0054 | -0.0043 | 0.0377 | 0.0078 | 0.0280 |
|  | C1-C2 | -0.4441 | -0.4416 | 0.3341 | 0.2434 | -0.5516 |
|  | C2-C3 | -0.4476 | -0.4427 | 0.3367 | 0.2444 | -0.5536 |
|  | C3-C4 | -0.4863 | -0.4709 | 0.3458 | 0.2541 | -0.6114 |
|  | C4-O1 | -0.4292 | -0.4258 | 0.5065 | 0.2473 | -0.3484 |
|  | O1-H10 | -1.8500 | -1.8022 | 1.5596 | 0.3652 | -2.0926 |
|  | O1-H11 | -0.0210 | -0.0193 | 0.0950 | 0.0179 | 0.0548 |
|  | N1-H5 | -0.0047 | -0.0041 | 0.0366 | 0.0076 | 0.0278 |
|  | N1-H3 | -0.0035 | -0.0019 | 0.0225 | 0.0051 | 0.0171 |
|  | Cage | 0.0015 | 0.0065 | 0.0065 | 0.0056 | 0.0241 |
| CR4 | C1-H1 | -0.6995 | -0.6958 | 0.4591 | 0.2751 | -0.9363 |
|  | C1-H2 | -0.6916 | -0.6881 | 0.4575 | 0.2735 | -0.9223 |
|  | C1-H3 | -0.6925 | -0.6890 | 0.4579 | 0.2737 | -0.9236 |
|  | C2-H4 | -0.6934 | -0.6907 | 0.4595 | 0.2740 | -0.9246 |
|  | C2-H5 | -0.6996 | -0.6979 | 0.4611 | 0.2756 | -0.9365 |
|  | C3-H6 | -0.6959 | -0.6938 | 0.4617 | 0.2744 | -0.9280 |
|  | C3-H7 | -0.6881 | -0.6859 | 0.4574 | 0.2730 | -0.9165 |
|  | C4-H8 | -0.7498 | -0.7201 | 0.4791 | 0.2816 | -0.9909 |
|  | C4-H9 | -0.7445 | -0.7146 | 0.4798 | 0.2796 | -0.9793 |
|  | N1-H4 | -0.0056 | -0.0035 | 0.0341 | 0.0073 | 0.0251 |
|  | O1-H11 | -0.0209 | -0.0198 | 0.0934 | 0.0182 | 0.0527 |
|  | C1-C2 | -0.4432 | -0.4409 | 0.3339 | 0.2430 | -0.5502 |
|  | C2-C3 | -0.4478 | -0.4436 | 0.3370 | 0.2446 | -0.5545 |
|  | C3-C4 | -0.4874 | -0.4717 | 0.3466 | 0.2544 | -0.6125 |
| CR5 | C1-H1 | -0.6971 | -0.6937 | 0.4581 | 0.2747 | -0.9326 |
|  | C1-H2 | -0.6935 | -0.6906 | 0.4571 | 0.2736 | -0.9270 |
|  | C1-H3 | -0.6832 | -0.6799 | 0.4545 | 0.2719 | -0.9085 |
|  | C2-H4 | -0.6889 | -0.6850 | 0.4589 | 0.2733 | -0.9150 |
|  | C2-H5 | -0.7115 | -0.7094 | 0.4649 | 0.2777 | -0.9559 |
|  | C3-H6 | -0.6855 | -0.6833 | 0.4583 | 0.2723 | -0.9105 |
|  | C3-H7 | -0.6918 | -0.6904 | 0.4572 | 0.2736 | -0.9250 |
|  | C4-H8 | -0.7464 | -0.7168 | 0.4807 | 0.2807 | -0.9825 |
|  | C4-H9 | -0.7399 | -0.7106 | 0.4797 | 0.2792 | -0.9708 |
|  | N1-H2 | -0.0057 | -0.0047 | 0.0345 | 0.0073 | 0.0242 |
|  | N1-H11 | -1.1955 | -1.1953 | 0.6594 | 0.3321 | -1.7315 |
|  | N1-H7 | -0.0062 | -0.0052 | 0.0369 | 0.0077 | 0.0255 |
|  | C1-C2 | -0.4434 | -0.4411 | 0.3339 | 0.2431 | -0.5506 |
|  | C2-C3 | -0.4486 | -0.4439 | 0.3372 | 0.2448 | -0.5554 |
|  | C3-C4 | -0.4875 | -0.4719 | 0.3472 | 0.2543 | -0.6123 |
| 3TS1 | C1-H1 | -0.6996 | -0.6957 | 0.4590 | 0.2751 | -0.9363 |
|  | C1-H2 | -0.6917 | -0.6879 | 0.4572 | 0.2736 | -0.9223 |
|  | C1-H3 | -0.6944 | -0.6909 | 0.4584 | 0.2740 | -0.9269 |
|  | C2-H4 | -0.6898 | -0.6861 | 0.4591 | 0.2735 | -1.0939 |
|  | C2-H5 | -0.7086 | -0.7069 | 0.4645 | 0.2772 | -0.9510 |
|  | C3-H6 | -0.6857 | -0.6837 | 0.4570 | 0.2723 | -0.9124 |
|  | C3-H7 | -0.6884 | -0.6867 | 0.4575 | 0.2731 | -0.9175 |
|  | C4-H8 | -0.7363 | -0.7098 | 0.4801 | 0.2779 | -0.9661 |
|  | C4-H9 | -0.7121 | -0.6925 | 0.4730 | 0.2714 | -0.9316 |
|  | O1-H10 | -0.3027 | -0.2877 | 0.6785 | 0.1186 | 0.0881 |
|  | N1-H10 | -0.8221 | -0.8098 | 0.6204 | 0.2452 | -1.0114 |
|  | C1-C2 | -0.4436 | -0.4413 | 0.3342 | 0.2432 | -0.5507 |
|  | C2-C3 | -0.4486 | -0.4443 | 0.3370 | 0.2450 | -0.5558 |
|  | C3-C4 | -0.4741 | -0.4577 | 0.3474 | 0.2494 | -0.5844 |
| 3TS3 | C1-H1 | -0.6994 | -0.6947 | 0.4586 | 0.2751 | -0.9356 |
|  | C1-H2 | -0.6926 | -0.6881 | 0.4576 | 0.2736 | -0.9231 |
|  | C1-H3 | -0.7030 | -0.6993 | 0.4601 | 0.2753 | -0.9423 |
|  | C2-H4 | -0.6739 | -0.6705 | 0.4577 | 0.2691 | -0.8867 |
|  | C2-H5 | -0.7081 | -0.7062 | 0.4650 | 0.2766 | -0.9494 |
|  | C3-H6 | -0.2500 | -0.2479 | 0.3863 | 0.1253 | -0.1116 |
|  | C3-H7 | -0.7069 | -0.6877 | 0.4545 | 0.2754 | -0.9401 |
|  | C4-H8 | -0.7520 | -0.7230 | 0.4829 | 0.2814 | -0.9921 |
|  | C4-H9 | -0.7286 | -0.6998 | 0.4790 | 0.2759 | -0.9494 |
|  | N1-H11 | -1.2066 | -1.1922 | 0.6738 | 0.3307 | -1.7251 |
|  | N1-H6 | -0.5613 | -0.5509 | 0.6467 | 0.1963 | -0.4655 |
|  | C1-C2 | -0.4418 | -0.4388 | 0.3336 | 0.2422 | -0.5469 |
|  | C2-C3 | -0.4674 | -0.4517 | 0.3368 | 0.2520 | -0.5823 |
|  | C3-C4 | -0.5051 | -0.4760 | 0.3472 | 0.3472 | -0.6339 |
|  | O1-H11 | -0.0098 | -0.0072 | 0.0547 | 0.0106 | 0.0377 |
| 3TS4 | C1-H1 | -0.7014 | -0.6971 | 0.4603 | 0.2751 | -0.9382 |
|  | C1-H2 | -0.6974 | -0.6935 | 0.4607 | 0.2739 | -0.9303 |
|  | C1-H3 | -0.6784 | -0.6757 | 0.4573 | 0.2696 | -0.8968 |
|  | C2-H4 | -0.2731 | -0.2719 | 0.4005 | 0.1339 | -0.1445 |
|  | C2-H5 | -0.7236 | -0.7090 | 0.4605 | 0.2789 | -0.9721 |
|  | C3-H6 | -0.6818 | -0.6800 | 0.4603 | 0.2704 | -0.9015 |
|  | C3-H7 | -0.6935 | -0.6913 | 0.4603 | 0.2735 | -0.9245 |
|  | C4-H8 | -0.7485 | -0.7192 | 0.4810 | 0.2811 | -0.9866 |
|  | C4-H9 | -0.7449 | -0.7162 | 0.4801 | 0.2799 | -0.9810 |
|  | O1-H10 | -1.8466 | -1.7987 | 1.5549 | 0.3663 | -2.0905 |
|  | N1-H4 | -0.5148 | -0.5075 | 0.6499 | 0.1851 | -0.3724 |
|  | C1-C2 | -0.4655 | -0.4503 | 0.3351 | 0.2511 | -0.5807 |
|  | C2-C3 | -0.4684 | -0.4532 | 0.3383 | 0.2524 | -0.5833 |
|  | C3-C4 | -0.4863 | -0.4701 | 0.3479 | 0.2535 | -1.0141 |
|  | C4-O1 | -0.4511 | -0.4453 | 0.5093 | 0.2527 | -0.3871 |
| 3TS5 | C1-H1 | -0.7090 | -0.6948 | 0.4553 | 0.2760 | -0.9485 |
|  | C1-H2 | -0.2462 | -0.2456 | 0.3833 | 0.1240 | -0.1086 |
|  | C1-H3 | -0.7156 | -0.7005 | 0.4561 | 0.2772 | -0.9600 |
|  | C2-H4 | -0.6944 | -0.6901 | 0.4618 | 0.2736 | -0.9226 |
|  | C2-H5 | -0.6954 | -0.6936 | 0.4626 | 0.2730 | -0.9264 |
|  | C3-H6 | -0.6948 | -0.6918 | 0.4610 | 0.2741 | -0.9256 |
|  | C3-H7 | -0.6949 | -0.6928 | 0.4584 | 0.2741 | -0.9292 |
|  | C4-H8 | -0.7474 | -0.7179 | 0.4809 | 0.2809 | -0.9844 |
|  | C4-H9 | -0.7417 | -0.7125 | 0.4801 | 0.2795 | -0.9741 |
|  | O1-H10 | -1.8455 | -1.7976 | 1.5537 | 0.3663 | -2.0894 |
|  | N1-H2 | -0.5626 | -0.5550 | 0.6470 | 0.1962 | -0.4705 |
|  | C1-C2 | -0.4686 | -0.4512 | 0.3344 | 0.2520 | -0.5854 |
|  | C2-C3 | -0.4466 | -0.4416 | 0.3372 | 0.2438 | -0.5510 |
|  | C3-C4 | -0.4885 | -0.4727 | 0.3475 | 0.2546 | -0.6137 |
|  | C4-O1 | -0.4505 | -0.4459 | 0.5086 | 0.2527 | -0.3878 |
| CP1 | C1-H1 | -0.6967 | -0.6918 | 0.4579 | 0.2745 | -0.9306 |
|  | C1-H2 | -0.6872 | -0.6827 | 0.4554 | 0.2727 | -0.9145 |
|  | C1-H3 | -0.7094 | -0.7061 | 0.4618 | 0.2762 | -0.9538 |
|  | C2-H4 | -0.6863 | -0.6831 | 0.4578 | 0.2730 | -0.9117 |
|  | C2-H5 | -0.7011 | -0.7001 | 0.4617 | 0.2756 | -0.9394 |
|  | C3-H6 | -0.6965 | -0.6954 | 0.4603 | 0.2741 | -0.9316 |
|  | C3-H7 | -0.6864 | -0.6841 | 0.4567 | 0.2727 | -0.9138 |
|  | C4-H8 | -0.6965 | -0.6757 | 0.4697 | 0.2671 | -0.9024 |
|  | C4-H9 | -0.7181 | -0.6927 | 0.4761 | 0.2722 | -0.9347 |
|  | C4-H10 | -0.5342 | -0.5179 | 0.7963 | 0.2798 | -0.2558 |
|  | N1-H6 | -0.0068 | -0.0061 | 0.0424 | 0.0089 | 0.0295 |
|  | N1-H10 | -1.2486 | -1.2304 | 0.7086 | 0.3342 | -1.7705 |
|  | C1-C2 | -0.4455 | -0.4429 | 0.3345 | 0.2438 | -0.5539 |
|  | C2-C3 | -0.4461 | -0.4423 | 0.3360 | 0.2442 | -0.5524 |
|  | C3-C4 | -0.4822 | -0.4675 | 0.3488 | 0.2526 | -0.6009 |
|  | N1-H5 | -0.0043 | -0.0033 | 0.0338 | 0.0072 | 0.0262 |
|  | N1-H3 | -0.0046 | -0.0037 | 0.0287 | 0.0062 | 0.0203 |
|  | O1-H10 | -0.0157 | -0.0154 | 0.0745 | 0.0142 | 0.0434 |
| CP2 | RCP1 | -0.0042 | 0.0039 | 0.0281 | 0.0073 | 0.0278 |
|  | RCP2 | -0.0039 | 0.0046 | 0.0273 | 0.0074 | 0.0280 |
| CP3 | C1-H1 | -0.7025 | -0.6975 | 0.4598 | 0.2755 | -0.9401 |
|  | C1-H2 | -0.6919 | -0.6871 | 0.4569 | 0.2736 | -0.9221 |
|  | C1-H3 | -0.7049 | -0.7003 | 0.4602 | 0.2756 | -0.9451 |
|  | C2-H4 | -0.6666 | -0.6615 | 0.4582 | 0.2668 | -0.8699 |
|  | C2-H5 | -0.7075 | -0.7043 | 0.4648 | 0.2757 | -0.9470 |
|  | C3-N1 | -0.0036 | -0.0017 | 0.0244 | 0.0068 | 0.0192 |
|  | C3-H7 | -0.7364 | -0.7086 | 0.4555 | 0.2810 | -0.9895 |
|  | C4-H8 | -0.7554 | -0.7263 | 0.4843 | 0.2820 | -0.9974 |
|  | C4-H9 | -0.7235 | -0.6958 | 0.4804 | 0.2746 | -0.9389 |
|  | O1-H10 | -1.8468 | -1.7982 | 1.5573 | 0.3647 | -2.0877 |
|  | N1-H6 | -1.2035 | -1.1791 | 0.6661 | 0.3338 | -1.7166 |
|  | C1-C2 | -0.4412 | -0.4381 | 0.3337 | 0.2418 | -0.5456 |
|  | C2-C3 | -0.4928 | -0.4675 | 0.3372 | 0.2605 | -0.6231 |
|  | C3-C4 | -0.5277 | -0.4919 | 0.3491 | 0.2681 | -0.6704 |
|  | N1-H5 | -0.0026 | -0.0022 | 0.0284 | 0.0064 | 0.0236 |
|  | N1-H3 | -0.0038 | -0.0035 | 0.0283 | 0.0061 | 0.0210 |
|  | O1-H11 | -0.0185 | -0.0171 | 0.0871 | 0.0163 | 0.0514 |
| CP4 | C1-H1 | -0.7013 | -0.6964 | 0.4608 | 0.2749 | -0.9369 |
|  | C1-H2 | -0.6903 | -0.6854 | 0.4596 | 0.2720 | -0.9160 |
|  | C1-H3 | -0.6696 | -0.6659 | 0.4586 | 0.2669 | -0.8769 |
|  | C2-H4 | -0.0124 | -0.0115 | 0.0597 | 0.0132 | 0.0358 |
|  | C2-H5 | -0.7474 | -0.7216 | 0.4594 | 0.2829 | -1.0096 |
|  | C3-H6 | -0.6729 | -0.6695 | 0.4607 | 0.2678 | -0.8817 |
|  | C3-H7 | -0.6978 | -0.6946 | 0.4613 | 0.2738 | -0.9311 |
|  | C4-H8 | -0.7500 | -0.7210 | 0.4815 | 0.2814 | -0.9895 |
|  | C4-H9 | -0.7426 | -0.7140 | 0.4798 | 0.2794 | -0.9768 |
|  | O1-H10 | -1.8445 | -1.7967 | 1.5532 | 0.3664 | -2.0880 |
|  | N1-H4 | -1.2220 | -1.2036 | 0.6925 | 0.3316 | -1.7332 |
|  | C1-C2 | -0.4898 | -0.4636 | 0.3364 | 0.2591 | -0.6170 |
|  | C2-C3 | -0.4927 | -0.4676 | 0.3395 | 0.2604 | -0.6208 |
|  | C3-C4 | -0.4845 | -0.4692 | 0.3478 | 0.2529 | -0.6059 |
|  | N1-H9 | -0.0053 | -0.0045 | 0.0348 | 0.0075 | 0.0250 |
| CP5 | C1-H1 | -0.7421 | -0.7187 | 0.4570 | 0.2822 | -1.0039 |
|  | C1-H2 | -0.0093 | -0.0076 | 0.0470 | 0.0104 | 0.0301 |
|  | C1-H3 | -0.7376 | -0.7143 | 0.4563 | 0.2811 | -0.9956 |
|  | C2-H4 | -0.6965 | -0.6905 | 0.4622 | 0.2734 | -0.9248 |
|  | C2-H5 | -0.6886 | -0.6847 | 0.4630 | 0.2709 | -0.9102 |
|  | C3-H6 | -0.6957 | -0.6927 | 0.4609 | 0.2743 | -0.9275 |
|  | C3-H7 | -0.6911 | -0.6891 | 0.4575 | 0.2735 | -0.9228 |
|  | C4-H8 | -0.7455 | -0.7157 | 0.4805 | 0.2805 | -0.9808 |
|  | C4-H9 | -0.7479 | -0.7190 | 0.4812 | 0.2806 | -0.9857 |
|  | O1-H10 | -1.8450 | -1.7970 | 1.5536 | 0.3664 | -2.0884 |
|  | N1-H2 | -1.2208 | -1.2022 | 0.6879 | 0.3321 | -1.7352 |
|  | C1-C2 | -0.4924 | -0.4659 | 0.3346 | 0.2600 | -0.6237 |
|  | C2-C3 | -0.4452 | -0.4400 | 0.3372 | 0.2431 | -0.5481 |
|  | C3-C4 | -0.4908 | -0.4746 | 0.3484 | 0.2553 | -0.6170 |
|  | N1-H7 | -0.0062 | -0.0061 | 0.0420 | 0.0088 | 0.0297 |

**Ethanol plus NH reaction**


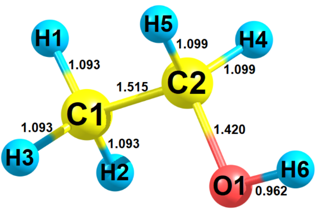

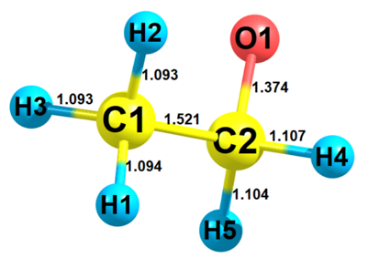

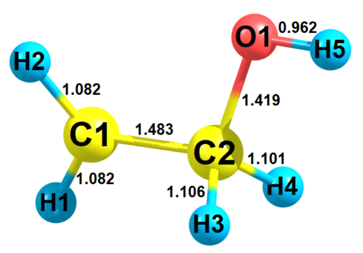

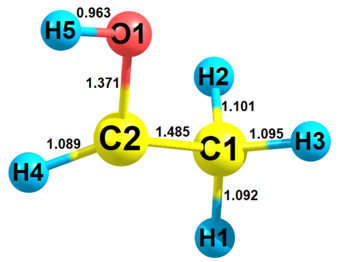


C_2_H_5_OH CH_3_CH_2_O CH_2_CH_2_OH CH_3_CHOH


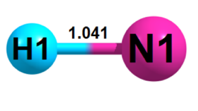

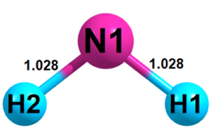

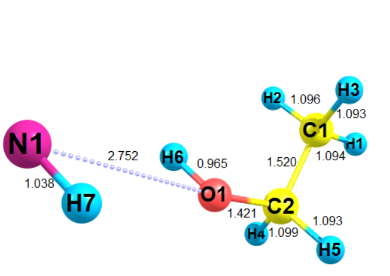

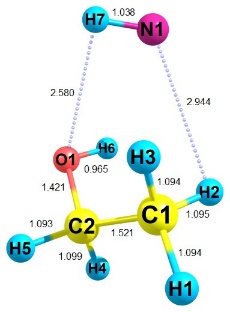


NH NH2 CR1(e-a) CR2(e-a)


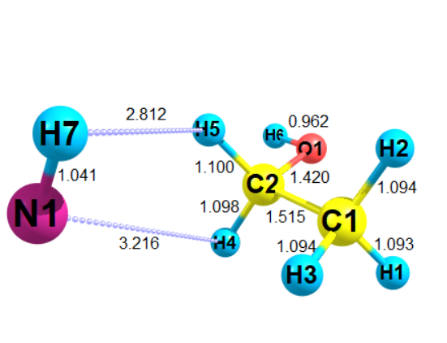

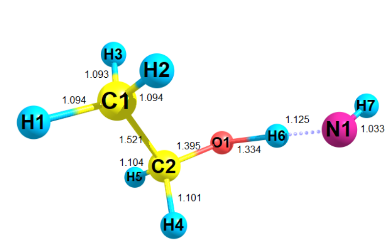

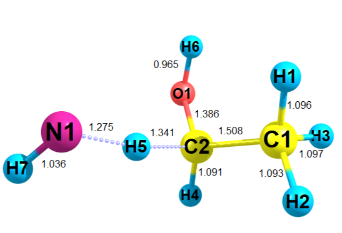


CR3(e-a) TS1(e-a) TS2(e-a)


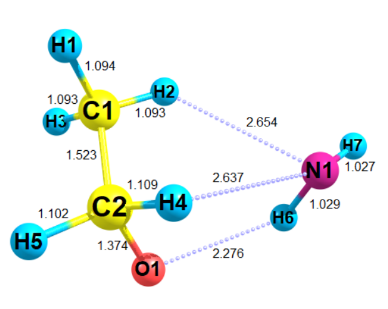

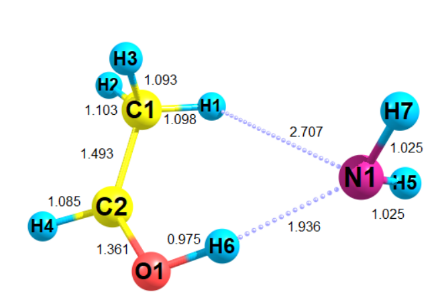

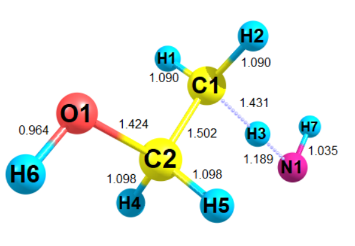

 TS3(e-a) CP1(e-a) CP2(e-a)


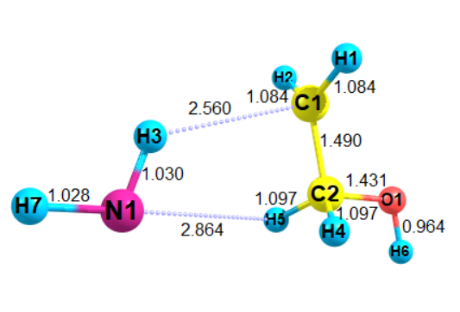


CP3 (e-a)

**Figure S1**. Structures of all stationary points including bond lengths (in angstrom) in the C_2_H_5_OH + NH reaction calculated at the M06-2X method.


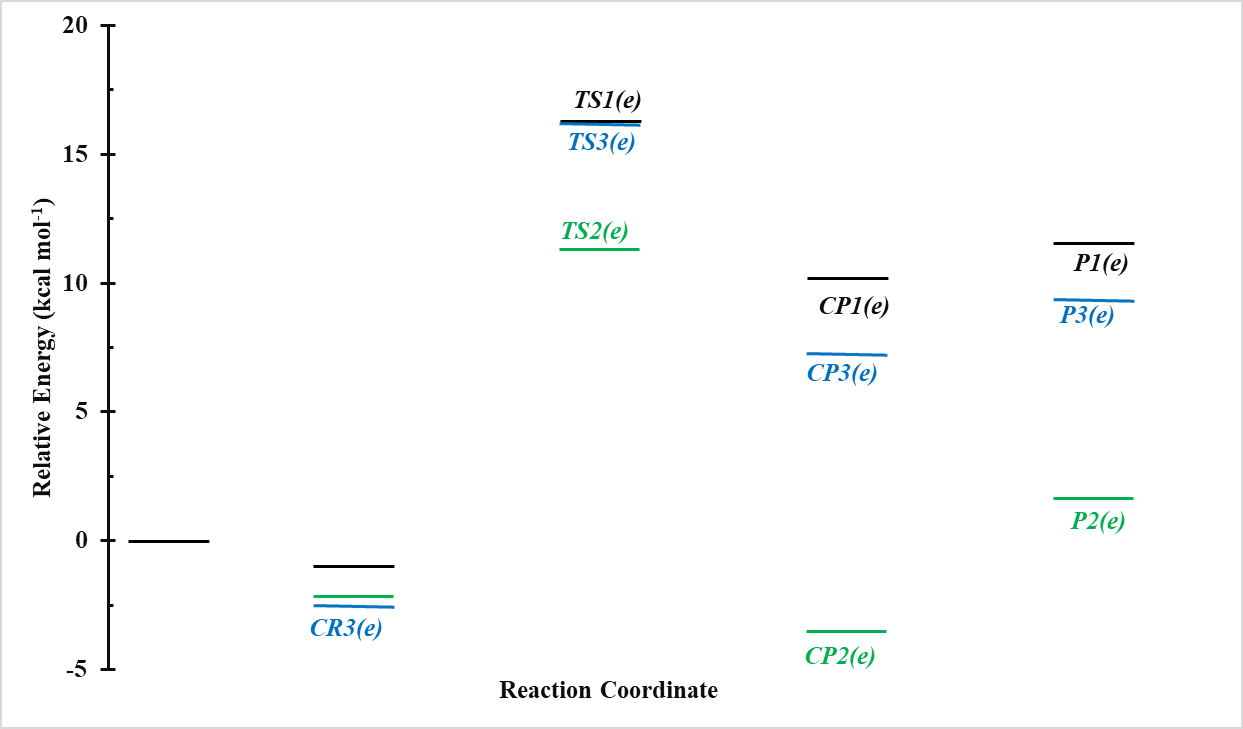


***R(e)***

***CR1(e)***

***CR2(e)***

**Figure S2**. Potential energy surface of the C_2_H_5_OH + NH reaction at the triplet ground state computed by the CBS-QB3 level.

**Table S25**. The computed relative energies for the stationary points of the C_2_H_5_OH + NH reaction. (Unit of all numbers is kcal mol^-1^)

| **Species** | ***∆E(0K)(W1BD)*** | ***∆E(0K)(CBS-QB3)*** | ***∆(E+ZPE)(M06-2X)*** | ***MUE^1^*** | ***MUE^2^*** | ***MUE^3^*** |
| --- | --- | --- | --- | --- | --- | --- |
| **R(e)** | 0.00 | 0.00 | 0.00 | 0.00 | 0.00 | 0.00 |
| **CR1(e-a)** | -1.96 | -2.04 | -2.82 | 0.00 | 0.08 | 0.86 |
| **CR2(e-a)** | -2.18 | -2.12 | -3.10 | 0.00 | 0.06 | 0.92 |
| **CR3(e-a)** | -0.31 | -0.36 | -1.04 | 0.00 | 0.05 | 0.73 |
| **TS1(e-a)** | 17.33 | 16.30 | 14.07 | 0.00 | 1.03 | 3.27 |
| **TS2(e-a)** | 12.59 | 11.34 | 10.23 | 0.00 | 1.25 | 2.36 |
| **TS3(e-a)** | 18.39 | 17.45 | 17.00 | 0.00 | 0.94 | 1.39 |
| **CP1(e-a)** | 9.90 | 10.17 | 8.78 | 0.00 | 0.27 | 1.11 |
| **CP2(e-a)** | -3.22 | -2.77 | -2.18 | 0.00 | 0.45 | 1.04 |
| **CP3(e-a)** | 6.56 | 9.46 | 10.45 | 0.00 | 2.90 | 3.89 |
| **P1(e) (CH_3_CH_2_O+NH_2_)** | 11.38 | 11.55 | 11.75 | 0.00 | 0.17 | 0.37 |
| **P2(e) (CH_3_CHOH+NH_2_)** | 1.11 | 1.61 | 3.47 | 0.00 | 0.50 | 2.36 |
| **P3(e) (CH_2_CH_2_OH+NH_2_)** | 8.65 | 9.32 | 11.40 | 0.00 | 0.67 | 2.75 |

MUE^1^, MUE^2^, and MUE^3^ are the mean unsinged errors for the W1BD, CBS-QB3, and M06-2X methods, respectively*.*

**Table S26**. Thermodynamic parameters for stationary points of the C_2_H_5_OH + NH reaction. (Unit of all numbers is kcal mol^-1^)

| **Species** | ***∆E˚(A)*** | ***∆E˚(B)*** | ***∆E˚(C)*** | ***∆H˚(A)*** | ***∆H˚(B)*** | ***∆H˚(C)*** | ***∆G˚(A)*** | ***∆G˚(B)*** | ***∆G˚(C)*** | ***T∆S˚(A)*** | ***T∆S˚(B)*** | ***T∆S˚(C)*** |
| --- | --- | --- | --- | --- | --- | --- | --- | --- | --- | --- | --- | --- |
| **R(e)** | 0.00 | 0.00 | 0.00 | 0.00 | 0.00 | 0.00 | 0.00 | 0.00 | 0.00 | 0.00 | 0.00 | 0.00 |
| **CR1(e-a)** | -2.20 | -1.97 | -1.23 | -2.79 | -1.97 | -1.82 | 3.74 | 3.81 | 3.63 | -6.53 | -5.78 | -5.45 |
| **CR2(e-a)** | -2.83 | -2.26 | -1.65 | -3.42 | -2.26 | -2.24 | 3.87 | 3.80 | 3.52 | -7.29 | -6.07 | -5.76 |
| **CR3(e-a)** | -1.16 | 0.19 | 0.86 | -1.75 | 0.19 | 0.26 | 6.54 | 3.39 | 3.34 | -8.29 | -3.20 | -3.07 |
| **TS1(e-a)** | 13.92 | 15.55 | 17.20 | 13.32 | 15.55 | 16.61 | 21.95 | 23.71 | 24.59 | -8.62 | -8.15 | -7.98 |
| **TS2(e-a)** | 13.46 | 10.74 | 12.58 | 12.87 | 10.74 | 11.99 | 22.17 | 18.70 | 19.94 | -9.31 | -7.96 | -7.95 |
| **TS3(e-a)** | 10.24 | 16.99 | 18.51 | 9.65 | 16.99 | 17.92 | 17.61 | 24.42 | 25.40 | -7.96 | -7.43 | -7.48 |
| **CP1(e-a)** | 17.08 | 10.35 | 10.77 | 16.49 | 10.35 | 10.17 | 24.27 | 16.09 | 15.59 | -7.79 | -5.74 | -5.42 |
| **CP2(e-a)** | -2.62 | -2.48 | -2.24 | -3.21 | -2.48 | -2.83 | 4.41 | 3.03 | 1.84 | -7.62 | -5.51 | -4.68 |
| **CP3(e-a)** | -1.54 | 10.01 | 7.82 | -2.14 | 10.01 | 7.22 | 4.51 | 13.16 | 11.49 | -6.64 | -3.14 | -4.26 |
| **P1(e) (CH_3_CH_2_O+NH_2_)** | 11.58 | 11.38 | 11.78 | 10.99 | 11.97 | 11.78 | 16.17 | 10.24 | 10.10 | -5.18 | 1.74 | 1.68 |
| **P2(e) (CH_3_CHOH+NH_2_)** | 11.98 | 1.38 | 1.47 | 11.98 | 1.97 | 1.47 | 10.77 | 0.55 | 0.07 | 1.21 | 1.42 | 1.40 |
| **P3(e) (CH_2_CH_2_OH+NH_2_)** | 3.83 | 9.27 | 9.22 | 3.83 | 9.86 | 9.22 | 2.41 | 8.07 | 7.30 | 1.42 | 1.79 | 1.92 |

A, B and C refer to the M06-2X, CBS-QB3, and W1BD methods, respectively.

**Table S27**. Absolute energies and thermodynamic parameters for stationary points of the C_2_H_5_OH + NH reaction computed at the W1BD level.

| **Species** | ***E(0K)(Hartree)*** | ***E˚(Hartree)*** | ***H˚(Hartree)*** | ***G˚(Hartree)*** |
| --- | --- | --- | --- | --- |
| **Ethanol** | -155.0318 | -155.0274 | -155.0265 | -155.0572 |
| **CR1(e-a)** | -210.2711 | -210.2632 | -210.2623 | -210.3049 |
| **CR2(e-a)** | -210.2714 | -210.2639 | -210.2629 | -210.3051 |
| **CR3(e-a)** | -210.2685 | -210.2599 | -210.2589 | -210.3054 |
| **TS1(e-a)** | -210.2403 | -210.2338 | -210.2329 | -210.2715 |
| **TS2(e-a)** | -210.2479 | -210.2412 | -210.2402 | -210.2789 |
| **TS3(e-a)** | -210.2387 | -210.2317 | -210.2308 | -210.2702 |
| **CP1(e-a)** | -210.2522 | -210.2441 | -210.2431 | -210.2858 |
| **CP2(e-a)** | -210.2731 | -210.2648 | -210.2639 | -210.3077 |
| **CP3(e-a)** | -210.2575 | -210.2488 | -210.2478 | -210.2924 |
| **CH_3_CH_2_O** | -154.3664 | -154.3619 | -154.3610 | -154.3929 |
| **CH_3_CHOH** | -154.3828 | -154.3783 | -154.3774 | -154.4088 |
| **CH_2_CH_2_OH** | -154.3708 | -154.3660 | -154.3650 | -154.3973 |

**Table S28**. Absolute energies and thermodynamic parameters for stationary points of the C_2_H_5_OH + NH reaction computed at the CBS-QB3 level.

| **Species** | ***E(0K)(Hartree)*** | ***E˚(Hartree)*** | ***H˚(Hartree)*** | ***G˚(Hartree)*** |
| --- | --- | --- | --- | --- |
| **Ethanol** | -154.7702 | -154.7649 | -154.7658 | -154.7956 |
| **CR1(e-a)** | -209.9179 | -209.9092 | -209.9102 | -209.9512 |
| **CR2(e-a)** | -209.9180 | -209.9097 | -209.9106 | -209.9512 |
| **CR3(e-a)** | -209.9152 | -209.9058 | -209.9067 | -209.9519 |
| **TS1(e-a)** | -209.8887 | -209.8813 | -209.8822 | -209.9195 |
| **TS2(e-a)** | -209.8966 | -209.8890 | -209.8899 | -209.9275 |
| **TS3(e-a)** | -209.8868 | -209.8790 | -209.8799 | -209.9184 |
| **CP1(e-a)** | -209.8984 | -209.8896 | -209.8905 | -209.9317 |
| **CP2(e-a)** | -209.9191 | -209.9100 | -209.9110 | -209.9525 |
| **CP3(e-a)** | -209.8996 | -209.8901 | -209.8911 | -209.9363 |
| **CH_3_CH_2_O** | -154.1050 | -154.0996 | -154.1005 | -154.1315 |
| **CH_3_CHOH** | -154.1209 | -154.1155 | -154.1165 | -154.1469 |
| **CH_2_CH_2_OH** | -154.1086 | -154.1029 | -154.1039 | -154.1349 |

**Table S29**. Absolute energies and thermodynamic parameters for stationary points of the C_2_H_5_OH + NH reaction calculated at the M06-2X method.

| **Species** | ***(E+ZPE) (Hartree)*** | ***E˚(Hartree)*** | ***H˚(Hartree)*** | ***G˚(Hartree)*** | ***S˚(cal/K mol)*** |
| --- | --- | --- | --- | --- | --- |
| **Ethanol** | -154.8913 | -154.8871 | -154.8861 | -154.9167 | 64.248 |
| **CR1(e-a)** | -55.1902 | -55.1879 | -55.1869 | -55.2075 | 43.257 |
| **CR2(e-a)** | -210.0675 | -210.0600 | -210.0591 | -210.0993 | 84.664 |
| **CR3(e-a)** | -210.0865 | -210.0795 | -210.0785 | -210.1180 | 83.039 |
| **TS1(e-a)** | -210.0832 | -210.0768 | -210.0758 | -210.1137 | 79.684 |
| **TS2(e-a)** | -210.0591 | -210.0535 | -210.0526 | -210.0888 | 76.288 |
| **TS3(e-a)** | -210.0652 | -210.0586 | -210.0577 | -210.0961 | 80.804 |
| **CP1(e-a)** | -210.0545 | -210.0477 | -210.0468 | -210.0854 | 81.391 |
| **CP2(e-a)** | -210.0860 | -210.0791 | -210.0782 | -210.1171 | 81.962 |
| **CP3(e-a)** | -210.0850 | -210.0774 | -210.0765 | -210.1169 | 85.223 |
| **CH_3_CH_2_O** | -210.0649 | -210.0565 | -210.0555 | -210.0984 | 90.121 |
| **CH_3_CHOH** | -154.2335 | -154.2294 | -154.2285 | -154.2594 | 65.058 |
| **CH_2_CH_2_OH** | -154.2467 | -154.2424 | -154.2414 | -154.2727 | 65.766 |

**Table S30**. The CCSD(T) absolute energies (in Hartree) and T1 diagnostic values for stationary points of the ethanol + NH reaction calculated at the CCSD(T)/6-31+g(d**´**)//M06-2X level of theory.

| **Species** | ***CCSD(T)*** | ***T1diagnostic*** |
| --- | --- | --- |
| **CR1(e-a)** | -209.6670 | 0.0114 |
| **CR2(e-a)** | -209.6690 | 0.0109 |
| **CR3(e-a)** | -209.6642 | 0.0109 |
| **TS1(e-a)** | -209.6308 | 0.0237 |
| **TS2(e-a)** | -209.6314 | 0.0173 |
| **TS3(e-a)** | -209.6204 | 0.0152 |
| **CP1(e-a)** | -209.6498 | 0.0176 |
| **CP2(e-a)** | -209.6610 | 0.0150 |
| **CP3(e-a)** | -209.6378 | 0.0117 |

**Propanol plus NH reaction**


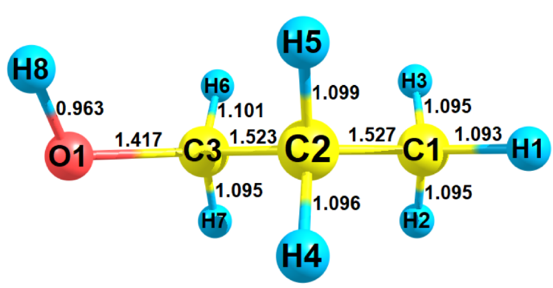

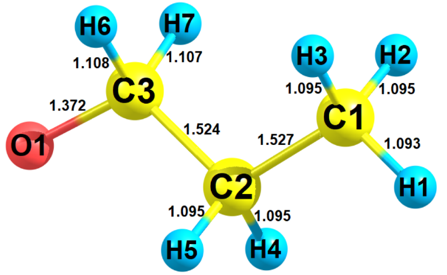

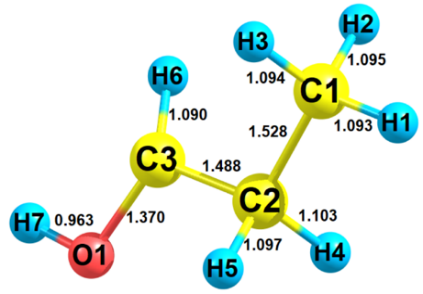


Propanol-a CH_3_CH_2_CH_2_O-a CH_3_CH_2_CHOH-a


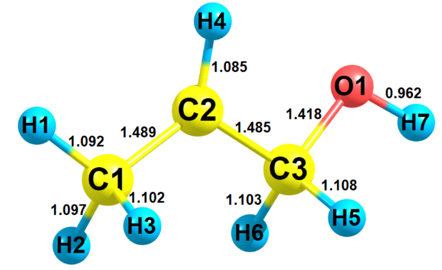

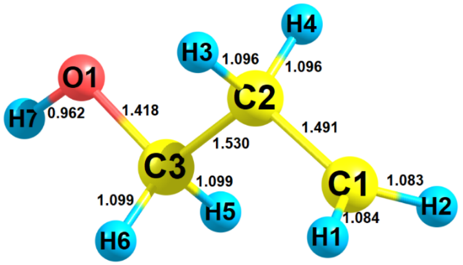

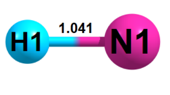

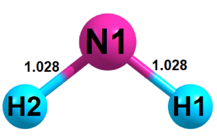


**
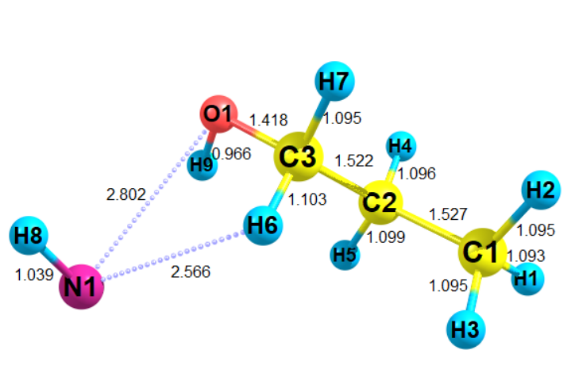
** CH_3_CHCH_2_OH-a CH_2_CH_2_CH_2_OH-a NH NH_2_

**
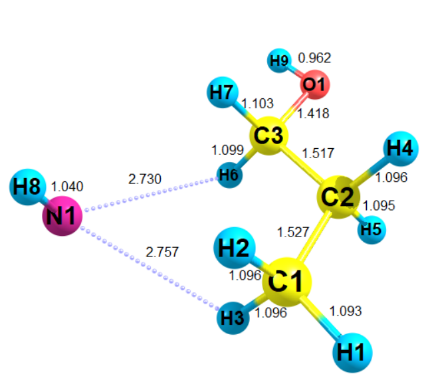

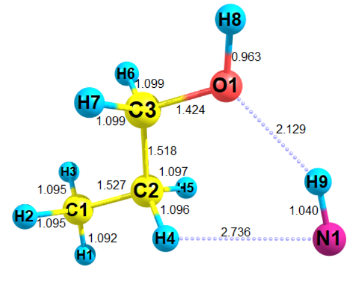
**

CR1(pr-a) CR2(pr-a) CR3(pr-a)

**
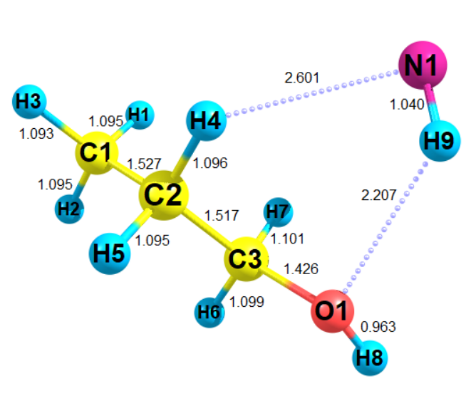

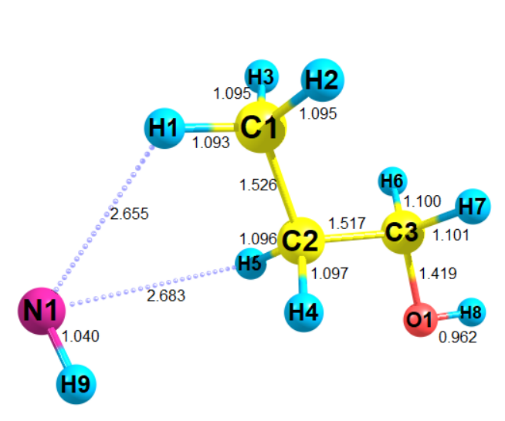
**

CR4(pr-a1) CR4(pr-a2)


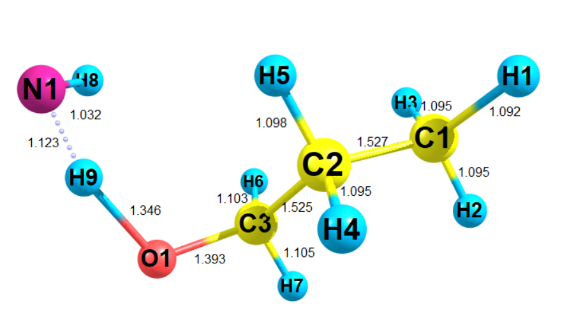

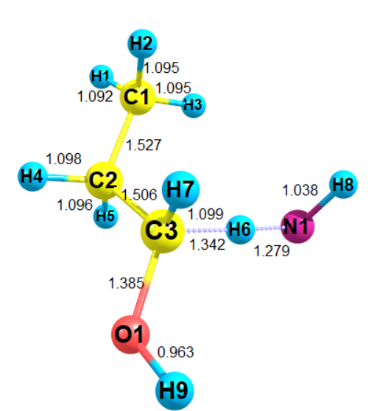


TS1(pr-a) TS2(pr-a)

**
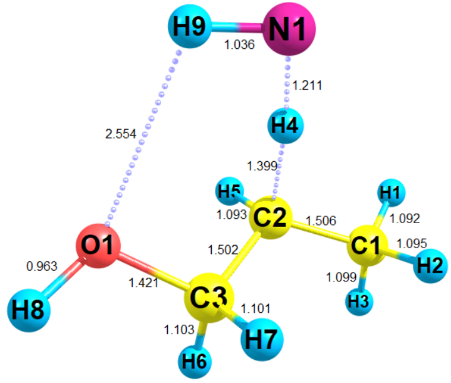

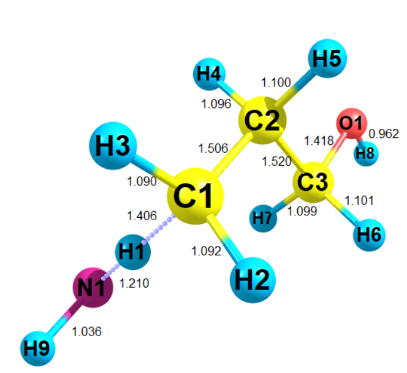

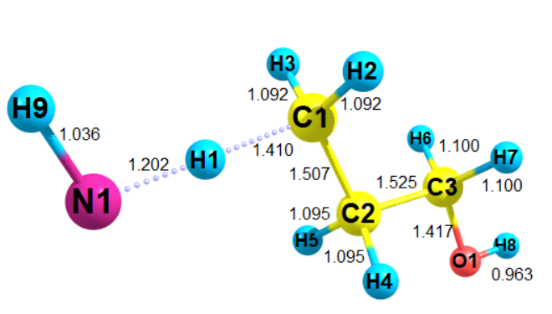
**

TS3(pr-a) TS4(pr-a) TS4(pr-a2)

**
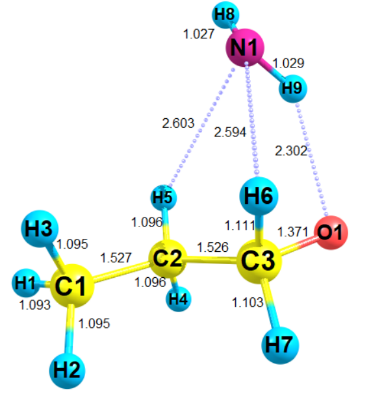

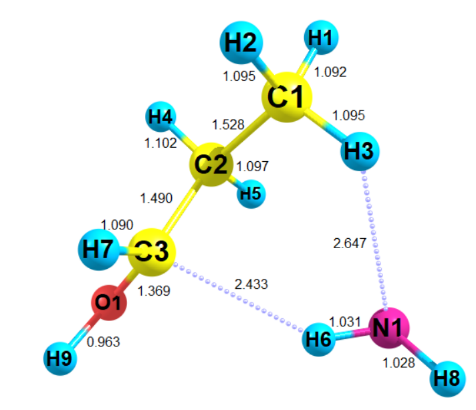
**

CP1(pr-a) CP2 (pr-a)

**
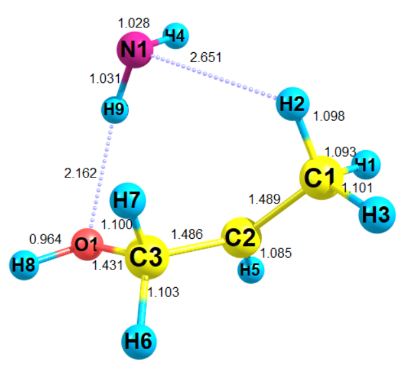

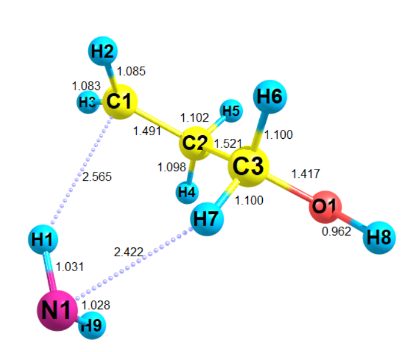

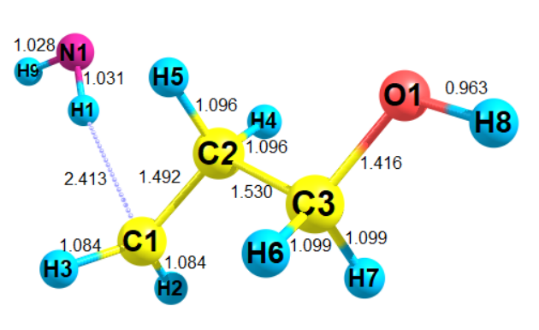
** CP4(pr-a) CP3(pr-a) CP4(pr-a2)

**Figure S3**. Structures of all stationary points including bond lengths (in angstrom) in the n-C_3_H_7_OH + NH reaction calculated at the M06-2X method.


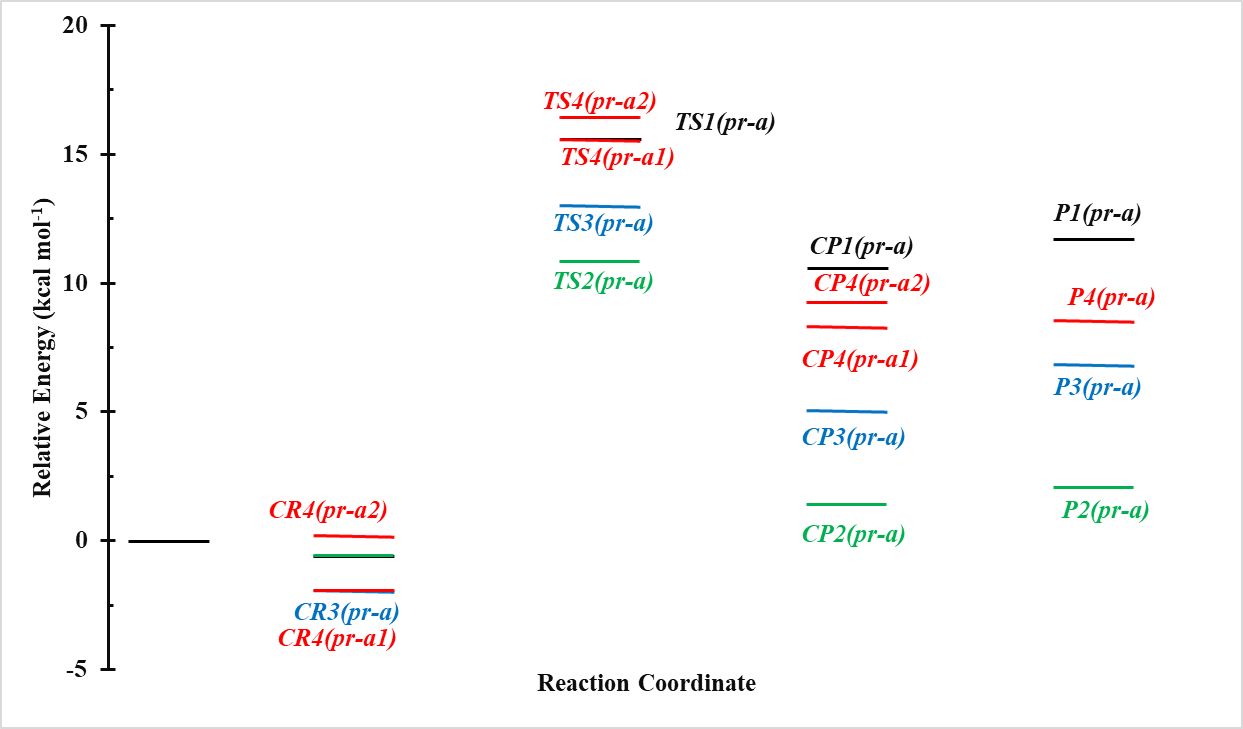


***R(pr-a)***

***CR1(pr-a)***

***CR2(pr-a)***

**Figure S4**. Potential energy surface of the n-propanol-a + NH reaction at the triplet ground state computed by the CBS-QB3 level.

**Table S31**. Relative energies and thermodynamic parameters for stationary points of the n-propanol-a + NH. (Unit of all numbers is kcal mol^-1^)

| **Species** | ***∆(E+ZPE)(A)*** | ***∆E(0K)(B)*** | ***∆E˚(A)*** | ***∆E˚(B)*** | ***∆H˚(A)*** | ***∆H˚(B)*** | ***∆G˚(A)*** | ***∆G˚(B)*** | ***T∆S˚(A)*** | ***T∆S˚(B)*** |
| --- | --- | --- | --- | --- | --- | --- | --- | --- | --- | --- |
| **R(pr-a)** | 0.00 | 0.00 | 0.00 | 0.00 | 0.00 | 0.00 | 0.00 | 0.00 | 0.00 | 0.00 |
| **CR1(pr-a)** | -3.09 | -1.24 | -2.67 | -0.58 | -3.26 | -1.17 | 3.72 | 4.86 | -6.98 | -6.03 |
| **CR2(pr-a)** | -1.66 | -1.29 | -1.05 | -0.59 | -1.64 | -1.19 | 5.18 | 4.70 | -6.82 | -5.89 |
| **CR3(pr-a)** | -3.96 | -2.48 | -3.61 | -1.99 | -4.20 | -2.58 | 3.28 | 3.40 | -7.48 | -5.97 |
| **CR4(pr-a)** | -3.71 | -2.48 | -3.46 | -1.99 | -4.05 | -2.58 | 3.61 | 3.40 | -7.67 | -5.98 |
| **CR4(pr-a2)** | -1.43 | -0.93 | -0.74 | 0.18 | -1.33 | -0.42 | 5.01 | 2.79 | -6.33 | -3.21 |
| **TS1(pr-a)** | 14.17 | 15.73 | 14.03 | 15.59 | 13.44 | 15.00 | 21.94 | 23.25 | -8.50 | -8.25 |
| **TS2(pr-a)** | 10.13 | 10.76 | 10.29 | 10.80 | 9.70 | 10.20 | 17.18 | 18.22 | -7.48 | -8.02 |
| **TS3(pr-a)** | 11.88 | 13.02 | 12.01 | 12.97 | 11.41 | 12.38 | 19.41 | 20.99 | -8.00 | -8.61 |
| **TS4(pr-a)** | 15.33 | 15.49 | 15.19 | 15.54 | 14.60 | 14.95 | 23.20 | 22.63 | -8.60 | -7.68 |
| **TS4(pr-a2)** | 16.10 | 16.24 | 16.09 | 16.38 | 15.50 | 15.79 | 23.65 | 23.09 | -8.15 | -7.30 |
| **CP1(pr-a)** | 8.03 | 9.72 | 8.34 | 10.57 | 7.75 | 9.98 | 15.20 | 15.63 | -7.45 | -5.65 |
| **CP2(pr-a)** | 0.87 | 0.23 | 2.05 | 1.38 | 1.46 | 0.79 | 6.28 | 5.39 | -4.83 | -4.60 |
| **CP3(pr-a)** | 3.60 | 3.87 | 4.58 | 5.01 | 3.99 | 4.42 | 10.24 | 9.42 | -6.25 | -5.00 |
| **CP4(pr-a)** | 8.63 | 6.95 | 8.18 | 8.26 | 8.99 | 7.67 | 14.29 | 12.42 | -6.98 | -4.75 |
| **CP4(pr-a2)** | 7.56 | 7.77 | 9.58 | 9.19 | 7.59 | 8.60 | 14.57 | 12.56 | -5.30 | -3.96 |
| **P1(pr-a) (CH_3_CH_2_CH_2_O+NH_2_)** | 11.56 | 11.35 | 9.58 | 11.72 | 8.99 | 11.72 | 14.29 | 10.15 | 1.27 | 1.57 |
| **P2(pr-a) (CH_3_CH_2_CHOH+NH_2_)** | 3.78 | 1.66 | 11.82 | 2.03 | 11.82 | 2.03 | 10.56 | 0.51 | 1.44 | 1.52 |
| **P3(pr-a) (CH_3_CHCH_2_OH+NH_2_)** | 8.08 | 6.13 | 4.14 | 6.81 | 4.14 | 6.81 | 2.70 | 4.35 | 2.52 | 2.46 |
| **P4(pr-a) (CH_2_CH_2_CH_2_OH+NH_2_)** | 10.00 | 7.94 | 8.80 | 8.50 | 8.80 | 8.50 | 6.29 | 6.58 | 1.94 | 1.92 |

A and B refer to the M06-2X and CBS-QB3 methods, respectively.

**Table S32**. Relative energies and thermodynamic parameters for stationary points of the n-propanol-a + NH calculated at the CBS-QB3 method.

| **Species** | ***E(0K) (Hartree)*** | ***E˚(Hartree)*** | ***H˚(Hartree)*** | ***G˚(Hartree)*** |
| --- | --- | --- | --- | --- |
| **Propanol-a** | -193.9952 | -193.9896 | -193.9887 | -194.0229 |
| **CR1(pr-a)** | -55.1445 | -249.1327 | -249.1317 | -249.1769 |
| **CR2(pr-a)** | -249.1397 | -249.1327 | -249.1317 | -249.1772 |
| **CR3(pr-a)** | -249.1416 | -249.1349 | -249.1340 | -249.1793 |
| **CR4(pr-a)** | -249.1417 | -249.1349 | -249.1340 | -249.1793 |
| **CR4(pr-a2)** | -249.1436 | -249.1315 | -249.1305 | -249.1802 |
| **TS1(pr-a)** | -249.1436 | -249.1069 | -249.1060 | -249.1476 |
| **TS2(pr-a)** | -249.1411 | -249.1145 | -249.1136 | -249.1556 |
| **TS3(pr-a)** | -249.1146 | -249.1111 | -249.1101 | -249.1512 |
| **TS4(pr-a)** | -249.1225 | -249.1070 | -249.1060 | -249.1486 |
| **TS4(pr-a2)** | -249.1189 | -249.1056 | -249.1047 | -249.1479 |
| **CP1(pr-a)** | -249.1150 | -249.1149 | -249.1140 | -249.1598 |
| **CP2(pr-a)** | -249.1138 | -249.1295 | -249.1286 | -249.1761 |
| **CP3(pr-a)** | -249.1242 | -249.1238 | -249.1228 | -249.1697 |
| **CP4(pr-a)** | -249.1393 | -249.1186 | -249.1176 | -249.1649 |
| **CP4(pr-a2)** | -249.1335 | -249.1171 | -249.1162 | -249.1647 |
| **CH_3_CH_2_CH_2_O-a** | -193.3304 | -193.3247 | -193.3238 | -193.3590 |
| **CH_3_CH_2_CHOH-a** | -193.3458 | -193.3402 | -193.3392 | -193.3743 |
| **CH_3_CHCH_2_OH-a** | -193.3387 | -193.3325 | -193.3316 | -193.3682 |
| **CH_2_CH_2_CH_2_OH-a** | -193.3358 | -193.3298 | -193.3289 | -193.3647 |

**Table S33**. Relative energies and thermodynamic parameters for stationary points of the n-propanol-a + NH calculated at the M06-2X method.

| **Species** | ***(E+ZPE) (Hartree)*** | ***E˚(Hartree)*** | ***H˚(Hartree)*** | ***G˚(Hartree)*** | ***S˚(Hartree)*** |
| --- | --- | --- | --- | --- | --- |
| **Propanol-a** | -194.1578 | -194.1524 | -194.1515 | -194.1854 | 71.527 |
| **CR1(pr-a)** | -249.3529 | -249.3445 | -249.3436 | -249.3870 | 91.366 |
| **CR2(pr-a)** | -249.3507 | -249.3419 | -249.3410 | -249.3847 | 91.916 |
| **CR3(pr-a)** | -249.3543 | -249.3460 | -249.3451 | -249.3877 | 89.696 |
| **CR4(pr-a)** | -249.3539 | -249.3458 | -249.3448 | -249.3872 | 89.065 |
| **CR4(pr-a2)** | -249.3503 | -249.3414 | -249.3405 | -249.3849 | 93.539 |
| **TS1(pr-a)** | -249.3254 | -249.3179 | -249.3170 | -249.3580 | 86.290 |
| **TS2(pr-a)** | -249.3319 | -249.3239 | -249.3229 | -249.3656 | 89.704 |
| **TS3(pr-a)** | -249.3291 | -249.3211 | -249.3202 | -249.3620 | 87.954 |
| **TS4(pr-a)** | -249.3236 | -249.3161 | -249.3151 | -249.3560 | 85.952 |
| **TS4(pr-a2)** | -249.3224 | -249.3146 | -249.3137 | -249.3552 | 87.448 |
| **CP1(pr-a)** | -249.3352 | -249.3270 | -249.3260 | -249.3687 | 89.790 |
| **CP2(pr-a)** | -249.3466 | -249.3370 | -249.3361 | -249.3829 | 98.598 |
| **CP3(pr-a)** | -249.3423 | -249.3330 | -249.3320 | -249.3766 | 93.814 |
| **CP4(pr-a)** | -249.3360 | -249.3272 | -249.3263 | -249.3697 | 91.367 |
| **CP4(pr-a2)** | -249.3343 | -249.3250 | -249.3241 | -249.3702 | 97.004 |
| **CH_3_CH_2_CH_2_O-a** | -193.5003 | -193.4950 | -193.4940 | -193.5285 | 72.528 |
| **CH_3_CH_2_CHOH-a** | -193.5127 | -193.5072 | -193.5063 | -193.5410 | 73.106 |
| **CH_3_CHCH_2_OH-a** | -193.5058 | -193.4998 | -193.4988 | -193.5353 | 76.723 |
| **CH_2_CH_2_CH_2_OH-a** | -193.5028 | -193.4969 | -193.4960 | -193.5315 | 74.778 |

**Table S34**. The CCSD(T) absolute energies (in Hartree) and T1 diagnostic values for stationary points of the n-propanol-a + NH reaction calculated at the CCSD(T)/6-31+g(d**´**)//M06-2X level of theory.

| **Species** | ***CCSD(T)*** | ***T1 diagnostic*** |
| --- | --- | --- |
| **Propanol-a** | -193.7650 | 0.0105 |
| **CR1(pr-a)** | -248.8519 | 0.0112 |
| **CR2(pr-a)** | -248.8484 | 0.0106 |
| **CR3(pr-a)** | -248.8546 | 0.0105 |
| **CR4(pr-a)** | -248.8492 | 0.0106 |
| **CR4(pr-a2)** | -248.8536 | 0.0106 |
| **TS1(pr-a)** | -248.8153 | 0.0220 |
| **TS2(pr-a)** | -248.8148 | 0.0162 |
| **TS3(pr-a)** | -248.8127 | 0.0148 |
| **TS4(pr-a)** | -248.8065 | 0.0144 |
| **TS4(pr-a2)** | -248.8071 | 0.0144 |
| **CP1(pr-a)** | -248.8347 | 0.0163 |
| **CP2(pr-a)** | -248.8366 | 0.0140 |
| **CP3(pr-a)** | -248.8323 | 0.0126 |
| **CP4(pr-a)** | -248.8252 | 0.0118 |
| **CP4(pr-a2)** | -248.8259 | 0.0120 |
| **CH_3_CH_2_CH_2_O-a** | -193.1126 | 0.0161 |
| **CH_3_CH_2_CHOH-a** | -193.1169 | 0.0144 |
| **CH_3_CHCH_2_OH-a** | -193.1085 | 0.0124 |
| **CH_2_CH_2_CH_2_OH-a** | -193.1064 | 0.0119 |

**N-Propanol-b plus NH reaction**

**
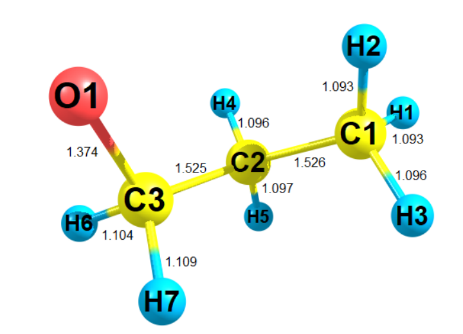

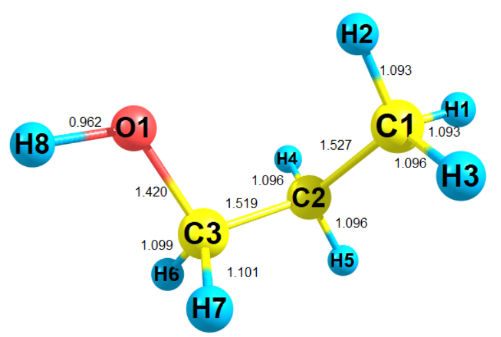

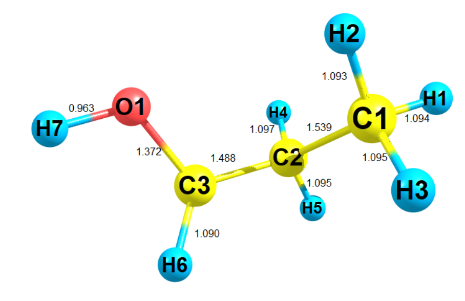
**
 N-Propanol-b CH_3_CH_2_CH_2_O-b CH_3_CH_2_CHOH-b

**
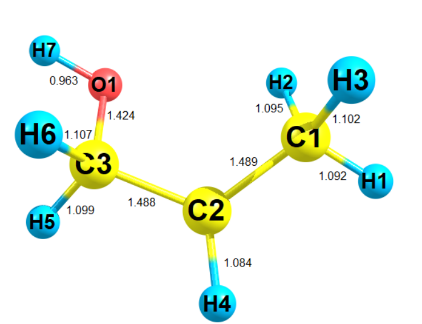

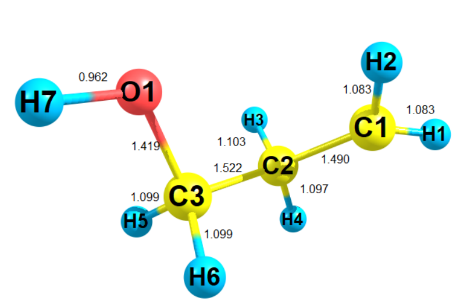
**
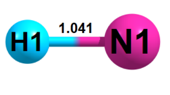

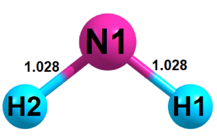


CH_3_CHCH_2_OH-b CH_2_CH_2_CH_2_OH-b NH NH_2_

**
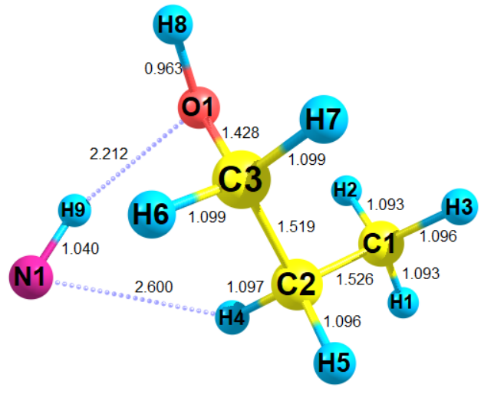

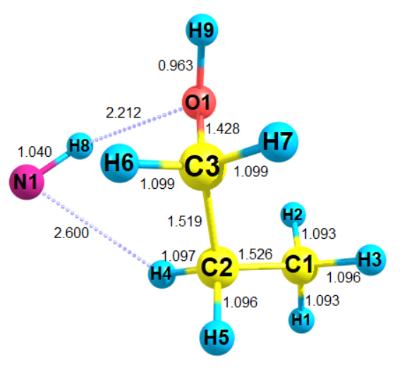

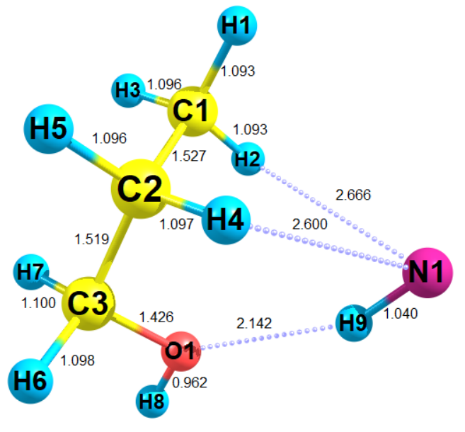
**
 CR1(pr-b) CR2(pr-b) CR3(pr-b)

**
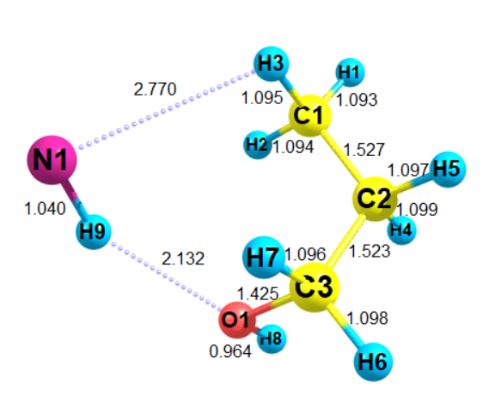

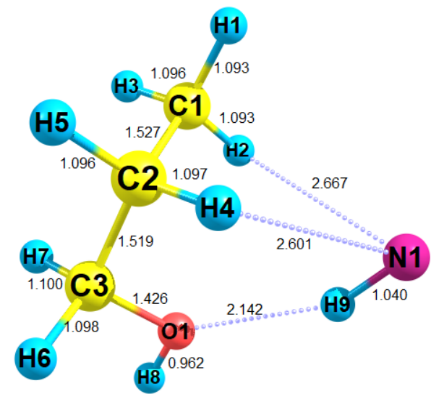

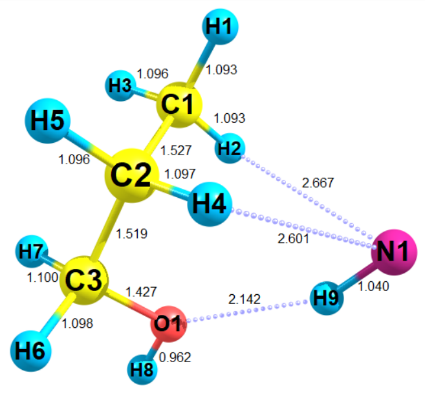
**

CR4(pr-b1) CR4(pr-b2) CR4(pr-b3)

**
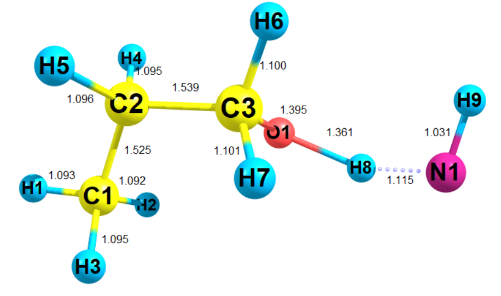
**
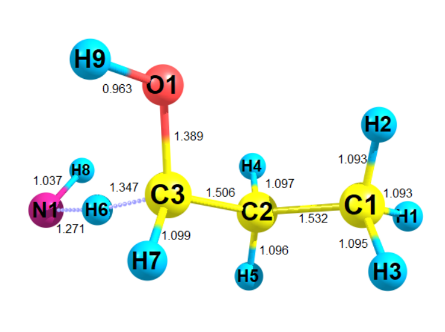
 **
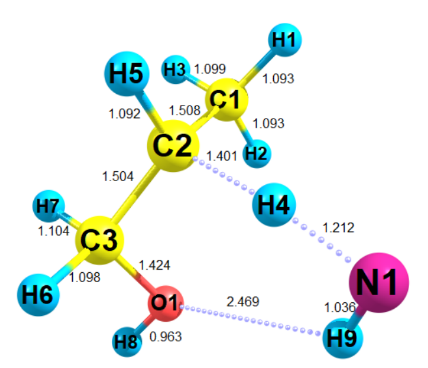
**

TS1(pr-b) TS2(pr-b) TS3(pr-b)

**
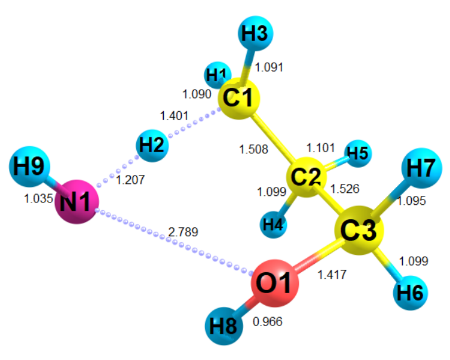

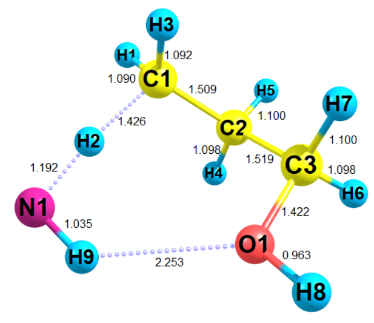
** **
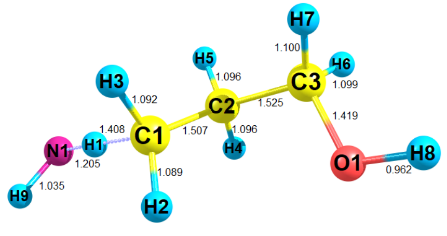
**

TS4(pr-b1) TS4(pr-b2) TS4(pr-b3)

**
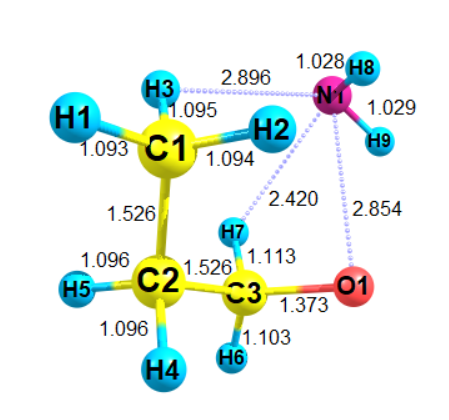

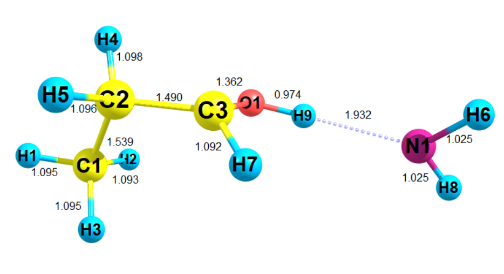

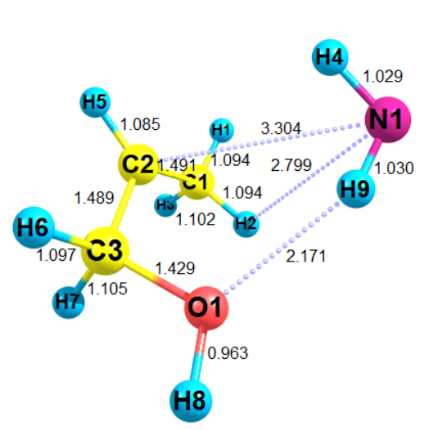
**

CP1(pr-b) CP2 (pr-b) CP3(pr-b)

**
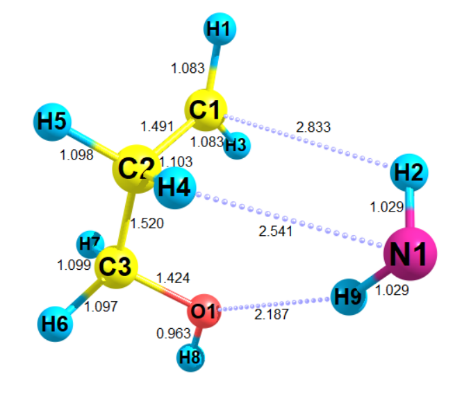

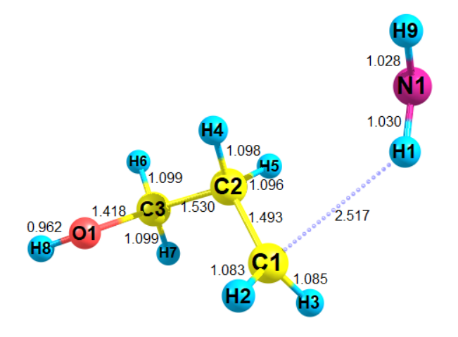

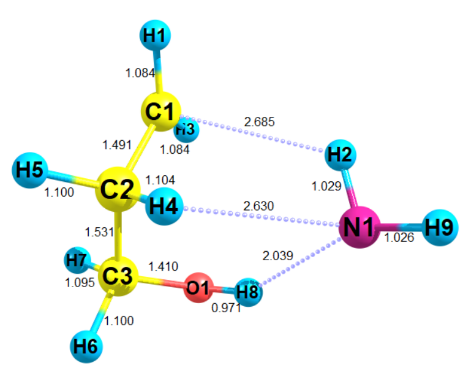
**

CP4(pr-b1) CP4(pr-b2) CP4(pr-b3)

**Figure S5**. Structures of all stationary points including bond lengths (in angstrom) in the n-C_3_H_7_OH + NH reaction calculated at the M06-2X method.


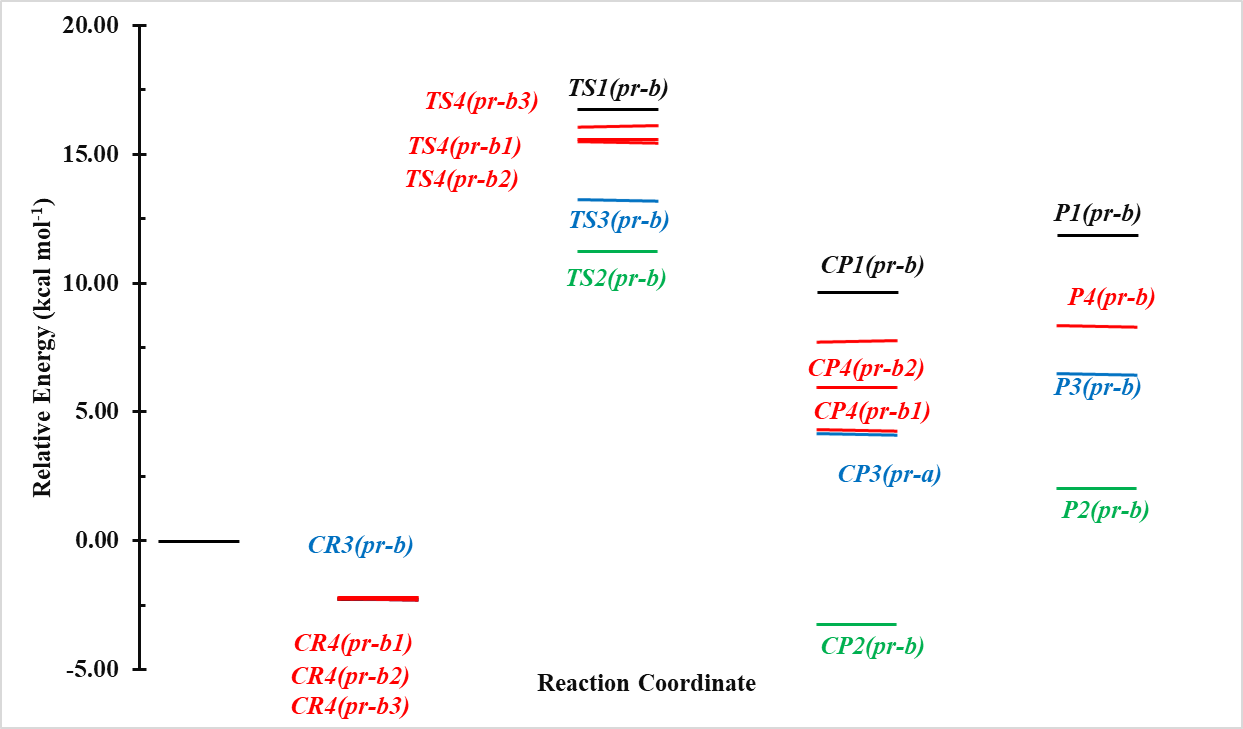


***R(pr-b)***

***CR1(pr-a)***

***CR2(pr-b)***

***CP4(pr-b2)***

**Figure S6**. Potential energy surface of the n-propanol-b reaction at the triplet ground state computed by the CBS-QB3 level.

**Table S35**. Relative energies and thermodynamic parameters for stationary points of the n-propanol-b + NH. (Unit of all numbers is kcal mol^-1^)

| **Species** | ***∆(E+ZPE)(A)*** | ***∆E(0K)(B)*** | ***∆E˚(A)*** | ***∆E˚(B)*** | ***∆H˚(A)*** | ***∆H˚(B)*** | ***∆G˚(A)*** | ***∆G˚(B)*** | ***T∆S˚(A)*** | ***T∆S˚(B)*** |
| --- | --- | --- | --- | --- | --- | --- | --- | --- | --- | --- |
| **R(pr-b)** | 0.00 | 0.00 | 0.00 | 0.00 | 0.00 | 0.00 | 0.00 | 0.00 | 0.00 | 0.00 |
| **CR1(pr-b)** | -3.58 | -2.26 | -3.34 | -2.42 | -3.93 | -1.83 | 3.89 | 4.02 | -7.82 | -5.85 |
| **CR2(pr-b)** | -3.58 | -2.26 | -3.34 | -2.42 | -3.93 | -1.83 | 3.89 | 4.01 | -7.82 | -5.84 |
| **CR3(pr-b)** | -4.21 | -2.26 | -4.03 | -2.42 | -4.63 | -1.83 | 3.51 | 4.02 | -8.14 | -5.85 |
| **CR4(pr-b1)** | -4.46 | -2.22 | -4.11 | -2.34 | -4.70 | -1.75 | 2.96 | 2.93 | -7.65 | -4.68 |
| **CR4(pr-b2)** | -4.21 | -2.26 | -4.04 | -2.42 | -4.63 | -1.83 | 3.51 | 4.02 | -8.13 | -5.85 |
| **CR4(pr-b3)** | -4.21 | -2.26 | -4.04 | -2.42 | -4.63 | -1.83 | 3.51 | 4.02 | -8.14 | -5.85 |
| **TS1(pr-b)** | 15.35 | 16.74 | 15.30 | 16.06 | 14.70 | 16.65 | 22.74 | 24.12 | -8.03 | -7.47 |
| **TS2(pr-b)** | 10.50 | 11.17 | 10.43 | 10.60 | 9.84 | 11.19 | 18.30 | 18.72 | -8.46 | -7.53 |
| **TS3(pr-b)** | 12.15 | 13.22 | 12.05 | 12.54 | 11.45 | 13.14 | 20.18 | 21.29 | -8.73 | -8.15 |
| **TS4(pr-b1)** | 14.34 | 15.44 | 14.03 | 14.57 | 13.43 | 15.16 | 22.61 | 23.58 | -9.18 | -8.42 |
| **TS4(pr-b2)** | 13.78 | 15.52 | 13.33 | 14.71 | 12.74 | 15.30 | 22.37 | 23.62 | -9.63 | -8.31 |
| **TS4(pr-b3)** | 16.04 | 16.04 | 15.91 | 15.58 | 15.32 | 16.17 | 23.71 | 22.56 | -8.39 | -6.39 |
| **CP1(pr-b)** | 8.85 | 9.67 | 9.32 | 9.92 | 8.73 | 10.51 | 16.15 | 15.69 | -7.42 | -5.18 |
| **CP2(pr-b)** | -2.60 | -3.27 | -1.71 | -2.96 | -2.30 | -2.37 | 2.31 | 2.55 | -4.61 | -4.92 |
| **CP3(pr-b)** | 4.08 | 4.13 | 4.95 | 4.85 | 4.36 | 5.44 | 10.96 | 8.85 | -6.60 | -3.40 |
| **CP4(pr-b1)** | 4.82 | 4.29 | 5.34 | 4.75 | 4.75 | 5.34 | 12.39 | 9.80 | -7.65 | -4.46 |
| **CP4(pr-b2)** | 6.08 | 5.91 | 6.93 | 6.37 | 6.34 | 6.96 | 13.12 | 11.83 | -6.78 | -4.86 |
| **CP4(pr-b3)** | 8.18 | 7.71 | 9.58 | 8.64 | 8.99 | 9.24 | 12.75 | 12.03 | -3.76 | -2.79 |
| **P1(pr-b) (CH_3_CH_2_CH_2_O+NH_2_)** | 11.07 | 11.85 | 11.05 | 11.97 | 11.05 | 11.97 | 10.24 | 10.93 | 0.81 | 1.04 |
| **P2(pr-b) (CH_3_CH_2_CHOH+NH_2_)** | 4.06 | 2.00 | 4.34 | 2.33 | 4.34 | 2.33 | 3.04 | 0.88 | 1.30 | 1.44 |
| **P3(pr-b) (CH_3_CHCH_2_OH+NH_2_)** | 8.20 | 6.45 | 8.87 | 7.09 | 8.87 | 7.09 | 6.54 | 4.81 | 2.33 | 2.28 |
| **P4(pr-b) (CH_2_CH_2_CH_2_OH+NH_2_)** | 10.30 | 8.30 | 10.69 | 8.75 | 10.69 | 8.75 | 9.29 | 7.17 | 1.40 | 1.58 |

A and B refer to the M06-2X and CBS-QB3 methods, respectively.

**Table S36**. Relative energies and thermodynamic parameters for stationary points of the n-propanol-b + NH calculated at the CBS-QB3 method.

| **Species** | ***E(0K) (Hartree)*** | ***E˚(Hartree)*** | ***H˚(Hartree)*** | ***G˚(Hartree)*** |
| --- | --- | --- | --- | --- |
| **Propanol-b** | -193.9957 | -193.9892 | -193.9901 | -194.0234 |
| **CR1(pr-b)** | -249.1438 | -249.1342 | -249.1352 | -249.1788 |
| **CR2(pr-b)** | -249.1438 | -249.1342 | -249.1352 | -249.1788 |
| **CR3(pr-b)** | -249.1438 | -249.1342 | -249.1352 | -249.1788 |
| **CR4(pr-b1)** | -249.1437 | -249.1341 | -249.1351 | -249.1805 |
| **CR4(pr-b2)** | -249.1438 | -249.1342 | -249.1352 | -249.1788 |
| **CR4(pr-b3)** | -249.1438 | -249.1342 | -249.1352 | -249.1788 |
| **TS1(pr-b)** | -249.1135 | -249.1048 | -249.1057 | -249.1467 |
| **TS2(pr-b)** | -249.1224 | -249.1135 | -249.1144 | -249.1553 |
| **TS3(pr-b)** | -249.1191 | -249.1104 | -249.1113 | -249.1512 |
| **TS4(pr-b1)** | -249.1155 | -249.1072 | -249.1081 | -249.1476 |
| **TS4(pr-b2)** | -249.1154 | -249.1069 | -249.1079 | -249.1475 |
| **TS4(pr-b3)** | -249.1146 | -249.1055 | -249.1065 | -249.1492 |
| **CP1(pr-b)** | -249.1247 | -249.1146 | -249.1155 | -249.1602 |
| **CP2(pr-b)** | -249.1454 | -249.1351 | -249.1360 | -249.1811 |
| **CP3(pr-b)** | -249.1336 | -249.1226 | -249.1236 | -249.1711 |
| **CP4(pr-b1)** | -249.1333 | -249.1228 | -249.1238 | -249.1696 |
| **CP4(pr-b2)** | -249.1307 | -249.1202 | -249.1212 | -249.1663 |
| **CP4(pr-b3)** | -249.1279 | -249.1166 | -249.1175 | -249.1660 |
| **CH_3_CH_2_CH_2_O-b** | -193.3301 | -193.3239 | -193.3248 | -193.3582 |
| **CH_3_CH_2_CHOH-b** | -193.3458 | -193.3393 | -193.3402 | -193.3742 |
| **CH_3_CHCH_2_OH-b** | -193.3387 | -193.3317 | -193.3326 | -193.3680 |
| **CH_2_CH_2_CH_2_OH-b** | -193.3357 | -193.3290 | -193.3300 | -193.3642 |

**Table S37**. Relative energies and thermodynamic parameters for stationary points of the n-propanol-b + NH calculated at the M06-2X method.

| **Species** | ***(E+ZPE) (Hartree)*** | ***E˚(Hartree)*** | ***H˚(Hartree)*** | ***G˚(Hartree)*** | ***S˚(Hartree)*** |
| --- | --- | --- | --- | --- | --- |
| **Propanol-b** | -194.1584 | -194.1529 | -194.1520 | -194.1861 | 71.885 |
| **CR1(pr-b)** | -249.3543 | -249.3461 | -249.3452 | -249.3874 | 88.911 |
| **CR2(pr-b)** | -249.3543 | -249.3461 | -249.3452 | -249.3874 | 88.930 |
| **CR3(pr-b)** | -249.3553 | -249.3472 | -249.3463 | -249.3880 | 87.850 |
| **CR4(pr-b1)** | -249.3557 | -249.3473 | -249.3464 | -249.3889 | 89.472 |
| **CR4(pr-b2)** | -249.3553 | -249.3472 | -249.3463 | -249.3880 | 87.860 |
| **CR4(pr-b3)** | -249.3553 | -249.3472 | -249.3463 | -249.3880 | 87.857 |
| **TS1(pr-b)** | -249.3242 | -249.3164 | -249.3155 | -249.3574 | 88.206 |
| **TS2(pr-b)** | -249.3319 | -249.3242 | -249.3232 | -249.3644 | 86.763 |
| **TS3(pr-b)** | -249.3293 | -249.3216 | -249.3207 | -249.3614 | 85.866 |
| **TS4(pr-b1)** | -249.3258 | -249.3184 | -249.3175 | -249.3576 | 84.362 |
| **TS4(pr-b2)** | -249.3267 | -249.3196 | -249.3186 | -249.3580 | 82.829 |
| **TS4(pr-b3)** | -249.3231 | -249.3154 | -249.3145 | -249.3558 | 86.995 |
| **CP1(pr-b)** | -249.3345 | -249.3259 | -249.3250 | -249.3679 | 90.242 |
| **CP2(pr-b)** | -249.3528 | -249.3435 | -249.3426 | -249.3899 | 99.688 |
| **CP3(pr-b)** | -249.3421 | -249.3329 | -249.3320 | -249.3761 | 92.995 |
| **CP4(pr-b1)** | -249.3409 | -249.3323 | -249.3313 | -249.3739 | 89.496 |
| **CP4(pr-b2)** | -249.3389 | -249.3298 | -249.3288 | -249.3727 | 92.390 |
| **CP4(pr-b3)** | -249.3356 | -249.3255 | -249.3246 | -249.3733 | 102.526 |
| **CH_3_CH_2_CH_2_O-b** | -193.5017 | -193.4967 | -193.4958 | -193.5297 | 71.366 |
| **CH_3_CH_2_CHOH-b** | -193.5129 | -193.5074 | -193.5065 | -193.5412 | 72.995 |
| **CH_3_CHCH_2_OH-b** | -193.5063 | -193.5002 | -193.4993 | -193.5356 | 76.453 |
| **CH_2_CH_2_CH_2_OH-b** | -193.5029 | -193.4973 | -193.4964 | -193.5312 | 73.336 |

**Table S38**. The CCSD(T) absolute energies (in Hartree) and T1 diagnostic values for stationary points of the n-propanol-b + NH reaction calculated at the CCSD(T)/6-31+g(d**´**)//M06-2X level of theory.

| **Species** | ***CCSD(T)*** | ***T1 diagnostic*** |
| --- | --- | --- |
| **Propanol-b** | -193.7655 | 0.0104 |
| **CR1(pr-b)** | -248.8538 | 0.0107 |
| **CR2(pr-b)** | -248.8538 | 0.0107 |
| **CR3(pr-b)** | -248.8546 | 0.0107 |
| **CR4(pr-b1)** | -248.8544 | 0.0107 |
| **CR4(pr-b2)** | -248.8546 | 0.0107 |
| **CR4(pr-b3)** | -248.8546 | 0.0107 |
| **TS1(pr-b)** | -248.8155 | 0.0221 |
| **TS2(pr-b)** | -248.8165 | 0.0161 |
| **TS3(pr-b)** | -248.8132 | 0.0149 |
| **TS4(pr-b1)** | -248.8097 | 0.0150 |
| **TS4(pr-b2)** | -248.8102 | 0.0145 |
| **TS4(pr-b3)** | -248.8078 | 0.0144 |
| **CP1(pr-b)** | -248.8342 | 0.0163 |
| **CP2(pr-b)** | -248.8473 | 0.0144 |
| **CP3(pr-b)** | -248.8323 | 0.0126 |
| **CP4(pr-b1)** | -248.8328 | 0.0121 |
| **CP4(pr-b2)** | -248.8300 | 0.0119 |
| **CP4(pr-b3)** | -248.8255 | 0.0119 |
| **CH_3_CH_2_CH_2_O-b** | -193.1131 | 0.0164 |
| **CH_3_CH_2_CHOH-b** | -193.1174 | 0.0146 |
| **CH_3_CHCH_2_OH-b** | -193.1091 | 0.0129 |
| **CH_2_CH_2_CH_2_OH-b** | -193.1069 | 0.0120 |

**N-Propanol-c plus NH reaction**

**
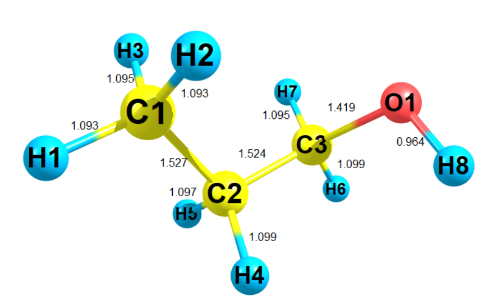

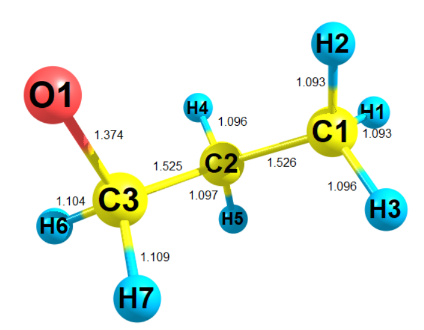
**
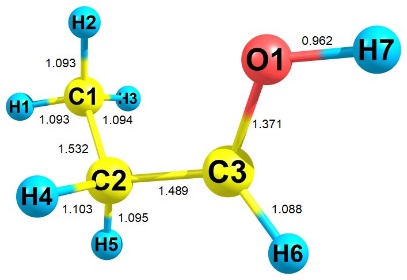


N-Propanol-c CH_3_CH_2_CH_2_O-c CH_3_CH_2_CHOH-c


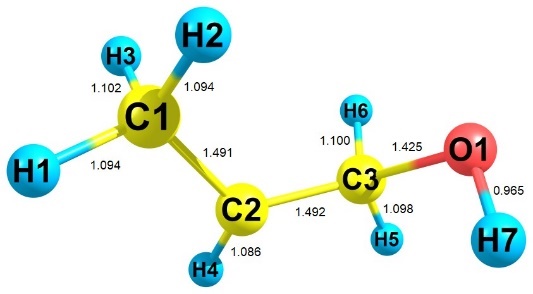

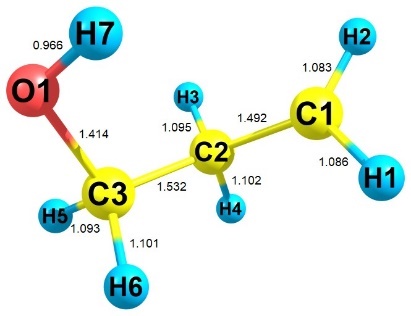

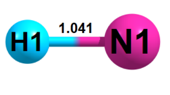

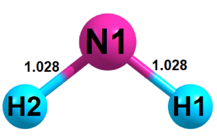


CH_3_CHCH_2_OH-c CH_2_CH_2_CH_2_OH-c NH NH_2_

**
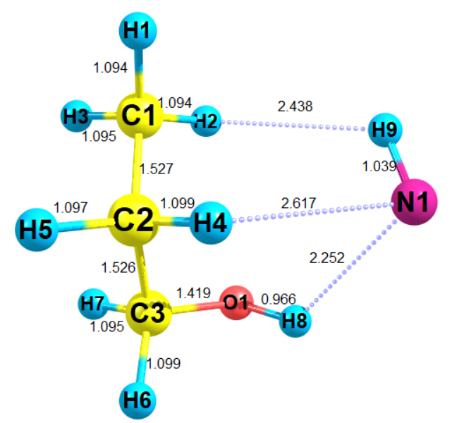
**
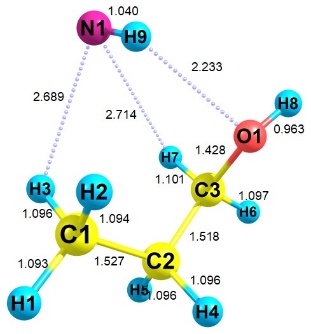


CR1(pr-c) CR2(pr-c1)

**
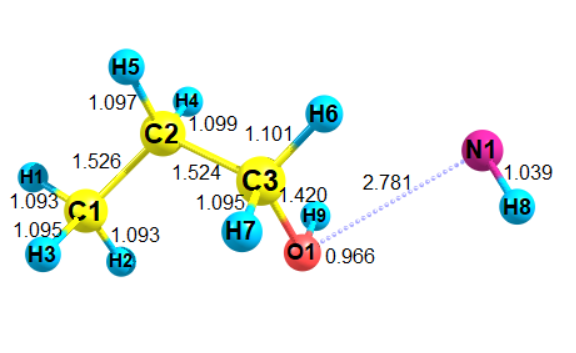
**
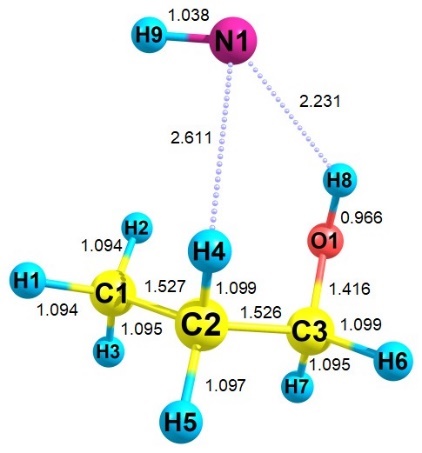


CR2(pr-c2) CR3(pr-c)

**
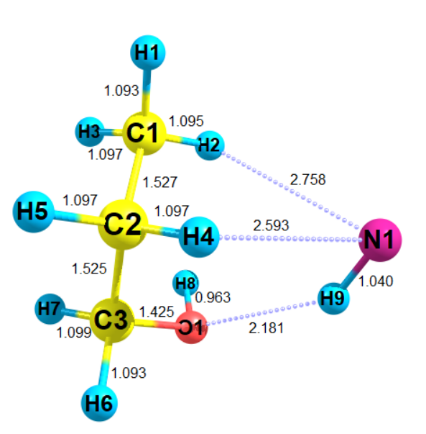

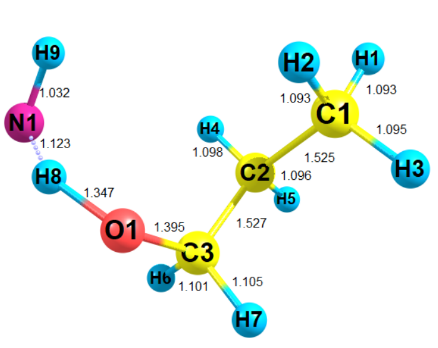
**
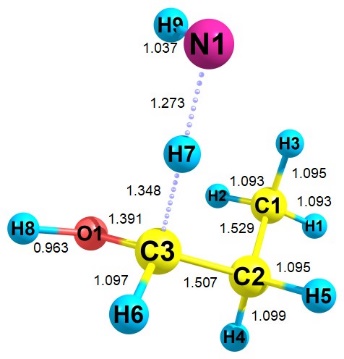


CR4(pr-c) TS1(pr-c) TS2(pr-c1)


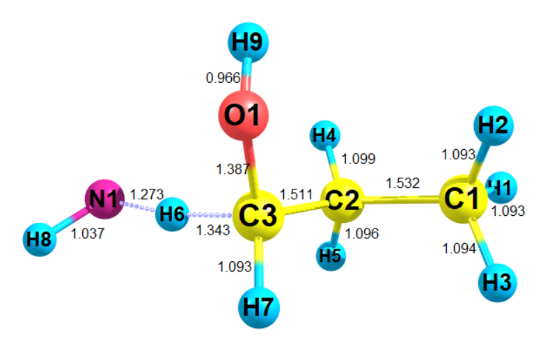


TS2(pr-c2)

**
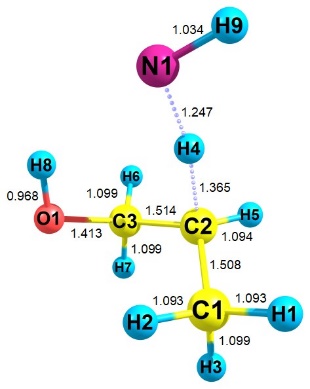
**
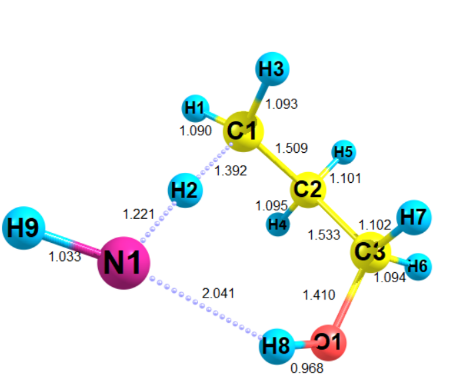
**
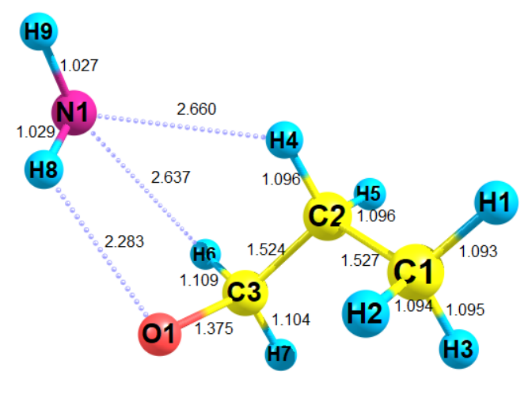
**

TS3(pr-c) TS4(pr-c) CP1(pr-c)


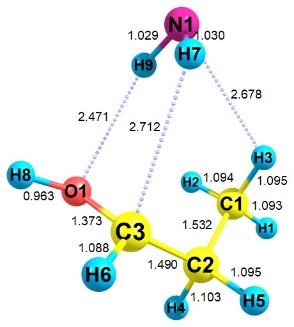
  **
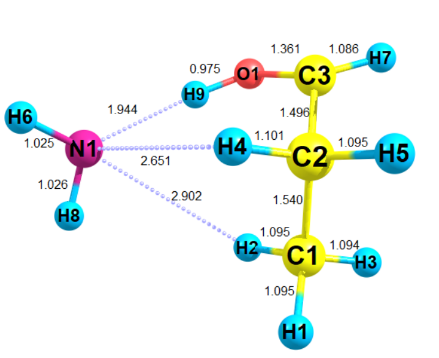
**

CP2 (pr-c1) CP2(pr-c)

**
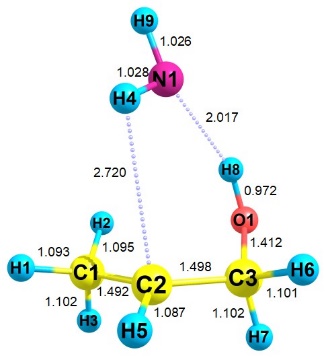
** **
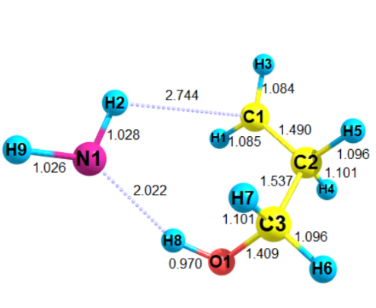
**

CP3(pr-c) CP4(pr-c)

**Figure S7**. Structures of all stationary points including bond lengths (in angstrom) in the n-C_3_H_7_OH + NH reaction calculated at the M06-2X method.


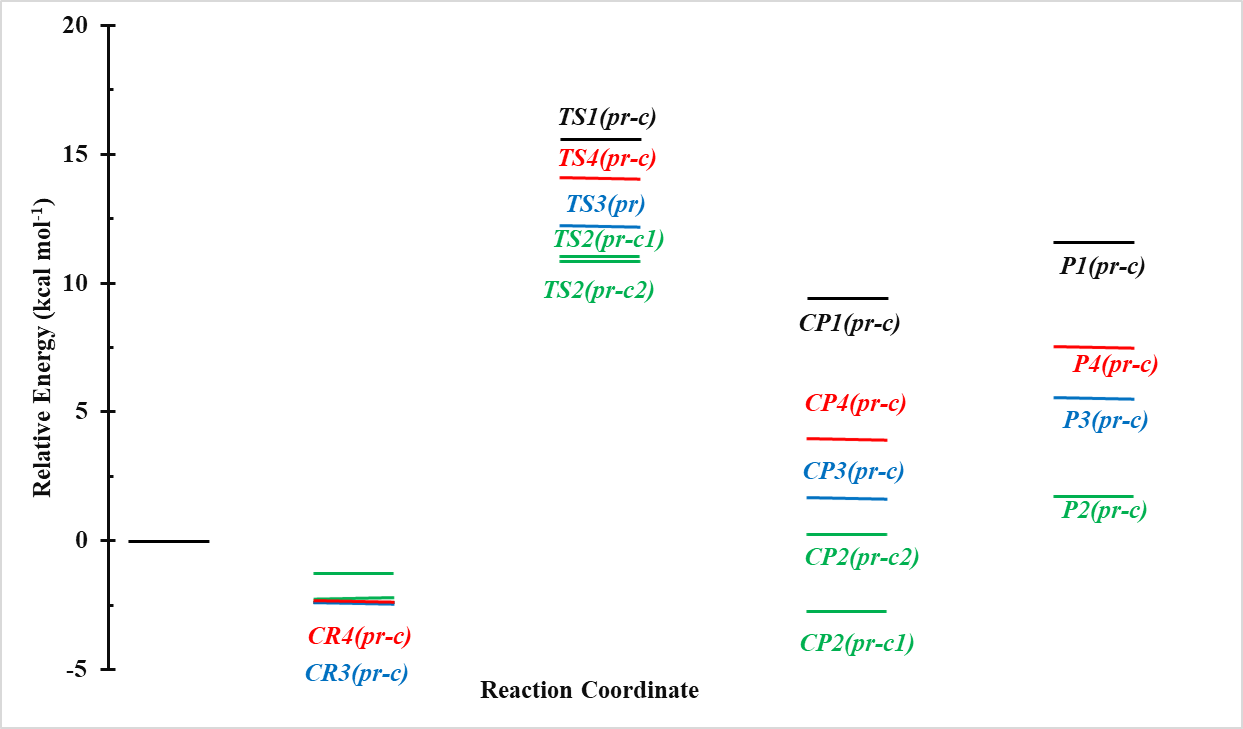


***R(pr-c)***

***CR1(pr-c)***

***CR2(pr-c1)***

***CR2(pr-c2)***

**Figure S8**. Potential energy surface of the n-propanol-c reaction at the triplet ground state computed by the CBS-QB3 level.

**Table S39**. Relative energies and thermodynamic parameters for stationary points of the n-propanol-c + NH. (Unit of all numbers is kcal mol^-1^)

| **Species** | ***∆(E+ZPE)(A)*** | ***∆E(0K)(B)*** | ***∆E˚(A)*** | ***∆E˚(B)*** | ***∆H˚(A)*** | ***∆H˚(B)*** | ***∆G˚(A)*** | ***∆G˚(B)*** | ***T∆S˚(A)*** | ***T∆S˚(B)*** |
| --- | --- | --- | --- | --- | --- | --- | --- | --- | --- | --- |
| **R(pr-c)** | 0.00 | 0.00 | 0.00 | 1.18 | 0.00 | -1.18 | 0.00 | 0.00 | 0.00 | -1.18 |
| **CR1(pr-c)** | -3.34 | -2.39 | -2.88 | -1.08 | -3.48 | -2.86 | 3.68 | 3.58 | -7.14 | -6.43 |
| **CR2(pr-c1)** | -4.10 | -1.31 | -3.78 | -0.02 | -4.37 | -1.80 | 3.33 | 4.66 | -7.68 | -6.46 |
| **CR2(pr-c2)** | -3.02 | -2.26 | -2.70 | -1.18 | -3.29 | -2.95 | 3.94 | 3.86 | -7.22 | -6.81 |
| **CR3(pr-c)** | -3.04 | -2.44 | -2.81 | -1.08 | -3.40 | -2.86 | 4.48 | 3.06 | -7.87 | -5.92 |
| **CR4(pr-c)** | -3.77 | -2.36 | -3.67 | -1.30 | -4.26 | -3.08 | 3.97 | 3.82 | -8.21 | -6.90 |
| **TS1(pr-c)** | 13.81 | 15.59 | 13.64 | 16.09 | 13.05 | 14.31 | 21.47 | 22.99 | -8.40 | -8.67 |
| **TS2(pr-c1)** | 9.86 | 10.99 | 9.77 | 11.69 | 9.17 | 9.91 | 17.79 | 18.32 | -8.60 | -8.40 |
| **TS2(pr-c2)** | 10.39 | 10.81 | 10.34 | 11.50 | 9.75 | 9.72 | 18.19 | 18.15 | -8.43 | -8.43 |
| **TS3(pr-c)** | 11.51 | 12.18 | 11.27 | 12.71 | 10.68 | 10.93 | 19.85 | 20.12 | -9.15 | -9.19 |
| **TS4(pr-c)** | 13.17 | 14.06 | 12.96 | 14.39 | 12.36 | 12.62 | 20.89 | 22.05 | -8.51 | -9.43 |
| **CP1(pr-c)** | 8.45 | 9.40 | 9.08 | 10.88 | 8.49 | 9.10 | 15.47 | 15.44 | -6.96 | -6.33 |
| **CP2(pr-c1)** | 0.71 | -2.80 | 1.49 | -1.24 | 0.90 | -3.02 | 7.65 | 2.90 | -6.74 | -5.92 |
| **CP2(pr-c2)** | -2.66 | 0.22 | -2.43 | 1.92 | -3.02 | 0.14 | 4.91 | 5.68 | -7.91 | -5.54 |
| **CP3(pr-c)** | 1.31 | 1.65 | 2.12 | 3.39 | 1.53 | 1.62 | 8.04 | 7.22 | -6.49 | -5.60 |
| **CP4(pr-c)** | 4.25 | 3.93 | 4.78 | 5.65 | 4.19 | 3.87 | 11.63 | 9.60 | -7.43 | -5.73 |
| **P1(pr-c) (CH_3_CH_2_CH_2_O+NH_2_)** | 10.68 | 11.59 | 10.72 | 12.95 | 10.73 | 10.58 | 9.81 | 10.61 | 0.94 | -0.03 |
| **P2(pr-c) (CH_3_CH_2_CHOH+NH_2_)** | 3.67 | 1.66 | 4.00 | 3.25 | 4.00 | 0.88 | 2.59 | 0.46 | 1.43 | 0.42 |
| **P3(pr-c) (CH_3_CHCH_2_OH+NH_2_)** | 7.01 | 5.51 | 7.64 | 7.25 | 7.64 | 4.89 | 5.20 | 4.03 | 2.46 | 0.86 |
| **P4(pr-c) (CH_2_CH_2_CH_2_OH+NH_2_)** | 9.11 | 7.51 | 9.58 | 9.19 | 9.58 | 6.82 | 7.82 | 6.22 | 1.77 | 0.60 |

A and B refer to the M06-2X and CBS-QB3 methods, respectively.

**Table S40**. Relative energies and thermodynamic parameters for stationary points of the n-propanol-c + NH calculated at the CBS-QB3 method.

| **Species** | ***E(0K) (Hartree)*** | ***E˚(Hartree)*** | ***H˚(Hartree)*** | ***G˚(Hartree)*** | ***S˚(Hartree)*** |
| --- | --- | --- | --- | --- | --- |
| **Propanol-c** | -193.9952 | -193.9898 | -193.9888 | -194.0228 |  |
| **CR1(pr-c)** | -249.1435 | -249.1345 | -249.1336 | -249.1788 |  |
| **CR2(pr-c1)** | -249.1418 | -249.1329 | -249.1319 | -249.1771 |  |
| **CR2(pr-c2)** | -249.1433 | -249.1347 | -249.1338 | -249.1784 |  |
| **CR3(pr-c)** | -249.1436 | -249.1346 | -249.1336 | -249.1797 |  |
| **CR4(pr-c)** | -249.1434 | -249.1349 | -249.1340 | -249.1785 |  |
| **TS1(pr-c)** | -249.1148 | -249.1072 | -249.1062 | -249.1479 |  |
| **TS2(pr-c1)** | -249.1222 | -249.1142 | -249.1133 | -249.1554 |  |
| **TS2(pr-c2)** | -249.1224 | -249.1145 | -249.1136 | -249.1556 |  |
| **TS3(pr-c)** | -249.1203 | -249.1126 | -249.1116 | -249.1525 |  |
| **TS4(pr-c)** | -249.1173 | -249.1099 | -249.1089 | -249.1494 |  |
| **CP1(pr-c)** | -249.1247 | -249.1155 | -249.1145 | -249.1599 |  |
| **CP2(pr-c1)** | -249.1441 | -249.1348 | -249.1339 | -249.1799 |  |
| **CP2(pr-c2)** | -249.1393 | -249.1298 | -249.1288 | -249.1755 |  |
| **CP3(pr-c)** | -249.1370 | -249.1274 | -249.1265 | -249.1730 |  |
| **CP4(pr-c)** | -249.1334 | -249.1238 | -249.1229 | -249.1692 |  |
| **CH_3_CH_2_CH_2_O-c** | -193.3300 | -193.3248 | -193.3238 | -193.3581 |  |
| **CH_3_CH_2_CHOH-c** | -193.3458 | -193.3402 | -193.3393 | -193.3743 |  |
| **CH_3_CHCH_2_OH-c** | -193.3397 | -193.3339 | -193.3329 | -193.3686 |  |
| **CH_2_CH_2_CH_2_OH-c** | -193.3365 | -193.3308 | -193.3298 | -193.3651 |  |

**Table S41**. Relative energies and thermodynamic parameters for stationary points of the n-propanol-c + NH calculated at the M06-2X method.

| **Species** | ***(E+ZPE) (Hartree)*** | ***E˚(Hartree)*** | ***H˚(Hartree)*** | ***G˚(Hartree)*** | ***S˚(Hartree)*** |
| --- | --- | --- | --- | --- | --- |
| **Propanol-c** | -194.1578 | -194.1524 | -194.1515 | -194.1854 | 71.469 |
| **CR1(pr-c)** | -249.3533 | -249.3449 | -249.3439 | -249.3871 | 90.789 |
| **CR2(pr-c1)** | -249.3546 | -249.3463 | -249.3453 | -249.3876 | 88.976 |
| **CR2(pr-c2)** | -249.3528 | -249.3446 | -249.3436 | -249.3866 | 90.522 |
| **CR3(pr-c)** | -249.3529 | -249.3447 | -249.3438 | -249.3858 | 88.335 |
| **CR4(pr-c)** | -249.3540 | -249.3461 | -249.3452 | -249.3866 | 87.182 |
| **TS1(pr-c)** | -249.3260 | -249.3185 | -249.3176 | -249.3587 | 86.542 |
| **TS2(pr-c1)** | -249.3323 | -249.3247 | -249.3238 | -249.3646 | 85.892 |
| **TS2(pr-c2)** | -249.3315 | -249.3238 | -249.3229 | -249.3639 | 86.466 |
| **TS3(pr-c)** | -249.3297 | -249.3223 | -249.3214 | -249.3613 | 84.041 |
| **TS4(pr-c)** | -249.3270 | -249.3196 | -249.3187 | -249.3596 | 86.191 |
| **CP1(pr-c)** | -249.3346 | -249.3258 | -249.3249 | -249.3683 | 91.368 |
| **CP2(pr-c1)** | -249.3469 | -249.3379 | -249.3370 | -249.3807 | 92.131 |
| **CP2(pr-c2)** | -249.3523 | -249.3441 | -249.3432 | -249.3851 | 88.191 |
| **CP3(pr-c)** | -249.3459 | -249.3369 | -249.3359 | -249.3801 | 92.945 |
| **CP4(pr-c)** | -249.3413 | -249.3327 | -249.3317 | -249.3744 | 89.812 |
| **CH_3_CH_2_CH_2_O-c** | -193.5017 | -193.4967 | -193.4958 | -193.5297 | 71.369 |
| **CH_3_CH_2_CHOH-c** | -193.5129 | -193.5074 | -193.5065 | -193.5412 | 73.028 |
| **CH_3_CHCH_2_OH-c** | -193.5076 | -193.5016 | -193.5007 | -193.5370 | 76.477 |
| **CH_2_CH_2_CH_2_OH-c** | -193.5042 | -193.4986 | -193.4976 | -193.5328 | 74.171 |

**Table S42**. The CCSD(T) absolute energies (in Hartree) and T1 diagnostic values for stationary points of the n-propanol-c + NH reaction calculated at the CCSD(T)/6-31+g(d**´**)//M06-2X level of theory.

| **Species** | ***CCSD(T)*** | ***T1 diagnostic*** |
| --- | --- | --- |
| **Propanol-c** | -193.7650 | 0.0105 |
| **CR1(pr-c)** | -248.8519 | 0.0112 |
| **CR2(pr-c1)** | -248.8533 | 0.0107 |
| **CR2(pr-c2)** | -248.8522 | 0.0112 |
| **CR3(pr-c)** | -248.8522 | 0.0110 |
| **CR4(pr-c)** | -248.8535 | 0.0108 |
| **TS1(pr-c)** | -248.8159 | 0.0220 |
| **TS2(pr-c1)** |  |  |
| **TS2(pr-c2)** | -248.8164 | 0.0162 |
| **TS3(pr-c)** |  |  |
| **TS4(pr-c)** | -248.8114 | 0.0148 |
| **CP1(pr-c)** | -248.8348 | 0.0162 |
| **CP2(pr-c1)** | -248.8379 | 0.0140 |
| **CP2(pr-c2)** | -248.8452 | 0.0145 |
| **CP3(pr-c)** | -248.8375 | 0.0127 |
| **CP4(pr-c)** | -248.8335 | 0.0124 |
| **CH_3_CH_2_CH_2_O-c** | -193.1174 | 0.0147 |
| **CH_3_CH_2_CHOH-c** | -193.1109 | 0.0131 |
| **CH_3_CHCH_2_OH-c** | -193.1130 | 0.0164 |
| **CH_2_CH_2_CH_2_OH-c** |  |  |

**N-Butanol-a plus NH reaction**


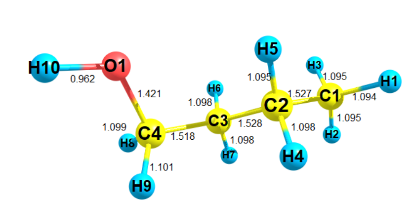

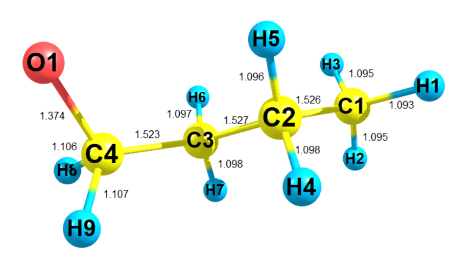

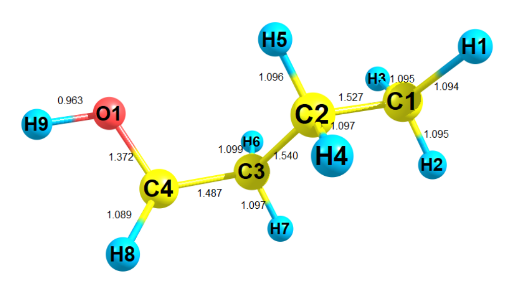


n-butanol-a CH_3_CH_2_CH_2_CH_2_O-a CH_3_CH_2_CH_2_CHOH-a


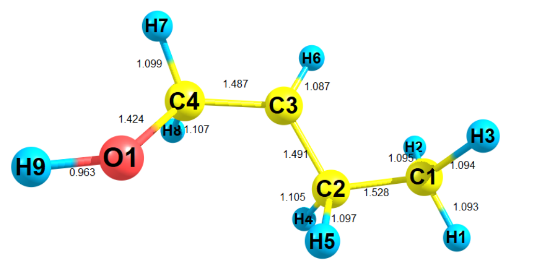

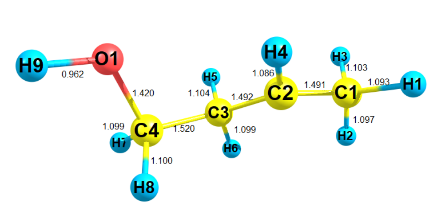

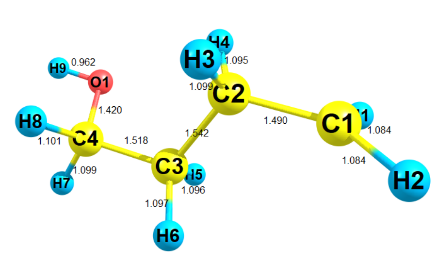


CH_3_CH_2_CHCH_2_OH-a CH_3_CHCH_2_CH_2_OH-a CH_2_CHCH_2_CH_2_OH-a


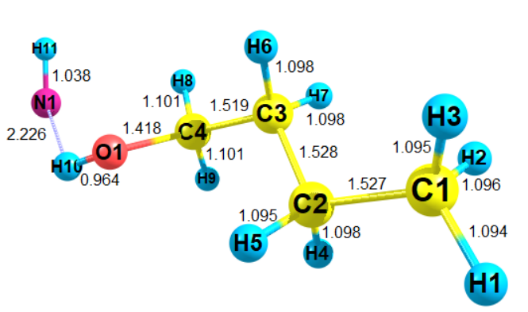

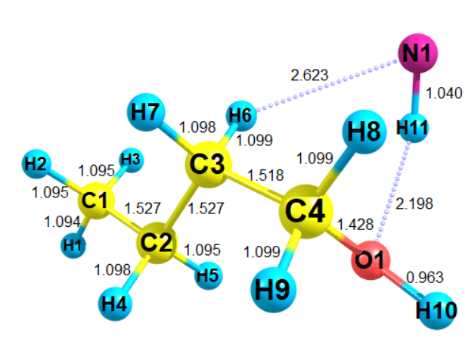

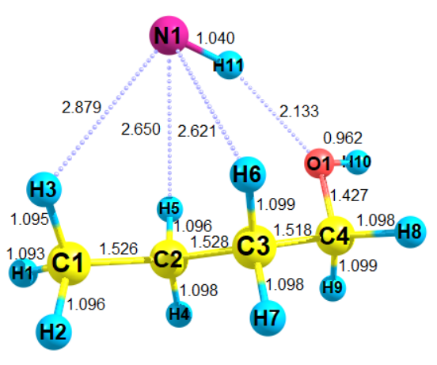


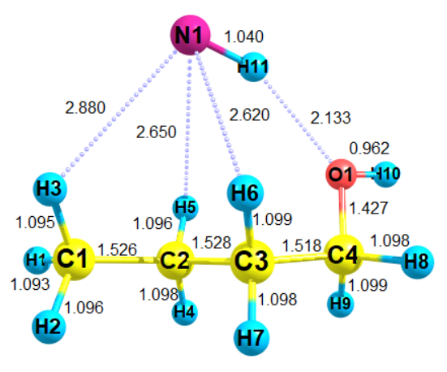
 CR1(bu-a) CR2(bu-a) CR3(bu-a)


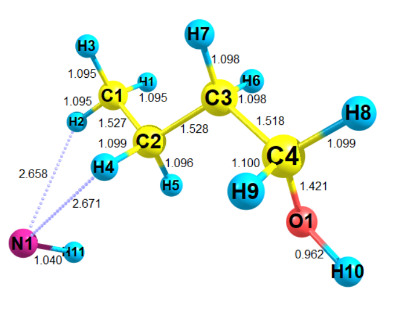

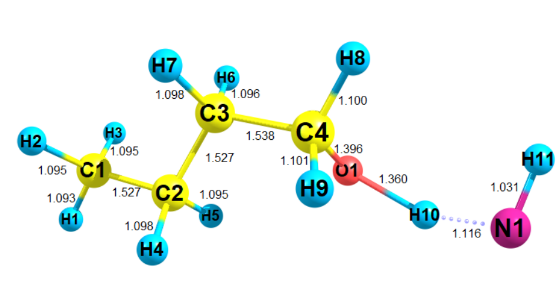


CR4(bu-a) CR5(bu-a) TS1(bu-a)

TS2(bu-a) TS3(bu-a) TS4(bu-a)

TS5(bu-a) CP1(bu-a) CP2 (bu-a)

CP3(bu-a) CP4(bu-a) CP5(bu-a)

**Figure S9**. Structures of all stationary points including bond lengths (in angstrom) in the n-C_4_H_9_OH + NH reaction calculated at the M06-2X/6-31+G(d,p) level of theory.

***R(bu-a)***

***CR1(bu-a)***

***CR2(bu-a)***

**Figure S10**. Potential energy surface of the n-butanol-a + NH reaction at the triplet ground state computed by the CBS-QB3 level.

**Table S43**. Relative energies and thermodynamic parameters for stationary points of the n-butanol-a + NH reaction. (Unit of all numbers is kcal mol^-1^)

| **Species** | ***∆(E+ZPE)(A)*** | ***∆E(0K)(B)*** | ***∆E˚(A)*** | ***∆E˚(B)*** | ***∆H˚(A)*** | ***∆H˚(B)*** | ***∆G˚(A)*** | ***∆G˚(B)*** | ***T∆S˚(A)*** | ***T∆S˚(B)*** |
| --- | --- | --- | --- | --- | --- | --- | --- | --- | --- | --- |
| **R(bu-a)** | 0.00 | 0.00 | 0.00 | 0.00 | 0.00 | 0.00 | 0.00 | 0.00 | 0.00 | 0.00 |
| **CR1(bu-a)** | -2.43 | -1.17 | -2.01 | -0.49 | -2.61 | -1.09 | 4.41 | 4.72 | -7.01 | -5.80 |
| **CR2(bu-a)** | -3.63 | -2.37 | -3.33 | -1.91 | -3.93 | -2.50 | 3.78 | 3.36 | -7.71 | -5.86 |
| **CR3(bu-a)** | -4.90 | -2.37 | -4.67 | -1.91 | -5.27 | -2.50 | 2.80 | 3.37 | -8.07 | -5.87 |
| **CR4(bu-a)** | -4.90 | -2.37 | -4.68 | -1.91 | -5.27 | -2.50 | 2.80 | 3.38 | -8.07 | -5.88 |
| **CR5(bu-a)** | -1.24 | -0.69 | -0.74 | 0.38 | -1.33 | -0.21 | 5.21 | 35.87 | -6.54 | -36.09 |
| **TS1(bu-a)** | 15.19 | 16.59 | 15.23 | 16.52 | 14.63 | 15.93 | 22.46 | 23.94 | -7.82 | -8.01 |
| **TS2(bu-a)** | 10.22 | 10.99 | 10.19 | 11.02 | 9.60 | 10.43 | 17.95 | 18.57 | -8.35 | -8.14 |
| **TS3(bu-a)** | 11.79 | 12.85 | 11.73 | 12.80 | 11.14 | 12.20 | 19.88 | 20.94 | -8.74 | -8.73 |
| **TS4(bu-a)** | 11.53 | 12.59 | 11.32 | 12.50 | 10.73 | 11.91 | 19.87 | 20.73 | -9.14 | -8.82 |
| **TS5(bu-a)** | 15.66 | 15.92 | 15.63 | 15.99 | 15.04 | 15.40 | 23.31 | 23.15 | -8.26 | -7.75 |
| **CP1(bu-a)** | 8.13 | 9.55 | 8.74 | 10.42 | 8.15 | 9.83 | 15.41 | 15.59 | -7.27 | -5.77 |
| **CP2(bu-a)** | -2.80 | -3.46 | -2.46 | -2.54 | -3.05 | -3.13 | 4.31 | 2.37 | -7.36 | -5.50 |
| **CP3(bu-a)** | 3.60 | 4.37 | 4.52 | 5.61 | 3.93 | 5.02 | 10.63 | 9.15 | -6.70 | -4.12 |
| **CP4(bu-a)** | 2.18 | 2.62 | 2.78 | 3.40 | 2.19 | 2.81 | 9.55 | 9.04 | -7.37 | -6.23 |
| **CP5(bu-a)** | 8.48 | 7.60 | 9.43 | 8.97 | 8.83 | 8.38 | 14.89 | 12.28 | -6.06 | -3.91 |
| **P1(bu-a)(CH_3_CH_2_CH_2_CH_2_O+NH_2_)** | 11.87 | 11.77 | 12.02 | 11.92 | 12.02 | 11.92 | 10.93 | 10.84 | 1.09 | 1.08 |
| **P2(bu-a)(CH_3_CH_2_CH_2_CHOH+NH_2_)** | 3.65 | 1.82 | 4.04 | 2.15 | 4.04 | 2.15 | 2.41 | 0.71 | 1.63 | 1.44 |
| **P3(bu-a)(CH_3_CH_2_CHCH_2_OH+NH_2_)** | 8.55 | 6.78 | 9.15 | 7.35 | 9.15 | 7.35 | 6.90 | 5.01 | 2.25 | 2.34 |
| **P4(bu-a)(CH_3_CHCH_2_CH_2_OH+NH_2_)** | 7.20 | 5.40 | 7.73 | 5.95 | 7.73 | 5.95 | 5.86 | 3.90 | 1.87 | 2.04 |
| **P5(bu-a)(CH_2_CH_2_CH_2_CH_2_OH+NH_2_)** | 10.32 | 8.23 | 10.80 | 8.87 | 10.80 | 8.87 | 9.13 | 6.26 | 1.67 | 2.61 |

A and B refer to the M06-2X and CBS-QB3 methods, respectively.

**Table S44**. Relative energies and thermodynamic parameters for stationary points of the n-butanol-a + NH reaction calculated at the CBS-QB3 level.

| **Species** | ***E(0K)( Hartree)*** | ***E˚(Hartree)*** | | ***H˚(Hartree)*** | ***G˚(Hartree)*** | ***S˚(cal/K mol)*** |
| --- | --- | --- | --- | --- | --- | --- |
| **n-butanol-a** | -233.2213 | -233.2145 | -233.2135 | | -233.2514 |  |
| **CR1(bu-a)** | -288.3677 | -288.3574 | -288.3565 | | -288.4057 |  |
| **CR2(bu-a)** | -288.3696 | -288.3597 | -288.3587 | | -288.4078 |  |
| **CR3(bu-a)** | -288.3696 | -288.3597 | -288.3587 | | -288.4078 |  |
| **CR4(bu-a)** | -288.3696 | -288.3597 | -288.3587 | | -288.4078 |  |
| **CR5(bu-a)** | -288.3669 | -288.3560 | -288.3551 | | -288.3560 |  |
| **TS1(bu-a)** | -288.3394 | -288.3303 | -288.3293 | | -288.3750 |  |
| **TS2(bu-a)** | -288.3483 | -288.3390 | -288.3381 | | -288.3836 |  |
| **TS3(bu-a)** | -288.3453 | -288.3362 | -288.3353 | | -288.3798 |  |
| **TS4(bu-a)** | -288.3458 | -288.3367 | -288.3358 | | -288.3801 |  |
| **TS5(bu-a)** | -288.3404 | -288.3311 | -288.3302 | | -288.3763 |  |
| **CP1(bu-a)** | -288.3506 | -288.3400 | -288.3391 | | -288.3883 |  |
| **CP2(bu-a)** | -288.3713 | -288.3607 | -288.3597 | | -288.4094 |  |
| **CP3(bu-a)** | -288.3589 | -288.3477 | -288.3467 | | -288.3986 |  |
| **CP4(bu-a)** | -288.3616 | -288.3512 | -288.3503 | | -288.3988 |  |
| **CP5(bu-a)** | -288.3537 | -288.3423 | -288.3414 | | -288.3936 |  |
| **CH_3_CH_2_CH_2_CH_2_O-a** | -232.5559 | -232.5493 | -232.5483 | | -232.5864 |  |
| **CH_3_CH_2_CH_2_CHOH-a** | -232.5717 | -232.5648 | -232.5639 | | -232.6025 |  |
| **CH_3_CH_2_CHCH_2_OH-a** | -232.5638 | -232.5565 | -232.5556 | | -232.5957 |  |
| **CH_3_CHCH_2_CH_2_OH-a** | -232.5660 | -232.5588 | -232.5578 | | -232.5974 |  |
| **CH_2_CH_2_CH_2_CH_2_OH-a** | -232.5615 | -232.5541 | -232.5532 | | -232.5937 |  |

**Table S45**. Relative energies and thermodynamic parameters for stationary points of the n-butanol-a + NH reaction calculated at the M06-2X method.

| **Species** | ***(E+ZPE)(Hartree)*** | ***E˚(Hartree)*** | ***H˚(Hartree)*** | ***G˚(Hartree)*** | ***S˚(cal/K mol)*** |
| --- | --- | --- | --- | --- | --- |
| **n-butanol-a** | -233.4250 | -233.4184 | -233.4174 | -233.4549 | 78.986 |
| **CR1(bu-a)** | -288.6191 | -288.6094 | -288.6085 | -288.6554 | 98.727 |
| **CR2(bu-a)** | -288.6210 | -288.6115 | -288.6106 | -288.6564 | 96.393 |
| **CR3(bu-a)** | -288.6231 | -288.6137 | -288.6127 | -288.6580 | 95.177 |
| **CR4(bu-a)** | -288.6231 | -288.6137 | -288.6127 | -288.6580 | 95.177 |
| **CR5(bu-a)** | -288.6172 | -288.6074 | -288.6065 | -288.6541 | 100.313 |
| **TS1(bu-a)** | -288.5910 | -288.5820 | -288.5810 | -288.6266 | 96.006 |
| **TS2(bu-a)** | -288.5990 | -288.5900 | -288.5890 | -288.6338 | 94.236 |
| **TS3(bu-a)** | -288.5965 | -288.5875 | -288.5866 | -288.6307 | 92.926 |
| **TS4(bu-a)** | -288.5969 | -288.5882 | -288.5872 | -288.6308 | 91.576 |
| **TS5(bu-a)** | -288.5903 | -288.5813 | -288.5804 | -288.6253 | 94.526 |
| **CP1(bu-a)** | -288.6023 | -288.5923 | -288.5914 | -288.6379 | 97.865 |
| **CP2(bu-a)** | -288.6197 | -288.6101 | -288.6092 | -288.6555 | 97.549 |
| **CP3(bu-a)** | -288.6095 | -288.5990 | -288.5981 | -288.6455 | 99.773 |
| **CP4(bu-a)** | -288.6118 | -288.6018 | -288.6009 | -288.6472 | 97.530 |
| **CP5(bu-a)** | -288.6017 | -288.5912 | -288.5903 | -288.6387 | 101.926 |
| **CH_3_CH_2_CH_2_CH_2_O-a** | -232.7670 | -232.7606 | -232.7597 | -232.7974 | 79.400 |
| **CH_3_CH_2_CH_2_CHOH-a** | -232.7801 | -232.7733 | -232.7724 | -232.8110 | 81.207 |
| **CH_3_CH_2_CHCH_2_OH-a** | -232.7723 | -232.7652 | -232.7642 | -232.8038 | 83.294 |
| **CH_3_CHCH_2_CH_2_OH-a** | -232.7745 | -232.7675 | -232.7665 | -232.8055 | 82.010 |
| **CH_2_CH_2_CH_2_CH_2_OH-a** | -232.7695 | -232.7626 | -232.7616 | -232.8003 | 81.347 |

**Table S46**. The CCSD(T) absolute energies (in Hartree) and T1 diagnostic values for stationary points of the n-butanol-a + NH reaction calculated at the CCSD(T)/6-31+g(d**´**)//M06-2X level of theory.

| **Species** | ***CCSD(T)*** | ***T1 diagnostic*** |
| --- | --- | --- |
| **n-butanol-a** | -232.9503 | 0.0103 |
| **CR1(bu-a)** | -288.0374 | 0.0107 |
| **CR2(bu-a)** | -288.0388 | 0.0105 |
| **CR3(bu-a)** | -288.0398 | 0.0105 |
| **CR4(bu-a)** | -288.0398 | 0.0105 |
| **CR5(bu-a)** | -288.0347 | 0.0105 |
| **TS1(bu-a)** | -288.0005 | 0.0207 |
| **TS2(bu-a)** | -288.0018 | 0.0154 |
| **TS3(bu-a)** | -287.9984 | 0.0143 |
| **TS4(bu-a)** | -287.9994 | 0.0141 |
| **TS5(bu-a)** | -287.9928 | 0.0139 |
| **CP1(bu-a)** | -288.0196 | 0.0157 |
| **CP2(bu-a)** | -288.0323 | 0.0138 |
| **CP3(bu-a)** | -288.0172 | 0.0124 |
| **CP4(bu-a)** | -288.0186 | 0.0122 |
| **CP5(bu-a)** | -288.0112 | 0.0117 |
| **CH_3_CH_2_CH_2_CH_2_O-a** | -232.2978 | 0.0152 |
| **CH_3_CH_2_CH_2_CHOH-a** | -232.3024 | 0.0138 |
| **CH_3_CH_2_CHCH_2_OH-a** | -232.2935 | 0.0125 |
| **CH_3_CHCH_2_CH_2_OH-a** | -232.2959 | 0.0122 |
| **CH_2_CH_2_CH_2_CH_2_OH-a** | -232.2708 | 0.0117 |

**N-Butanol-b plus NH reaction**

n-butanol-b CH_3_CH_2_CH_2_CH_2_O-b CH_3_CH_2_CH_2_CHOH-b

CH_3_CH_2_CHCH_2_OH-b CH_3_CHCH_2_CH_2_OH-b CH_2_CHCH_2_CH_2_OH-b

CR1(bu-b) CR2(bu-b) CR3(bu-b1)

CR3(bu-b2) CR4(bu-b) CR5(bu-b)

TS1(bu-b) TS2(bu-b) TS3(bu-b1)

TS3(bu-b2) TS4(bu-b) TS5(bu-b)

CP1(bu-b) CP2 (bu-b) CP3(bu-b1)

CP3(bu-b2) CP4(bu-b) CP5(bu-b)

**Figure S11**. Structures of all stationary points including bond lengths (in angstrom) in the n-butanol-b + NH reaction calculated at the M06-2X/6-31+G(d,p) level of theory.

***R(bu-b)***

***CR1(bu-b)***

***CR2(bu-b)***

**Figure S12**. Potential energy surface of the n-butanol-b + NH reaction at the triplet ground state computed by the CBS-QB3 level.

**Table S47**. Relative energies and thermodynamic parameters for stationary points of the n-butanol-b + NH reaction. (Unit of all numbers is kcal mol^-1^)

| **Species** | ***∆(E+ZPE)(A)*** | ***∆E(0K)(B)*** | ***∆E˚(A)*** | ***∆E˚(B)*** | ***∆H˚(A)*** | ***∆H˚(B)*** | ***∆G˚(A)*** | ***∆G˚(B)*** | ***T∆S˚(A)*** | ***T∆S˚(B)*** |
| --- | --- | --- | --- | --- | --- | --- | --- | --- | --- | --- |
| **R(bu-b)** | 0.00 | 0.00 | 0.00 | 1.18 | 0.00 | -1.18 | 0.00 | 0.00 | 0.00 | -1.18 |
| **CR1(bu-b)** | -3.93 | -2.13 | -3.54 | -1.09 | -4.13 | -2.87 | 3.28 | 4.08 | -7.41 | -6.95 |
| **CR2(bu-b)** | -3.52 | -2.42 | -3.30 | -1.37 | -3.89 | -3.14 | 3.98 | 3.52 | -7.87 | -6.67 |
| **CR3(bu-b1)** | -5.09 | -2.42 | -4.76 | -1.37 | -5.35 | -3.15 | 2.63 | 3.51 | -7.98 | -6.66 |
| **CR3(bu-b2)** | -2.16 | -1.32 | -1.65 | -0.06 | -2.24 | -1.84 | 4.84 | 4.65 | -7.09 | -6.49 |
| **CR4(bu-b)** | -4.81 | -2.41 | -4.65 | -1.35 | -5.24 | -3.13 | 2.95 | 3.31 | -8.19 | -6.44 |
| **CR5(bu-b)** | -5.09 | -2.41 | -4.76 | -1.36 | -5.35 | -3.14 | 2.62 | 3.54 | -7.98 | -6.68 |
| **TS1(bu-b)** | -3.19 | 16.55 | -3.08 | 17.09 | -3.67 | 15.31 | 4.56 | 23.88 | -8.23 | -8.57 |
| **TS2(bu-b)** | 10.33 | 11.08 | 10.23 | 11.74 | 9.64 | 9.96 | 18.14 | 18.49 | -8.51 | -8.54 |
| **TS3(bu-b1)** | 11.76 | 12.81 | 11.66 | 13.35 | 11.07 | 11.58 | 19.93 | 20.87 | -8.86 | -9.30 |
| **TS3(bu-b2)** | 13.66 | 13.73 | 13.62 | 14.57 | 13.03 | 12.79 | 21.46 | 20.89 | -8.43 | -8.10 |
| **TS4(bu-b)** | 11.20 | 12.53 | 10.75 | 13.05 | 10.16 | 11.27 | 19.62 | 20.61 | -9.46 | -9.34 |
| **TS5(bu-b)** | 14.89 | 15.48 | 14.76 | 16.06 | 14.17 | 14.28 | 22.65 | 22.84 | -8.48 | -8.56 |
| **CP1(bu-b)** | 8.52 | 9.14 | 9.22 | 10.56 | 8.63 | 8.78 | 15.26 | 15.31 | -6.64 | -6.53 |
| **CP2(bu-b)** | -2.73 | -3.51 | -2.43 | -1.99 | -3.02 | -3.77 | 4.39 | 2.35 | -7.42 | -6.12 |
| **CP3(bu-b1)** | 3.43 | 4.02 | 4.46 | 5.85 | 3.87 | 4.07 | 10.16 | 9.62 | -6.29 | -5.55 |
| **CP3(bu-b2)** | 6.19 | 5.43 | 7.19 | 7.63 | 6.60 | 5.85 | 12.67 | 9.69 | -6.07 | -3.83 |
| **CP4(bu-b)** | 2.62 | 2.51 | 3.38 | 4.35 | 2.79 | 2.58 | 9.89 | 8.24 | -7.10 | -5.67 |
| **CP5(bu-b)** | 5.73 | 5.54 | 6.10 | 7.40 | 5.51 | 5.62 | 13.62 | 10.66 | -8.11 | -5.04 |
| **P1(bu-b)(CH_3_CH_2_CH_2_CH_2_O+NH_2_)** | 11.98 | 11.74 | 12.06 | 13.08 | 12.06 | 10.71 | 11.13 | 10.80 | 0.93 | -0.09 |
| **P2(bu-b)(CH_3_CH_2_CH_2_CHOH+NH_2_)** | 3.76 | 1.77 | 4.08 | 3.30 | 4.08 | 0.93 | 2.61 | 0.65 | 1.47 | 0.28 |
| **P3(bu-b)(CH_3_CH_2_CHCH_2_OH+NH_2_)** | 8.61 | 6.73 | 9.18 | 8.50 | 9.18 | 6.13 | 6.99 | 4.94 | 2.19 | 1.19 |
| **P4(bu-b)(CH_3_CHCH_2_CH_2_OH+NH_2_)** | 7.30 | 5.36 | 7.76 | 7.10 | 7.76 | 4.73 | 6.05 | 3.83 | 1.71 | 0.89 |
| **P5(bu-b)(CH_2_CH_2_CH_2_CH_2_OH+NH_2_)** | 10.22 | 7.97 | 10.61 | 9.72 | 10.61 | 7.35 | 9.21 | 6.55 | 1.40 | 0.80 |

A and B refer to the M06-2X and CBS-QB3 methods, respectively.

**Table S48**. Relative energies and thermodynamic parameters for stationary points of the n-butanol-b + NH reaction calculated at the CBS-QB3 level.

| **Species** | ***E(0K)( Hartree)*** | ***E˚(Hartree)*** | | ***H˚(Hartree)*** | ***G˚(Hartree)*** |
| --- | --- | --- | --- | --- | --- |
| **butanol-b** | -233.2213 | -233.2144 | -233.2135 | | -233.2513 |
| **CR1(bu-b)** | -288.3691 | -288.3592 | -288.3583 | | -288.4066 |
| **CR2(bu-b)** | -288.3696 | -288.3597 | -288.3587 | | -288.4075 |
| **CR3(bu-b1)** | -288.3696 | -288.3597 | -288.3587 | | -288.4075 |
| **CR3(bu-b2)** | -288.3678 | -288.3576 | -288.3567 | | -288.4057 |
| **CR4(bu-b)** | -288.3696 | -288.3597 | -288.3587 | | -288.4078 |
| **CR5(bu-b)** | -288.3696 | -288.3597 | -288.3587 | | -288.4074 |
| **TS1(bu-b)** | -288.3394 | -288.3303 | -288.3293 | | -288.3750 |
| **TS2(bu-b)** | -288.3481 | -288.3388 | -288.3379 | | -288.3836 |
| **TS3(bu-b1)** | -288.3453 | -288.3362 | -288.3353 | | -288.3798 |
| **TS3(bu-b2)** | -288.3439 | -288.3343 | -288.3333 | | -288.3798 |
| **TS4(bu-b)** | -288.3458 | -288.3367 | -288.3358 | | -288.3802 |
| **TS5(bu-b)** | -288.3411 | -288.3319 | -288.3310 | | -288.3767 |
| **CP1(bu-b)** | -288.3512 | -288.3407 | -288.3397 | | -288.3887 |
| **CP2(bu-b)** | -288.3713 | -288.3607 | -288.3597 | | -288.4093 |
| **CP3(bu-b1)** | -288.3593 | -288.3482 | -288.3472 | | -288.3977 |
| **CP3(bu-b2)** | -288.3571 | -288.3453 | -288.3444 | | -288.3976 |
| **CP4(bu-b)** | -288.3617 | -288.3506 | -288.3496 | | -288.3999 |
| **CP5(bu-b)** | -288.3569 | -288.3457 | -288.3448 | | -288.3961 |
| **CH_3_CH_2_CH_2_CH_2_O -b** | -232.5558 | -232.5493 | -232.5483 | | -232.5863 |
| **CH_3_CH_2_CH_2_CHOH -b** | -232.5717 | -232.5648 | -232.5639 | | -232.6025 |
| **CH_3_CH_2_CHCH_2_OH-b** | -232.5638 | -232.5565 | -232.5556 | | -232.5957 |
| **CH_3_CHCH_2_CH_2_OH-b** | -232.5660 | -232.5588 | -232.5578 | | -232.5974 |
| **CH_2_CH_2_CH_2_CH_2_OH-b** | -232.5618 | -232.5546 | -232.5537 | | -232.5931 |

**Table S49**. Relative energies and thermodynamic parameters for stationary points of the n-butanol-b + NH reaction calculated at the M06-2X method.

| **Species** | ***(E+ZPE)(Hartree)*** | ***E˚(Hartree)*** | ***H˚(Hartree)*** | ***G˚(Hartree)*** | ***S˚(cal/K mol)*** |
| --- | --- | --- | --- | --- | --- |
| **butanol-b** | -233.4252 | -233.4184 | -233.4175 | -233.4553 | 79.523 |
| **CR1(bu-b)** | -288.6217 | -288.6119 | -288.6110 | -288.6575 | 97.919 |
| **CR2(bu-b)** | -288.6210 | -288.6115 | -288.6106 | -288.6564 | 96.393 |
| **CR3(bu-b1)** | -288.6235 | -288.6139 | -288.6129 | -288.6585 | 96.012 |
| **CR3(bu-b2)** | -288.6189 | -288.6089 | -288.6080 | -288.6550 | 99.014 |
| **CR4(bu-b)** | -288.6231 | -288.6137 | -288.6127 | -288.6580 | 95.303 |
| **CR5(bu-b)** | -288.6235 | -288.6139 | -288.6129 | -288.6586 | 96.017 |
| **TS1(bu-b)** | -288.6205 | -288.6112 | -288.6102 | -288.6555 | 95.161 |
| **TS2(bu-b)** | -288.5990 | -288.5900 | -288.5890 | -288.6338 | 94.243 |
| **TS3(bu-b1)** | -288.5967 | -288.5877 | -288.5868 | -288.6310 | 93.055 |
| **TS3(bu-b2)** | -288.5936 | -288.5846 | -288.5836 | -288.6285 | 94.498 |
| **TS4(bu-b)** | -288.5976 | -288.5891 | -288.5882 | -288.6315 | 91.057 |
| **TS5(bu-b)** | -288.5917 | -288.5828 | -288.5818 | -288.6266 | 94.340 |
| **CP1(bu-b)** | -288.6018 | -288.5916 | -288.5907 | -288.6384 | 100.518 |
| **CP2(bu-b)** | -288.6198 | -288.6102 | -288.6092 | -288.6557 | 97.895 |
| **CP3(bu-b1)** | -288.6099 | -288.5992 | -288.5982 | -288.6465 | 101.673 |
| **CP3(bu-b2)** | -288.6056 | -288.5948 | -288.5939 | -288.6425 | 102.414 |
| **CP4(bu-b)** | -288.6112 | -288.6009 | -288.6000 | -288.6470 | 98.971 |
| **CP5(bu-b)** | -288.6063 | -288.5966 | -288.5956 | -288.6410 | 95.593 |
| **CH_3_CH_2_CH_2_CH_2_O -b** | -232.7670 | -232.7606 | -232.7597 | -232.7974 | 79.399 |
| **CH_3_CH_2_CH_2_CHOH -b** | -232.7801 | -232.7733 | -232.7724 | -232.8110 | 81.207 |
| **CH_3_CH_2_CHCH_2_OH-b** | -232.7724 | -232.7652 | -232.7643 | -232.8040 | 83.623 |
| **CH_3_CHCH_2_CH_2_OH-b** | -232.7745 | -232.7675 | -232.7665 | -232.8055 | 82.009 |
| **CH_2_CH_2_CH_2_CH_2_OH-b** | -232.7698 | -232.7629 | -232.7620 | -232.8005 | 80.981 |

**Table S50**. The CCSD(T) absolute energies (in Hartree) and T1 diagnostic values for stationary points of the n-butanol-b + NH reaction calculated at the CCSD(T)/6-31+g(d**´**)//M06-2X level of theory.

| **Species** | ***CCSD(T)*** | ***T1 diagnostic*** |
| --- | --- | --- |
| **butanol-b** | -232.9503 | 0.0103 |
| **CR1(bu-b)** | -288.0386 | 0.0106 |
| **CR2(bu-b)** | -288.0388 | 0.0105 |
| **CR3(bu-b1)** | -288.0398 | 0.0105 |
| **CR3(bu-b2)** | -288.0346 | 0.0106 |
| **CR4(bu-b)** | -288.0398 | 0.0105 |
| **CR5(bu-b)** | -288.0398 | 0.0105 |
| **TS1(bu-b)** | -288.0004 | 0.0207 |
| **TS2(bu-b)** | -288.0018 | 0.0154 |
| **TS3(bu-b1)** | -287.9984 | 0.0143 |
| **TS3(bu-b2)** | -287.9957 | 0.0142 |
| **TS4(bu-b)** | -287.9992 | 0.0141 |
| **TS5(bu-b)** | -287.9932 | 0.0139 |
| **CP1(bu-b)** | -288.0198 | 0.0154 |
| **CP2(bu-b)** | -288.0324 | 0.0138 |
| **CP3(bu-b1)** | -288.0172 | 0.0124 |
| **CP3(bu-b2)** | -288.0134 | 0.0123 |
| **CP4(bu-b)** | -288.0196 | 0.0120 |
| **CP5(bu-b)** | -288.0143 | 0.0116 |
| **CH_3_CH_2_CH_2_CH_2_O-b** | -232.2978 | 0.0152 |
| **CH_3_CH_2_CH_2_CHOH-b** | -232.3024 | 0.0138 |
| **CH_3_CH_2_CHCH_2_OH-b** | -232.2935 | 0.0125 |
| **CH_3_CHCH_2_CH_2_OH-b** | -232.2959 | 0.0122 |
| **CH_2_CH_2_CH_2_CH_2_OH-b** | -232.2918 | 0.0116 |

**N-Butanol-c plus NH reaction**

n-butanol-c CH_3_CH_2_CH_2_CH_2_O-c CH_3_CH_2_CH_2_CHOH-c

CH_3_CH_2_CHCH_2_OH-c CH_3_CHCH_2_CH_2_OH-c CH_2_CHCH_2_CH_2_OH-c

CR1(bu-c) CR2(bu-c1) CR2(bu-c2)

CR3(bu-c) CR4(bu-c) CR5(bu-c)

TS1(bu-c) TS2(bu-c) TS2(bu-c1)

TS3(bu-c) TS4(bu-c) TS5(bu-c)

CP1(bu-c) CP2 (bu-c1) CP2 (bu-c2)

CP3(bu-c) CP4(bu-c) CP5(bu-c)

**Figure S13**. Structures of all stationary points including bond lengths (in angstrom) in the n-butanol-c + NH reaction calculated at the M06-2X/6-31+G(d,p) level of theory.

***R(bu-c)***

***CR1(bu-c)***

***CR2(bu-c1)***

**Figure S14**. Potential energy surface of the n-butanol-c + NH reaction at the triplet ground state computed by the CBS-QB3 level.

**Table S51**. Relative energies and thermodynamic parameters for stationary points of the n-butanol-c + NH reaction. (Unit of all numbers is kcal mol^-1^)

| **Species** | ***∆(E+ZPE)(A)*** | ***∆E(0K)(B)*** | ***∆E˚(A)*** | ***∆E˚(B)*** | ***∆H˚(A)*** | ***∆H˚(B)*** | ***∆G˚(A)*** | ***∆G˚(B)*** | ***T∆S˚(A)*** | ***T∆S˚(B)*** |
| --- | --- | --- | --- | --- | --- | --- | --- | --- | --- | --- |
| **R(bu-c)** | 0.00 | 0.00 | 0.00 | 0.00 | 0.00 | 0.00 | 0.00 | 0.00 | 0.00 | 0.00 |
| **CR1(bu-c)** | -3.52 | -2.48 | -3.28 | -1.73 | -3.87 | -2.32 | 4.26 | 3.38 | -8.13 | -5.70 |
| **CR2(bu-c1)** | -1.23 | -2.46 | -0.75 | -1.90 | -1.35 | -2.49 | 6.00 | 3.57 | -7.34 | -6.06 |
| **CR2(bu-c2)** | -3.29 | -1.34 | -3.26 | -0.63 | -3.85 | -1.22 | 4.62 | 4.37 | -8.47 | -5.59 |
| **CR3(bu-c)** | -2.20 | -1.34 | -1.76 | -0.67 | -2.35 | -1.26 | 5.01 | 4.02 | -7.36 | -5.28 |
| **CR4(bu-c)** | -3.81 | -2.72 | -3.20 | -2.21 | -3.80 | -2.80 | 2.63 | 3.27 | -6.43 | -6.07 |
| **CR5(bu-c)** |  |  |  |  |  |  |  |  |  |  |
| **TS1(bu-c)** | 14.19 | 15.48 | 13.87 | 15.41 | 13.27 | 14.82 | 22.27 | 22.81 | -9.00 | -8.00 |
| **TS2(bu-c1)** | 10.61 | 10.67 | 10.54 | 10.80 | 9.95 | 10.21 | 18.71 | 18.09 | -8.77 | -7.88 |
| **TS2(bu-c2)** | 10.29 | 10.79 | 10.24 | 11.50 | 9.64 | 9.72 | 18.13 | 18.16 | -8.49 | -8.45 |
| **TS3(bu-c)** | 13.80 | 13.48 | 13.81 | 13.75 | 13.22 | 13.16 | 21.79 | 20.73 | -8.57 | -7.57 |
| **TS4(bu-c)** | 10.79 | 12.20 | 10.54 | 12.11 | 9.94 | 11.52 | 19.45 | 20.39 | -9.50 | -8.87 |
| **TS5(bu-c)** |  |  |  |  |  |  |  |  |  |  |
| **CP1(bu-c)** | 7.98 | 8.92 | 8.26 | 9.77 | 7.67 | 9.18 | 15.38 | 15.17 | -7.71 | -5.99 |
| **CP2(bu-c1)** | 0.79 | 0.12 | 1.31 | 1.37 | 0.72 | 0.78 | 7.74 | 5.26 | -7.02 | -4.48 |
| **CP2(bu-c2)** | -3.03 | -3.10 | -2.90 | -2.13 | -3.49 | -2.72 | 5.00 | 2.73 | -8.49 | -5.45 |
| **CP3(bu-c)** | 6.59 | 5.15 | 7.54 | 6.82 | 6.95 | 6.22 | 13.08 | 9.30 | -6.13 | -3.08 |
| **CP4(bu-c)** | 1.43 | 1.08 | 2.08 | 2.34 | 1.49 | 1.74 | 8.84 | 6.06 | -7.35 | -4.31 |
| **CP5(bu-c)** |  |  |  |  |  |  |  |  |  |  |
| **P1(bu-c)(CH_3_CH_2_CH_2_CH_2_O+NH_2_)** | 11.73 | 11.48 | 11.85 | 11.68 | 11.85 | 11.68 | 10.94 | 10.54 | 0.91 | 1.14 |
| **P2(bu-c)(CH_3_CH_2_CH_2_CHOH+NH_2_)** | 3.62 | 1.65 | 4.12 | 2.10 | 4.12 | 2.10 | 2.24 | 0.38 | 1.88 | 1.72 |
| **P3(bu-c)(CH_3_CH_2_CHCH_2_OH+NH_2_)** | 8.37 | 6.46 | 8.97 | 7.10 | 8.97 | 7.10 | 6.80 | 4.62 | 2.17 | 2.48 |
| **P4(bu-c)(CH_3_CHCH_2_CH_2_OH+NH_2_)** | 6.95 | 5.10 | 7.48 | 5.70 | 7.48 | 5.70 | 5.71 | 3.56 | 1.77 | 2.14 |
| **P5(bu-c)(CH_2_CH_2_CH_2_CH_2_OH+NH_2_)** |  |  |  |  |  |  |  |  |  |  |

A and B refer to the M06-2X and CBS-QB3 methods, respectively.

**Table S52**. Absolute energies and thermodynamic parameters for stationary points of the n-butanol-b + NH reaction calculated at the CBS-QB3 level.

| **Species** | ***E(0K)( Hartree)*** | ***E˚(Hartree)*** | | ***H˚(Hartree)*** | ***G˚(Hartree)*** |
| --- | --- | --- | --- | --- | --- |
| **butanol-c** | -233.2208 | -233.2141 | -233.2131 | | -233.2508 |
| **CR1(bu-c)** | -288.3692 | -288.3589 | -288.3580 | | -288.4072 |
| **CR2(bu-c1)** | -288.3692 | -288.3592 | -288.3583 | | -288.4069 |
| **CR2(bu-c2)** | -288.3674 | -288.3572 | -288.3562 | | -288.4056 |
| **CR3(bu-c)** | -288.3674 | -288.3572 | -288.3563 | | -288.4061 |
| **CR4(bu-c)** | -288.3696 | -288.3597 | -288.3588 | | -288.4073 |
| **CR5(bu-c)** |  |  |  | |  |
| **TS1(bu-c)** | -288.3406 | -288.3316 | -288.3307 | | -288.3762 |
| **TS2(bu-c1)** | -288.3483 | -288.3390 | -288.3380 | | -288.3837 |
| **TS2(bu-c2)** | -288.3481 | -288.3379 | -288.3388 | | -288.3836 |
| **TS3(bu-c)** | -288.3438 | -288.3343 | -288.3333 | | -288.3795 |
| **TS4(bu-c)** | -288.3458 | -288.3369 | -288.3359 | | -288.3800 |
| **TS5(bu-c)** |  |  |  | |  |
| **CP1(bu-c)** | -288.3511 | -288.3406 | -288.3397 | | -288.3884 |
| **CP2(bu-c1)** | -288.3651 | -288.3540 | -288.3530 | | -288.4042 |
| **CP2(bu-c2)** | -288.3702 | -288.3596 | -288.3586 | | -288.4082 |
| **CP3(bu-c)** | -288.3571 | -288.3453 | -288.3444 | | -288.3977 |
| **CP4(bu-c)** | -288.3636 | -288.3525 | -288.3515 | | -288.4029 |
| **CP5(bu-c)** |  |  |  | |  |
| **CH_3_CH_2_CH_2_CH_2_O-c** | -232.5558 | -232.5492 | -232.5483 | | -232.5862 |
| **CH_3_CH_2_CH_2_CHOH-c** | -232.5715 | -232.5645 | -232.5635 | | -232.6024 |
| **CH_3_CH_2_CHCH_2_OH-c** | -232.5638 | -232.5565 | -232.5556 | | -232.5957 |
| **CH_3_CHCH_2_CH_2_OH-c** | -232.5660 | -232.5587 | -232.5578 | | -232.5974 |
| **CH_2_CH_2_CH_2_CH_2_OH-c** |  |  |  | |  |

**Table S53**. Relative energies and thermodynamic parameters for stationary points of the n-butanol-c + NH reaction calculated at the M06-2X method.

| Species | E(0K)( Hartree) | E˚(Hartree) | H˚(Hartree) | G˚(Hartree) | S˚(cal/K mol) |
| --- | --- | --- | --- | --- | --- |
| butanol-c | -233.4248 | -233.4181 | -233.4171 | -233.4550 | 79.589 |
| CR1(bu-c) | -288.6206 | -288.6112 | -288.6102 | -288.6556 | 95.575 |
| CR2(bu-c1) | -288.6170 | -288.6072 | -288.6062 | -288.6529 | 98.218 |
| CR2(bu-c2) | -288.6203 | -288.6112 | -288.6102 | -288.6551 | 94.430 |
| CR3(bu-c) | -288.6185 | -288.6088 | -288.6078 | -288.6544 | 98.158 |
| CR4(bu-c) | -288.6211 | -288.6111 | -288.6101 | -288.6582 | 101.291 |
| CR5(bu-c) |  |  |  |  |  |
| TS1(bu-c) | -288.5924 | -288.5839 | -288.5829 | -288.6269 | 92.673 |
| TS2(bu-c1) | -288.5981 | -288.5892 | -288.5882 | -288.6326 | 93.443 |
| TS2(bu-c2) | -288.5986 | -288.5896 | -288.5887 | -288.6335 | 94.375 |
| TS3(bu-c) | -288.5930 | -288.5839 | -288.5830 | -288.6277 | 94.105 |
| TS4(bu-c) | -288.5978 | -288.5892 | -288.5882 | -288.6314 | 90.971 |
| TS5(bu-c) |  |  |  |  |  |
| CP1(bu-c) | -288.6023 | -288.5928 | -288.5918 | -288.6379 | 96.980 |
| CP2(bu-c1) | -288.6138 | -288.6039 | -288.6029 | -288.6501 | 99.297 |
| CP2(bu-c2) | -288.6199 | -288.6106 | -288.6096 | -288.6545 | 94.378 |
| CP3(bu-c) | -288.6045 | -288.5939 | -288.5930 | -288.6416 | 102.284 |
| CP4(bu-c) | -288.6127 | -288.6026 | -288.6017 | -288.6483 | 98.192 |
| CP5(bu-c) |  |  |  |  |  |
| CH3CH2CH2CH2O-c | -232.7670 | -232.7606 | -232.7597 | -232.7974 | 79.400 |
| CH3CH2CH2CHOH-c | -232.7800 | -232.7729 | -232.7720 | -232.8113 | 82.641 |
| CH3CH2CHCH2OH-c | -232.7724 | -232.7652 | -232.7643 | -232.8040 | 83.622 |
| CH3CHCH2CH2OH-c | -232.7746 | -232.7676 | -232.7666 | -232.8057 | 82.270 |
| CH2CH2CH2CH2OH-c |  |  |  |  |  |

**Table S54**. The CCSD(T) absolute energies (in Hartree) and T1 diagnostic values for stationary points of the n-butanol-c + NH reaction calculated at the CCSD(T)/6-31+g(d**´**)//M06-2X level of theory.

| **Species** | ***CCSD(T)*** | ***T1 diagnostic*** |
| --- | --- | --- |
| **butanol-c** | -232.9498 | 0.0103 |
| **CR1(bu-c)** | -288.0362 | 0.0109 |
| **CR2(bu-c1)** | -288.0335 | 0.0105 |
| **CR2(bu-c2)** | -288.0370 | 0.0109 |
| **CR3(bu-c)** | -288.0346 | 0.0106 |
| **CR4(bu-c)** | -288.0218 | 0.0122 |
| **CR5(bu-c)** |  |  |
| **TS1(bu-c)** | -288.0008 | 0.0206 |
| **TS2(bu-c1)** | -287.9997 | 0.0154 |
| **TS2(bu-c2)** | -288.0016 | 0.0154 |
| **TS3(bu-c)** | -287.9948 | 0.0141 |
| **TS4(bu-c)** | -287.9990 | 0.0145 |
| **TS5(bu-c)** |  |  |
| **CP1(bu-c)** | -288.0198 | 0.0155 |
| **CP2(bu-c1)** | -288.0215 | 0.0134 |
| **CP2(bu-c2)** | -288.0298 | 0.0138 |
| **CP3(bu-c)** | -288.0128 | 0.0122 |
| **CP4(bu-c)** | -288.0387 | 0.0107 |
| **CP5(bu-c)** |  |  |
| **CH_3_CH_2_CH_2_CH_2_O-c** | -232.2978 | 0.0152 |
| **CH_3_CH_2_CH_2_CHOH-c** | -232.3024 | 0.0138 |
| **CH_3_CH_2_CHCH_2_OH-c** | -232.2937 | 0.0124 |
| **CH_3_CHCH_2_CH_2_OH-c** | -232.2955 | 0.0122 |
| **CH_2_CH_2_CH_2_CH_2_OH-c** |  |  |

**N-Butanol-d plus NH reaction**

n-butanol-d CH_3_CH_2_CH_2_CH_2_O-d CH_3_CH_2_CH_2_CHOH-d

CH_3_CH_2_CHCH_2_OH-d CH_3_CHCH_2_CH_2_OH-d CH_2_CHCH_2_CH_2_OH-d

CR1(bu-d) CR2(bu-d) CR3(bu-d)

CR4(bu-d) CR5(bu-d) TS1(bu-d)

TS2(bu-d) TS3(bu-d) TS4(bu-d)

TS5(bu-d) CP1(bu-d) CP2 (bu-d)

CP3(bu-d) CP4(bu-d) CP5(bu-d)

**Figure S15**. Structures of all stationary points including bond lengths (in angstrom) in the n-butanol-d + NH reaction calculated at the M06-2X/6-31+G(d,p) level of theory.

***R(bu-d)***

***CR1(bu-d)***

***CR2(bu-d)***

**Figure S16**. Potential energy surface of the n-butanol-d + NH reaction at the triplet ground state computed by the CBS-QB3 level.

**Table S55**. Relative energies and thermodynamic parameters for stationary points of the n-butanol-c + NH reaction. (Unit of all numbers is kcal mol^-1^)

| **Species** | ***∆(E+ZPE)(A)*** | ***∆E(0K)(B)*** | ***∆E˚(A)*** | ***∆E˚(B)*** | ***∆H˚(A)*** | ***∆H˚(B)*** | ***∆G˚(A)*** | ***∆G˚(B)*** | ***T∆S˚(A)*** | ***T∆S˚(B)*** |
| --- | --- | --- | --- | --- | --- | --- | --- | --- | --- | --- |
| **R(bu-d)** | 0.00 | 0.00 | 0.00 | 0.00 | 0.00 | 0.00 | 0.00 | 0.00 | 0.00 | 0.00 |
| **CR1(bu-d)** | -4.00 | -1.63 | -3.84 | -1.00 | -4.43 | -1.59 | 3.97 | 4.31 | -8.40 | -5.90 |
| **CR2(bu-d)** | -3.95 | -2.64 | -3.75 | -2.22 | -4.34 | -2.82 | 3.53 | 3.40 | -7.87 | -6.22 |
| **CR3(bu-d)** | -3.97 | -2.36 | -3.75 | -1.96 | -4.35 | -2.55 | 3.64 | 3.98 | -7.99 | -6.53 |
| **CR4(bu-d)** | -4.69 | -2.50 | -4.34 | -2.12 | -4.93 | -2.71 | 2.80 | 3.80 | -7.73 | -6.51 |
| **CR5(bu-d)** | -3.74 | -2.55 | -3.55 | -1.91 | -4.14 | -2.50 | 4.12 | 3.98 | -8.26 | -6.48 |
| **TS1(bu-d)** | 13.42 | 15.86 | 13.09 | 15.69 | 12.50 | 15.10 | 21.90 | 23.82 | -9.40 | -8.72 |
| **TS2(bu-d)** | 10.02 | 10.57 | 10.01 | 10.62 | 9.42 | 10.03 | 17.81 | 18.12 | -8.39 | -8.10 |
| **TS3(bu-d)** | 12.66 | 13.26 | 12.53 | 13.17 | 11.94 | 12.58 | 20.79 | 21.51 | -8.85 | -8.94 |
| **TS4(bu-d)** | 11.14 | 12.28 | 10.94 | 12.14 | 10.35 | 11.55 | 19.60 | 20.66 | -9.25 | -9.11 |
| **TS5(bu-d)** | 11.36 | 12.76 | 10.81 | 12.30 | 10.22 | 11.71 | 20.38 | 21.72 | -10.16 | -10.02 |
| **CP1(bu-d)** | 7.84 | 9.13 | 8.35 | 9.96 | 7.75 | 9.37 | 15.31 | 15.25 | -7.55 | -5.88 |
| **CP2(bu-d)** | -3.23 | -4.13 | -2.45 | -3.21 | -3.05 | -3.80 | 3.03 | 1.68 | -6.08 | -5.48 |
| **CP3(bu-d)** | 3.56 | 2.13 | 4.35 | 3.33 | 3.76 | 2.74 | 10.39 | 7.26 | -6.63 | -4.52 |
| **CP4(bu-d)** | 1.86 | 2.31 | 2.89 | 3.40 | 2.30 | 2.81 | 8.34 | 8.55 | -6.03 | -5.73 |
| **CP5(bu-d)** | 2.47 | 3.09 | 2.60 | 3.92 | 2.00 | 3.33 | 10.75 | 10.07 | -8.74 | -6.75 |
| **P1(bu-d)(CH_3_CH_2_CH_2_CH_2_O+NH_2_)** | 11.58 | 11.65 | 11.73 | 11.71 | 11.73 | 11.71 | 10.53 | 10.87 | 1.19 | 0.84 |
| **P2(bu-d)(CH_3_CH_2_CH_2_CHOH+NH_2_)** | 3.03 | 1.25 | 3.42 | 1.56 | 3.42 | 1.56 | 1.71 | 0.27 | 1.71 | 1.29 |
| **P3(bu-d)(CH_3_CH_2_CHCH_2_OH+NH_2_)** | 8.50 | 6.53 | 9.18 | 7.13 | 9.18 | 7.13 | 6.71 | 4.78 | 2.47 | 2.35 |
| **P4(bu-d)(CH_3_CHCH_2_CH_2_OH+NH_2_)** | 6.84 | 4.59 | 7.45 | 5.13 | 7.45 | 5.13 | 5.00 | 3.27 | 2.46 | 1.87 |
| **P5(bu-d)(CH_2_CH_2_CH_2_CH_2_OH+NH_2_)** | 9.40 | 7.55 | 9.68 | 7.98 | 9.68 | 7.98 | 8.48 | 6.46 | 1.21 | 1.52 |

A and B refer to the M06-2X and CBS-QB3 methods, respectively.

**Table S56**. Absolute energies and thermodynamic parameters for stationary points of the n-butanol-d + NH reaction calculated at the CBS-QB3 level.

| **Species** | ***E(0K)( Hartree)*** | ***E˚(Hartree)*** | | ***H˚(Hartree)*** | ***G˚(Hartree)*** |
| --- | --- | --- | --- | --- | --- |
| **butanol-d** | -233.2200 | -233.2132 | -233.2122 | | -233.2503 |
| **CR1(bu-d)** | -288.3671 | -288.3569 | -288.3559 | | -288.4052 |
| **CR2(bu-d)** | -288.3687 | -288.3588 | -288.3579 | | -288.4067 |
| **CR3(bu-d)** | -288.3682 | -288.3584 | -288.3575 | | -288.4058 |
| **CR4(bu-d)** | -288.3685 | -288.3587 | -288.3577 | | -288.4060 |
| **CR5(bu-d)** | -288.3685 | -288.3583 | -288.3574 | | -288.4057 |
| **TS1(bu-d)** | -288.3392 | -288.3303 | -288.3293 | | -288.3741 |
| **TS2(bu-d)** | -288.3476 | -288.3384 | -288.3374 | | -288.3832 |
| **TS3(bu-d)** | -288.3433 | -288.3343 | -288.3334 | | -288.3778 |
| **TS4(bu-d)** | -288.3449 | -288.3359 | -288.3350 | | -288.3792 |
| **TS5(bu-d)** | -288.3441 | -288.3357 | -288.3347 | | -288.3775 |
| **CP1(bu-d)** | -288.3499 | -288.3394 | -288.3385 | | -288.3878 |
| **CP2(bu-d)** | -288.3710 | -288.3604 | -288.3594 | | -288.4094 |
| **CP3(bu-d)** | -288.3611 | -288.3500 | -288.3490 | | -288.4005 |
| **CP4(bu-d)** | -288.3608 | -288.3499 | -288.3489 | | -288.3985 |
| **CP5(bu-d)** | -288.3595 | -288.3490 | -288.3481 | | -288.3960 |
| **CH_3_CH_2_CH_2_CH_2_O-d** | -232.5547 | -232.5483 | -232.5473 | | -232.5852 |
| **CH_3_CH_2_CH_2_CHOH-d** | -232.5713 | -232.5644 | -232.5635 | | -232.6021 |
| **CH_3_CH_2_CHCH_2_OH-d** | -232.5629 | -232.5556 | -232.5546 | | -232.5950 |
| **CH_3_CHCH_2_CH_2_OH-d** | -232.5660 | -232.5587 | -232.5578 | | -232.5974 |
| **CH_2_CH_2_CH_2_CH_2_OH-d** | -232.5613 | -232.5542 | -232.5533 | | -232.5923 |

**Table S57**. Relative energies and thermodynamic parameters for stationary points of the n-butanol-d + NH reaction calculated at the M06-2X method.

| **Species** | ***(E+ZPE)(Hartree)*** | ***E˚(Hartree)*** | ***H˚(Hartree)*** | ***G˚(Hartree)*** | ***S˚(cal/K mol)*** |
| --- | --- | --- | --- | --- | --- |
| **butanol-d** | -233.4239 | -233.4173 | -233.4164 | -233.4538 | 78.687 |
| **CR1(bu-d)** | -288.6205 | -288.6113 | -288.6104 | -288.6549 | 93.775 |
| **CR2(bu-d)** | -288.6205 | -288.6112 | -288.6102 | -288.6556 | 95.544 |
| **CR3(bu-d)** | -288.6205 | -288.6112 | -288.6102 | -288.6555 | 95.161 |
| **CR4(bu-d)** | -288.6217 | -288.6121 | -288.6112 | -288.6568 | 96.016 |
| **CR5(bu-d)** | -288.6201 | -288.6109 | -288.6099 | -288.6547 | 94.249 |
| **TS1(bu-d)** | -288.5928 | -288.5843 | -288.5834 | -288.6264 | 90.416 |
| **TS2(bu-d)** | -288.5982 | -288.5893 | -288.5883 | -288.6329 | 93.799 |
| **TS3(bu-d)** | -288.5940 | -288.5852 | -288.5843 | -288.6281 | 92.266 |
| **TS4(bu-d)** | -288.5964 | -288.5878 | -288.5868 | -288.6300 | 90.921 |
| **TS5(bu-d)** | -288.5961 | -288.5880 | -288.5870 | -288.6288 | 87.868 |
| **CP1(bu-d)** | -288.6017 | -288.5919 | -288.5910 | -288.6369 | 96.616 |
| **CP2(bu-d)** | -288.6193 | -288.6091 | -288.6082 | -288.6564 | 101.562 |
| **CP3(bu-d)** | -288.6085 | -288.5983 | -288.5973 | -288.6447 | 99.723 |
| **CP4(bu-d)** | -288.6112 | -288.6006 | -288.5997 | -288.6480 | 101.708 |
| **CP5(bu-d)** | -288.6102 | -288.6011 | -288.6001 | -288.6441 | 92.621 |
| **CH_3_CH_2_CH_2_CH_2_O-d** | -232.7664 | -232.7601 | -232.7591 | -232.7969 | 79.440 |
| **CH_3_CH_2_CH_2_CHOH-d** | -232.7800 | -232.7733 | -232.7724 | -232.8109 | 81.169 |
| **CH_3_CH_2_CHCH_2_OH-d** | -232.7713 | -232.7641 | -232.7632 | -232.8030 | 83.734 |
| **CH_3_CHCH_2_CH_2_OH-d** | -232.7740 | -232.7669 | -232.7659 | -232.8057 | 83.680 |
| **CH_2_CH_2_CH_2_CH_2_OH-d** | -232.7699 | -232.7633 | -232.7624 | -232.8001 | 79.493 |

**Table S58**. The CCSD(T) absolute energies (in Hartree) and T1 diagnostic values for stationary points of the n-butanol-d + NH reaction calculated at the CCSD(T)/6-31+g(d**´**)//M06-2X level of theory.

| **Species** | ***CCSD(T)*** | ***T1 diagnostic*** |
| --- | --- | --- |
| **butanol-d** | -232.9485 | 0.0104 |
| **CR1(bu-d)** | -288.0364 | 0.0110 |
| **CR2(bu-d)** | -288.0380 | 0.0105 |
| **CR3(bu-d)** | -288.0378 | 0.0106 |
| **CR4(bu-d)** | -288.0384 | 0.0106 |
| **CR5(bu-d)** | -288.0367 | 0.0108 |
| **TS1(bu-d)** | -287.9990 | 0.0212 |
| **TS2(bu-d)** | -288.0008 | 0.0153 |
| **TS3(bu-d)** | -287.9961 | 0.0143 |
| **TS4(bu-d)** | -287.9984 | 0.0140 |
| **TS5(bu-d)** | -287.9976 | 0.0140 |
| **CP1(bu-d)** | -288.0186 | 0.0154 |
| **CP2(bu-d)** | -288.0320 | 0.0138 |
| **CP3(bu-d)** | -288.0156 | 0.0124 |
| **CP4(bu-d)** | -288.0192 | 0.0121 |
| **CP5(bu-d)** | -288.0189 | 0.0120 |
| **CH_3_CH_2_CH_2_CH_2_O-d** | -232.2967 | 0.0155 |
| **CH_3_CH_2_CH_2_CHOH-d** | -232.3019 | 0.0138 |
| **CH_3_CH_2_CHCH_2_OH-d** | -232.2919 | 0.0124 |
| **CH_3_CHCH_2_CH_2_OH-d** | -232.2951 | 0.0122 |
| **CH_2_CH_2_CH_2_CH_2_OH-d** | -232.2910 | 0.0119 |

**N-Butanol-e plus NH reaction**

n-butanol-e CH_3_CH_2_CH_2_CH_2_O-e CH_3_CH_2_CH_2_CHOH-e

CH_3_CH_2_CHCH_2_OH-e CH_3_CHCH_2_CH_2_OH-e CH_2_CHCH_2_CH_2_OH-e

CR1(bu-e) CR2(bu-e) CR3(bu-e)

CR4(bu-e) CR5(bu-e) TS1(bu-e)

TS2(bu-e) TS3(bu-e) TS4(bu-e)

TS5(bu-e) CP1(bu-e) CP2 (bu-e)

CP3(bu-e) CP4(bu-e) CP5(bu-e)

**Figure S17**. Structures of all stationary points including bond lengths (in angstrom) in the n-butanol-e + NH reaction calculated at the M06-2X/6-31+G(d,p) level of theory.

***R(bu-e)***

***CR1(bu-e)***

***CR2(bu-e)***

**Figure S18**. Potential energy surface of the n-butanol-e + NH reaction at the triplet ground state computed by the CBS-QB3 level.

**Table S59**. Relative energies and thermodynamic parameters for stationary points of the n-butanol-e + NH reaction. (Unit of all numbers is kcal mol^-1^)

| **Species** | ***∆(E+ZPE)(A)*** | ***∆E(0K)(B)*** | ***∆E˚(A)*** | ***∆E˚(B)*** | ***∆H˚(A)*** | ***∆H˚(B)*** | ***∆G˚(A)*** | ***∆G˚(B)*** | ***T∆S˚(A)*** | ***T∆S˚(B)*** |
| --- | --- | --- | --- | --- | --- | --- | --- | --- | --- | --- |
| **R(bu-e)** | 0.00 | 0.00 | 0.00 | 0.00 | 0.00 | 0.00 | 0.00 | 0.00 | 0.00 | 0.00 |
| **CR1(bu-e)** | -3.37 | -2.51 | -3.08 | -1.75 | -3.68 | -2.34 | 4.27 | 3.28 | -7.94 | -5.63 |
| **CR2(bu-e)** | -2.90 | -1.39 | -2.57 | -0.66 | -3.16 | -1.25 | 4.06 | 4.40 | -7.22 | -5.65 |
| **CR3(bu-e)** | -3.04 | -2.50 | -2.90 | -1.75 | -3.49 | -2.34 | 5.02 | 3.29 | -8.51 | -5.63 |
| **CR4(bu-e)** | -4.44 | -3.45 | -4.26 | -2.70 | -4.85 | -3.30 | 3.54 | 2.77 | -8.39 | -6.07 |
| **CR5(bu-e)** | -5.70 | -3.76 | -5.37 | -3.26 | -5.96 | -3.85 | 1.91 | 2.39 | -7.87 | -6.24 |
| **TS1(bu-e)** | 13.75 | 15.56 | 13.53 | 15.49 | 12.94 | 14.90 | 21.96 | 22.95 | -9.02 | -8.05 |
| **TS2(bu-e)** | 9.49 | 10.34 | 9.66 | 10.43 | 9.07 | 9.84 | 16.70 | 17.81 | -7.64 | -7.97 |
| **TS3(bu-e)** | 11.19 | 12.16 | 11.07 | 12.10 | 10.47 | 11.50 | 19.51 | 20.27 | -9.03 | -8.76 |
| **TS4(bu-e)** | 9.75 | 10.24 | 9.57 | 10.12 | 8.98 | 9.53 | 18.06 | 18.27 | -9.08 | -8.74 |
| **TS5(bu-e)** | 12.23 | 13.84 | 11.75 | 13.48 | 11.15 | 12.89 | 21.25 | 22.43 | -10.09 | -9.54 |
| **CP1(bu-e)** | 7.37 | 8.22 | 8.18 | 8.97 | 7.59 | 8.38 | 13.94 | 14.75 | -6.34 | -6.37 |
| **CP2(bu-e)** | -3.96 | -4.08 | -3.20 | -3.17 | -3.80 | -3.76 | 2.96 | 2.09 | -6.76 | -5.85 |
| **CP3(bu-e)** | 0.27 | 0.40 | 1.11 | 1.60 | 0.51 | 1.01 | 7.11 | 5.54 | -6.60 | -4.53 |
| **CP4(bu-e)** | -0.41 | -0.41 | 0.36 | 0.88 | -0.23 | 0.29 | 6.81 | 4.86 | -7.05 | -4.57 |
| **CP5(bu-e)** | 4.30 | 4.38 | 4.73 | 5.45 | 4.14 | 4.86 | 11.99 | 10.56 | -7.85 | -5.71 |
| **P1(bu-e)(CH_3_CH_2_CH_2_CH_2_O+NH_2_)** | 10.85 | 10.81 | 10.90 | 10.92 | 10.90 | 10.92 | 10.19 | 9.91 | 0.72 | 1.01 |
| **P2(bu-e)(CH_3_CH_2_CH_2_CHOH+NH_2_)** | 3.02 | 0.95 | 3.33 | 1.31 | 3.33 | 1.31 | 2.16 | -0.11 | 1.17 | 1.42 |
| **P3(bu-e)(CH_3_CH_2_CHCH_2_OH+NH_2_)** | 6.37 | 4.42 | 6.92 | 4.96 | 6.92 | 4.96 | 4.82 | 2.77 | 2.10 | 2.19 |
| **P4(bu-e)(CH_3_CHCH_2_CH_2_OH+NH_2_)** | 5.18 | 3.31 | 5.76 | 3.96 | 5.76 | 3.96 | 3.71 | 1.49 | 2.04 | 2.48 |
| **P5(bu-e)(CH_2_CH_2_CH_2_CH_2_OH+NH_2_)** | 8.86 | 6.76 | 9.29 | 7.28 | 9.29 | 7.28 | 7.81 | 5.41 | 1.48 | 1.87 |

A and B refer to the M06-2X and CBS-QB3 methods, respectively.

**Table S60**. Absolute energies and thermodynamic parameters for stationary points of the n-butanol-e + NH reaction calculated at the CBS-QB3 level.

| **Species** | ***E(0K)( Hartree)*** | ***E˚(Hartree)*** | | ***H˚(Hartree)*** | ***G˚(Hartree)*** |
| --- | --- | --- | --- | --- | --- |
| **butanol-e** | -233.2186 | -233.2119 | -233.2109 | | -233.2484 |
| **CR1(bu-e)** | -288.3670 | -288.3568 | -288.3559 | | -288.4049 |
| **CR2(bu-e)** | -288.3653 | -288.3551 | -288.3541 | | -288.4032 |
| **CR3(bu-e)** | -288.3670 | -288.3568 | -288.3559 | | -288.4049 |
| **CR4(bu-e)** | -288.3685 | -288.3583 | -288.3574 | | -288.4057 |
| **CR5(bu-e)** | -288.3690 | -288.3592 | -288.3583 | | -288.4064 |
| **TS1(bu-e)** | -288.3382 | -288.3293 | -288.3284 | | -288.3736 |
| **TS2(bu-e)** | -288.3466 | -288.3374 | -288.3364 | | -288.3818 |
| **TS3(bu-e)** | -288.3437 | -288.3347 | -288.3338 | | -288.3779 |
| **TS4(bu-e)** | -288.3467 | -288.3379 | -288.3369 | | -288.3810 |
| **TS5(bu-e)** | -288.3410 | -288.3325 | -288.3316 | | -288.3744 |
| **CP1(bu-e)** | -288.3499 | -288.3397 | -288.3388 | | -288.3866 |
| **CP2(bu-e)** | -288.3695 | -288.3591 | -288.3581 | | -288.4068 |
| **CP3(bu-e)** | -288.3624 | -288.3515 | -288.3505 | | -288.4013 |
| **CP4(bu-e)** | -288.3637 | -288.3526 | -288.3517 | | -288.4024 |
| **CP5(bu-e)** | -288.3560 | -288.3453 | -288.3444 | | -288.3933 |
| **CH_3_CH_2_CH_2_CH_2_O+NH_2_-e** | -232.5546 | -232.5483 | -232.5473 | | -232.5848 |
| **CH_3_CH_2_CH_2_CHOH+NH_2_-e** | -232.5703 | -232.5636 | -232.5626 | | -232.6008 |
| **H_3_CH_2_CHCH_2_OH+NH_2_-e** | -232.5648 | -232.5578 | -232.5568 | | -232.5962 |
| **CH_3_CHCH_2_CH_2_OH+NH_2_-e** | -232.5666 | -232.5593 | -232.5584 | | -232.5983 |
| **CH_2_CH_2_CH_2_CH_2_OH+NH_2_-e** | -232.5611 | -232.5541 | -232.5531 | | -232.5920 |

**Table S61**. Relative energies and thermodynamic parameters for stationary points of the n-butanol-e + NH reaction calculated at the M06-2X method.

| **Species** | ***(E+ZPE)(Hartree)*** | ***E˚(Hartree)*** | ***H˚(Hartree)*** | ***G˚(Hartree)*** | ***S˚(cal/K mol)*** |
| --- | --- | --- | --- | --- | --- |
| **butanol-e** | -233.4228 | -233.4162 | -233.4153 | -233.4529 | 79.156 |
| **CR1(bu-e)** | -288.6185 | -288.6090 | -288.6081 | -288.6536 | 95.768 |
| **CR2(bu-e)** | -288.6177 | -288.6082 | -288.6072 | -288.6539 | 98.197 |
| **CR3(bu-e)** | -288.6179 | -288.6087 | -288.6078 | -288.6524 | 93.885 |
| **CR4(bu-e)** | -288.6202 | -288.6109 | -288.6099 | -288.6547 | 94.289 |
| **CR5(bu-e)** | -288.6222 | -288.6127 | -288.6117 | -288.6573 | 96.014 |
| **TS1(bu-e)** | -288.5912 | -288.5825 | -288.5816 | -288.6254 | 92.158 |
| **TS2(bu-e)** | -288.5980 | -288.5887 | -288.5878 | -288.6338 | 96.805 |
| **TS3(bu-e)** | -288.5952 | -288.5865 | -288.5855 | -288.6293 | 92.123 |
| **TS4(bu-e)** | -288.5975 | -288.5888 | -288.5879 | -288.6316 | 91.958 |
| **TS5(bu-e)** | -288.5936 | -288.5854 | -288.5844 | -288.6265 | 88.556 |
| **CP1(bu-e)** | -288.6013 | -288.5911 | -288.5901 | -288.6382 | 101.139 |
| **CP2(bu-e)** | -288.6194 | -288.6092 | -288.6083 | -288.6556 | 99.742 |
| **CP3(bu-e)** | -288.6126 | -288.6023 | -288.6014 | -288.6490 | 100.269 |
| **CP4(bu-e)** | -288.6137 | -288.6035 | -288.6026 | -288.6495 | 98.783 |
| **CP5(bu-e)** | -288.6062 | -288.5965 | -288.5956 | -288.6413 | 96.086 |
| **CH_3_CH_2_CH_2_CH_2_O-e** | -232.7665 | -232.7603 | -232.7593 | -232.7965 | 78.312 |
| **CH_3_CH_2_CH_2_CHOH-e** | -232.7790 | -232.7723 | -232.7714 | -232.8093 | 79.828 |
| **CH_3_CH_2_CHCH_2_OH-e** | -232.7736 | -232.7666 | -232.7657 | -232.8051 | 82.968 |
| **CH_3_CHCH_2_CH_2_OH-e** | -232.7755 | -232.7685 | -232.7675 | -232.8068 | 82.769 |
| **CH_2_CH_2_CH_2_CH_2_OH-e** | -232.7697 | -232.7628 | -232.7619 | -232.8003 | 80.876 |

**Table S62**. The CCSD(T) absolute energies (in Hartree) and T1 diagnostic values for stationary points of the n-butanol-e + NH reaction calculated at the CCSD(T)/6-31+g(d**´**)//M06-2X level of theory.

| **Species** | ***CCSD(T)*** | ***T1 diagnostic*** |
| --- | --- | --- |
| **butanol-e** | -232.9476 | 0.0103 |
| **CR1(bu-e)** | -288.0348 | 0.0109 |
| **CR2(bu-e)** | -288.0347 | 0.0110 |
| **CR3(bu-e)** | -288.0351 | 0.0107 |
| **CR4(bu-e)** | -288.0367 | 0.0108 |
| **CR5(bu-e)** | -288.0384 | 0.0106 |
| **TS1(bu-e)** | -287.9986 | 0.0206 |
| **TS2(bu-e)** | -288.0000 | 0.0156 |
| **TS3(bu-e)** | -287.9965 | 0.0142 |
| **TS4(bu-e)** | -287.9997 | 0.0144 |
| **TS5(bu-e)** | -287.9944 | 0.0140 |
| **CP1(bu-e)** | -288.0184 | 0.0156 |
| **CP2(bu-e)** | -288.0296 | 0.0140 |
| **CP3(bu-e)** | -288.0213 | 0.0127 |
| **CP4(bu-e)** | -288.0232 | 0.0124 |
| **CP5(bu-e)** | -288.0141 | 0.0117 |
| **CH_3_CH_2_CH_2_CH_2_O-e** | -232.2967 | 0.0156 |
| **CH_3_CH_2_CH_2_CHOH-e** | -232.3006 | 0.0140 |
| **CH_3_CH_2_CHCH_2_OH-e** | -232.2953 | 0.0128 |
| **CH_3_CHCH_2_CH_2_OH-e** | -232.2962 | 0.0125 |
| **CH_2_CH_2_CH_2_CH_2_OH-e** | -232.2910 | 0.0117 |

**Table S63.** Cartesian coordinates (Å) of all optimized species in the CH_3_OH + NH reaction.

| Species | Method | Cartesian Coordindates |
| --- | --- | --- |
| CH_3_OH | CBS-QB3 | C -0.66334400 -0.01949800 0.00000100  H -1.03511700 -0.54300700 -0.89159600  H -1.03520800 -0.54198200 0.89216600  H -1.08396100 0.98718500 -0.00060300  H 1.13432300 -0.75918400 0.00001500  O 0.75000300 0.12174700 0.00000100 |
|  | M06-2X | C -0.66290900 0.02093000 0.00000200  H -1.02364500 0.54663600 0.89280900  H -1.02382900 0.54493000 -0.89374800  H -1.08108000 -0.98674000 0.00097500  H 1.15223600 0.74863200 -0.00006400  O 0.74422200 -0.12238000 0.00000200 |
| NH | CBS-QB3 | N 0.00000000 0.00000000 0.13049300  H 0.00000000 0.00000000 -0.91345200 |
|  | M06-2X | N 0.00000000 0.00000000 0.13007300  H 0.00000000 0.00000000 -0.91051200 |
| CR1(m) | CBS-QB3 | C 1.39278900 -0.40271100 0.00537800  H 0.88851900 -1.37158500 0.11388100  H 1.97809000 -0.41544400 -0.92337300  H 2.08343000 -0.28143900 0.84121200  H -0.15898800 0.57213000 -0.65417500  O 0.48407300 0.68994100 0.05426400  N -2.12000600 -0.21869500 -0.10913000  H -2.18032700 -0.07606000 0.91998400 |
|  | M06-2X | C 1.15481300 -0.52931900 -0.01061900  H 0.38183000 -1.29207100 -0.17468700  H 1.89782000 -0.59811900 -0.81379400  H 1.65187000 -0.73044200 0.93963300  H 0.14185800 0.97361100 -0.73854700  O 0.59901300 0.77024300 0.08525000  N -1.99741700 -0.19043500 -0.10449400  H -1.81244700 -0.00596900 0.90056500 |
| CR2(m) | CBS-QB3 | C -1.51477000 -0.42565300 -0.02254100  H -1.63416300 -0.76957000 -1.05708600  H -1.18746700 -1.26914100 0.58551500  H -2.48536600 -0.08409500 0.35591900  H -0.77353200 1.33595700 -0.43927300  O -0.51328700 0.58289700 0.09924600  N 2.53592200 -0.19763800 -0.01786200  H 1.52399000 0.06105400 0.02123400 |
|  | M06-2X | C 1.12434900 0.55914400 -0.00734200  H 0.37641900 1.24688000 -0.42220700  H 1.36870700 0.87557500 1.00790600  H 2.03544300 0.60671800 -0.61443200  H 0.42467200 -1.08955300 -0.79673100  O 0.62396500 -0.76495100 0.08789800  N -2.04474100 0.19276100 -0.08617700  H -1.62986500 -0.22419900 0.76957500 |
| TS1(m) | CBS-QB3 | C 1.27246300 -0.37859000 -0.02331600  H 1.23389900 -1.03420300 0.85851100  H 1.13944900 -0.97558100 -0.93585300  H 2.28294400 0.05442700 -0.05917000  H -0.80229800 0.34598100 -0.23143100  O 0.39429200 0.70428100 0.05987900  N -1.83013600 -0.17051000 -0.11598900  H -1.83215500 -0.55976300 0.84073300 |
|  | M06-2X | C 1.29662900 -0.37327700 -0.01615100  H 1.19768600 -1.01850600 0.86549000  H 1.20336300 -0.95897500 -0.93891400  H 2.30463300 0.07053700 0.00127200  H -0.79945400 0.16197400 0.07097500  O 0.42608300 0.71254700 0.01759600  N -1.81167600 -0.31458100 0.02607600  H -2.41293800 0.48632500 -0.22522200 |
| TS2(m) | CBS-QB3 | C 0.52594300 0.62291800 0.00949400  H -0.77445800 0.25378800 -0.11702100  H 0.57115900 1.18395300 0.94110400  H 0.67612600 1.24905000 -0.87625300  H 1.41713800 -0.89519500 -0.77284400  O 1.35325500 -0.48690500 0.09637300  N -1.98102400 -0.15287700 -0.11948500  H -2.00449800 -0.56372400 0.83346100 |
|  | M06-2X | C 0.55598700 0.65174200 0.00439600  H -0.76774500 0.34429500 -0.08408900  H 0.67295100 1.22701200 0.92162400  H 0.73010600 1.23288500 -0.90640100  H 1.27841800 -0.97553100 -0.75465000  O 1.27303600 -0.52588000 0.09733200  N -1.92500900 -0.14326200 -0.11788900  H -1.95887600 -0.52923500 0.84370800 |
| CP1(m) | CBS-QB3 | C 1.23626800 -0.52537600 -0.01411200  H 1.43663300 -0.77933000 1.04769900  H 0.39941200 -1.16956800 -0.31874900  H 2.15971800 -0.73993400 -0.57059700  H -1.33107300 0.52852800 0.05686300  O 0.90343500 0.80246700 -0.00987400  N -2.05359600 -0.20489300 -0.00730700  H -2.93461000 0.32707600 -0.00039900 |
|  | M06-2X | C 1.19247600 -0.53744500 -0.00831600  H 1.17384300 -0.84242800 1.05461200  H 0.39372800 -1.11895100 -0.49015700  H 2.18404000 -0.75918500 -0.42030100  H -1.33879500 0.61813800 0.04969500  O 0.89626700 0.80460600 -0.01044900  N -1.97434300 -0.18879300 -0.01547400  H -2.91741400 0.21179900 0.04795600 |
| CP2(m) | CBS-QB3 | C 1.51606400 0.48296100 0.05244600  H -2.51630800 0.36971300 0.82546800  H 2.58335800 0.35185000 -0.06803300  H 1.04027100 1.41113300 -0.25248600  H -0.15139200 -0.44933400 -0.00401000  O 0.79904800 -0.67013400 -0.00410100  N -1.97232600 0.09302800 -0.00027800  H -2.63841100 0.12875200 -0.78086600 |
|  | M06-2X | C 1.41112500 0.53317200 0.03764400  H -2.36307300 0.54440400 0.79424500  H 2.48695700 0.53644500 -0.07603100  H 0.81957700 1.37589100 -0.31098800  H -0.12130200 -0.59432600 0.00224200  O 0.84763900 -0.69837600 0.01171400  N -1.91925100 0.07165500 0.00034700  H -2.63525800 0.02397700 -0.73146600 |
| CH_3_O | CBS-QB3 | C 0.57370700 0.00151500 -0.01382200  H 0.87117900 -0.05220500 1.05334400  H 1.00955700 -0.88848600 -0.49445800  H 1.01376400 0.92762400 -0.41414700  O -0.79209300 0.00049700 -0.00772600 |
|  | M06-2X | C 0.57912500 -0.00009700 -0.01177000  H 0.87056000 0.00414900 1.05460000  H 1.00071200 -0.91013100 -0.45882400  H 1.00044700 0.90686300 -0.46531200  O -0.79330800 -0.00003700 -0.00748100 |
| CH_2_OH | CBS-QB3 | C -0.68451200 0.02764500 -0.06626500  H -1.23067200 -0.88948800 0.10124600  H -1.12330100 0.98967400 0.17695500  H 1.09171300 0.73509000 -0.06874600  O 0.67116600 -0.12514300 0.02351700 |
|  | M06-2X | C -0.68341100 0.02939500 -0.07113800  H -1.23010300 -0.88641400 0.10354500  H -1.11541700 0.98910700 0.19273100  H 1.10910800 0.72305700 -0.08245400  O 0.66711000 -0.12526500 0.02662600 |
| NH_2_ | CBS-QB3 | N 0.00000000 0.00000000 0.14395500  H 0.00000000 0.80198400 -0.50384400  H 0.00000000 -0.80198400 -0.50384400 |
|  | M06-2X | N 0.00000000 0.00000000 0.14073800  H 0.00000000 0.80930100 -0.49258300  H 0.00000000 -0.80930100 -0.49258300 |

**Table S64.** Cartesian coordinates (Å) of all optimized species in the C_2_H_5_OH + NH reaction.

| Species | Method | Cartesian Coordindates |
| --- | --- | --- |
| C_2_H_5_OH | CBS-QB3 | C -1.22127200 -0.22259000 0.00009500  C 0.08537700 0.55010400 0.00005300  H -2.07576500 0.45949800 0.00026300  H -1.28487100 -0.86006500 -0.88484600  H -1.28469000 -0.86026900 0.88490300  H 0.13653500 1.19798100 -0.88732200  H 0.13670500 1.19779000 0.88755700  O 1.15070900 -0.39812500 -0.00015000  H 1.98178400 0.08497400 -0.00024000 |
|  | M06-2X | C -1.21707500 -0.22271400 0.00009000  C 0.08473500 0.55129400 0.00005300  H -2.07117100 0.45988300 0.00025600  H -1.27556400 -0.85951400 -0.88623300  H -1.27538800 -0.85972000 0.88627700  H 0.14299100 1.19416900 -0.88922900  H 0.14315300 1.19398200 0.88946000  O 1.14272400 -0.39553300 -0.00014100  H 1.98822500 0.06398600 -0.00026400 |
| CR1(e) | CBS-QB3 | C 1.85184700 -0.56432300 -0.12931100  C 0.79583400 0.34359200 0.49041800  H 2.63571500 -0.80446000 0.59586200  H 1.40871200 -1.50693000 -0.46638600  H 2.31341700 -0.07831000 -0.99220400  H 0.33790100 -0.14790300 1.36022800  H 1.25400200 1.27019700 0.84458800  O -0.20165100 0.74607200 -0.44611200  H -0.68536300 -0.04277700 -0.72116400  N -2.64366000 -0.52366500 0.04158200  H -3.03164100 0.33164800 0.49025000 |
|  | M06-2X | C 1.57082000 -0.68946500 -0.20622000  C 0.63636600 0.23675400 0.55423700  H 2.15472000 -1.30592800 0.48355000  H 0.99817500 -1.36267800 -0.85398500  H 2.25893600 -0.11007800 -0.82768300  H -0.05278300 -0.35627000 1.17584500  H 1.19961700 0.89627800 1.21992800  O -0.09007500 1.09727500 -0.30916300  H -0.49622200 0.55676400 -0.99862800  N -2.28609300 -0.63690500 -0.06333900  H -2.58230300 0.07832100 0.62955000 |
| CR2(e) | CBS-QB3 | C -1.18326800 -1.07713000 -0.14076100  C -1.14776200 0.36300300 0.33414700  H -2.02238800 -1.60899700 0.31511700  H -1.29592300 -1.11732300 -1.22649700  H -0.26067900 -1.59670900 0.12854200  H -2.08772300 0.86854200 0.07736800  H -1.03092300 0.40130300 1.42546100  O -0.04606000 1.01619600 -0.31181800  H -0.03727500 1.93907600 -0.04002000  N 2.74698500 -0.40471200 0.10967400  H 1.86067500 0.10228800 -0.11345700 |
|  | M06-2X | C -0.98640400 -1.08701100 -0.23372500  C -0.82102400 0.22112200 0.51208500  H -1.46216700 -1.83250200 0.40901600  H -1.60476000 -0.94004000 -1.12293300  H -0.01345200 -1.47793300 -0.54578600  H -1.79740900 0.59270400 0.84824200  H -0.18369300 0.07681500 1.39597400  O -0.21930100 1.16020500 -0.37858300  H -0.22474300 2.03407000 0.02596000  N 2.30007900 -0.40130600 0.11906800  H 1.78465200 0.26971700 -0.48544800 |
| CR3(e) | CBS-QB3 | C -1.08693700 1.13362700 -0.00025800  C -1.31074300 -0.36662800 0.00058100  H -2.04350800 1.66244400 0.00064800  H -0.52333600 1.43811300 0.88464900  H -0.52537300 1.43743300 -0.88669100  H -1.88381200 -0.66241700 0.88939500  H -1.88574900 -0.66312300 -0.88674500  O -0.02955100 -1.00810500 -0.00058200  H -0.16002400 -1.96086100 -0.00008000  N 2.82229100 0.33478500 0.00023700  H 1.88824500 -0.13223900 -0.00011900 |
|  | M06-2X | C -0.51328400 1.22716500 0.00000700  C -1.28438200 -0.07711600 0.00007300  H -1.20281000 2.07559200 -0.00058200  H 0.12417600 1.29706800 0.88621400  H 0.12449700 1.29639800 -0.88617800  H -1.92455800 -0.14068100 0.88967900  H -1.92457100 -0.14047000 -0.88952100  O -0.33909500 -1.14408000 0.00003600  H -0.79967400 -1.98939700 -0.00028300  N 2.48161300 0.07158900 0.00009200  H 1.73040900 -0.64728000 -0.00073900 |
| TS1(e) | CBS-QB3 | C -1.46214100 0.78016800 -0.12307900  C -0.81164400 -0.51217000 0.36508000  H -2.37268500 0.99844300 0.44275300  H -0.78073100 1.62735600 -0.00069700  H -1.71868600 0.69898100 -1.18162500  H -0.55279500 -0.44836400 1.43287000  H -1.53041400 -1.34354000 0.27837700  O 0.28367200 -0.92165000 -0.40235600  H 1.36524600 -0.35198500 -0.02608700  N 2.16426300 0.46644900 0.16078100  H 1.81355300 1.31918300 -0.30421400 |
|  | M06-2X | C -1.30730500 0.85778600 -0.14448300  C -0.83519500 -0.49577600 0.36733600  H -2.19216600 1.19076600 0.40546700  H -0.52292800 1.61108000 -0.01930700  H -1.55422600 0.79464900 -1.20707900  H -0.60565600 -0.46615100 1.44411500  H -1.62966900 -1.25149600 0.24645400  O 0.23437400 -1.02593100 -0.35532100  H 1.24067800 -0.13161700 -0.40049600  N 2.02405300 0.56430300 0.00386500  H 2.07559900 0.33803000 1.00925000 |
| TS2(e) | CBS-QB3 | C -0.93927500 -1.18131800 -0.12027100  C -0.35897100 0.09341300 0.42797600  H -0.49645900 -2.04880800 0.37440700  H -2.02427900 -1.21335600 0.03033400  H -0.74889000 -1.25631300 -1.19395300  H -0.44727200 0.19893100 1.51657300  H 0.97259100 0.01582500 0.28244400  O -0.83870300 1.19752900 -0.27610100  H -0.55229000 2.00004300 0.17131400  N 2.23332800 -0.07535600 0.02585400  H 2.16239900 -0.22162700 -0.99952100 |
|  | M06-2X | C -1.01011300 -1.13176900 -0.13185000  C -0.36383000 0.09572200 0.44459300  H -0.65127100 -2.02803900 0.38034300  H -2.10016600 -1.08116700 -0.02791700  H -0.77689100 -1.21616200 -1.19725300  H -0.47915900 0.21369500 1.52918100  H 0.96579800 -0.07120900 0.32066700  O -0.72533400 1.22494700 -0.27832600  H -0.40342900 2.01818300 0.16387900  N 2.19958200 -0.16592200 0.02613100  H 2.09437200 -0.25714200 -1.00166500 |
| TS3(e) | CBS-QB3 | C -0.07661700 1.15141400 -0.24051200  C 1.02003300 0.31226500 0.34145700  H -0.25939500 2.09733300 0.26612200  H -0.06263000 1.23117500 -1.32625500  H -1.28231400 0.43489500 -0.00394600  H 1.99776400 0.78143100 0.14022900  H 0.90744500 0.24590900 1.43297500  O 0.96030300 -0.98484600 -0.25939400  H 1.69743800 -1.50483600 0.07397100  N -2.11683500 -0.42159000 0.14020000  H -1.52338100 -1.23808200 -0.09501800 |
|  | M06-2X | C -0.05067600 1.16580700 -0.24746200  C 1.00982500 0.29483900 0.35358600  H -0.24357100 2.10458700 0.26976500  H -0.01934400 1.24705900 -1.33390800  H -1.26799800 0.43292400 -0.01763800  H 2.00571700 0.73356900 0.17979600  H 0.86398700 0.21330500 1.43986200  O 0.92360200 -0.98533900 -0.26212200  H 1.65390700 -1.53680800 0.03725100  N -2.08687600 -0.40698800 0.14752900  H -1.52827100 -1.22688200 -0.14760600 |
| CP1(e) | CBS-QB3 | C -0.94559200 1.15136700 -0.13474200  C -1.24291500 -0.29830400 0.24349500  H -1.65731700 1.82639000 0.34905700  H 0.06722300 1.42576200 0.16585800  H -1.03397700 1.28418900 -1.21602900  H -1.18499700 -0.42602600 1.34739400  H -2.27810500 -0.59870500 0.00116900  O -0.36125200 -1.23088600 -0.23221200  H 1.72680700 -0.42900300 -0.16519600  N 2.43756300 0.25641300 0.13364400  H 3.31848200 -0.14879200 -0.21258900 |
|  | M06-2X | C -1.09002900 1.05518700 -0.20668600  C -0.90036200 -0.30730700 0.44663900  H -1.53810200 1.75854700 0.50090100  H -0.12287500 1.45236600 -0.52696600  H -1.74632600 0.97489800 -1.07717800  H -0.18613300 -0.21858300 1.29082400  H -1.83148900 -0.71081600 0.87534400  O -0.30680600 -1.24227200 -0.36678000  H 1.82814900 -0.45922800 -0.47030200  N 2.15063500 0.27628800 0.17353100  H 2.93912600 0.71970000 -0.31281500 |
| CP2(e) | CBS-QB3 | C -2.25715900 -0.13050600 0.02734600  C -0.79733800 -0.38151800 -0.10287500  H -2.82741800 -1.03827600 -0.18563300  H -2.53948700 0.21369700 1.03718700  H -2.57895700 0.64904000 -0.67185600  H -0.36719400 -1.31612600 0.25671300  H 3.26155900 -0.46542100 -0.80890000  O -0.01060100 0.73156600 -0.00950900  H 0.92495100 0.45775100 0.00491600  N 2.73362500 -0.15388000 0.01496000  H 3.40295900 -0.20389000 0.79209800 |
|  | M06-2X | C -2.19344200 -0.22401900 0.01734200  C -0.71411600 -0.33963000 -0.07810700  H -2.66729500 -1.19446700 -0.15025100  H -2.51824200 0.14837300 1.00188300  H -2.56932700 0.47990700 -0.73268200  H -0.19922200 -1.20501200 0.34093200  H 3.01180500 -0.69888100 -0.80313500  O -0.04936500 0.84710700 -0.01148500  H 0.90651700 0.66412700 0.01599700  N 2.64368500 -0.19070300 0.00792400  H 3.37023800 -0.25408400 0.72826400 |
| CP3(e) | CBS-QB3 | C 1.62309100 0.85989600 -0.25933600  C 0.87177500 -0.13941400 0.53133300  H 1.91144200 1.80423900 0.18291100  H 2.01364600 0.58346700 -1.23001900  H -3.05548300 0.88746700 -0.58293700  H 1.56067800 -0.70982500 1.18207000  H 0.14603800 0.35581700 1.19297400  O 0.20500500 -1.03989900 -0.36553600  H -0.14140900 -1.77039200 0.15697500  N -2.47602500 0.40656000 0.11905200  H -1.71197400 -0.00038900 -0.44303200 |
|  | M06-2X | C 0.90865200 1.19043000 -0.25312600  C 0.86154900 -0.07577400 0.51835700  H 0.87022800 2.14114700 0.26317700  H 1.21238600 1.16109900 -1.29242300  H -2.17507000 1.06121300 -0.22914700  H 1.84162100 -0.28490700 0.98230200  H 0.12290800 0.00221100 1.33124800  O 0.51509900 -1.12809900 -0.37584000  H 0.56459900 -1.97071700 0.08833100  N -2.22825000 0.09199900 0.11123500  H -1.58092000 -0.41717700 -0.50680000 |
| CH_3_CH_2_O | CBS-QB3 | C 1.18917700 -0.19466600 -0.00004200  C -0.18121700 0.47869100 -0.00115200  H 1.99275300 0.54788100 0.01203900  H 1.29697700 -0.83575400 0.87790900  H 1.30774900 -0.81566000 -0.89109400  H -0.30540200 1.14625700 0.87611500  H -0.29709000 1.17444100 -0.85580100  O -1.25534400 -0.36516500 -0.00150100 |
|  | M06-2X | C 1.18242600 -0.19790800 -0.00017800  C -0.17549700 0.48823800 -0.00191400  H 1.99071100 0.53907500 0.02308300  H 1.27634000 -0.84797600 0.87322800  H 1.29808300 -0.81030900 -0.89802700  H -0.30726700 1.12919000 0.89075200  H -0.29164200 1.18257900 -0.85297100  O -1.25097500 -0.36681700 -0.00293900 |
| CH_3_CHOH | CBS-QB3 | C 1.22776000 -0.16335700 0.01124700  C -0.09169600 0.50742100 -0.10092900  H 2.03381400 0.53510600 -0.22508200  H 1.41348100 -0.56099500 1.02256100  H 1.29120400 -1.00966200 -0.68059300  H -0.23093000 1.52700400 0.25351100  O -1.16817400 -0.34140700 0.02208200  H -1.97855900 0.17542200 -0.00895400 |
|  | M06-2X | C 1.22408300 -0.16509700 0.01118300  C -0.09260100 0.51149300 -0.10500800  H 2.03322700 0.53714900 -0.20238100  H 1.38797000 -0.57956900 1.01769200  H 1.28890300 -0.99696700 -0.69762300  H -0.23213700 1.52403900 0.26934800  O -1.16009000 -0.33937600 0.02565900  H -1.98613800 0.15197900 -0.02936100 |
| CH_2_CH_2_OH | CBS-QB3 | C 1.25190700 -0.25463900 -0.01964900  C -0.00850700 0.51943000 0.03721300  H 2.18986300 0.23786700 -0.24024700  H 1.25283500 -1.28696200 0.30426900  H -0.06025100 1.08641700 0.98809200  H -0.02609800 1.27195600 -0.76900700  O -1.11274800 -0.37712900 -0.06390100  H -1.91476400 0.11900500 0.12272000 |
|  | M06-2X | C 1.24890900 -0.25460800 -0.02503800  C -0.01157700 0.52323100 0.04008300  H 2.18747700 0.24271600 -0.23189700  H 1.24875600 -1.28478000 0.30718700  H -0.06856400 1.08417800 0.99138400  H -0.03933200 1.26820800 -0.77059700  O -1.10338900 -0.37777200 -0.05963500  H -1.92522100 0.10011500 0.09072700 |

**Table S65.** Cartesian coordinates (Å) of all optimized species in the n-C_3_H_7_OH+ NH reaction.

| Species | Method | Cartesian Coordindates |
| --- | --- | --- |
| C_3_H_7_OH | CBS-QB3 | C 1.90265700 0.12384400 0.01393500  C 0.51481900 -0.52070700 -0.04046000  H 2.69162000 -0.63073600 -0.02939200  H 2.05365800 0.80879100 -0.82594000  H 2.04196400 0.69568900 0.93667400  C -0.61983000 0.49990100 0.02920200  H 0.39869800 -1.10145200 -0.96109900  H 0.40546700 -1.22917400 0.79132900  H -0.54039900 1.08623700 0.95745500  H -0.54347700 1.20295900 -0.80470500  O -1.90885200 -0.09411200 -0.09123500  H -2.02259300 -0.69764700 0.64949500 |
|  | M06-2X | C -1.88911100 -0.12833100 0.02080300  C -0.51278800 0.53022800 -0.04777200  H -2.68851600 0.61492400 -0.03202600  H -2.02928000 -0.82922700 -0.80850100  H -2.01314400 -0.68720800 0.95427300  C 0.61140200 -0.49478800 0.02150700  H -0.40505500 1.10231400 -0.97585000  H -0.40078000 1.24356100 0.78020300  H 0.52461400 -1.08566000 0.94638200  H 0.53456500 -1.19156000 -0.81919000  O 1.89874200 0.08783200 -0.08385100  H 2.03063800 0.68755000 0.65829400 |
| CR1(pr) | CBS-QB3 | C -2.34945500 -0.71419600 0.13095500  C -1.00277700 -0.27405200 -0.45079200  H -2.82106000 -1.47195600 -0.49859800  H -2.22747700 -1.14327600 1.12990000  H -3.04367900 0.12769000 0.21315100  C -0.31381100 0.79025300 0.39918600  H -0.33090400 -1.13316300 -0.54527400  H -1.15100700 0.12101300 -1.46429400  H -0.95951000 1.67285700 0.50462200  H -0.11769700 0.40759300 1.40368900  N 3.08607400 -1.14517100 0.09537100  H 2.48023000 -0.29529500 0.04057300  O 0.96600100 1.17293200 -0.11609000  H 0.83683100 1.53524500 -0.99875300 |
|  | M06-2X | C 2.23850200 -0.62020600 -0.00696200  C 0.72221500 -0.44260800 0.03548600  H 2.51538700 -1.67572500 0.04892600  H 2.65578200 -0.21374800 -0.93411600  H 2.71968200 -0.10360600 0.83002300  C 0.32264800 1.02486200 -0.04446300  H 0.25190300 -0.98096700 -0.79625900  H 0.31592800 -0.87738600 0.95797300  H 0.80016900 1.58835500 0.77103200  H 0.66878900 1.45857500 -0.98799800  N -2.43636100 -1.25724600 0.06746700  H -2.47693200 -0.81079800 -0.86903400  O -1.08368100 1.21783600 -0.02689700  H -1.42691900 0.90105000 0.81799700 |
| CR2(pr) | CBS-QB3 | C 2.30037200 0.73792100 -0.11707800  C 0.89123600 0.37097600 0.35534600  H 2.64215000 1.66111000 0.35598800  H 3.02193300 -0.04693100 0.12987400  H 2.33202800 0.89059400 -1.20018700  C 0.37728100 -0.90867800 -0.28493600  H 0.87580800 0.24203400 1.44210900  H 0.19425800 1.18293200 0.12399200  H 0.34351300 -0.79614500 -1.37774900  H 1.05559100 -1.74099400 -0.05224700  N -2.97458300 1.20702300 -0.13361700  H -2.35798100 0.38611300 0.06195600  O -0.93165800 -1.17800900 0.23441300  H -1.24528800 -2.00511400 -0.14371400 |
|  | M06-2X | C 2.12522700 -0.55747900 0.13803900  C 0.85854100 -0.02344100 -0.52590300  H 2.72460600 -1.13616700 -0.56874900  H 2.74993000 0.25923800 0.51494500  H 1.88017400 -1.21130400 0.98115600  C 0.01684100 0.78354000 0.44490800  H 1.10711300 0.61100500 -1.38363000  H 0.25595600 -0.85679100 -0.90578000  H -0.25159600 0.16240700 1.31301900  H 0.59236200 1.64703300 0.80667700  N -1.99971300 -1.65403200 0.11473900  H -2.17044300 -0.73848100 -0.34810600  O -1.15912800 1.21977600 -0.23307900  H -1.62073800 1.86735800 0.30966000 |
| CR3(pr) | CBS-QB3 | C -2.30024800 -0.73789200 0.11691800  C -0.89098600 -0.37105100 -0.35519500  H -2.64182600 -1.66129500 -0.35588000  H -2.33227000 -0.89014200 1.20007800  H -3.02177000 0.04681800 -0.13058800  C -0.37714200 0.90867200 0.28505300  H -0.19409300 -1.18301200 -0.12360300  H -0.87527900 -0.24220200 -1.44196500  H -1.05566700 1.74086200 0.05251900  H -0.34321400 0.79609100 1.37786200  H 1.24501300 2.00569300 0.14317500  O 0.93165500 1.17826000 -0.23445100  N 2.97400000 -1.20734900 0.13358000  H 2.35812900 -0.38582600 -0.06170600 |
|  | M06-2X | C 2.13806200 0.75006400 0.00439300  C 0.64490900 0.43299600 -0.02991700  H 2.31009500 1.82769600 -0.04418200  H 2.59942300 0.37917000 0.92555700  H 2.65967900 0.28956500 -0.84109400  C 0.39057700 -1.06176900 0.03084000  H 0.13323100 0.91387300 0.81221500  H 0.19084500 0.83084300 -0.94538100  H 0.88872000 -1.55962000 -0.81328200  H 0.80438400 -1.47271800 0.96267300  H -1.20035300 -2.22602200 0.02254900  O -1.01516000 -1.28262100 -0.02584600  N -2.43058500 1.44714000 0.02296200  H -2.29193400 0.42045600 -0.06492200 |
| CR4(pr) | CBS-QB3 | C -0.55335200 1.63068100 -0.00007100  C 0.02578300 0.21432200 -0.00266400  H -1.64500000 1.60123200 -0.00311600  H -0.23735100 2.18986900 0.88611500  H -0.23244500 2.19517200 -0.88111600  C 1.54584200 0.18724400 0.00200400  H -0.32659300 -0.33946200 0.87397500  H -0.32110500 -0.33412500 -0.88483500  H 1.92864800 0.71662700 -0.88348900  H 1.92308900 0.71150900 0.89291300  H 2.92073800 -1.20045300 0.00186300  O 1.95975900 -1.17825900 -0.00062600  N -3.31017100 -0.93995800 0.00145200  H -2.62648800 -1.72806900 -0.00308000 |
|  | M06-2X | C -0.75173800 1.48112400 0.01861200  C -0.03535500 0.13541600 0.09348800  H -1.83260600 1.35941700 0.13030600  H -0.40401200 2.15887100 0.80542700  H -0.57420900 1.96725000 -0.94630900  C 1.46588200 0.27680500 -0.07429900  H -0.22385200 -0.34889400 1.05971800  H -0.40639000 -0.53955700 -0.68694400  H 1.68526700 0.73273400 -1.05139400  H 1.86591800 0.94129600 0.70646400  H 2.99306700 -0.95569200 -0.11103700  O 2.04162500 -1.01630600 0.02037600  N -2.99355000 -1.01232200 -0.14082600  H -2.55406600 -1.45879700 0.68974000 |
| TS1(pr) | CBS-QB3 | C 2.38111400 -0.41391000 0.16890700  C 0.97634100 -0.28699800 -0.42588000  H 2.96935200 -1.16446700 -0.36410500  H 2.92371600 0.53453100 0.10977000  H 2.34429800 -0.70986300 1.22192200  C 0.11216500 0.74239600 0.30690300  H 1.02891100 -0.00019200 -1.48065000  H 0.46924900 -1.25854800 -0.38920200  H 0.01204300 0.48377200 1.37318800  H 0.61503600 1.72428700 0.28081600  N -2.57314400 -0.88526900 0.21945900  H -2.10282700 -1.56106200 -0.40372000  O -1.13271800 0.97284200 -0.28474700  H -2.00374400 0.11676300 0.09417100 |
|  | M06-2X | C 2.30910300 -0.43446400 0.15393200  C 0.90506900 -0.28627500 -0.42782600  H 2.86896600 -1.21961700 -0.35961500  H 2.87460500 0.49815100 0.05747500  H 2.26978600 -0.69373000 1.21692800  C 0.10321500 0.78777700 0.29921700  H 0.94811100 -0.02714100 -1.49118900  H 0.36477100 -1.23838100 -0.34760500  H 0.01731700 0.56004900 1.37445300  H 0.62834700 1.75771900 0.22865600  N -2.34963000 -1.00473800 0.25770600  H -2.31730700 -1.37806100 -0.70354200  O -1.14672100 1.04216300 -0.26099100  H -1.93774600 0.03464200 0.15647900 |
| TS2(pr) | CBS-QB3 | C -1.99898000 0.32539200 0.13478800  C -0.89718700 -0.61060800 -0.36999100  H -2.91899700 0.18702300 -0.43734200  H -2.23075500 0.13437200 1.18681000  H -1.70222200 1.37383100 0.04651200  C 0.39794100 -0.45550400 0.38736800  H -1.22257300 -1.65637800 -0.28988200  H -0.70059700 -0.43364700 -1.43299700  H 0.78735700 0.82323300 0.25351200  H 0.29181500 -0.53593600 1.47759100  N 1.18003300 2.02603000 -0.00330700  H 1.19804800 1.95210200 -1.03878500  O 1.38439700 -1.28941800 -0.13727000  H 2.15186400 -1.26714700 0.44289600 |
|  | M06-2X | C -1.98050200 0.17085700 0.13469300  C -0.82544000 -0.68804600 -0.37708700  H -2.89285700 -0.02065800 -0.43488800  H -2.19204900 -0.04269100 1.18732700  H -1.74518600 1.23716200 0.05249900  C 0.43906200 -0.44094600 0.40128700  H -1.07986400 -1.75498600 -0.31348300  H -0.62601800 -0.47884200 -1.43500500  H 0.67339100 0.87944000 0.28694700  H 0.33584700 -0.56484700 1.48805300  N 0.90937500 2.09668600 -0.00662300  H 1.01079600 1.97811800 -1.03234100  O 1.50921500 -1.13690700 -0.14250500  H 2.27786400 -1.06542800 0.43393100 |
| TS3(pr) | CBS-QB3 | C 1.99898100 -0.35123500 0.07695900  C 0.58100100 -0.16623100 -0.40590800  H 2.67942100 0.34200900 -0.42234900  H 2.08193500 -0.18438600 1.15532300  H 2.36343200 -1.36824100 -0.12576100  C -0.47868700 -0.98161800 0.28203700  H 0.24702600 1.15034100 -0.11308300  H 0.46533100 -0.17547700 -1.49148300  H -0.33594800 -2.05056700 0.04744400  H -0.38701000 -0.87066400 1.37289300  H -2.43081400 -1.09891900 0.22621600  O -1.75922600 -0.53848900 -0.17308500  N -0.27926800 2.23216900 0.14422400  H -1.26246700 1.93314100 0.00738300 |
|  | M06-2X | C -1.98047100 -0.36865300 -0.08090400  C -0.57481000 -0.20160000 0.43458400  H -2.67793300 0.27453600 0.46069900  H -2.04234700 -0.11490300 -1.14440000  H -2.32815500 -1.40518800 0.03358100  C 0.49732700 -0.95420000 -0.30002700  H -0.24855100 1.13033800 0.15885100  H -0.46008100 -0.25354000 1.52025200  H 0.38419300 -2.03756300 -0.12476300  H 0.39824300 -0.77794900 -1.38216200  H 2.45986100 -1.03529200 -0.21736100  O 1.75926400 -0.50275500 0.17280500  N 0.22507000 2.19969400 -0.15490500  H 1.21288600 1.99047100 0.07528000 |
| TS4(pr) | CBS-QB3 | C -0.99149100 0.84092500 0.02468100  C 0.11026700 -0.18928000 -0.02357600  H -2.21682200 0.17296400 -0.11495600  H -1.11008600 1.36828200 0.97148700  H -1.05388200 1.51099100 -0.83334700  C 1.50841600 0.42942400 0.02355100  H 0.02671100 -0.88503000 0.81766900  H 0.04010200 -0.78494400 -0.93925100  H 1.63939100 1.10935500 -0.83056000  H 1.62068600 1.02007000 0.94434400  H 3.33396100 -0.26152000 -0.00297400  O 2.45006300 -0.63940700 -0.01982800  N -3.22807700 -0.50695200 -0.10650600  H -3.04717900 -1.07265900 0.74381200 |
|  | M06-2X | C -0.98243400 0.86435300 0.03311000  C 0.09740500 -0.18714500 0.00718900  H -2.22553700 0.19771000 -0.05454600  H -1.08872900 1.42161100 0.96557000  H -1.05363600 1.50052400 -0.85097100  C 1.49381300 0.42581900 -0.00265500  H 0.02417600 -0.84242300 0.88276300  H 0.00106400 -0.82038500 -0.88231900  H 1.60964000 1.06271800 -0.89155600  H 1.62643800 1.05637700 0.88859400  H 3.32269100 -0.28310000 -0.02776500  O 2.42860700 -0.63946900 -0.01475100  N -3.20578400 -0.49687600 -0.08320700  H -2.85718100 -1.31730500 0.44481700 |
| CP1(pr) | CBS-QB3 | C -2.16529500 -0.86645800 -0.12462000  C -0.80526000 -0.36843600 0.37019100  H -2.41030600 -1.83465600 0.31733600  H -2.96912400 -0.17100900 0.13734900  H -2.17277500 -0.98923700 -1.21221200  C -0.44742400 1.00730900 -0.20324800  H -0.80955800 -0.29009000 1.46265700  H -0.01260700 -1.07230200 0.10334900  H -0.40855900 0.94803300 -1.31498600  H -1.23062600 1.76256300 -0.00597700  N 2.61914000 -1.10041000 -0.14860000  H 3.60895900 -1.10072300 0.13497400  O 0.78410800 1.49517200 0.13492800  H 2.30562300 -0.14557700 0.08434800 |
|  | M06-2X | C 2.04944200 -0.75039300 0.16445700  C 0.90030100 -0.04958000 -0.55567900  H 2.61111000 -1.39261800 -0.51816500  H 2.75034600 -0.02533900 0.59166500  H 1.67213200 -1.37550300 0.98027500  C 0.13594600 0.87392600 0.38882600  H 1.27771400 0.54013100 -1.39865300  H 0.19522300 -0.78524900 -0.95857500  H -0.28770800 0.26672800 1.21757500  H 0.79141000 1.62378900 0.86380900  N -2.11450700 -1.24814100 0.17008600  H -2.60616900 -1.96643900 -0.37525100  O -0.96923600 1.47087900 -0.16158000  H -2.36275500 -0.35926300 -0.28626700 |
| CP2(pr) | CBS-QB3 | C 2.18793400 0.87475400 -0.18271100  C 0.87411700 0.32802400 0.38704200  H 2.44479900 1.82903900 0.28253100  H 3.01825000 0.18446600 -0.00339500  H 2.11159800 1.03323800 -1.26142500  C 0.49962100 -0.98165700 -0.21087900  H 0.96205600 0.23418700 1.48365800  H 0.06032200 1.04111500 0.21563800  H -3.62188600 1.07359600 -0.19201100  H 1.24688800 -1.75918300 -0.36349200  N -2.60563400 1.20669000 -0.09071100  H -2.24866800 0.24302700 -0.00566000  O -0.77483100 -1.41957800 0.09910600  H -0.90530300 -2.29642400 -0.27442900 |
|  | M06-2X | C -2.00703900 0.59085500 0.18257300  C -0.87872600 -0.13914600 -0.54510000  H -2.65278800 1.11563100 -0.52520700  H -2.62897800 -0.11017700 0.74893600  H -1.60362300 1.32697700 0.88433200  C -0.01400500 -0.90036100 0.39766800  H -1.29968600 -0.81958000 -1.30370000  H -0.25643000 0.57929900 -1.09439100  H 1.26171200 1.44582600 0.88147800  H -0.46312700 -1.53124200 1.16423000  N 1.71216700 1.78092100 0.01818000  H 2.13305300 0.92041400 -0.35697700  O 1.16398400 -1.34478400 -0.14937500  H 1.61144800 -1.94341300 0.45818700 |
| CP3(pr) | CBS-QB3 | C -2.17404900 0.92436100 -0.04609600  C -1.22642600 -0.12506100 0.41954600  H -2.65378900 1.43225600 0.79404300  H -1.67044800 1.68033600 -0.65957400  H -2.98509900 0.51048600 -0.66902300  C -0.22609300 -0.72630500 -0.49571500  H 2.11784100 0.40236000 0.36500700  H -1.39785500 -0.63936800 1.35912700  H -0.69712300 -1.49686900 -1.13549300  H 0.18657000 0.04017800 -1.16910500  H 1.35292600 -1.86841200 -0.30737000  O 0.81872000 -1.32896400 0.28432500  N 2.57576100 1.19507700 -0.11245300  H 2.92629600 1.76723800 0.66854900 |
|  | M06-2X | C 2.01917600 0.48989200 0.08022500  C 0.92417300 -0.36872900 -0.45224800  H 2.58277400 0.96717500 -0.72488600  H 1.61439100 1.27434800 0.73138600  H 2.74048700 -0.08419500 0.68341500  C -0.10395300 -0.92746400 0.46491100  H -0.82622400 1.46304400 -0.70063000  H 0.99887600 -0.81378400 -1.44013900  H 0.23235600 -1.89390800 0.88221800  H -0.26066600 -0.23644300 1.30698100  H -1.93044300 -1.62383800 0.27002600  O -1.31430700 -1.11331700 -0.26607200  N -1.33726100 1.86640100 0.09741200  H -2.31264100 1.62713400 -0.11901100 |
| CP4(pr) | CBS-QB3 | C 0.68189900 1.30459300 0.00001100  C -0.14246600 0.06317200 0.00008700  H 2.85761700 0.09491900 -0.00010300  H 0.91054600 1.81986300 -0.92581800  H 0.91057700 1.81996500 0.92577400  C -1.65558600 0.33387800 -0.00005600  H 0.07445100 -0.54849700 -0.88204600  H 0.07432800 -0.54830600 0.88238000  H -1.92316300 0.92214300 0.88870500  H -1.92302600 0.92195900 -0.88898000  H -3.25734700 -0.78105500 -0.00018200  O -2.30782300 -0.93215000 0.00002600  N 3.51008300 -0.70622900 -0.00002500  H 2.86494100 -1.51004800 -0.00001600 |
|  | M06-2X | C -0.67047100 1.33103100 -0.00022900  C 0.12246100 0.06701700 -0.00048700  H -2.79369000 0.07878300 -0.00013300  H -0.86620400 1.85872500 0.92644600  H -0.86747300 1.85839700 -0.92681600  C 1.63062200 0.32505900 0.00031000  H -0.10749700 -0.53991800 0.88313800  H -0.10675700 -0.53910400 -0.88484800  H 1.90353800 0.90973100 -0.88934800  H 1.90272900 0.90892900 0.89074600  H 3.22776900 -0.81456200 0.00088800  O 2.27305100 -0.93756000 0.00002200  N -3.44931200 -0.71749000 0.00092400  H -2.82730900 -1.53670900 -0.00428400 |
| CH_3_CH_2_CH_2_O | CBS-QB3 | C -1.82866200 -0.16733500 0.00466200  C -0.47549000 0.54809200 -0.00515600  H -2.65409600 0.54798600 -0.00005500  H -1.94638600 -0.81184800 -0.87233400  H -1.94190700 -0.79568600 0.89395300  C 0.70683000 -0.42370400 -0.00809900  H -0.39275400 1.19129900 -0.88724800  H -0.38115600 1.20106100 0.86823700  H 0.66714700 -1.09897000 0.87354000  H 0.65200500 -1.13158500 -0.86004900  O 1.94763500 0.14442800 0.00443900 |
|  | M06-2X | C -1.81726300 -0.16776800 0.00334100  C -0.47155400 0.55367800 -0.00373900  H -2.64758200 0.54225800 0.00098600  H -1.92598500 -0.81005500 -0.87716700  H -1.92197100 -0.79999500 0.89165200  C 0.69358000 -0.42802800 -0.00609500  H -0.38679000 1.19622200 -0.88694000  H -0.37739900 1.20337900 0.87306300  H 0.65666800 -1.09087400 0.88158700  H 0.63874800 -1.12550000 -0.86334300  O 1.94196600 0.14215900 0.00239000 |
| CH_3_CH_2_CHOH | CBS-QB3 | C -1.86916000 0.18425100 -0.07588100  C -0.51421700 -0.51042800 0.09719900  H -2.69042900 -0.53079300 0.01262700  H -2.01862100 0.95509000 0.68647600  H -1.94099500 0.66574200 -1.05459200  C 0.63009900 0.43468500 -0.01009400  H -0.49094500 -1.03270400 1.06961900  H -0.39029400 -1.29455000 -0.65944000  H 0.58215800 1.41351000 0.46655000  O 1.86541900 -0.16747700 -0.06509900  H 2.54544500 0.51247300 -0.06779100 |
|  | M06-2X | C -1.86023700 0.18654800 -0.06479300  C -0.51251900 -0.51807100 0.08461100  H -2.68679900 -0.52175800 0.03053300  H -1.98815900 0.95446600 0.70515600  H -1.93784300 0.67402400 -1.04093700  C 0.62455800 0.43493600 -0.02668100  H -0.47265100 -1.04604300 1.05212400  H -0.39651800 -1.29081800 -0.68522500  H 0.56468000 1.41348500 0.45065500  O 1.85698400 -0.16344200 -0.04984600  H 2.55060100 0.50370100 -0.07236100 |
| CH_3_CHCH_2_OH | CBS-QB3 | C 1.92894100 0.06626600 -0.06320900  C 0.53716300 -0.44916300 0.05303800  H 2.62686500 -0.72363300 -0.35164700  H 2.00280000 0.87118500 -0.80424600  H 2.30243700 0.48592800 0.88651600  C -0.62693800 0.47089300 0.08322400  H 0.35515500 -1.47297600 0.36059700  H -0.66784900 1.00349600 1.05557700  H -0.50700000 1.25128100 -0.68854100  H -2.56903700 0.30022400 0.05225000  O -1.82229500 -0.28043500 -0.11860300 |
|  | M06-2X | C -1.92175900 -0.07234900 -0.04341500  C -0.53426700 0.46561000 0.01632200  H -2.64632600 0.71162300 -0.27557500  H -2.01286600 -0.85541100 -0.80641100  H -2.23273800 -0.52781900 0.91086600  C 0.61929700 -0.46861700 0.06500400  H -0.34692100 1.49405400 0.30684300  H 0.62789000 -1.01823900 1.02662700  H 0.51658700 -1.22690600 -0.72995800  H 2.57258100 -0.31010900 0.03955200  O 1.81777100 0.27311900 -0.08742700 |
| CH_2_CH_2_CH_2_OH | CBS-QB3 | C -1.94377900 0.11976900 -0.00000800  C -0.59483300 -0.51206300 -0.00007700  H -2.42955100 0.40133700 0.92649700  H -2.42961600 0.40141400 -0.92645600  C 0.55714400 0.50485200 0.00006900  H -0.46316400 -1.14895000 0.88119800  H -0.46314600 -1.14872300 -0.88151200  H 0.47953400 1.14713100 -0.88849400  H 0.47951000 1.14690200 0.88879600  H 2.50488400 0.39366800 0.00015400  O 1.77629400 -0.23351600 -0.00001100 |
|  | M06-2X | C -1.93239700 -0.12421300 -0.00005500  C -0.59114400 0.52663400 -0.00020900  H -2.40136700 -0.43152500 -0.92720600  H -2.40289000 -0.42826900 0.92738800  C 0.54646900 -0.49591400 0.00033500  H -0.46418100 1.16323200 -0.88321300  H -0.46437400 1.16414000 0.88214700  H 0.46424600 -1.13588300 0.89045300  H 0.46417500 -1.13685800 -0.88907700  H 2.50560600 -0.40011400 -0.00002300  O 1.77015200 0.22077900 -0.00011200 |

**Table S66.** Cartesian coordinates (Å) of all optimized species in the n-C_4_H_9_OH+ NH reaction.

| Species | Method | Cartesian Coordindates |
| --- | --- | --- |
| C_4_H_9_OH | CBS-QB3 | C -2.39640500 -0.15735500 -0.13972400  C -0.96195400 -0.32223300 0.36863800  H -3.06435800 -0.90071800 0.30398500  H -2.79447000 0.83333100 0.10310100  H -2.44558500 -0.27502100 -1.22681200  C 0.00613100 0.70676100 -0.22534500  H -0.94879300 -0.23622900 1.46256600  H -0.59394300 -1.32444200 0.13352900  C 1.44806700 0.51952500 0.22299000  H -0.02149600 0.65102600 -1.31991800  H -0.31215600 1.72063000 0.04745000  H 2.06815600 1.33486400 -0.17618200  H 1.50416300 0.56185100 1.32146000  O 1.90487300 -0.74440900 -0.26019700  H 2.79445900 -0.89020700 0.07304700 |
|  | M06-2X | C -2.38511500 -0.14532100 -0.12444600  C -0.94541100 -0.34945500 0.34135100  H -3.05285000 -0.90655600 0.28861900  H -2.76340600 0.83539600 0.18378300  H -2.45230700 -0.19892500 -1.21626400  C 0.00080900 0.70802300 -0.22415600  H -0.90245500 -0.31850200 1.43836700  H -0.58774400 -1.33964600 0.04135700  C 1.43724600 0.52198900 0.22995600  H -0.02472500 0.67887500 -1.32107400  H -0.33337800 1.70894800 0.07957500  H 2.06438300 1.33559200 -0.16044300  H 1.48478400 0.55051200 1.32937000  O 1.89091700 -0.73414600 -0.25468700  H 2.79519000 -0.88393200 0.03797500 |
| CR1(bu) | CBS-QB3 | C -2.98627500 -0.65721200 -0.10865400  C -1.72022800 0.00448800 0.44144900  H -3.68569800 -0.90738900 0.69371100  H -3.50827700 0.00160600 -0.81017200  H -2.74918200 -1.58365600 -0.64139600  C -0.71120800 0.37777600 -0.64905800  H -1.99210300 0.90889100 0.99976800  H -1.23144600 -0.66068700 1.15911200  C 0.58744100 0.97375200 -0.10621100  H -0.47080000 -0.51075400 -1.24905200  H -1.16033600 1.10079100 -1.34281900  H 1.22947400 1.29244400 -0.93860400  H 0.37081000 1.85953500 0.49797100  O 1.29260300 0.08501500 0.76101300  H 1.62698800 -0.64659300 0.22710600  N 3.70816200 -0.79812200 -0.34282200  H 4.25423800 -0.14028400 0.25086900 |
|  | M06-2X | C 3.03988900 -0.03419700 -0.42567000  C 1.71968100 0.36891000 0.22651600  H 3.80834500 0.73022600 -0.28135600  H 3.41691800 -0.97266800 -0.00506100  H 2.91330800 -0.18186900 -1.50354700  C 0.63393000 -0.69090900 0.04990700  H 1.87338900 0.54408600 1.29933500  H 1.36449200 1.31636800 -0.19318300  C -0.70823000 -0.27354200 0.63641700  H 0.50281100 -0.91380600 -1.01964400  H 0.94731000 -1.63141900 0.52296900  H -1.43000300 -1.10069700 0.56472300  H -0.59405200 -0.01667400 1.69488200  O -1.23426200 0.89415400 0.01937100  H -1.41665400 0.69112700 -0.90690600  N -3.69308100 -0.30715600 -0.51016900  H -3.77181700 0.31062000 0.32098300 |
| CR2(bu) | CBS-QB3 | C -1.85560500 -0.22533800 0.31847600  C -0.43113600 -0.09405200 -0.23201700  H -2.27228500 -1.19548600 0.02214200  H -1.82411600 -0.22815800 1.41464400  C 0.49290300 -1.20294900 0.24503800  H -0.44891900 -0.10647800 -1.32729300  H -0.00590400 0.87105000 0.06536300  H 0.56521700 -1.18896800 1.34148800  H 0.09054800 -2.18021600 -0.05446800  N 3.01887800 1.86287500 0.18242000  H 2.66879100 0.91136400 -0.07129000  O 1.78338200 -0.99905800 -0.34662200  H 2.36119300 -1.71730200 -0.07138100  C -2.78395900 0.89448900 -0.15983800  H -2.86375200 0.90187400 -1.25107200  H -3.79168400 0.77663700 0.24700100  H -2.41150800 1.87512100 0.15094200 |
|  | M06-2X | C -1.59460200 -0.17633700 0.49994100  C -0.33251500 -0.29471100 -0.35199400  H -1.87184700 -1.16680600 0.88413100  H -1.38157000 0.45032200 1.37521700  C 0.83080800 -0.88006100 0.42572400  H -0.52310200 -0.92420500 -1.23001700  H -0.04772000 0.69773600 -0.72521500  H 1.03373500 -0.26473500 1.31559600  H 0.58076400 -1.89568200 0.76215400  N 1.96303600 2.06237500 0.15929200  H 2.36574500 1.26443200 -0.37297800  O 1.97493600 -0.90712500 -0.42569300  H 2.66478500 -1.44206900 -0.01940000  C -2.76525600 0.41645800 -0.28091500  H -3.00808000 -0.20228900 -1.15103400  H -3.66294300 0.49145300 0.33878400  H -2.52110800 1.42012700 -0.64327000 |
| CR3(bu) | CBS-QB3 | C -2.51804700 0.97002300 -0.08448400  C -1.46070000 -0.07710600 -0.44317200  H -3.18010000 1.17206100 -0.93063800  H -3.14112800 0.63810200 0.75236200  H -2.05302900 1.91716200 0.20558200  C -0.51383200 -0.39244900 0.71991700  H -1.95457500 -1.00325500 -0.76303300  H -0.86961200 0.26681700 -1.29635200  C 0.57582100 -1.39455800 0.36927700  H -0.03948900 0.53041800 1.07424000  H -1.08358300 -0.79375500 1.56688200  H 1.17176200 -1.63021500 1.26071100  H 0.12397600 -2.32752500 0.00538600  O 1.40987900 -0.82297700 -0.65019000  H 2.03723400 -1.49076600 -0.94255000  N 2.70090600 1.94639900 0.15598100  H 2.30370900 1.04451800 -0.19216100 |
|  | M06-2X | C -2.36958600 -0.26191100 -0.06293000  C -0.94953700 -0.56126900 -0.53596300  H -3.05411500 -0.14059200 -0.90691600  H -2.75310800 -1.07168100 0.56764300  H -2.39015300 0.66318600 0.52188600  C 0.01261700 -0.77232800 0.63246800  H -0.94378000 -1.45847900 -1.16923700  H -0.58772900 0.26297400 -1.16162100  C 1.45655200 -0.95113900 0.19925600  H -0.04598600 0.08064300 1.32272700  H -0.28916900 -1.65936200 1.20421600  H 2.08706500 -1.18817300 1.06599100  H 1.53183200 -1.77797700 -0.52154900  O 1.89332600 0.26468200 -0.40616600  H 2.79613900 0.16394500 -0.72394400  N -0.16031600 2.46722300 0.24566500  H 0.72433100 2.01737200 -0.06651100 |
| CR4(bu) | CBS-QB3 | C 2.48100600 0.92284100 -0.16659800  C 1.06210900 0.48739100 0.20855900  H 2.62766200 1.99128700 0.01114300  H 3.23139600 0.38197600 0.41894700  H 2.68580200 0.73126100 -1.22445600  C 0.81783700 -1.00724800 -0.02831300  H 0.87335200 0.72168300 1.26346600  H 0.33788600 1.06789500 -0.36991900  C -0.57230000 -1.48739900 0.35910300  H 0.98282100 -1.24756000 -1.08489500  H 1.54650900 -1.59321100 0.54567000  H -0.63406900 -2.57481500 0.22796800  H -0.77163700 -1.26098200 1.41633600  O -1.54048800 -0.83853200 -0.48052100  H -2.38661100 -1.27957000 -0.35881000  N -2.40373100 2.15512800 0.12462600  H -2.07500300 1.19088600 -0.11017300 |
|  | M06-2X | C 2.57445400 0.39299200 -0.18634000  C 1.05358600 0.37464200 -0.04918600  H 2.94165300 1.39220900 -0.43535800  H 3.05527800 0.08115900 0.74706800  H 2.90335500 -0.29200000 -0.97500000  C 0.52522600 -1.01651400 0.29661400  H 0.74176400 1.08478600 0.72869500  H 0.59522600 0.71009300 -0.98701800  C -0.98051300 -1.06478900 0.48214100  H 0.80434500 -1.72571900 -0.49307400  H 0.99430600 -1.36682600 1.22557700  H -1.29234000 -2.07963400 0.75877800  H -1.28412500 -0.37832100 1.28789400  O -1.59997100 -0.67648700 -0.74455200  H -2.52431700 -0.94584600 -0.73324800  N -1.64876500 2.18963000 0.29239500  H -1.63054600 1.48659300 -0.47404500 |
| CR5(bu) | CBS-QB3 | C 1.29208200 1.75347800 -0.12183400  C -0.02555800 1.12958800 0.34505600  H 1.39978000 2.77949900 0.24052100  H 2.15184900 1.18188400 0.24384300  H 1.34960900 1.78169300 -1.21492400  C -0.21177100 -0.31103600 -0.14309000  H -0.06696200 1.14361900 1.44133000  H -0.86956200 1.73054000 -0.00407400  C -1.53786100 -0.92996000 0.27238700  H -0.15124300 -0.34011300 -1.23795300  H 0.60060300 -0.94184400 0.23858900  H -1.56868200 -1.98332100 -0.04039800  H -1.63274600 -0.90227500 1.36842600  O -2.59053500 -0.18868300 -0.34730300  H -3.42906900 -0.54024400 -0.03590300  N 3.19994000 -1.58361800 0.07376500  H 3.43976900 -1.16707000 -0.85250200 |
|  | M06-2X | C -1.52735200 1.48095300 0.08860400  C -0.07853300 1.12820900 -0.24060000  H -1.71431200 2.55299500 -0.02062900  H -2.22240200 0.94925500 -0.57124600  H -1.76816800 1.20954200 1.12336600  C 0.20180800 -0.36698500 -0.10197500  H 0.15226600 1.44020700 -1.26782600  H 0.60330200 1.67863000 0.41575900  C 1.64373000 -0.73136600 -0.40622700  H -0.02492400 -0.69194800 0.92336100  H -0.45260200 -0.93516300 -0.77650900  H 1.77829700 -1.81987400 -0.34060700  H 1.89677500 -0.41598800 -1.42977500  O 2.47166900 -0.06775300 0.53875600  H 3.39482500 -0.26218800 0.34910300  N -2.88006100 -1.55491600 0.03061200  H -2.69390100 -1.34390000 1.03186000 |
| TS1(bu) | CBS-QB3 | C -2.70016300 0.72358200 -0.05288600  C -1.49251000 -0.13289700 -0.44177000  H -3.38387900 0.85188300 -0.89613300  H -3.26505500 0.26800800 0.76653900  H -2.38944600 1.72058200 0.27558800  C -0.50691800 -0.34506500 0.71105100  H -1.83684500 -1.11047000 -0.80099400  H -0.95778200 0.32796000 -1.27724600  C 0.74483000 -1.13105600 0.31042500  H -0.19668800 0.62826200 1.11443600  H -0.99822400 -0.87479600 1.53700200  H 1.38560000 -1.32415300 1.18376100  H 0.44668800 -2.12223300 -0.07193700  O 1.47337800 -0.56085200 -0.73909700  H 2.28033900 0.34613900 -0.35081700  N 2.70789800 1.32002100 0.11263500  H 1.90154500 1.84809800 0.48321800 |
|  | M06-2X | C 2.60960200 -0.64452400 -0.03552000  C 1.37984100 0.15642600 -0.45686800  H 3.29317600 -0.79859100 -0.87469900  H 3.16170200 -0.12934400 0.75774000  H 2.31963300 -1.62926800 0.34545800  C 0.41709400 0.38808300 0.70502900  H 1.68916200 1.12763400 -0.86514000  H 0.85085900 -0.36430200 -1.26212100  C -0.83103700 1.17117700 0.30599000  H 0.11024300 -0.57980400 1.12758600  H 0.92317900 0.93361800 1.51231900  H -1.47855800 1.37192300 1.17380000  H -0.53708700 2.15621100 -0.09953200  O -1.55454700 0.59339800 -0.73706600  H -1.79346500 -0.70167300 -0.48265700  N -2.13056300 -1.61617500 0.07931900  H -2.64152300 -1.24733700 0.89676600 |
| TS2(bu) | CBS-QB3 | C -1.41949100 0.25296300 0.41215400  C -0.34412200 -0.64287700 -0.21399700  H -1.47313400 0.05457300 1.48913300  H -1.12216000 1.30246100 0.31125900  C 1.02253600 -0.44749200 0.39300400  H -0.62710500 -1.69931400 -0.10461200  H -0.27635100 -0.45638600 -1.29230600  H 1.35329800 0.84304400 0.22605400  H 1.04471100 -0.53609400 1.48735200  N 1.67282200 2.06045700 -0.06198700  H 1.56405300 1.99623800 -1.09253300  O 1.97017800 -1.24587200 -0.24679600  H 2.79781200 -1.19869400 0.24204600  C -2.80166200 0.04709700 -0.21301400  H -3.13920700 -0.98731000 -0.09630000  H -3.54800500 0.69537900 0.25323200  H -2.78866200 0.27173600 -1.28392700 |
|  | M06-2X | C -1.39486700 0.22318200 0.41882500  C -0.31929300 -0.66078700 -0.21213200  H -1.44999800 0.01537500 1.49494200  H -1.10231000 1.27680700 0.32282600  C 1.03505700 -0.44688400 0.40851800  H -0.59646300 -1.72110400 -0.11510900  H -0.24417000 -0.45810400 -1.28891900  H 1.29019600 0.86565100 0.26386000  H 1.06241200 -0.57164400 1.49957400  N 1.52248900 2.07558600 -0.06330900  H 1.44869900 1.96050600 -1.09184700  O 2.01319700 -1.16813700 -0.26148500  H 2.84771200 -1.11896900 0.21754700  C -2.76554900 0.00545200 -0.21768000  H -3.08731400 -1.03533300 -0.10763100  H -3.52501900 0.64290500 0.24267800  H -2.73882800 0.23412600 -1.28806700 |
| TS3(bu) | CBS-QB3 | C -2.45045400 -0.11381600 -0.16510600  C -1.06884600 -0.76218600 -0.30070000  H -3.15419000 -0.53307900 -0.88876500  H -2.86612300 -0.27309200 0.83476100  H -2.39555500 0.96467800 -0.33351100  C -0.05465000 -0.23540800 0.69184500  H -1.16201400 -1.85143600 -0.16283300  H -0.68126200 -0.62170800 -1.31386900  C 1.36357500 -0.71982000 0.55000800  H 0.02787400 1.13330900 0.46182800  H -0.39700700 -0.24832100 1.72979000  H 1.98704600 -0.29332500 1.34754700  H 1.39455300 -1.81792400 0.65448500  O 1.85421000 -0.33354200 -0.73936300  H 2.71648800 -0.73898000 -0.86791700  N 0.27740100 2.27439800 0.05949200  H 1.01696000 2.01481800 -0.61932800 |
|  | M06-2X | C -2.40039500 -0.15827900 -0.16408600  C -1.01718800 -0.78677700 -0.33114500  H -3.10197900 -0.53900300 -0.91092300  H -2.81164600 -0.37578000 0.82726000  H -2.34441000 0.92970900 -0.27051300  C -0.03268000 -0.29881000 0.70444300  H -1.09526700 -1.88388500 -0.25977400  H -0.61866500 -0.57228100 -1.32932400  C 1.40335300 -0.70596500 0.52264400  H -0.01468000 1.09152800 0.52423300  H -0.37921700 -0.38006800 1.73894400  H 2.01691100 -0.31834500 1.34687200  H 1.48246200 -1.80659700 0.52608000  O 1.85651200 -0.18966700 -0.72551400  H 2.72997900 -0.54263100 -0.92274200  N 0.11014200 2.22655600 0.11183600  H 0.79487800 2.02778200 -0.63998900 |
| TS4(bu) | CBS-QB3 | C 2.01024700 -1.17027200 -0.17790900  C 0.72836600 -0.38223900 -0.31423400  H 2.76550900 -0.84430200 -0.89795200  H 2.43608500 -1.07508800 0.82598000  H 1.83234300 -2.23995700 -0.35483100  C -0.36065800 -0.67438100 0.69522900  H 1.05792400 0.91972200 -0.04867700  H 0.34568900 -0.32384700 -1.33370000  C -1.60420000 0.18748900 0.52036200  H -0.66031600 -1.73019000 0.61266200  H 0.02270600 -0.53787800 1.71362100  H -2.32453500 -0.04173300 1.31768100  H -1.33008400 1.24796200 0.60283400  O -2.16224400 -0.09940100 -0.76360600  H -2.89088000 0.50847700 -0.91615600  N 1.37765900 2.11621100 0.14838700  H 1.75737000 2.33498800 -0.79201400 |
|  | M06-2X | C 2.10572600 -1.02191800 -0.17643700  C 0.75296900 -0.37846800 -0.35226900  H 2.81894800 -0.67838500 -0.93056900  H 2.52026000 -0.80026600 0.81287900  H 2.02951800 -2.11473100 -0.26510100  C -0.28333300 -0.69991000 0.69781400  H 0.97698200 0.95424200 -0.12220000  H 0.35654500 -0.40808500 -1.36976800  C -1.56246300 0.10282300 0.52940000  H -0.53283500 -1.77156400 0.65336200  H 0.12775600 -0.50659800 1.69725300  H -2.27540300 -0.15888500 1.32271200  H -1.33502100 1.17660300 0.61051700  O -2.09945100 -0.20192700 -0.75038800  H -2.91258200 0.29480500 -0.88409800  N 1.14051900 2.15022600 0.15263200  H 0.96040600 2.56153300 -0.78135400 |
| TS5(bu) | CBS-QB3 | C -1.57468700 1.10662400 0.17967200  C -0.22210300 0.72769000 -0.37040000  H -2.10985300 1.88282500 -0.36736900  H -2.37508100 -0.02082100 -0.02035700  H -1.61853300 1.25702200 1.25941500  C 0.42603900 -0.45309100 0.36374500  H -0.31175800 0.49034900 -1.43781200  H 0.45594200 1.58944800 -0.30818600  C 1.82177000 -0.78152900 -0.14305400  H 0.49388600 -0.22733700 1.43430700  H -0.20452700 -1.34277500 0.26192600  H 2.20137100 -1.67357000 0.37444500  H 1.78268200 -1.00821300 -1.21910800  O 2.66316400 0.34545400 0.10837000  H 3.52864900 0.16570400 -0.26892200  N -3.12765900 -0.98052100 -0.15629500  H -3.96059100 -0.61077600 0.33898400 |
|  | M06-2X | C -1.59072800 1.16633900 0.18358100  C -0.23999200 0.77273300 -0.35537700  H -2.13974600 1.91991200 -0.38239700  H -2.34817400 0.01369700 -0.04565200  H -1.64127600 1.32371800 1.26321700  C 0.37028500 -0.40384400 0.40671800  H -0.32942100 0.51301500 -1.41864200  H 0.45017600 1.62629300 -0.29983600  C 1.73975600 -0.79243000 -0.11889800  H 0.46403100 -0.14640900 1.46964800  H -0.29455200 -1.27453600 0.33524400  H 2.11712500 -1.66747600 0.42758800  H 1.66549200 -1.05847400 -1.18392900  O 2.60557100 0.32034700 0.05798900  H 3.47767100 0.10783000 -0.28861900  N -2.92141600 -1.04059500 -0.23744800  H -3.49190300 -1.09296500 0.62545600 |
| CP1(bu) | CBS-QB3 | C -2.44264900 0.31053900 -0.03781500  C -1.15440600 -0.36588800 -0.51035300  H -3.08394400 0.57540200 -0.88284400  H -3.01833100 -0.34573200 0.62302300  H -2.21538600 1.22837600 0.51083500  C -0.23848000 -0.78054200 0.64719700  H -1.39558500 -1.24773900 -1.11770200  H -0.60398000 0.32039400 -1.16266300  C 1.08612500 -1.37255900 0.16746300  H -0.02720300 0.09148600 1.27406700  H -0.74541900 -1.52062500 1.27898400  H 1.68630600 -1.74738300 1.02502300  H 0.94250600 -2.27854500 -0.45286400  O 1.92731700 -0.51428100 -0.48906200  H 1.45593500 1.65321900 -0.12441300  N 0.94064700 2.48103300 0.21253200  H 1.49849300 3.26886200 -0.14562600 |
|  | M06-2X | C -2.33925900 -0.27778900 -0.04510200  C -0.93873700 -0.66769500 -0.50661900  H -3.03892800 -0.24076500 -0.88469800  H -2.72765100 -0.99604100 0.68552200  H -2.31293100 0.71282200 0.41869500  C 0.04162400 -0.78681200 0.65952500  H -0.96611300 -1.62063300 -1.05251500  H -0.56698800 0.08924600 -1.20783700  C 1.47441100 -0.98219800 0.18458900  H -0.00623100 0.12225000 1.27355300  H -0.24228900 -1.62861000 1.30467200  H 2.17137200 -1.14380700 1.03070000  H 1.58835700 -1.88451000 -0.44359400  O 2.00171400 0.09131900 -0.49810500  H 0.74472700 1.97843900 -0.34451400  N -0.06233700 2.42660300 0.11116700  H 0.35108700 3.18180600 0.67233400 |
| CP2(bu) | CBS-QB3 | C -1.87085600 -0.15120800 0.39945700  C -0.51265200 -0.19970800 -0.31364800  H -2.36311300 -1.12618400 0.30251200  H -1.70628400 0.00349900 1.47104900  C 0.38211100 -1.26312600 0.21568200  H -0.67557900 -0.33571900 -1.39766000  H -0.00343600 0.76789500 -0.21381600  H 2.32639700 2.31839100 0.11004300  H -0.00250800 -2.25646200 0.43932000  N 3.13383500 1.67974300 0.15357000  H 2.70360500 0.76022000 -0.02705100  O 1.68254900 -1.19780100 -0.24059200  H 2.18229800 -1.94333500 0.10665400  C -2.79301500 0.94107500 -0.14722500  H -3.00155900 0.78924200 -1.21067700  H -3.74997100 0.95301000 0.38084200  H -2.34061900 1.93145200 -0.03706700 |
|  | M06-2X | C -1.43571800 -0.16972200 0.53085600  C -0.26050400 -0.53900600 -0.37648800  H -1.64366600 -1.00213300 1.21566200  H -1.14809700 0.68573000 1.15361300  C 0.95478800 -0.92067000 0.39302400  H -0.56314100 -1.35877300 -1.05132900  H -0.01392600 0.31250300 -1.02501900  H 1.22927800 1.68026500 0.90159900  H 0.89839400 -1.68661000 1.16568300  N 1.28838000 2.23852600 0.03841300  H 2.01639600 1.74418100 -0.49432200  O 2.12433800 -0.85533700 -0.32277400  H 2.84875900 -1.23889800 0.18286700  C -2.69194700 0.17193900 -0.26622600  H -3.01031900 -0.67773200 -0.87922500  H -3.52184600 0.44155200 0.39232200  H -2.50491300 1.01767500 -0.93554900 |
| CP3(bu) | CBS-QB3 | C -2.32939200 0.84234000 -0.13827000  C -1.46720400 -0.41211500 -0.33698200  H -3.09471900 0.92015200 -0.91465700  H -2.83723600 0.82513600 0.83086500  H -1.71569500 1.74602000 -0.17837700  C -0.43576500 -0.60143100 0.72409300  H -2.13101500 -1.29517200 -0.36342400  H -0.97523900 -0.38114500 -1.31330100  C 0.83366800 -1.32971400 0.46301200  H 1.03815800 2.23045800 0.70735500  H -0.69988400 -0.39797700 1.75756900  H 1.50867900 -1.23641500 1.32349800  H 0.63449300 -2.40804000 0.31895900  O 1.44878800 -0.80661900 -0.72826600  H 2.16718500 -1.39505800 -0.98112500  N 1.87080500 2.22340300 0.09970500  H 1.81149200 1.28669300 -0.33028700 |
|  | M06-2X | C -2.32898200 -0.36730300 -0.05139300  C -0.91830400 -0.72361500 -0.52009500  H -3.02882100 -0.35058100 -0.89063300  H -2.69459400 -1.09575900 0.67998100  H -2.34229900 0.62314300 0.41292800  C 0.06043900 -0.83544100 0.60109800  H -0.94671000 -1.68065900 -1.07125400  H -0.56089500 0.02105500 -1.24185700  C 1.52349000 -0.87283600 0.32823800  H -0.35100200 2.01461200 1.09854000  H -0.28554700 -1.18064400 1.57173300  H 2.08924400 -0.86614700 1.26839800  H 1.78512700 -1.79867400 -0.21585400  O 1.87600000 0.25474900 -0.47632900  H 2.77128600 0.14322200 -0.81289600  N -0.28724400 2.42402000 0.15627500  H 0.54706200 1.95947600 -0.22946000 |
| CP4(bu) | CBS-QB3 | C 2.19874800 -1.14191200 -0.16456000  C 0.80734400 -0.63673700 -0.34758400  H 2.88022900 -0.75397300 -0.92623400  H 2.60103400 -0.88030600 0.82136200  H 2.24339600 -2.24252500 -0.23069200  C -0.22695000 -0.83573100 0.71098800  H 0.98368400 1.83443000 -0.17173500  H 0.44619500 -0.44326800 -1.35255000  C -1.49301500 -0.00936500 0.50499700  H -0.52304700 -1.89940700 0.74916000  H 0.18802800 -0.60872500 1.70206900  H -2.18431900 -0.18919600 1.34016200  H -1.24087100 1.05747800 0.49035000  O -2.08318000 -0.41324900 -0.73322200  H -2.77305100 0.21879500 -0.95263100  N 0.69110200 2.79846900 0.06153600  H 1.48968000 3.36587000 -0.25728100 |
|  | M06-2X | C 1.98431300 -1.34661300 -0.09018600  C 0.60646400 -0.83884200 -0.35703000  H 2.55836800 -1.47995300 -1.01100300  H 2.53805500 -0.66550100 0.56837800  H 1.96117400 -2.32280100 0.42121100  C -0.25216800 -0.39985700 0.78392500  H 1.32613200 1.44294000 -0.58323700  H 0.10507700 -1.11810300 -1.27937700  C -1.46100000 0.41159400 0.34677000  H -0.61721000 -1.27935900 1.34252400  H 0.33635500 0.20053400 1.49037400  H -2.04715300 0.70875700 1.22632700  H -1.12514800 1.32594500 -0.16554300  O -2.23609300 -0.39849400 -0.52592700  H -3.00261200 0.09799700 -0.82901800  N 1.32386100 2.32296800 -0.04456300  H 1.32302600 3.05902600 -0.76215700 |
| CP5(bu) | CBS-QB3 | C -1.29353000 1.65814000 0.17127100  C -0.12775500 0.92215800 -0.39141800  H -1.89041300 2.31002700 -0.45689300  H -2.80982700 -0.30013500 0.01717800  H -1.41103600 1.75979400 1.24529600  C 0.33844900 -0.26252100 0.46873500  H -0.36081300 0.57214300 -1.40604300  H 0.72622100 1.60841100 -0.51208200  C 1.60438200 -0.91891400 -0.05868900  H 0.53000700 0.08211500 1.49141400  H -0.45302400 -1.01636300 0.51956900  H 1.84950700 -1.79332800 0.55998100  H 1.43941900 -1.27171300 -1.08773400  O 2.66014800 0.04351800 -0.01489000  H 3.44163400 -0.34800700 -0.41454500  N -3.05552200 -1.29601200 -0.11395600  H -4.08348700 -1.27217200 -0.17871200 |
|  | M06-2X | C -1.53956600 1.63583500 0.08483600  C -0.32434000 0.90894600 -0.38200300  H -2.21660400 2.09234400 -0.62884500  H -2.56270800 -0.65243000 -0.13330700  H -1.61560000 1.96026200 1.11819700  C 0.31766500 0.05665100 0.71425500  H -0.57465500 0.26873300 -1.23924400  H 0.43064800 1.61896200 -0.75409400  C 1.46021200 -0.79111700 0.18951000  H 0.68805800 0.70434600 1.51899700  H -0.43161300 -0.61563100 1.15175700  H 1.87579200 -1.40536000 1.00037800  H 1.08116500 -1.46317400 -0.59407300  O 2.45163700 0.08432700 -0.33193200  H 3.16481000 -0.43314500 -0.71835400  N -2.29705800 -1.63530000 -0.29828500  H -2.85681500 -2.16431900 0.38246400 |
| CH_3_CH_2_CH_2_CH_2_O | CBS-QB3 | C -2.35425000 -0.12232500 -0.13912200  C -0.91700700 -0.34305900 0.33801900  H -3.02600000 -0.88899600 0.25595300  H -2.73641900 0.85174500 0.18250000  H -2.41671500 -0.15744400 -1.23114400  C 0.06398600 0.70935100 -0.18704500  H -0.89044800 -0.34476700 1.43510000  H -0.56551800 -1.32967600 0.02017700  C 1.51675400 0.41621700 0.19823900  H 0.00257300 0.75961300 -1.28055500  H -0.20638900 1.70437500 0.18849900  H 2.19966300 1.23680400 -0.09254600  H 1.62638100 0.37305600 1.30378700  O 2.01949700 -0.77072700 -0.25529000 |
|  | M06-2X | C -2.34422300 -0.11431900 -0.13116600  C -0.90949800 -0.35645200 0.33007700  H -3.02549300 -0.87394800 0.26162700  H -2.70405000 0.86434900 0.20396400  H -2.41062100 -0.13715700 -1.22383600  C 0.06140800 0.69586800 -0.20246700  H -0.86802900 -0.36168600 1.42752800  H -0.57061800 -1.34532300 -0.00095100  C 1.49671500 0.41870300 0.22524600  H 0.02018400 0.71813200 -1.29871200  H -0.23294100 1.69260800 0.15249300  H 2.18745100 1.21409000 -0.11453200  H 1.60235800 0.41340800 1.32633800  O 2.02191900 -0.75590900 -0.25800800 |
| CH_3_CH_2_CH_2_CHOH | CBS-QB3 | C -1.18521500 0.53028600 -0.09004700  C 0.06348700 -0.34483900 0.08216900  H -1.18055400 1.31874000 0.67225500  H -1.13247600 1.04058800 -1.05778600  C 1.33010600 0.43086400 -0.00366000  H 0.00189300 -0.87835100 1.04790600  H 0.08070100 -1.12950600 -0.68516300  H 1.41741600 1.40012100 0.48630700  O 2.46927600 -0.33828600 -0.06120600  H 3.23788200 0.23964100 -0.05012400  C -2.49082600 -0.26252500 0.00778200  H -2.58288300 -0.75635300 0.98010600  H -3.36104100 0.38707500 -0.11883400  H -2.54046500 -1.03838500 -0.76247900 |
|  | M06-2X | C -1.17912100 0.53419500 -0.08937600  C 0.05933200 -0.34690700 0.07758000  H -1.17047000 1.31956400 0.67756400  H -1.12598900 1.04485400 -1.05812400  C 1.32317500 0.43258700 -0.00717100  H -0.00092300 -0.88241800 1.04171800  H 0.07466700 -1.12472600 -0.69785000  H 1.40703900 1.39592700 0.49626000  O 2.45585100 -0.33691100 -0.05449700  H 3.23861200 0.22342200 -0.07056600  C -2.47686500 -0.26361300 0.00932600  H -2.55865800 -0.75943100 0.98222100  H -3.35269600 0.37936600 -0.11442100  H -2.51751700 -1.03884500 -0.76298100 |
| CH_3_CH_2_CHCH_2_OH | CBS-QB3 | C -2.35067900 -0.16359500 -0.23743200  C -0.97277700 -0.25577800 0.43031800  H -3.04406600 -0.89457000 0.18686900  H -2.79051400 0.82965600 -0.10314000  H -2.27649400 -0.35219200 -1.31167400  C 0.00744300 0.74298300 -0.08485800  H -1.09781800 -0.12299500 1.52108100  H -0.55479100 -1.25907100 0.30647900  C 1.47469300 0.55926300 0.07120200  H -0.34658800 1.72685800 -0.37885900  H 2.00949600 1.26748700 -0.57813100  H 1.77647100 0.79118100 1.11242900  O 1.83121200 -0.79245300 -0.23970200  H 2.72253000 -0.94396200 0.08718400 |
|  | M06-2X | C -2.38481000 -0.09962400 -0.14920700  C -0.94795400 -0.36518500 0.29696300  H -3.05502800 -0.90213000 0.16993100  H -2.75917900 0.83919300 0.27220500  H -2.44224700 -0.02363200 -1.23932400  C -0.00245000 0.71002300 -0.11949300  H -0.91703800 -0.47879300 1.39532000  H -0.59239500 -1.32414600 -0.09954000  C 1.45683100 0.57282400 0.13279700  H -0.38648700 1.69413300 -0.37352800  H 2.01180900 1.33530700 -0.43091900  H 1.67311400 0.73333100 1.20663000  O 1.87038200 -0.73772200 -0.24052300  H 2.77468900 -0.87971000 0.05704800 |
| CH_3_CHCH_2_CH_2_OH | CBS-QB3 | C 2.44007900 -0.16780800 -0.01543100  C 0.96977800 -0.35538700 -0.16616400  H 3.00552300 -0.91915300 -0.57291200  H 2.76129200 0.82343800 -0.35856300  H 2.76144400 -0.24567700 1.03770900  C 0.00495000 0.70348500 0.25098500  H 0.57501900 -1.35337300 -0.32049000  C -1.40251500 0.50159200 -0.29938900  H -0.07367300 0.74546100 1.35216400  H 0.36655300 1.69426400 -0.05693300  H -2.03679100 1.35106200 -0.00889100  H -1.36426900 0.46236000 -1.39723000  O -1.91380400 -0.71866500 0.23625100  H -2.75841900 -0.90035200 -0.18486100 |
|  | M06-2X | C 2.42660600 -0.16720800 -0.00987400  C 0.95843100 -0.35892700 -0.18180900  H 3.00125200 -0.93678600 -0.53153600  H 2.74765500 0.81261400 -0.38502500  H 2.72200800 -0.20549600 1.05167000  C 0.00663400 0.70597700 0.24984100  H 0.56091200 -1.36065200 -0.31245100  C -1.39746200 0.50280500 -0.29669600  H -0.06316900 0.74563800 1.35130600  H 0.37235400 1.69255900 -0.06827300  H -2.04042200 1.34327400 -0.00123800  H -1.36018400 0.46066100 -1.39480600  O -1.89376800 -0.71741600 0.23387000  H -2.75550900 -0.90836200 -0.14938100 |
| CH_2_CH_2_CH_2_CH_2_OH | CBS-QB3 | C -2.42364000 -0.15840900 -0.15918100  C -1.03364700 -0.32207400 0.34756000  H -3.27015900 -0.54347200 0.39597700  H -2.60412800 0.20346300 -1.16525200  C -0.05325300 0.73426500 -0.18695100  H -1.03038700 -0.30478200 1.44562800  H -0.63697200 -1.31252800 0.07225200  C 1.38428600 0.50056600 0.25200900  H -0.08221800 0.73776300 -1.28242100  H -0.36455700 1.73130400 0.14372200  H 2.01580600 1.32864500 -0.09978300  H 1.43942900 0.47988300 1.35103700  O 1.82329200 -0.74006300 -0.30197700  H 2.70437300 -0.92586200 0.03403000 |
|  | M06-2X | C -2.41444300 -0.14489400 -0.14539900  C -1.01671600 -0.34269200 0.33088500  H -3.26055500 -0.49058200 0.43560900  H -2.59883600 0.16541400 -1.16854300  C -0.05780000 0.73302800 -0.18949900  H -0.99079500 -0.35640700 1.42861600  H -0.63077900 -1.32264500 0.00783800  C 1.37530000 0.50434300 0.25495200  H -0.08525500 0.74900400 -1.28623800  H -0.38437900 1.72041800 0.15829800  H 2.01235500 1.32840200 -0.09482200  H 1.42322700 0.47735400 1.35428800  O 1.81098000 -0.73182400 -0.29258100  H 2.70913200 -0.91508400 -0.00002900 |
